# Engineering DszC Mutants from Transition State Macrodipole Considerations and Evolutionary Sequence Analysis

**DOI:** 10.1021/acs.jcim.2c01337

**Published:** 2022-12-19

**Authors:** Rui P. P. Neves, Maria J. Ramos, Pedro A. Fernandes

**Affiliations:** LAQV, REQUIMTE, Departamento de Química e Bioquímica, Faculdade de Ciências, Universidade do Porto, Rua do Campo Alegre, s/n, 4169-007 Porto, Portugal

## Abstract

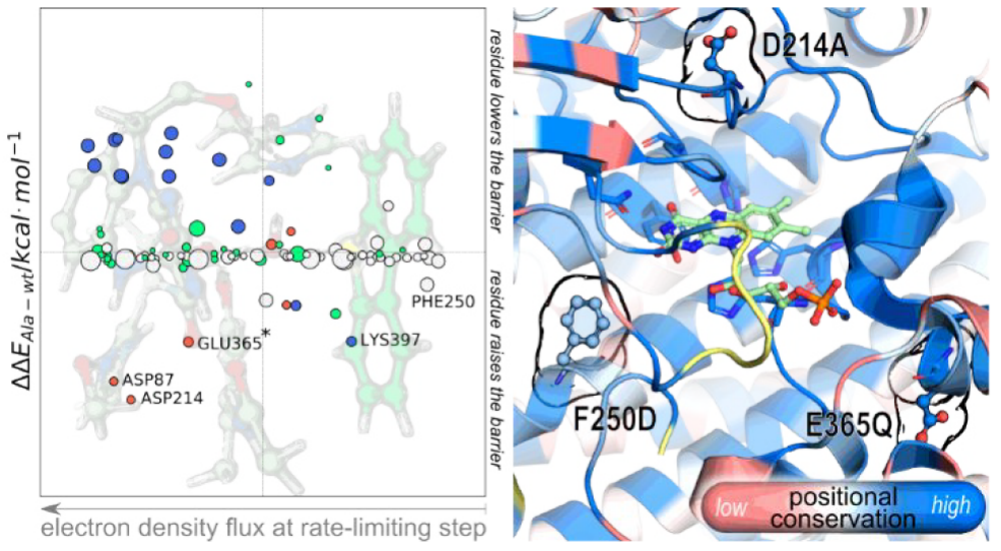

We describe an approach to identify enzyme mutants with increased turnover using the enzyme DszC as a case study. Our approach is based on recalculating the barriers of alanine mutants through single-point energy calculations at the hybrid QM/MM level in the wild-type reactant and transition state geometries. We analyze the difference in the electron density between the reactant and transition state to identify sites/residues where electrostatic interactions stabilize the transition state over the reactants. We also assess the insertion of a unit probe charge to identify positions in which the introduction of charged residues lowers the barrier.

Enzymes are increasingly regarded as the future of catalysts. By 2030, the enzyme market is expected to be worth over 20 billion dollars, and about 40% of the catalyzed chemical syntheses are expected to use enzymes.^1^ Enzymes can operate under mild conditions, with low energy requirements, can be easily synthesized, and are fully biodegradable. However, enzyme efficiencies rarely meet the standards required for industrial use and satisfy the fast worldwide demand for newer and cleaner products and technologies. As such, the interest in the engineering of enzyme efficiency and specificity has been growing steadily.^2−5^

The recent successes of enzyme directed evolution led to a worldwide recognition of the field, being often regarded as state-of-the-art for enzyme engineering.^6^ However, despite its successes, it is still more successful at tuning highly promiscuous enzymes into enzymes with high specificity or enzymes that catalyze reactions nonexistent in the biochemical repertoire^7−9^ than evolving enzymes into near-native or native kinetics. Directed evolution is also highly laborious and often leads to efficiency dead-ends^10^ due to efficiency/stability trade-offs.

Alternatively, rational strategies may assist directed evolution and improve its chances of success by dragging it out from dead ends or ultimately replacing it, leading to less laborious protocols and more efficient management of wet lab resources.^3,4^ Rational strategies focus on studying the underlying molecular determinants behind enzyme efficiency. However, rationalizing enzyme efficiency is tricky in practical terms, as current physics-based methods and models struggle to reproduce the complexity of enzyme folding and oligomerization, substrate binding, or enzyme reactivity accurately. Consequently, so-called semirational strategies, which combine directed evolution and the knowledge drawn from rational approaches, namely structure–sequence analysis or coevolutionary analysis, to quickly outline libraries of mutations that can proceed for high-throughput screenings of enzyme mutants, have become increasingly popular.^3,11,12^ Several software and Web servers built out of algorithms established from empirical, data-driven, or machine-learning models have also been made available, and they have been finding wide application in the search for novel enzymes with industrial use.^1,13−18^

## The Case of Sulfur Oxidation by the Enzyme DszC

Here we combine QM/MM calculations and a multisequence and coevolutionary analysis to identify enzyme mutants with increased reaction rates. As a case study, we focused on the flavin-dependent DszC oxidoreductase, which catalyzes the double oxidation of heteroaromatic sulfur compounds. The enzyme is the first of the bacterial 4S metabolic pathway, which is under study for application in crude oil green desulfurization to replace the hydrodesulfurization method currently used in the oil refining industry. DszC is one of the least efficient enzymes in the 4S-pathway (*k*_cat_/*K*_M_ of 1.3 μM^–1^ min^–1^), mainly due to its low *k*_cat_ of 1.6 ± 0.3 min^–1^.^19^ As such, the engineering of DszC is a desirable way to improve the efficiency of the 4S pathway. Up to now, the engineering of DszC has modestly improved its activity (20-fold),^20−22^ which is still insufficient given that a 500-fold increase is desired to meet industrial requirements. Hence, searching for rational ways to engineer DszC could represent a promising alternative to past attempts.^23,24^

We recently studied the reaction mechanism of oxidation of dibenzothiophene (DBT), the principal substrate of DszC and one of the most recalcitrant organosulfur compounds in crude oil, using hybrid QM/MM methods.^25^ Here, we analyze the contribution of residues around its active site for the barrier of the rate-limiting step of the reaction mechanism of DszC and identify specific mutations that are expected to increase its rate.

**Figure 1:**
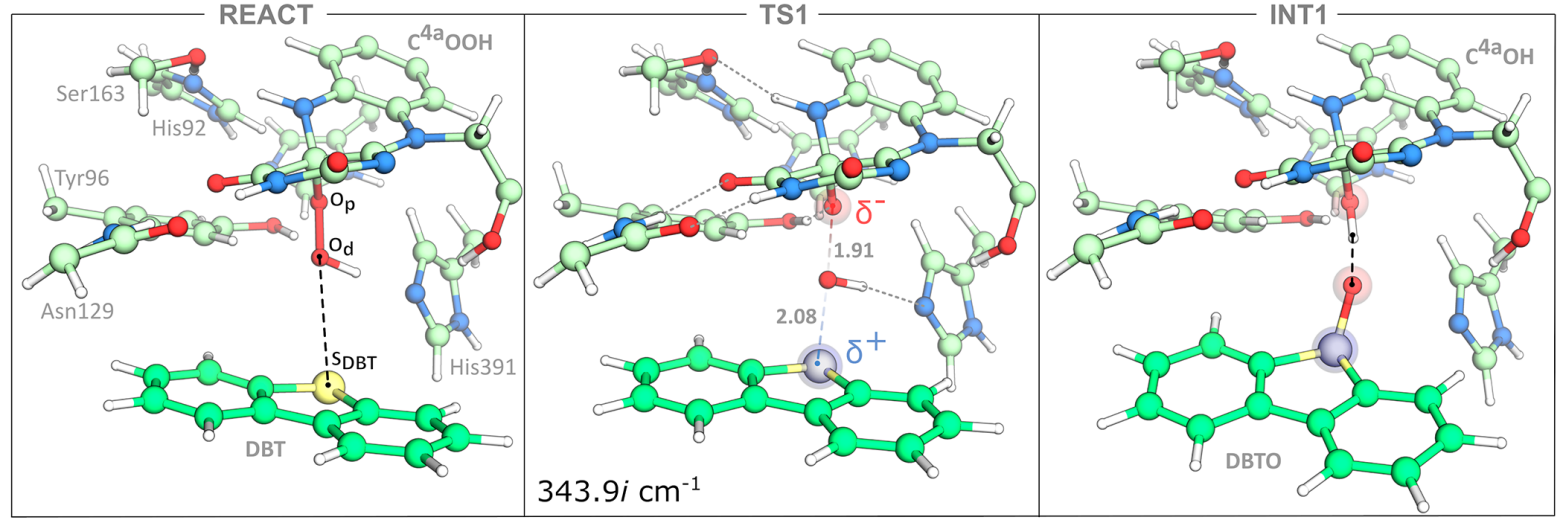
Stationary points of the rate-limiting step of DszC: the oxidation of DBT. The participating atoms are highlighted by transparent spheres (blue for a decrease in electron density and red for an increase in electron density). All distances shown are in Å.

The rate-limiting step of the reaction mechanism corresponds to the oxidation of DBT by a C^4a^-hydroperoxyflavin intermediate (C^4a^OOH), which is formed upon the entrance of molecular oxygen at the active site. This step is shown in Figure 1. It consists of an irreversible nucleophilic S_N_2 substitution with a Gibbs activation energy of 19.7 kcal·mol^–1^, in line with the experimentally determined 20.4 kcal·mol^–1^ obtained from transition-state theory and the experimental enzyme turnover.^19^

The imaginary vibrational frequency of the transition state of DBT oxidation (TS1) indicated a dominant antisymmetric stretching of the O_d_–O_p,_ and O_d_–S_DBT_ bonds, along which the O_d_H transfer occurs.^25^ As there was oxidation of DBT to DBT-sulfoxide (DBTO), a charge analysis confirmed the decrease in electron density in the sulfur of DBT (S_DBT_) upon oxidation and an increase in the electron density of the proximal oxygen (O_p_) at the C^4a^ of the C^4a^OOH intermediate.

## Our Approach to Finding Suitable Candidate Residues for Enzyme Mutagenesis

Based on the charge transfer represented in the TS1 in Figure 1, positively charged residues closer to the O_p_ than the S_DBT_ should stabilize the building negative charge at the O_p_, whereas the insertion of negatively charged residues closer to the S_DBT_ than the O_p_ should stabilize the oxidized DBT-sulfoxide. Both kinds of insertions should thus lower the activation energy of the reaction.

Having that in mind, and recalling other studies discussing the effect of active site polarization on the kinetics of the enzyme,^26,27^ we evaluated the contribution of each residue within 10 Å of the residues composing the active site of DszC (as depicted in REACT in Figure 1) and correlated its contribution with the distance to the atoms where we observe a more significant change in electron density: O_p_ (increase) and S_DBT_ (decrease). These changes in electronic density occur during the oxidation reaction and give rise to a dipole moment at TS1 that was nonexistent at the REACT state. In summary, we expect that positive residues closer to O_p_ than to S_DBT_ and negative residues closer to S_DBT_ than to O_p_ would stabilize the transition state more than the reactants as the O_p_ becomes more negative and the S_DBT_ more positive as the reaction progresses.

All residues with at least one atom within 10 Å of the active site region, other than Gly, Ala, or Pro, were individually mutated by Ala at the REACT and TS1 optimized stationary points of the DBT oxidation reaction, as previously modeled in ref (25); the modeling protocol and optimized coordinates are included in the Supporting Information. At this point, we stress that this approach is not restricted to single stationary points, specifically to the ones modeled from the X-ray structure or closely resembling the X-ray structure. However, in our experience, the latter is likely to best represent the interaction between the enzyme and its native substrate.^28,29^ When this is not the case, or if more sampling is required, the protocol can be applied to several reactant/transition state structures to best represent the conformational diversity of the enzyme:substrate complex.

The mutation by Ala is a means to calculate the side chain contribution to the wild-type barrier, as it conserves the backbone properties and replaces the side chain with the minimal non-hydrogen substituent (methyl). Another option would be to delete the residue (computationally) altogether. Finally, the activation energy differences upon Ala mutation without geometry relaxation reveal the contribution of the residue for the wild-type reaction, which is what we are interested in measuring. The purpose is to quickly identify which wild-type residues are not contributing to lowering the activation energy. In practical terms, after identifying the best candidate residues for mutation through this scheme, one can decide wisely which residue should be used as a replacement.

Alternatively, we repeated the alanine mutagenesis procedure described above, but we also included a unitary ±1.0 probe charge in the geometric center of each mutated side chain for simplicity. We assumed that the geometric center and the center of mass of each residue should be similar because residues are composed mainly of C, N, and O. By doing so, we considered that introducing charged residues through mutation may be beneficial for catalysis, as their insertion modulates the macrodipole at the active site, improving the turnover. The insertion of positively charged residues closer to the O_p_ than the S_DBT_ should stabilize the building negative charge at the O_p_, and the insertion of negatively charged residues closer to the S_DBT_ than the O_p_ should stabilize the oxidized DBT-sulfoxide. Hence, it would be favorable to seek mutations in which noncharged residues could be replaced by charged residues that would stabilize each of these bonds and thus increase enzyme turnover, even knowing that some might be detrimental to the enzyme stability due to the significant structural perturbation that this riskier strategy might bring.

The change in energy between TS1 and REACT was calculated through single-point energy calculations at the ONIOM(B3LYP/6-31G(d):AMBER) level, and the resulting energy difference relative to the activation energy of the DBT oxidation in the wild-type was plotted against the distance to the O_p_ and S_DBT_ atoms. The larger 6-311+G(2d,2p) basis set, combined with the functionals B3LYP, BLYP, mPW1N, and M06-2X, was also tested with similar results (Figures S1–S4), even though the BLYP functional seems to produce exaggerated energy differences when the ±1e probe is placed near catalytic residues. No corrections were made to the entropy, as most studied enzyme-catalyzed reactions are enthalpy-driven,^25,30−32^ unless there was a significant change in interactions at the protein/water interface.^33^ In particular, entropy corrections calculated through the harmonic oscillator-rigid rotor approximation used in adiabatic mapping QM/MM studies systematically led to changes in Gibbs activation energy below 2 kcal·mol^–1^,^25,27,30,34^ well below the 15–20 kcal·mol^–1^ contribution from enthalpy. Therefore, the differential contribution to the entropy is negligible. Implicit solvation models were not considered, as a large part of the enzyme:substrate system (i.e., most intermolecular interactions are present) and waters within 6 Å from the active site were explicitly modeled.

Finally, to predict favorable mutant candidates, we used the ConSurf server^35^ to evaluate the positional conservation of the residues that were the most likely candidates to produce more efficient DszC mutants, as suggested by the computational alanine mutagenesis strategy (the complete results can be consulted in Table S1 in Supporting Information). The ConSurf server estimates the evolutionary conservation of amino/nucleic acid positions in a protein/DNA/RNA molecule based on the phylogenetic relations between homologous sequences.^35^ It is widely accepted that a residue’s positional degree of conservation is strongly related to its structural and functional relevance. Hence, the mutation of a given enzyme residue by others occupying the same position in homologue enzymes is likely to improve the chances of designing functional enzyme mutants.

### Assessment of Activation Energy Differences through Computational Alanine Mutagenesis

**Figure 2:**
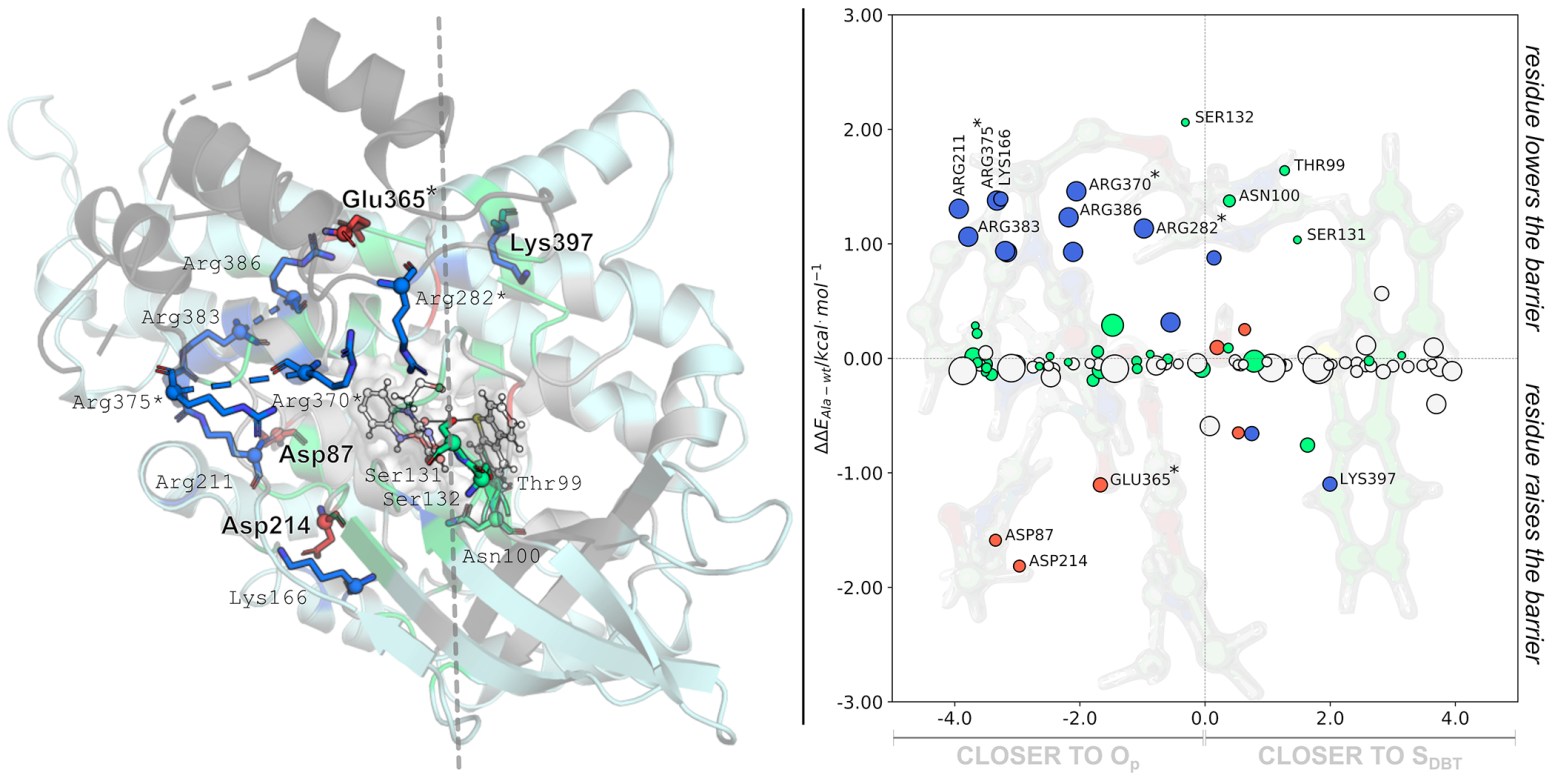
Left, representation of the residues that led to changes in activation energy larger than 1.0 kcal·mol^–1^, with those labeled in bold decreasing the reaction rate (their mutation by alanine decreases the barrier). The C^4a^OOH intermediate and the DBT substrate are highlighted in gray ball-and-stick. The dashed gray line defines the points equidistant to the O_d_ and S_DBT_ atoms used as a reference. Right, activation energy differences upon alanine mutation, as a function of the relative proximity of each residue to the O_p_ and the S_DBT_ atoms. Activation energy differences are calculated relative to the activation energy of the wild-type form at the same level of theory (Δ*E*_mut_^⧧^ – Δ*E*_wt_^⧧^): to measure the distance to charged residues, only the heavy atoms of the charged group were considered; all side chain heavy atoms were considered for other residues. Larger markers represent bulkier amino acids. Residues whose mutation provides a change larger than |1.0 kcal·mol^–1^| in the activation energy are labeled. Residues colored in blue and red correspond to positively and negatively charged residues, and those colored in green and gray correspond to polar and apolar residues. All calculations were performed at the ONIOM(B3LYP/6-31G(d):AMBER) level of theory.

The results of the computational alanine mutagenesis are summarized in Figure 2 and show the distribution of charged (negative and positive), polar, and apolar residues around the S_DBT_ and O_p_ atoms and whether these increase/decrease the activation energy of the reaction.

The left panel of Figure 2 depicts the distribution of the residues around the O_p_ of C^4a^OOH (where electron density increases) and the S_DBT_ of DBT (where electron density decreases), highlighting in bold those whose mutation by Ala led to a decrease in activation energy (higher reaction rate); these are the ones where experimental mutations should be introduced. The right panel plots the activation energy differences calculated for each residue upon mutation by Ala against the relative position of each residue to the O_p_ and S_DBT_ atoms. Since we were particularly interested in the contribution of charged residues for the rate-limiting step, the position of these residues was defined after the center of mass of the heavy atoms in their charged group (carboxyl for Asp and Glu, amine for Lys, and guanidine for Arg), while for the remaining it was defined after the center of mass of the heavy atoms in their side chain.

A few analyzed polar residues (Thr99, Asn 100, Ser131, and 132) lower the wild-type activation energy (Figure 2, right). Despite the abundance of hydrophobic residues, they were not reported to actively contribute to DBT oxidation, although they may still play a significant role in DBT binding.

In agreement with previous studies,^27,36,37^ we observe that positively charged residues close to the O_p_ atom may contribute to a faster DBT oxidation. In contrast, the opposite effect would be expected for negatively charged ones (Figure 2, right). Although few charged residues are observed close to the S_DBT_ atom, where hydrophobic residues are abundant, the three above-mentioned polar residues lowering the activation energy of DBT oxidation are closer to the S_DBT_ than the O_p_ atom. All observed changes in activation energy are between −2 and 2 kcal·mol^–1^. Therefore, an initial enzyme optimization strategy could pass through mutations of negatively charged residues close to the hydroperoxyl group of C^4a^OOH (Asp87, Asp214, Glu365) by noncharged residues with good similarity scores in BLOSUM matrices or those prevalent in sequence alignment analyses of enzymes sharing a similar fold, to minimize the chances of unintended structural destabilization.

The position of residues Asp87, Asp214, Glu365, and Lys 397 is highly conserved in the primary sequence of the proteins considered for the analysis, as summarized in Table 1.

**Table 1:** Results of the ConSurf Server for Residues Whose Mutation by Ala Lowers the Activation Energy^a^

RESIDUE	SCORE (1–9)	VARIABILITY
Asp87	9	Glu
Asp214	8–9	Val, **Gly**, **Ala**, Glu, **Ser**
Glu365*	8–9	Asp, **Gln**
Lys397	9	Arg

a SCORE indicates the degree of conservation of the residue (the higher the SCORE, the higher the conservation of the position of the residues in the primary sequence); VARIABILITY indicates which residues can be found in the same position when considering proteins with a similar fold.

This analysis suggests that Asp214 and Glu365 could be mutated by Gly, Ala or Ser and Gln, respectively, as highlighted in Table 1, with little risk. Nevertheless, mutation of any of the four residues (Asp87, Asp214, Glu365, and Lys 397) by residues with good scores in BLOSUM62 matrices (e.g., Asp87 and Asp214 by Asn, Glu365 and Lys397 by Gln, for example) could also be considered.

### Assessment of Activation Energy Differences upon Insertion of a ±1*e* Probe

In line with the expected trends, the insertion of the ±1*e* probe at the geometric center of residues already bearing the same total unitary charge leads to small changes in the activation energy. In contrast, the insertion of a −1*e* probe in the geometric center of positively charged residues contributes to lowering the activation energy of DszC (Lys166 and Arg211, 383, 386, 282*, 370*, and 375* labeled on the upper-left side of Figure 2) leading to an increase in the activation energy of DBT oxidation by DszC. Fewer hits are observed for residues that could be replaced with negatively charged residues. It should be emphasized that mutation of charged residues by others of opposite charge leads to a significant chance of obtaining nonfunctional DszC mutants, as there is a risk of inducing structurally disruptive changes.

**Figure 3:**
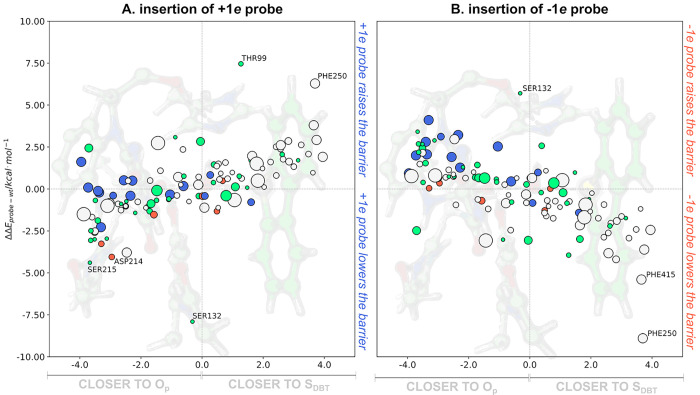
Activation energy differences upon insertion of a unitary probe charge in the center of the side chain of the residues within 10 Å of the active site of DszC, as a function of the distance of the side chain center of mass to the O_p_ and S_DBT_ atoms. Larger markers represent bulkier amino acids. Residues colored in blue and red correspond to positively and negatively charged residues, and those colored in green and gray correspond to polar and apolar residues. All calculations were performed at the ONIOM(B3LYP/6-31G(d):AMBER) level of theory.

In conclusion, after analysis of the results in Figure 3, and although these mutations should be seen as more extreme cases, we suggest that the catalytic rate of DszC could be increased if Ser132, Asp214, or Ser215 are mutated by a positively charged residue or Phe250 or Phe415 are mutated by a negatively charged residue.

Despite that mutation of Ser132 and 215 or Asp214 by positively charged residues could be beneficial for DszC activity from the point of view of the stabilization of the dipole moment of the active site, the evolutionary analysis, summarized in Table 2, indicates that positively charged residues are not found in homologous sequences, which indicates that it might be unlikely that functional DszC mutants result from these mutations. Positively charged residues such as Lys or Arg are also longer and bulkier than Ser or Asp, and such mutations might lead to significant structural changes in DszC. A limiting case could be the mutation of Ser132 by a His (highlighted in bold in Table 2). Histidine interchange between the acid and basic forms can introduce positive charge near the active site. However, such mutation would likely disrupt the tight hydrogen bond that Ser132 establishes with the isoalloxazine ring of the FMN cofactor.

**Table 2:** Results of the ConSurf Server for Residues Whose Mutation by Charged Residues Lower Activation Energy^a^

RESIDUE	SCORE (1–9)	VARIABILITY
Mutation by Positively Charged Residues		
Ser132	9	Ala, **His**, Asn, Gln, Asp
Asp214	9	Val, Gly, Ala, Glu, Ser
Ser215	9	Glu, Gly
Mutation by Negatively Charged Residues		
Phe250	6–7	Leu, Gly, Val, Arg, **Asp**, Thr, Ala, Cys, His, Ile, Ser, Asn, Tyr
Phe415	6–7	Ile, Leu, Gly, Trp, Val, Tyr

a SCORE indicates the degree of conservation of the residue (the higher the SCORE, the higher the positional conservation of the residues); VARIABILITY indicates which residues can be found alternatively to the identified RESIDUE.

Regarding Phe250, it could be mutated by an Asp (highlighted in bold in Table 2), as it happens in homologous structures. However, as for the previous case, these residues are considerably different in bulkiness and may represent a lower chance of expressing nonfunctional forms of DszC unless compensatory mutations are introduced. These aspects can be realized by analyzing the structure of the specific homologue bearing the Phe/Asp mutation.

## Take-Home Message

In summary, the method for enzyme engineering proposed here works within the assumption that the enzyme will not suffer significant structural changes upon introducing the mutations. Even though this holds for some cases only, the traditional methods used nowadays for enzyme engineering have a high failure rate; thus, it is acceptable to accommodate the risk here. Furthermore, our fast and straightforward screening approach based on QM/MM energy calculations can be easily parallelized and provide candidate residues for mutation more rationally than conventional methods such as directed evolution, complementing experimental enzyme engineering.

## Data Availability

Gaussian input files and PDB files for the stationary points of DBT oxidation, and the scripts required to write the alanine mutants (gau_prep-mutation.sh) and introduce unitary charge probes (gau_addprobe.py) are available in the Supporting Information. The AmberTools 18 package required to parametrize the alanine mutants (*xleap* module) and perform data collection (*cpptraj* module) is available free of charge, upon registration, at https://ambermd.org/GetAmber.php#ambertools; required parameters for ligands and cofactors are also included in the Supporting Information (ligand_params folder). In-house python scripts were developed using Python 2.7.18, available free of charge at https://www.python.org/downloads/release/python-2718, and the python modules: *pandas* 0.24.2 (https://pypi.org/project/pandas/0.24.2), *numpy* 1.14.0 (https://pypi.org/project/numpy/1.14.0), and *matplotlib* 2.2.5 (https://matplotlib.org/2.2.3/contents.html). ONIOM calculations were performed with the Gaussian 16 software, which is a licensed software that is available for purchase at https://gaussian.com/pricing. The ConSurf Web server is available free of charge at https://consurf.tau.ac.il/consurf_index.php; the results are included in Supporting Information.

## Supporting Information Available

Modeling of the QM/MM model of the DszC:DBT complex, auxiliary plots for the differences in activation energy upon alanine mutagenesis of residues around the active site and upon insertion of unit charge probes around the active site with varying density functionals and basis sets and summary table of the multisequence analysis performed on the DszC with the ConSurf server (PDF)
Files required to prepare Gaussian inputs for the QM/MM calculations (ZIP)
Outputs of the ConSurf server for the DszC enzyme (ZIP)

## Abbreviations

QM/MM: quantum mechanics/molecular mechanics
ONIOM: Our own *N*-layered Integrated molecular Orbital and Molecular mechanics
FMN: flavin mononucleotide
