# Supplementary material for: Engineering DszC Mutants from Transition State Macrodipole Considerations and Evolutionary Sequence Analysis
Source: J Chem Inf Model. 2022 Dec 19;63(1):20–6. doi: 10.1021/acs.jcim.2c01337 (PMC9832474; doi:10.1021/acs.jcim.2c01337)
Supplement: Supplementary file 1 — ci2c01337_si_001.pdf [file ci2c01337_si_001.pdf]

Supporting Information

Engineering DszC mutants from transition state macrodipole considerations and evolutionary sequence analysis

*Rui P. P. Neves, Maria J. Ramos, Pedro A. Fernandes**

LAQV, REQUIMTE, Departamento de Química e Bioquímica, Faculdade de Ciências, Universidade do Porto, Rua do Campo Alegre, s/n, 4169-007 Porto, Portugal

*E-mail for P.A.F.: pafernan@fc.up.pt.

INDEX

MODELING OF THE QM/MM MODEL OF THE DSZC:DBT COMPLEX 2

TRANSITION STATE MACRODIPOLE CALCULATIONS WITH DIFFERENT DENSITY FUNCTIONALS AND BASIS SETS 3

POSITIONAL CONSERVATION SCORES OBTAINED FROM THE CONSURF SERVER 8

REFERENCES 16

Modeling of the QM/MM model of the DszC:DBT complex

The DszC:DBT complex was modelled in the catalytically-competent homodimeric form (chain A and F, with 417 residues each, from PDB ID 3X0Y with 2.3 Å resolution), in complex with the FMN cofactor in the oxidized form and with all X-ray waters within 6 Å of each catalytic site.[1] The FMN cofactor was modelled as C^{4a}-hydroperoxyflavin intermediate ($C^{4a}OOH$), as there was a well-defined electronic density for FMN in the active site of DszC that suggested a tight-binding of FMN to the active site of DszC. The binding pose of DBT was modelled as in the TdsC homolog enzyme from *Paenibacillus sp.* (64% identity and identical active site residues) binding oxidized FMN and DBT (PDB ID: 5XDE),[2] after aligning the conserved FMN cofactor and the backbone of catalytic residues His92, Ser163, His391 of both DszC and TdsC. As predicted by the Propka3.1 program,[3] physiological protonation states were attributed to all residues except His92, His388 and His391: His92 and His388 were protonated as δ-tautomers and His391 was doubly-protonated as in the imidazolium form.

The DszC homodimer was described with the ff10 force field[4], whereas DBT and $C^{4a}OOH$ were parameterized with the GAFF[5] and with RESP charges derived after geometry optimization of each molecule at the HF/6-31G* level – using the Antechamber module available in AMBER12 and the Gaussian software for QM calculations[6]. The Xleap module of AMBER12 was used to add 33 Na^+ ions to counteract the overall positive charge of the system, and to add TIP3P waters[7] to fill a rectangular box with faces at a minimum distance of 12 Å from the solute. Afterwards, a four-step molecular mechanics (MM) energy minimization protocol was applied to remove bad contacts in the system: (1) positional harmonic restraints of 50 $kcal{\cdot}mol^{-1}$ were applied to water molecules; (2) positional harmonic restraints of 50 $kcal{\cdot}mol^{-1}$ were applied to all heavy atoms; (3) positional harmonic restraints of 10 $kcal{\cdot}mol^{-1}$ were applied backbone atoms of the solute; and (4) no restraints were applied.

The QM/MM model proceeding for ONIOM calculations was taken from the final minimized structure of the MM protocol. It comprised one monomer binding $C^{4a}OOH$ and DBT, all residues of the second monomer within a radius of 18 Å from the His92, Tyr96, Asn129, Ser163, His388 and His391, $C^{4a}OOH$ and DBT in the first monomer, and the water molecules at least 6 Å away from the catalytic site – in a total of 7238 atoms and total charge of -15. The QM layer included all atoms of DBT, the isoalloxazine ring (except for the two methyl groups in the phenyl moiety), the N^{10}-bound hydroxymethyl of the ribitol tail of $C^{4a}OOH$, and the side chains of residues His92, Tyr96, Asn129, Ser163, His388 and His391 – a total of 112 atoms, zero total charge and singlet multiplicity – whereas the MM layer comprised all the remaining atoms of the model. All atoms beyond a radius of 15 Å from the molecules composing the QM layer were kept rigid throughout the ONIOM calculations. All ONIOM calculations were ran with the Gaussian software.

All QM/MM calculations were carried out at the B3LYP/6-31G(d):ff10 level of theory,[8-16] with the Coulomb QM/MM interactions calculated with the electrostatic embedding scheme, as implemented in Gaussian 09.[6] The B3LYP/6-31G(d) level has been successfully employed in other QM/MM studies of the catalytic mechanism of enzymes.[17-20] In particular, to study the triplet-singlet spin inversion taking place upon formation of the $C^{4a}OOH$, we allowed break of the symmetry of the singlet configuration by accepting the inclusion of imaginary coefficients and/or mixed-spin orbitals in the wavefunction.[21-23] We used hydrogens as link atoms to complete the valence of the bonds in the boundary of the two layers.[24-27]

Transition state macrodipole calculations with different density functionals and basis sets

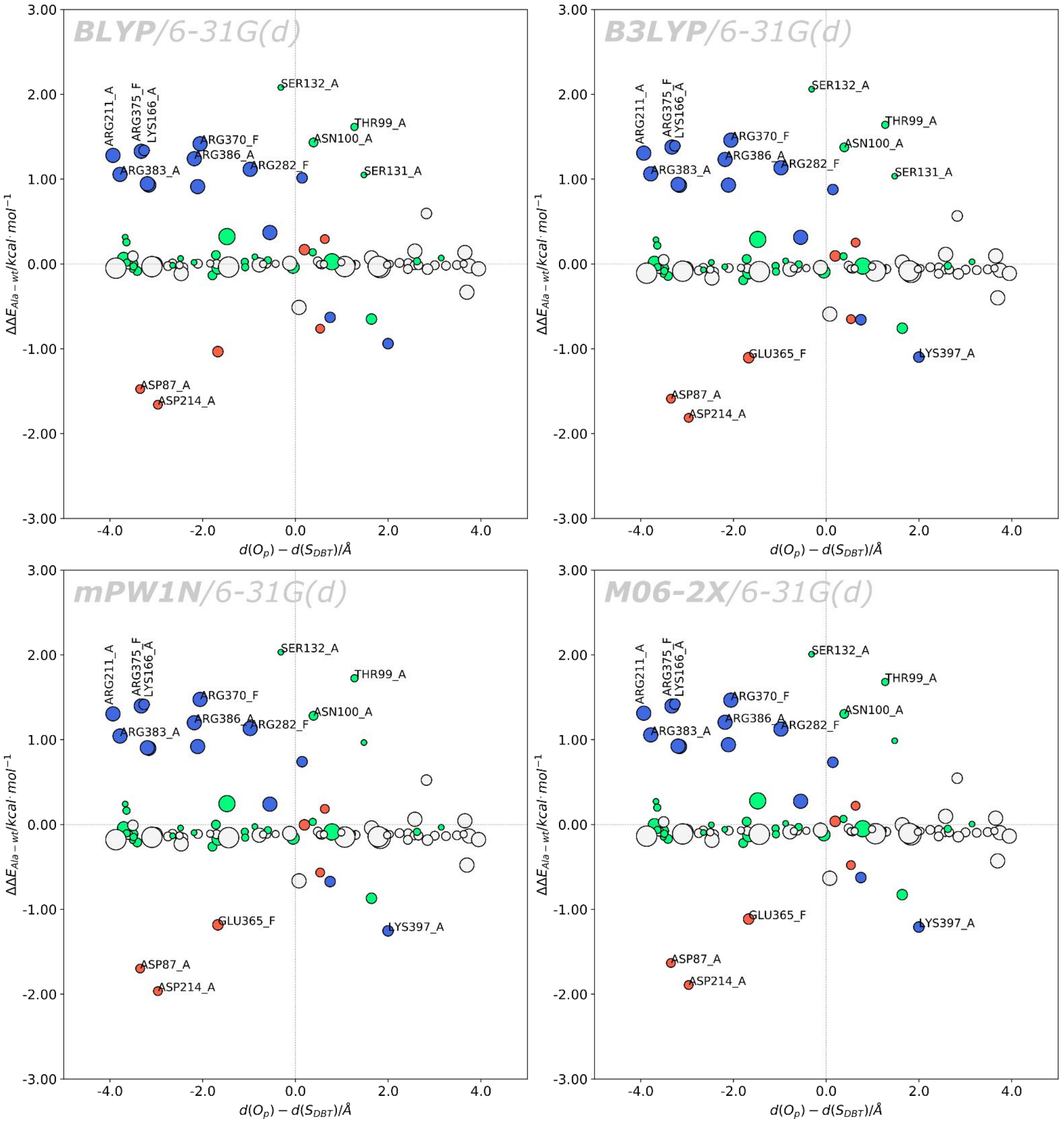

Figure S1. Activation energy differences upon alanine mutation for different density functionals (BLYP, B3LYP, mPW1N and M06-2X) as a function of the relative proximity of each residue to the O_p and the S_{DBT} atoms. Activation energy differences are calculated relatively to the activation energy of the wild-type form at the same level of theory; to determine the representative position of charged residues, only the heavy atoms of the charged group were considered to describe the position of the residue relatively to the active site, whereas the heavy atoms of the whole sidechain were considered for the remaining residues. Larger markers represent bulkier amino acids. Residues whose mutation provides a change larger than |1.0 kcal·mol-1| in the activation energy are labelled. Residues colored in blue and red correspond to positively and negatively charged residues, and those colored in green and gray correspond to polar and apolar residues. All calculations were performed at the ONIOM(DFT/6-31G(d):AMBER) level of theory.

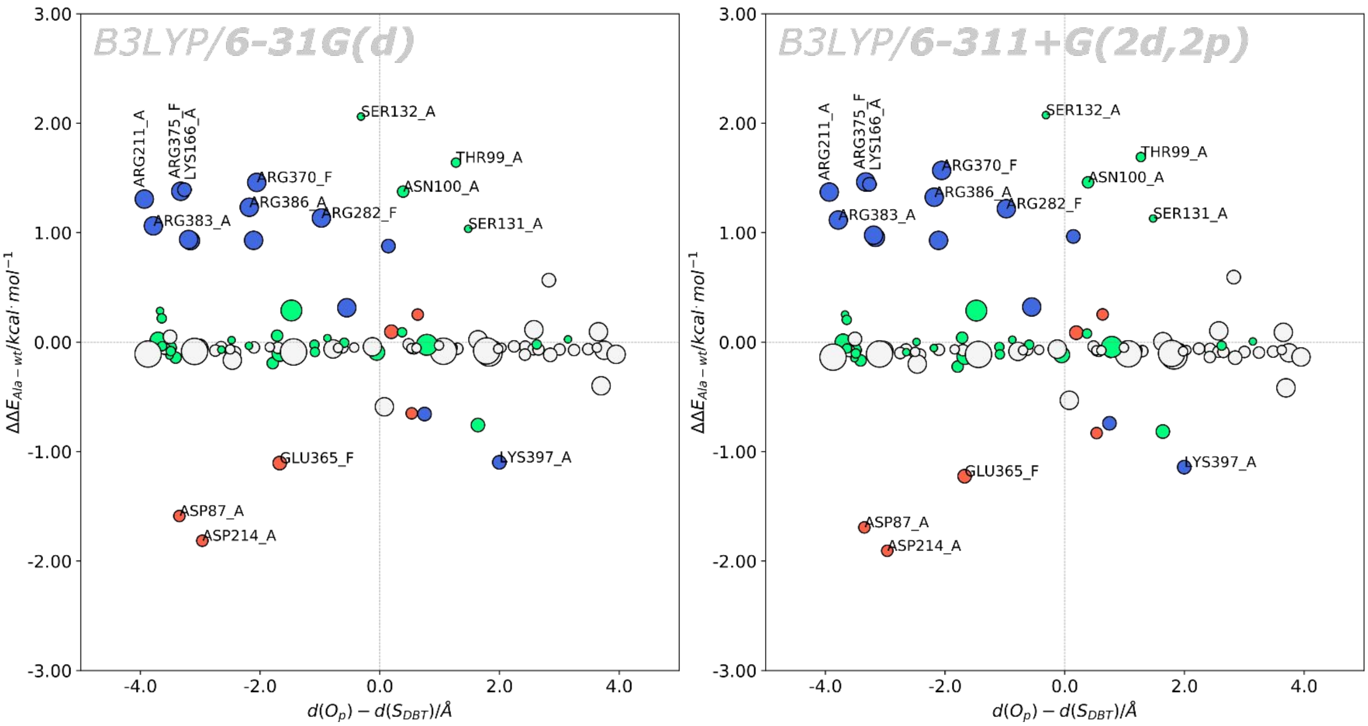

Figure S2. Activation energy differences upon alanine mutation for different basis sets (6-31G(d) and 6-311+G(2d,2p)) as a function of the relative proximity of each residue to the O_p and the S_{DBT} atoms. Activation energy differences are calculated relatively to the activation energy of the wild-type form at the same level of theory; to determine the representative position of charged residues, only the heavy atoms of the charged group were considered to describe the position of the residue relatively to the active site, whereas the heavy atoms of the whole sidechain were considered for the remaining residues. Larger markers represent bulkier amino acids. Residues whose mutation provides a change larger than |1.0 kcal·mol-1| in the activation energy are labelled. Residues colored in blue and red correspond to positively and negatively charged residues, and those colored in green and gray correspond to polar and apolar residues. All calculations were performed at the ONIOM(B3LYP:AMBER) level of theory.

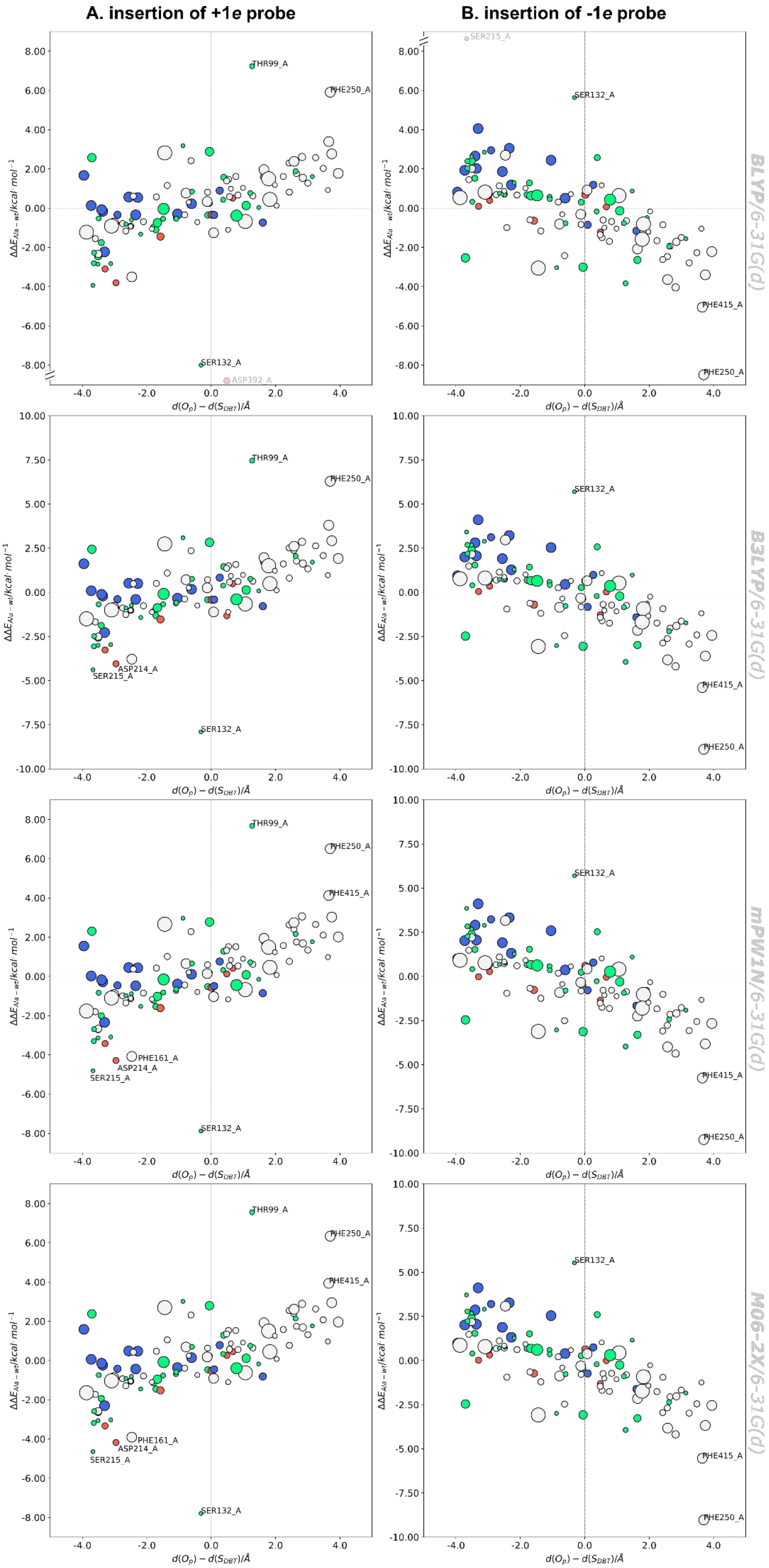
A. insertion of +1e probe
B. insertion of -1e probe
BLYP/6-31G(d)
B3LYP/6-31G(d)
mPW1N/6-31G(d)
M06-2X/6-31G(d)
$\Delta\Delta E_{Ala-wt}/kcal \cdot mol^{-1}$
$d(O_p) - d(S_{DBT})/Å$
THR99_A
PHE250_A
PHE415_A
SER132_A
ASP392_A
SER215_A
ASP214_A
PHE161_A

Figure S3. Activation energy differences for different density functionals (BLYP, B3LYP, mPW1N and M06-2X) upon insertion of a unitary probe charge in the geometric centre of the sidechain of the residues within 10 Å of the active site of DszC, as a function of the distance of the sidechain centre of mass to the O_p and S_{DBT} atoms. Larger markers represent bulkier amino acids. Since most residues are composed of C, N and O, we assumed for simplicity that the geometric center of each sidechain should closely resemble their centre of mass. Residues in which the probe insertion leads to a change larger than 50% of the maximum energy difference relative to the activation energy are labelled. Residues colored in blue and red correspond to positively and negatively charged residues, and those colored in green and gray correspond to polar and apolar residues.

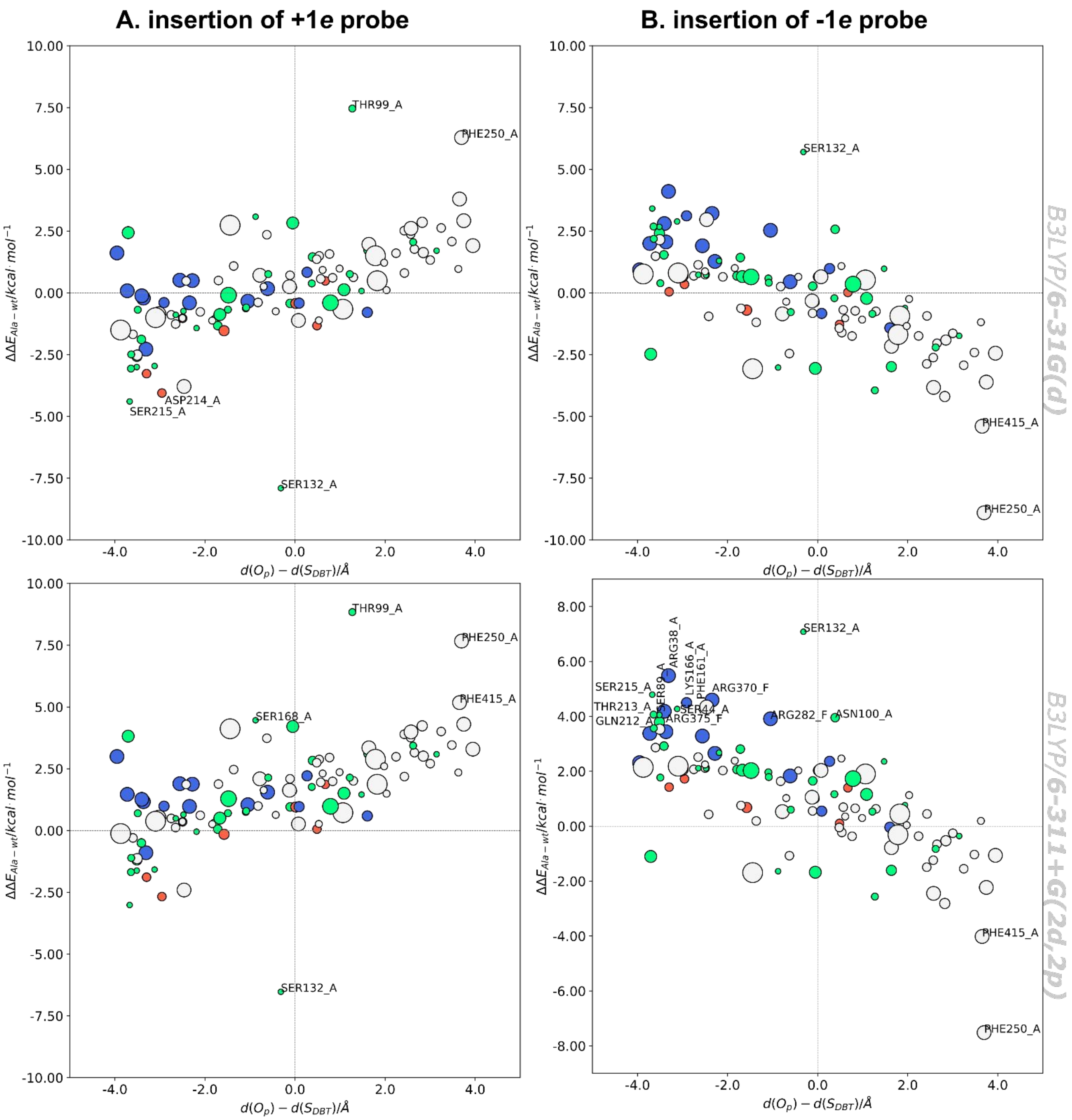

Figure S4. Activation energy differences for different basis sets (6-31G(d) and 6-311+G(2d,2p)) upon insertion of a unitary probe charge in the geometric centre of the sidechain of the residues within 10 Å of the active site of DszC, as a function of the distance of the sidechain centre of mass to the O_p and S_{DBT} atoms. Larger markers represent bulkier amino acids. Since most residues are composed of C, N and O, we assumed for simplicity

that the geometric center of each sidechain should closely resemble their centre of mass. Residues in which the probe insertion leads to a change larger than 50% of the maximum energy difference relative to the activation energy are labelled. Residues colored in blue and red correspond to positively and negatively charged residues, and those colored in green and gray correspond to polar and apolar residues.

Positional conservation scores obtained from the ConSurf server

	pos	ALA	CYS	ASP	GLU	PHE	GLY	HIS	ILE	LYS	LEU	MET	ASN	PRO	GLN	ARG	SER	THR	VAL	TRP	TYR	MAX AA	ConSurf Grade
**MET**	**1**											100.0										M 100.000	6*
**THR**	**2**																	100.0				T 100.000	8*
**LEU**	**3**										100.0											L 100.000	7*
**SER**	**4**																40.0	60.0				T 60.000	7*
**PRO**	**5**													80.0					20.0			P 80.000	6*
**GLU**	**6**	20.0			40.0									20.0				20.0				E 40.000	3*
**LYS**	**7**			20.0				20.0		40.0						20.0						K 40.000	3*
**GLN**	**8**					20.0									40.0			20.0		20.0		Q 40.000	1*
**HIS**	**9**							50.0							16.7		16.7	16.7				H 50.000	4*
**VAL**	**10**			12.5			12.5				12.5							25.0	37.5			V 37.500	2*
**ARG**	**11**	20.0					10.0			10.0						40.0	10.0	10.0				R 40.000	2*
**PRO**	**12**	16.7		8.3										66.7				8.3				P 66.667	5
**ARG**	**13**	25.0		12.5			6.3							6.3	6.3	25.0		12.5			6.3	AR 25.000	1
**ASP**	**14**	18.2		9.1		4.5	4.5				9.1		4.5	36.4	4.5	4.5		4.5				P 36.364	1
**ALA**	**15**	53.3		3.3	3.3		3.3	3.3						10.0		6.7	16.7					A 53.333	5
**ALA**	**16**	20.5		28.2	7.7		2.6								2.6	7.7	7.7	20.5	2.6			D 28.205	3
**ASP**	**17**	12.2		34.1	4.9	2.4	2.4	4.9			7.3		2.4	4.9	4.9	7.3	4.9	7.3				D 34.146	1
**ASN**	**18**	32.1		9.4	5.7	3.8				5.7			9.4	7.5	9.4	1.9	5.7	5.7	1.9		1.9	A 32.075	1
**ASP**	**19**	4.7		40.6	20.3		4.7	3.1					4.7	3.1	3.1	1.6	6.3	7.8				D 40.625	4
**PRO**	**20**	25.4				1.5		1.5	3.0		3.0			29.9			1.5		1.5	26.9	6.0	P 29.851	3
**VAL**	**21**	1.4		1.4		2.7			20.5		57.5		1.4		1.4	1.4			9.6	2.7		L 57.534	2
**ALA**	**22**	52.1		6.4	6.4		1.1	1.1		1.1	2.1		1.1	2.1	9.6	8.5	3.2	3.2	2.1			A 52.128	4
**VAL**	**23**	8.9		1.0					17.8	5.0	4.0	1.0		1.0	3.0	15.8	4.0	9.9	28.7			V 28.713	3
**ALA**	**24**	84.2				0.9			0.9		4.4							1.8	7.9			A 84.211	8
**ARG**	**25**	16.4		12.3	13.9		3.3	3.3	1.6	3.3					11.5	26.2	2.5	4.1	1.6			R 26.230	1
**GLY**	**26**	19.4		11.6	27.9	1.6	2.3	0.8		3.1	3.1				8.5	9.3	10.9	1.6				E 27.907	1
**LEU**	**27**	3.7				0.7			3.0		56.3	0.7						2.2	33.3			L 56.296	7
**ALA**	**28**	74.6	0.7				0.7		3.6		2.2					6.5	6.5	1.4	3.6			A 74.638	7
**GLU**	**29**	36.7		18.0	17.3		0.7	0.7		1.4	0.7		1.4	2.2	10.1	4.3	2.2	2.9	1.4			A 36.691	1
**LYS**	**30**	12.7		8.5	21.8		1.4	1.4	7.7	4.2	2.8		0.7		7.0	12.7	2.1	8.5	7.7		0.7	E 21.831	1
**TRP**	**31**	0.7				33.8			2.1		61.3									2.1		L 61.268	7
**ARG**	**32**	59.4		1.4	2.8		0.7	2.1		0.7					0.7	22.4	4.2	3.5	0.7		1.4	A 59.441	6
**ALA**	**33**	34.2		0.7	10.3				1.4	6.8	6.2			4.1	4.8	9.6	3.4	8.9	9.6			A 34.247	1
**THR**	**34**			32.7			7.5						2.7			4.1	8.8	44.2				T 44.218	7
**ALA**	**35**	93.9					1.4		0.7		0.7				0.7				2.7			A 93.878	9
**VAL**	**36**	35.6	0.7	0.7	0.7		1.3		2.0		7.4		1.3	4.0			4.7	0.7	40.9			V 40.940	4
**GLU**	**37**	22.8		4.7	51.0		0.7	1.3		2.0	2.7				5.4	1.3	0.7	4.0	3.4			E 51.007	3
**ARG**	**38**										4.7					95.3						R 95.302	9
**ASP**	**39**			92.6	7.4																	D 92.617	9
**ARG**	**40**	9.4			4.0			2.7	1.3	13.4	1.3				14.8	51.0	0.7	0.7			0.7	R 51.007	4
**ALA**	**41**	41.6		2.7	16.8		0.7	0.7	0.7	4.7	5.4				10.7	10.7	1.3	2.7	1.3			A 41.611	3
**GLY**	**42**	3.4		0.7			66.4				0.7		19.5		0.7	8.1		0.7				G 66.443	7
**GLY**	**43**	14.8			0.7		52.3		0.7	12.1	4.0				3.4	10.7		0.7	0.7			G 52.349	6
**SER**	**44**	6.0		2.7	2.7			16.8	0.7		8.7		4.0	9.4	1.3	5.4	9.4	30.9	1.3		0.7	T 30.872	6
**ALA**	**45**	27.5												72.5								P 72.483	8
**THR**	**46**	14.1	0.7	1.3	1.3	8.7		4.0		20.8	10.1				1.3	16.1	2.7	6.0	6.7	1.3	4.7	K 20.805	1
**ALA**	**47**	47.7		10.7	9.4	2.0	1.3	3.4	2.0	3.4	1.3		1.3	1.3	4.0	4.7	2.0	2.0	2.0		1.3	A 47.651	1
**GLU**	**48**	2.0		0.7	89.9						0.7				6.7							E 89.933	9
**ARG**	**49**	2.7							20.8	6.0	9.4					38.3			22.8			R 38.255	7
**GLU**	**50**	14.8		26.8	22.1		2.0		1.3	0.7			1.3		14.8	8.1	4.0	1.3	2.7			D 26.846	3
**ASP**	**51**	10.7		1.3	1.3			1.3	0.7	4.0	54.4	1.3			1.3	15.4	0.7	0.7	0.7	6.0		L 54.362	4
**LEU**	**52**					2.7			23.5		72.5	0.7							0.7			L 72.483	7
**ARG**	**53**	4.7								25.5						61.7	6.7	1.3				R 61.745	8
**ALA**	**54**	20.8		20.1	18.1		2.0	8.7		3.4			4.0		14.8	4.7	2.0	0.7			0.7	A 20.805	1

Table S1. Per residue variety for each position in the sequence of DszC, after the multi-sequence analysis performed by the ConSurf server

**SER**	**55**	22.1			0.7		0.7	3.4			1.3		1.3		1.3		67.8		0.7		0.7	S 67.785	7
**GLY**	**56**			4.0			94.6			0.7						0.7						G 94.631	9
**LEU**	**57**										100.0											L 100.000	9
**LEU**	**58**	0.7					0.7				81.2		1.3	2.0	0.7			2.7	9.4	1.3		L 81.208	8
**SER**	**59**	18.9		2.7	1.4		7.4	0.7		10.1	1.4		7.4	3.4	3.4	4.1	12.2	26.4			0.7	T 26.351	2
**LEU**	**60**	8.1				1.4			5.4		68.9	0.7							15.5			L 68.919	7
**LEU**	**61**	17.8				0.7	2.7		4.8		33.6	1.4			2.7		21.9	11.0	2.7	0.7		L 33.562	6
**VAL**	**62**	2.7		1.4	4.1	0.7	14.4	4.8	38.4		2.7		0.7				0.7	2.7	26.7			I 38.356	6
**PRO**	**63**	6.8					0.7				0.7			89.7			1.4			0.7		P 89.726	8
**ARG**	**64**	22.6	0.7		3.4		3.4	0.7		17.1			0.7	6.2	2.7	21.2	0.7	10.3	10.3			A 22.603	2
**GLU**	**65**	21.9	0.7	3.4	47.9		0.7	0.7		6.2			0.7	0.7	10.3	3.4	2.1	0.7		0.7		E 47.945	3
**TYR**	**66**	2.1			0.7	21.9		21.2	4.8		9.6		0.7				0.7		2.1	1.4	34.9	Y 34.932	2
**GLY**	**67**			0.7			98.6								0.7							G 98.630	9
**GLY**	**68**			0.7			99.3															G 99.315	9
**TRP**	**69**	19.2		2.7	4.8	0.7	26.7	4.8	0.7		13.0	0.7	0.7	0.7	13.0	1.4	3.4	1.4	0.7	3.4	2.1	G 26.712	1
**GLY**	**70**	2.1		6.8	11.6		76.0			0.7			0.7		1.4		0.7					G 76.027	5
**ALA**	**71**	40.5			7.4		8.1	1.4	0.7	0.7	14.2				17.6		0.7	0.7	7.4	0.7		A 40.541	5
**ASP**	**72**	2.0		26.2	2.7		6.0	2.0					9.4	15.4	0.7	7.4	20.8	7.4				D 26.174	1
**TRP**	**73**	4.0				2.7		1.3			4.0			0.7	1.3			0.7	0.7	79.2	5.4	W 79.195	7
**PRO**	**74**	13.4		3.4	5.4		2.7	2.0	2.7	2.0	4.7			20.1	8.1	3.4	18.1	5.4	8.7			P 20.134	1
**THR**	**75**	3.4		12.1	11.4	0.7			2.7		7.4		0.7		10.7	1.3	2.7	38.3	8.7			T 38.255	4
**ALA**	**76**	30.2				2.0	9.4		17.5		10.7	0.7			1.3		2.0	16.1	10.1			A 30.201	6
**ILE**	**77**	7.4	0.7			11.4		2.7	1.3		35.6	2.0	0.7		4.0		1.3	1.3	1.3		30.2	L 35.570	4
**GLU**	**78**	10.1		4.0	17.5		3.4	4.0		14.8	2.7		1.3		13.4	26.8	1.3	0.7				R 26.846	2
**VAL**	**79**	10.7	0.7				1.3	1.3	25.5		8.7							8.7	43.0			V 42.953	4
**VAL**	**80**	1.3				6.0	1.3		26.2		4.0		0.7				3.4	7.4	49.7			V 49.664	5
**ARG**	**81**	1.3					0.7			0.7					3.4	93.3		0.7				R 93.289	9
**GLU**	**82**	5.4			31.5		0.7	0.7	18.1	2.0	8.7				4.0	21.5		4.7	2.7			E 31.544	3
**ILE**	**83**					18.8			30.9		28.2	2.0							20.1			I 30.872	5
**ALA**	**84**	88.6					3.4										8.1					A 88.591	9
**ALA**	**85**	22.1			4.0					18.8					8.7	36.9	5.4	3.4	0.7			R 36.913	4
**ALA**	**86**	40.3					19.5				0.7						1.3	5.4	32.9			A 40.268	6
ASP	87			95.3	4.7																	D 95.302	9
**GLY**	**88**	8.1					36.2							3.4			47.0	5.4				S 46.980	8
**SER**	**89**	18.8											2.0				79.2					S 79.195	9
**LEU**	**90**	0.7				0.7			43.0		40.9	0.7					0.7		13.4			I 42.953	6
**GLY**	**91**	61.7					38.3															A 61.745	8
**HIS**	**92**							59.7				6.7			33.6							H 59.732	9
**LEU**	**93**								9.4		65.1								25.5			L 65.101	7
**PHE**	**94**					29.5			3.4		44.3			8.1					2.7		12.1	L 44.295	6
**GLY**	**95**	19.5					66.4				1.3				8.1		3.4	0.7	0.7			G 66.443	6
**TYR**	**96**		2.0			36.9		0.7					6.0				5.4				49.0	Y 48.993	8
**HIS**	**97**							82.6					0.7		15.4			1.3				H 82.550	8
**LEU**	**98**		2.7			16.1		35.6	1.3		10.7				1.3			0.7	0.7		30.9	H 35.570	6
**THR**	**99**	5.4				3.4	2.0	0.7	0.7		65.8		1.3				4.7	2.0	13.4		0.7	L 65.772	6
**ASN**	**100**		2.0				3.4	4.0			13.4	11.4	12.1	4.0	26.8		2.0			14.8	6.0	Q 26.846	6
**ALA**	**101**	6.0			0.7	2.7	3.4		10.1		28.9	0.7	1.3		3.4		17.5	3.4	22.1			L 28.859	6
**PRO**	**102**	34.2		0.7	0.7	0.7	2.0	2.0	4.0	0.7	1.3		8.1	1.3	7.4	4.0	0.7	8.1	9.4	14.1	0.7	A 34.228	5
**MET**	**103**	9.4				0.7	4.7	0.7	7.4		3.4	2.0	6.0			4.0	15.4	30.2	8.1	3.4	4.7	T 30.201	6
**ILE**	**104**	22.1				0.7	2.0		18.1		7.4	0.7		9.4			2.7	6.0	30.9			V 30.872	4
**GLU**	**105**	4.0		5.4	6.0		4.7	8.7	2.0		10.7	0.7	6.0		7.4	30.2	3.4	2.0	2.7		6.0	R 30.201	2
**LEU**	**106**	1.3				9.4	0.7		10.7		51.0	0.7				6.7		1.3	10.7	7.4		L 51.007	6
**ILE**	**107**	6.7			0.7	36.9	1.3	0.7	0.7		5.4	0.7			6.0	4.7	2.7	1.3	16.1	4.7	11.4	F 36.913	5
**GLY**	**108**	15.3		2.0	1.3	0.7	70.7		0.7		2.0				0.7	0.7	4.7	0.7	0.7			G 70.667	5
**SER**	**109**	3.4		5.4	4.0		3.4		0.7	1.3			7.4	0.7	4.0	11.4	22.8	34.2	1.3			T 34.228	3
**GLN**	**110**	12.8		10.1	10.7		2.7	0.7		0.7	2.0		0.7	47.7	2.7	5.4	1.3	2.7				P 47.651	1
**GLU**	**111**	31.8		8.1	45.3	0.7	2.7	1.4			0.7			0.7	3.4	1.4	1.4	1.4	1.4			E 45.270	3
**GLN**	**112**	0.7			0.7		0.7	1.4		0.7	5.4				82.4	4.7		2.0	1.4			Q 82.432	8
**GLU**	**113**	23.0			1.4		2.0	3.4	6.8	8.8	0.7				12.8	14.2	1.4	0.7	4.1	16.9	4.1	A 22.973	5
**GLU**	**114**	18.2		7.4	35.8		0.7	2.7		3.4			0.7	1.4	15.5	9.5	1.4	1.4	0.7	1.4		E 35.811	1
**HIS**	**115**	15.5		4.1	4.1	8.8		6.1		2.7	0.7		2.7	10.1	4.1	33.1	2.0	2.7	0.7		2.7	R 33.108	1
**LEU**	**116**	0.7				18.9	0.7	1.4	8.8		20.9	0.7			0.7	0.7	0.7	2.0	7.4	25.0	11.5	W 25.000	1

**TYR**	**117**	4.7		1.4	10.1	18.9	4.7	1.4	2.0		26.4				8.1	0.7	6.8	2.0		0.7	12.2	L 26.351	3
**THR**	**118**	10.8		1.4	15.5		6.8			8.8	0.7			1.4	2.7	35.1	5.4	9.5	2.0			R 35.135	2
**GLN**	**119**	10.0		4.0	22.0	1.3	11.3	2.7	1.3	2.7	11.3		0.7	2.7	11.3	16.0	0.7		0.7	1.3		E 22.000	1
**ILE**	**120**	8.0				0.7	0.7		9.3		2.7		4.0				12.7	51.3	8.0		2.7	T 51.333	7
**ALA**	**121**	36.7	0.7				2.7		8.7		9.3	0.7				0.7	0.7	9.3	30.7			A 36.667	5
**GLN**	**122**	22.0		2.7	23.3		1.3	1.3		4.0			2.0		8.7	20.0	10.0	4.7				E 23.333	1
**ASN**	**123**	2.0		1.3	18.0	0.7	18.7	10.7		8.7	2.0		22.7	0.7	9.3	4.0	1.3					N 22.667	2
**ASN**	**124**	2.0		5.3	1.3		9.3	2.0		7.3	0.7		32.7		9.3	25.3	4.0	0.7				N 32.667	3
**TRP**	**125**	0.7	0.7			0.7	0.7	1.3			22.0		0.7	0.7	1.3	8.0		0.7		54.7	8.0	W 54.667	4
**TRP**	**126**		3.3			71.3			0.7		9.3								2.0	8.7	4.7	F 71.333	7
**THR**	**127**					8.0		0.7	1.4						3.6			1.4	5.8	77.5	1.4	W 77.536	8
**GLY**	**128**	5.3	2.7				88.0										4.0					G 88.000	8
**ASN**	**129**	4.7		3.3			29.3						60.0				2.0	0.7				N 60.000	9
**ALA**	**130**	70.7					5.3		2.0								4.7	6.7	10.7			A 70.667	8
**SER**	**131**	2.7				4.7	4.0		2.0		33.3	0.7	0.7		10.0		8.7	2.7	30.7			L 33.333	6
**SER**	**132**	3.3		0.7				6.7					86.7		0.7		2.0					N 86.667	9
**GLU**	**133**	5.4			1.3		0.7							75.8		2.0	5.4	2.0	7.4			P 75.839	7
**ASN**	**134**												100.0									N 100.000	6*
**ASN**	**135**												100.0									N 100.000	6*
**SER**	**136**																100.0					S 100.000	6*
**HIS**	**137**							100.0														H 100.000	6*
**VAL**	**138**																		100.0			V 100.000	6*
**LEU**	**139**	3.3				2.7		0.7			40.0	2.0	1.3		8.0	38.7		0.7	2.7			L 40.000	7
**ASP**	**140**			84.7	3.3		4.7						1.3		0.7		4.7				0.7	D 84.667	9
**TRP**	**141**	9.3		2.7	1.3		5.3		6.7	12.0	2.7		3.3	18.7	2.7	12.0	5.3	16.0	0.7	1.3		P 18.667	2
**LYS**	**142**	4.7		18.0	1.3		10.0	2.0		2.7			0.7	2.0	1.3	46.7	6.7	3.3		0.7		R 46.667	6
**VAL**	**143**	5.3	0.7	0.7			1.3	0.7	1.3		43.3						6.7	30.0	10.0			L 43.333	6
**ARG**	**144**	2.7			7.3	2.0		0.7	6.7	10.7	4.0	0.7			6.0	17.3	2.7	20.7	18.7			T 20.667	1
**ALA**	**145**	30.7	1.3				4.7		14.0		33.3	1.3					2.0		12.7			L 33.333	6
**THR**	**146**	7.3		0.7	6.0		0.7	2.0	1.3	7.3	2.0		0.7		4.0	12.0	7.3	45.3	2.7		0.7	T 45.333	3
**PRO**	**147**	8.0		10.0	10.7		0.7	1.3		7.3	1.3		0.7	22.0	3.3	24.7	6.7	1.3	0.7	1.3		R 24.667	1
**THR**	**148**	3.3		26.7	10.0	5.3	1.3	2.7	1.3	0.7	3.3		1.3		4.0	12.7	3.3	14.7	8.7		0.7	D 26.667	1
**GLU**	**149**	12.5			6.3		25.0						3.1	37.5		6.3	9.4					P 37.500	1
**ASP**	**150**	8.0		28.7	8.7		42.7			2.0			2.0	4.7	0.7		2.0	0.7				G 42.667	1
**GLY**	**151**	1.3		28.0	10.0		41.3	1.3		2.0			7.3	0.7	1.3		1.3	4.7			0.7	G 41.333	1
**GLY**	**152**	1.3		6.0	5.3	0.7	48.7	7.3	0.7	1.3			4.7		2.0	1.3	7.3	3.3		8.7	1.3	G 48.667	1
**TYR**	**153**		0.7			20.7		4.0	6.7		12.7				0.7	5.3	0.7		2.0	11.3	35.3	Y 35.333	1
**VAL**	**154**	3.3			10.7	2.0			6.7		12.7				1.3	26.7	0.7	2.0	32.7		1.3	V 32.667	1
**LEU**	**155**					27.3			5.3		52.0								11.3		4.0	L 52.000	6
**ASN**	**156**	1.3		24.0	2.0			5.3		0.7			37.3		3.3	1.3	16.0	8.0	0.7			N 37.333	5
**GLY**	**157**						100.0															G 100.000	9
**THR**	**158**	4.0		4.0	2.7	6.7		2.7	4.7	13.3	4.0		0.7	1.3	4.7	26.7	4.7	5.3	13.3		1.3	R 26.667	1
**LYS**	**159**									83.3					3.3	13.3						K 83.333	9
**HIS**	**160**	0.7				5.3	10.0	10.7					2.7	6.0		2.7	46.0	15.3			0.7	S 46.000	6
**PHE**	**161**					86.7															13.3	F 86.667	9
**CYS**	**162**	20.7	40.7				2.0						6.0				28.0	1.3	1.3			C 40.667	7
**SER**	**163**	1.3															54.7	44.0				S 54.667	9
**GLY**	**164**	2.7					96.0						1.3									G 96.000	9
**ALA**	**165**	55.3					9.3										20.7	8.7	6.0			A 55.333	7
**LYS**	**166**	8.0			0.7		2.0	9.3	4.0	8.7	18.0	1.3	1.3	0.7	2.0	10.7	13.3	3.3	16.0		0.7	L 18.000	3
**GLY**	**167**	0.7	0.7	26.0		8.0	18.7		12.0		2.7						0.7	1.3	29.3			V 29.333	6
**SER**	**168**	31.3					5.3										63.3					S 63.333	8
**ASP**	**169**			78.0	8.0			4.0							8.7		1.3					D 78.000	8
**LEU**	**170**	2.7	0.7					0.7	6.0		13.3	17.3			4.7	20.0			15.3	10.0	9.3	R 20.000	4
**LEU**	**171**								19.3		53.3	7.3	0.7			1.3	0.7	14.7	2.7			L 53.333	7
**PHE**	**172**	2.0				0.7		0.7	12.0		13.3	0.7	7.3	10.0	2.0		4.7	20.0	25.3	0.7	0.7	V 25.333	6
**VAL**	**173**	16.0	2.7			2.0			10.7		18.7							2.0	48.0			V 48.000	6
**PHE**	**174**	3.3			14.7	1.3	3.3				8.7		5.3			4.7	49.3	8.7		0.7		S 49.333	8
**GLY**	**175**	64.0				0.7	23.3		0.7		2.0								4.0	5.3		A 64.000	7
**VAL**	**176**	6.7		2.0	4.0	2.0		6.0	6.0		6.0			2.0	6.0	8.7	4.7	7.3	24.7	8.7	5.3	V 24.667	1
**VAL**	**177**					10.0			20.0		30.0							10.0	30.0			LV 30.000	2*
**GLN**	**178**	20.0			20.0		20.0								40.0							Q 40.000	4*

**ASP**	**179**			66.7									33.3									D 66.667	6*
**ASP**	**180**			60.0			40.0															D 60.000	5*
**SER**	**181**	7.5		25.6	2.3	1.5	4.5	10.5	1.5	3.8	15.8	0.8	5.3	0.8	3.8	7.5	1.5	4.5	1.5		1.5	D 25.564	1
**PRO**	**182**	10.9		10.9	35.7		3.9		0.8	3.1			1.6	13.2	6.2	1.6	5.4	3.9	2.3		0.8	E 35.659	2
**GLN**	**183**	12.1		22.7	9.1	3.0	20.5	1.5	0.8	1.5	6.1		3.0	3.8	1.5	0.8	6.1	3.8	3.0		0.8	D 22.727	1
**GLN**	**184**	6.8	1.5	11.3	3.0		14.3	2.3	1.5		0.8		3.0	3.8	2.3	1.5	10.5	36.1	1.5			T 36.090	1
**GLY**	**185**	4.0		21.5	8.1		46.3			2.0			3.4		5.4	2.0	5.4	2.0				G 46.309	1
**ALA**	**186**	12.0		10.7	15.3		5.3			19.3			3.3		8.7	14.0	4.7	6.0	0.7			K 19.333	1
**ILE**	**187**	4.7		0.7		4.0	0.7	14.7	6.0	4.0	36.0	0.7		12.7		9.3	0.7	1.3	4.7			L 36.000	4
**ILE**	**188**	6.7						6.0	14.7		30.0					3.3	0.7	1.3	32.0	2.7	2.7	V 32.000	4
**ALA**	**189**	6.0				28.7	2.0	1.3	21.3		5.3	1.3	2.7		0.7		2.7	8.0	18.0		2.0	F 28.667	4
**ALA**	**190**	57.3				11.3	9.3				11.3						0.7	1.3	7.3		1.3	A 57.333	6
**ALA**	**191**	29.3	0.7			4.7		1.3	12.0		7.3				2.0		3.3	2.7	30.0		6.7	V 30.000	4
**ILE**	**192**	1.3							30.7		17.3							0.7	50.0			V 50.000	6
**PRO**	**193**	1.3		9.3	3.3					2.7				82.0		0.7		0.7				P 82.000	7
**THR**	**194**	13.3			0.7		6.0	0.7		2.7	0.7			4.0		8.7	13.3	49.3			0.7	T 49.333	5
**SER**	**195**	9.3		28.7	2.7	0.7	14.7	2.0	0.7	3.3	0.7		8.7	0.7	6.0	10.0	8.0	4.0				D 28.667	1
**ARG**	**196**	12.7		0.7				2.7	0.7	0.7	0.7				13.3	60.0	4.0	3.3	1.3			R 60.000	7
**ALA**	**197**	18.0		12.7	18.7		0.7	3.3		2.0				29.3	3.3	0.7	6.0	5.3				P 29.333	1
**GLY**	**198**	1.3					98.7															G 98.667	9
**VAL**	**199**					5.3			47.3		12.0								30.7		4.7	I 47.333	5
**THR**	**200**	5.3	0.7	0.7	2.0		3.3	2.0	3.3	1.3	2.7		1.3		10.0	8.0	8.7	35.3	14.7		0.7	T 35.333	1
**PRO**	**201**	6.0	0.7			22.0		2.7	16.7	0.7	7.3			4.0		1.3	1.3	0.7	32.7		4.0	V 32.667	5
**ASN**	**202**	7.3		2.0	5.3	0.7	10.7	17.3	4.0	0.7	18.7	0.7	14.0	0.7	6.0	2.7	1.3	1.3	5.3		1.3	L 18.667	1
**ASP**	**203**	2.7		50.7	1.3		17.3	10.7					7.3	2.7	4.0	1.3	1.3				0.7	D 50.667	3
**ASP**	**204**			95.3			1.3						3.3									D 95.333	9
**TRP**	**205**	2.0					1.3													96.7		W 96.667	9
**ALA**	**206**	2.0		80.7	0.7			2.7		0.7			12.0				1.3					D 80.667	7
**ALA**	**207**	15.3					14.0	0.7					55.3	7.3			4.7	2.0			0.7	N 55.333	8
**ILE**	**208**					18.0			39.3		16.7	20.7							5.3			I 39.333	6
**GLY**	**209**						100.0															G 100.000	9
**MET**	**210**										6.0	2.0			92.0							Q 92.000	9
**ARG**	**211**									2.0						96.7		1.3				R 96.667	9
**GLN**	**212**	0.7	0.7								37.3				50.7	2.7		7.3	0.7			Q 50.667	9
**THR**	**213**	3.3															12.0	84.7				T 84.667	9
**ASP**	**214**	22.0		54.0	16.0		6.0										0.7		1.3			D 54.000	8
**SER**	**215**				0.7		2.7										96.7					S 96.667	9
**GLY**	**216**			1.3			97.3						1.3									G 97.333	9
**SER**	**217**	1.3					10.0						4.0		0.7	1.3	52.7	30.0				S 52.667	7
**THR**	**218**	4.7				1.3			5.3		0.7						2.7	2.7	82.0		0.7	V 82.000	8
**ASP**	**219**	2.0		4.0	17.3	1.3	0.7	6.0	4.7	5.3	2.0		2.7		3.3	15.3	10.7	18.0	6.7			T 18.000	1
**PHE**	**220**					74.0			12.0		10.7								1.3		2.0	F 74.000	8
**HIS**	**221**	4.7		28.0	29.3			8.7		4.0			9.3		2.7	4.7	1.3	4.0	3.3			E 29.333	2
**ASN**	**222**	4.7		24.7	0.7		14.0	1.3		4.7			21.3		12.0	12.7	4.0					D 24.667	2
**VAL**	**223**	2.7				4.0			1.3		2.7							2.7	86.0		0.7	V 86.000	9
**LYS**	**224**	6.7			8.7	2.7		2.0	0.7	6.7	8.0			12.7	2.0	37.3	2.7	4.7	4.7		0.7	R 37.333	1
**VAL**	**225**	3.3							15.3		13.3							2.0	66.0			V 66.000	6
**GLU**	**226**	12.0		6.0	24.0	2.0	2.7	2.7		3.3	2.7			23.3	1.3	6.0	4.0	3.3			6.7	E 24.000	1
**PRO**	**227**	18.9		10.1	12.8	0.7	1.4	6.1		5.4	0.7		2.0	16.2	0.7	6.1	2.0	1.4	0.7	14.9		A 18.919	1
**ASP**	**228**	12.8		35.1	12.2	0.7	3.4	8.1		2.7	0.7		4.1		1.4	1.4	13.5	3.4	0.7			D 35.135	1
**GLU**	**229**	7.3		22.7	50.0			1.3			1.3		1.3		4.7	6.0	4.0			1.3		E 50.000	6
**VAL**	**230**	15.3	0.7			4.7			18.0		18.7	1.3				1.3			40.0			V 40.000	6
**LEU**	**231**	7.3				4.0			5.3		78.0	0.7							4.7			L 78.000	7
**GLY**	**232**	5.3		2.0	0.7	1.3	42.7	4.0	1.3	1.3	13.3		1.3	8.7	6.0	2.0	3.3	4.7	2.0			G 42.667	3
**ALA**	**233**	10.2	0.7	12.2	6.1	6.8	2.7	6.1			2.0	0.7	6.8	9.5	2.7	6.8	8.2	0.7	4.1	8.2	5.4	D 12.245	1
**PRO**	**234**	4.1		8.8	2.0	0.7	3.4	8.8	0.7	0.7	9.5		0.7	43.2	1.4	1.4	4.1	1.4	8.8		0.7	P 43.243	4
**ASN**	**235**	8.8		14.3	8.2		32.7	1.4			6.8		2.0	9.5	0.7	1.4	8.2	3.4	2.7			G 32.653	1
**ALA**	**236**	15.4		3.7	7.4	2.9	0.7	2.9	1.5	8.1	2.2		1.5	24.3	8.1	2.9	6.6	7.4	4.4			P 24.265	1
**PHE**	**237**	6.9		6.2	6.9	1.5	4.6		1.5	3.8	21.5		2.3	18.5	4.6	1.5	6.2	7.7	3.8	0.8	1.5	L 21.538	1
**VAL**	**238**	6.2		8.5	6.2	8.5	10.0		0.8	9.2	2.3		0.8	13.1	1.5	0.8	23.8	3.8	2.3	1.5	0.8	S 23.846	2
**LEU**	**239**	15.4		13.5	3.8		11.5			17.3	1.9		1.9	3.8	17.3	7.7		3.8	1.9			KQ 17.308	1
**ALA**	**240**	17.6		5.9	7.8				2.0					45.1			7.8	7.8	5.9			P 45.098	2

**PHE**	**241**			50.0		16.7			16.7		8.3					8.3						D 50.000	3*
**ILE**	**242**								15.4		76.9			7.7								L 76.923	6
**GLN**	**243**	5.4				3.6	7.1			3.6	16.1	1.8		16.1	5.4	5.4	12.5	19.6	3.6			T 19.643	1
**SER**	**244**	1.3		3.3	4.7	6.7	0.7	2.0	4.7	2.0	2.7		2.7	4.0		7.3	22.0	34.7	1.3			T 34.667	4
**GLU**	**245**	8.0		1.3	3.3	0.7	0.7	0.7	1.3		1.3			59.3	2.7	4.7	0.7	2.0	12.7		0.7	P 59.333	4
**ARG**	**246**	2.0				34.7	6.0	0.7	0.7	0.7	4.7				4.0	26.0	1.3		0.7	2.0	16.7	F 34.667	4
**GLY**	**247**	40.0		4.0	2.7		3.3	1.3	2.0	0.7			8.0	9.3	5.3	0.7	17.3	4.0	1.3			A 40.000	4
**SER**	**248**	4.0	10.7				4.0	0.7			1.3				9.3		24.7	45.3				T 45.333	6
**LEU**	**249**		0.7			8.0			3.3		83.3	0.7						0.7	3.3			L 83.333	8
**PHE**	**250**	4.0	0.7	1.3		2.0	0.7	6.7	3.3		8.7		12.7			46.7	1.3	2.0	10.0			R 46.667	6
**ALA**	**251**	10.7				0.7	10.0		0.7		8.0		3.3	20.7			14.0	22.0	10.0			T 22.000	5
**PRO**	**252**	6.0	7.3					2.0	2.7		34.0	2.7		34.7	7.3			1.3	1.3		0.7	P 34.667	5
**ILE**	**253**	6.0				6.0	3.3	4.0	20.7		26.7	0.7			1.3		0.7	12.0	18.0		0.7	L 26.667	3
**ALA**	**254**	39.3				4.0	5.3	1.3	22.0		0.7	0.7			4.7		14.7	2.7	0.7	4.0		A 39.333	7
**GLN**	**255**							1.3							94.7	3.3					0.7	Q 94.667	9
**LEU**	**256**	4.7							3.3		81.3	0.7			0.7		8.0	0.7	0.7			L 81.333	7
**ILE**	**257**	6.0					0.7	2.0	26.7		10.7	1.3	2.7				0.7	3.3	46.0			V 46.000	6
**PHE**	**258**					40.7		6.7	2.0	2.0	40.0	3.3			1.3	0.7	3.3					F 40.667	8
**ALA**	**259**	31.3					4.0		2.7		4.0						9.3	20.7	28.0			A 31.333	5
**ASN**	**260**	8.7	0.7		2.7	0.7		14.0			0.7		59.3		8.0	0.7	2.7	0.7			1.3	N 59.333	7
**VAL**	**261**					27.3			27.3		30.0	3.3							12.0			L 30.000	6
**TYR**	**262**	0.7	2.7	8.0		20.0					4.0					1.3					63.3	Y 63.333	7
**LEU**	**263**	10.0							10.0		58.7				0.7			1.3	19.3			L 58.667	6
**GLY**	**264**	3.3					96.7															G 96.667	9
**ILE**	**265**								64.0		7.3	2.0	3.3				6.0	12.0	5.3			I 64.000	8
**ALA**	**266**	92.0					5.3										1.3	0.7	0.7			A 92.000	9
**HIS**	**267**	3.3		0.7	53.3	0.7	2.7	2.7	0.7	0.7	6.0	0.7			18.7	6.0	1.3	0.7	0.7	1.3		E 53.333	5
**GLY**	**268**	2.7		0.7			96.0						0.7									G 96.000	8
**ALA**	**269**	92.0					1.3		1.3		0.7						1.3	0.7	2.7			A 92.000	9
**LEU**	**270**			0.7		23.3		0.7	0.7		72.0				1.3	0.7			0.7			L 72.000	7
**ASP**	**271**	28.0		16.0	24.7		0.7	0.7	0.7	4.0			2.7		11.3	4.0	2.7	4.0	0.7			A 28.000	1
**ALA**	**272**	22.0		5.3	44.0	4.7	0.7		0.7				0.7		7.3	1.3	0.7	8.7	3.3		0.7	E 44.000	4
**ALA**	**273**	91.3				0.7	6.7											0.7	0.7			A 91.333	9
**ARG**	**274**	14.7	1.3		0.7	0.7		1.3	2.0	12.7	13.3		1.3		0.7	36.0	7.3	1.3	6.0	0.7		R 36.000	4
**GLU**	**275**	10.7		22.0	27.3		4.0	4.0		6.0			2.7	1.3	6.0	11.3	2.0	2.7				E 27.333	1
**TYR**	**276**	1.3				12.0		2.7			2.0	1.3				1.3		2.0		4.7	72.7	Y 72.667	7
**THR**	**277**	2.0				1.3	0.7		2.7		4.0						2.7	62.0	24.7			T 62.000	7
**ARG**	**278**	2.7					0.7	2.7		6.0	17.3	0.7	1.3		4.7	50.0	2.0	6.0	6.0			R 50.000	4
**THR**	**279**	12.3		6.2	15.8		2.1	2.1		8.2	2.7		2.1		3.4	5.5	4.1	35.6				T 35.616	2
**GLN**	**280**	4.7		1.3	13.3		2.7	9.3		12.0			5.3		14.7	9.3	8.7	14.7	1.3	1.3	1.3	QT 14.667	1
**ALA**	**281**	20.1		0.7			11.4	2.0		0.7	0.7			1.3	2.7	3.4	23.5	31.5	2.0			T 31.544	5
**ARG**	**282**	0.7					1.4			8.1			0.7		2.7	79.7	6.1		0.7			R 79.730	8
**PRO**	**283**	23.0						0.7			0.7			73.0			2.0		0.7			P 72.973	6
**TRP**	**284**					2.0											0.7			97.3		W 97.279	9
**THR**	**285**	3.4			4.1	20.4	0.7	4.8	16.3	0.7	12.9	1.4		17.7	2.0	1.4	2.7	2.0	8.8		0.7	F 20.408	2
**PRO**	**286**	16.8		2.7	10.1	5.4	2.0	9.4	0.7	3.4	8.1	1.3	2.0	4.0		3.4	4.7	15.4	2.0		8.7	A 16.779	3
**ALA**	**287**	14.7		1.3	0.7	0.7	13.3							0.7			66.7	2.0				S 66.667	7
**GLY**	**288**	2.7		6.7	0.7		67.3	2.0	0.7	0.7	3.3		4.0	2.0	1.3	0.7	3.3	2.0		2.0	0.7	G 67.333	2
**ILE**	**289**	16.0		9.3	2.0	1.3		2.0	2.0		6.7			3.3		0.7	2.7	0.7	52.7		0.7	V 52.667	5
**GLN**	**290**	16.0		16.7	42.7		0.7	0.7			0.7		2.7	0.7	7.3	1.3	4.7	4.0	1.3		0.7	E 42.667	1
**GLN**	**291**	5.3	0.7	4.7	3.3		2.0	4.7	0.7	6.7	0.7		0.7		8.7	34.7	16.7	10.7				R 34.667	1
**ALA**	**292**	69.3					3.3	5.3	1.3		0.7		0.7	4.7	0.7		5.3	2.7	4.7		1.3	A 69.333	7
**THR**	**293**	16.7		0.7	3.3		4.7	2.0	4.7	0.7	0.7		2.0	3.3	5.3	4.0	12.7	23.3	16.0			T 23.333	1
**GLU**	**294**	2.7		46.0	28.7		0.7	1.3		0.7	1.3		3.3		12.7	2.0			0.7			D 46.000	4
**ASP**	**295**	0.7		84.0	14.7								0.7									D 84.000	9
**PRO**	**296**					7.3	0.7	0.7			0.7			86.7		0.7			0.7	2.7		P 86.667	8
**TYR**	**297**					6.0	0.7	1.3			13.3				2.0					4.7	72.0	Y 72.000	7
**THR**	**298**	2.0						3.3	39.3		13.3	0.7	1.3		1.3			10.7	28.0			I 39.333	4
**ILE**	**299**	0.7						0.7	10.7	2.7	58.7				19.3	3.3			4.0			L 58.667	6
**ARG**	**300**	10.7		4.7	18.0		3.3	8.7		4.0	4.0		0.7		18.7	15.3	4.7	3.3	2.7		1.3	Q 18.667	4
**SER**	**301**	5.3		0.7	0.7		4.7	24.0	1.3	3.3	6.7		2.0		4.0	32.7	0.7	12.0	2.0			R 32.667	4
**TYR**	**302**	2.7				25.3		0.7	2.0		3.3	0.7						0.7	8.0		56.7	Y 56.667	5

**GLY**	**303**	4.7					95.3															G 95.333	9
**GLU**	**304**			19.3	54.0		2.0			0.7			10.0		4.7	7.3	0.7	1.3				E 54.000	5
**PHE**	**305**					33.3			1.3		52.7	11.3							0.7		0.7	L 52.667	7
**THR**	**306**	9.3		1.3	0.7	5.3	0.7	5.3	0.7	0.7	0.7	0.7			6.7	10.0	5.3	9.3	6.0	33.3	4.0	W 33.333	3
**ILE**	**307**	22.7					0.7		9.3		14.0						15.3	4.0	34.0			V 34.000	5
**ALA**	**308**	14.7		3.3	7.3		9.3	8.0		14.0	5.3		0.7	0.7	17.3	13.3	4.0	2.0				Q 17.333	2
**LEU**	**309**	6.7					0.7		8.0		58.0							9.3	16.7		0.7	L 58.000	5
**GLN**	**310**	6.7		1.3	14.0	0.7		0.7		7.3	3.3		1.3		14.0	34.0	2.7	1.3	4.7	8.0		R 34.000	6
**GLY**	**311**	66.7	0.7				12.7							4.0			16.0					A 66.667	7
**ALA**	**312**	66.0		2.0			3.3				1.3						10.0	4.7	12.7			A 66.000	7
**ASP**	**313**	13.3		7.3	26.7		2.7		8.0	1.3	0.7		0.7		3.3	19.3	5.3	8.0	3.3			E 26.667	3
**ALA**	**314**	62.7	0.7		2.7		0.7				23.3						2.0	0.7	7.3			A 62.667	7
**ALA**	**315**	2.0						1.3	0.7		94.7	0.7			0.7							L 94.667	9
**ALA**	**316**	60.0					0.7		1.3		11.3		0.7				2.7	12.0	10.7		0.7	A 60.000	6
**ARG**	**317**	1.3		64.0	14.0		0.7	0.7		1.3			5.3		2.7	7.3	0.7	1.3	0.7			D 64.000	5
**GLU**	**318**	10.0		2.0	10.7			4.0	2.0	8.0	5.3		2.0		10.7	34.0	6.0	2.0	2.7		0.7	R 34.000	1
**ALA**	**319**	86.0					1.3										2.0	2.0	8.7			A 86.000	8
**ALA**	**320**	51.3		0.7	0.7		26.0		1.3		4.7	0.7	3.3		1.3	1.3	1.3	3.3	4.0			A 51.333	4
**HIS**	**321**	19.3		10.0	16.0		3.3	6.0	1.3	1.3	6.7		1.3		7.3	18.7	4.7	0.7	2.7		0.7	A 19.333	1
**LEU**	**322**	37.3			14.0		0.7	0.7	1.3	4.7	9.3	1.3			10.7	6.7	6.0	4.0	3.3			A 37.333	2
**LEU**	**323**	2.7				12.7	2.0		11.3		48.7	2.0			0.7				18.7	0.7	0.7	L 48.667	4
**GLN**	**324**	1.3	0.7	38.7	9.3		0.7	0.7			2.0	0.7	0.7		32.0	2.7	6.0	2.7	1.3	0.7		D 38.667	4
**THR**	**325**	35.3	0.7	6.7	10.0		4.7	4.7	0.7	4.7	0.7		4.0		12.7	4.7	3.3	5.3	0.7	1.3		A 35.333	1
**VAL**	**326**	64.7	0.7		0.7		5.3	1.3	6.0		10.0						0.7	4.0	6.7			A 64.667	7
**TRP**	**327**	2.9	0.7	0.7		7.2		8.6	2.9		21.6		0.7			0.7	1.4	1.4	1.4	40.3	9.4	W 40.288	4
**ASP**	**328**	35.3		15.8	12.2	1.4	4.3	2.2	0.7		4.3		3.6	0.7	4.3	7.2	7.2		0.7			A 35.252	1
**LYS**	**329**	6.5	0.7	5.0	2.2	1.4		0.7	3.6	22.3	10.1		1.4	2.2	10.8	27.3	2.9	1.4	1.4			R 27.338	1
**GLY**	**330**	3.4		2.0	7.4		62.2	0.7		2.7				2.0	2.7	16.2	0.7					G 62.162	4
**ASP**	**331**	17.6		16.2	14.9	1.4	2.7	4.7		1.4	2.0		2.0	12.8	2.0	6.1	6.1	4.1	1.4	4.7		A 17.568	1
**ALA**	**332**	34.7		20.7	6.7		9.3	1.3		3.3	2.0		7.3		3.3	0.7	9.3	1.3				A 34.667	1
**LEU**	**333**					0.7			4.0		73.3			2.0				0.7	19.3			L 73.333	7
**THR**	**334**	1.3		3.3	1.3		3.3			0.7		0.7	1.3				12.0	76.0				T 76.000	7
**PRO**	**335**	49.3		9.3	18.7	4.0	0.7	1.3						7.3	0.7	1.3	2.0	0.7	1.3	2.0	1.3	A 49.333	4
**GLU**	**336**	16.0		22.7	29.3		2.0	1.3					1.3	0.7	9.3	12.0	2.0	2.0	1.3			E 29.333	1
**ASP**	**337**	9.3		8.7	43.3	0.7	2.0		0.7	1.3	0.7	0.7	1.3		18.7	2.0	7.3	3.3				E 43.333	4
**ARG**	**338**	6.0	1.3			0.7		0.7								84.7	0.7		1.3	3.3	1.3	R 84.667	9
**GLY**	**339**	27.3					71.3	0.7					0.7									G 71.333	8
**GLU**	**340**	12.0		6.0	60.0				0.7	3.3	1.3				2.7	9.3	2.0	0.7	2.0			E 60.000	4
**LEU**	**341**	28.0	4.0			1.3		2.0	7.3		20.7				0.7			5.3	30.7			V 30.667	4
**MET**	**342**	74.0			3.3		0.7					1.3					18.7	1.3	0.7			A 74.000	8
**VAL**	**343**	4.0	0.7		7.3				34.0		14.0	2.0						2.7	35.3			V 35.333	4
**LYS**	**344**	48.7	0.7	4.0	8.7			1.3		1.3	10.0					8.0	5.3	6.0	0.7	5.3		A 48.667	5
**VAL**	**345**	6.7					1.3		41.3		1.3						0.7	2.0	46.7			V 46.667	6
**SER**	**346**	68.0		3.3		2.7	2.7						0.7	0.7			14.0				8.0	A 68.000	7
**GLY**	**347**	38.7			20.7		1.3			0.7		2.0			6.0		10.7	19.3	0.7			A 38.667	7
**VAL**	**348**	84.7					0.7		0.7		3.3						1.3	2.0	7.3			A 84.667	8
**LYS**	**349**	0.7			3.3					86.7			6.0			3.3						K 86.667	9
**ALA**	**350**	14.7	0.7						10.0		2.7				2.7		1.3	3.3	60.7		4.0	V 60.667	6
**LEU**	**351**	22.0				2.7		6.0	2.0		16.7	1.3	2.0		6.7	7.3	2.0	2.7	25.3	1.3	2.0	V 25.333	4
**ALA**	**352**	68.0	0.7						2.0								11.3	15.3	2.7			A 68.000	7
**THR**	**353**	12.7					6.7	12.7	2.0				2.0		3.3		13.3	43.3	4.0			T 43.333	7
**ASN**	**354**	0.7		17.3	12.7		0.7	2.7		13.3			6.7		4.7	40.0	1.3					R 40.000	4
**ALA**	**355**	31.3	0.7	2.7					6.0		3.3	0.7	2.0		1.3		0.7	21.3	30.0			A 31.333	5
**ALA**	**356**	46.0					32.0										18.7	1.3	2.0			A 46.000	6
**LEU**	**357**								2.7		80.0		2.7		3.3			1.3	10.0			L 80.000	8
**ASN**	**358**	3.3		33.3	40.7	2.0		2.7		1.3	1.3	0.7	2.7		2.0	8.7		0.7			0.7	E 40.667	4
**ILE**	**359**	10.7							27.3		7.3		1.3				2.7	3.3	47.3			V 47.333	4
**SER**	**360**	15.3	6.7				12.7						0.7	0.7			15.3	48.0	0.7			T 48.000	5
**SER**	**361**	9.3	0.7	0.7	8.0		1.3	3.3					10.0		6.7		49.3	10.0	0.7			S 49.333	5
**GLY**	**362**	12.0		2.0	8.0		11.3			11.3			3.3		9.3	40.7	0.7	1.3				R 40.667	5
**VAL**	**363**	3.3							30.0		32.0	10.0							24.7			L 32.000	5
**PHE**	**364**					88.7					6.0										5.3	F 88.667	8

GLU	365			16.0	78.0										6.0							E 78.000	8
VAL	366	10.7				2.0	0.7		6.7		22.7	0.7			1.3	0.7		0.7	53.3	0.7		V 53.333	6
ILE	367	10.0	5.3				1.3		2.7		4.7	20.0			4.0		4.0	42.7	5.3			T 42.667	6
GLY	368						100.0															G 100.000	9
ALA	369	80.0					1.3							0.7			6.7	11.3				A 80.000	8
ARG	370	1.3		0.7			0.7	0.7		2.7	0.7				0.7	88.0	4.0	0.7				R 88.000	8
GLY	371	57.3					4.7										36.0	1.3			0.7	A 57.333	7
THR	372	16.7									1.3						3.3	78.0	0.7			T 78.000	8
HIS	373	36.0		0.7			0.7	14.0		2.0	8.0		1.3	0.7	1.3	0.7	16.7	16.0	2.0			A 36.000	5
PRO	374	37.3		4.7	1.3		4.0	1.3	0.7	1.3	2.0		12.7	1.3	1.3	7.3	17.3	6.7	0.7			A 37.333	4
ARG	375	19.3		2.7	2.0		6.0	3.3		22.0			0.7	8.0	5.3	12.0	18.0	0.7				K 22.000	2
TYR	376	2.7			1.3	6.7		7.3			36.0		2.7		0.7	2.0		1.3	14.0		25.3	L 36.000	3
GLY	377	4.7	0.7				67.3	0.7					10.7			16.0						G 67.333	7
PHE	378					37.3		0.7			55.3	1.3							1.3		4.0	L 55.333	5
ASP	379			93.3				6.0					0.7									D 93.333	9
ARG	380							0.7	4.7		3.3					90.7			0.7			R 90.667	9
PHE	381					60.7		20.0													19.3	F 60.667	6
TRP	382					0.7														99.3		W 99.333	9
ARG	383															100.0						R 100.000	9
ASN	384			16.0									84.0									N 84.000	9
VAL	385	32.7							8.0		24.0				0.7				34.7			V 34.667	7
ARG	386															100.0						R 100.000	9
THR	387								2.0		0.7							82.7	14.7			T 82.667	9
HIS	388					4.0		80.0			6.0				9.3						0.7	H 80.000	9
SER	389																15.3	84.7				T 84.667	9
LEU	390										94.0							2.0	4.0			L 94.000	9
HIS	391		0.7					96.7								0.7					2.0	H 96.667	9
ASP	392			90.7	2.7								6.0						0.7			D 90.667	9
PRO	393													98.7	0.7		0.7					P 98.667	9
VAL	394	10.1							12.1		20.1			0.7				1.3	55.7			V 55.705	6
SER	395	23.5		42.3	7.4	4.7		0.7						2.7	2.0	8.1	4.7	0.7	3.4			D 42.282	7
TYR	396							5.4						1.3					0.7	8.1	84.6	Y 84.564	8
LYS	397									85.2						14.8						K 85.235	9
ILE	398	16.1			0.7	0.7		0.7	14.8	4.7	28.9		6.0		2.7	6.7	1.3	2.7	10.1		4.0	L 28.859	3
ALA	399	2.7						5.4	0.7	8.7	2.7		0.7		15.4	61.1	0.7	0.7	0.7		0.7	R 61.074	6
ASP	400	4.0		25.5	54.4		0.7	6.7			0.7		3.4		0.7			3.4	0.7			E 54.362	8
VAL	401								19.5		37.6								43.0			V 42.953	7
GLY	402						100.0															G 100.000	9
LYS	403	7.4		21.5	13.4					2.7	0.7		9.4		5.4	34.2	1.3	2.7	1.3			R 34.228	4
HIS	404	0.7				15.4		18.1												40.3	25.5	W 40.268	4
THR	405	41.6			1.3	10.7			0.7		10.1	0.7			4.7	1.3		5.4	20.1		3.4	A 41.611	5
LEU	406					0.7			1.3		95.3							0.7	2.0			L 95.302	9
ASN	407	0.7		3.4	1.3			1.3	1.3	2.0	12.8		43.0			3.4	7.4	23.5				N 42.953	5
GLY	408	1.3		14.1	3.4		60.4	3.4					4.0		5.4	6.0	1.3				0.7	G 60.403	1
GLN	409	9.4		2.7	25.5			1.3	3.4	5.4	0.7		1.3	0.7	12.8	12.1	2.7	16.1	6.0			E 25.503	1
TYR	410	6.1			0.7	7.5		8.8	10.2	1.4	21.8			4.8		9.5	0.7		13.6	1.4	13.6	L 21.769	1
PRO	411													100.0								P 100.000	9
ILE	412	10.8		5.9	36.3			2.9	5.9	8.8	2.0		2.0	2.0	1.0	2.0	2.9	13.7	3.9			E 36.275	1
PRO	413	2.2				1.1		1.1						94.6			1.1					P 94.565	8
GLY	414	2.6		1.3			11.8						2.6			3.9	39.5	38.2				S 39.474	6
PHE	415					70.2	2.1		4.3		4.3								2.1	17.0		F 70.213	6
THR	416																	6.5			93.5	Y 93.548	8
SER	417																100.0					S 100.000	6*

References

1. Guan, L. J.; Lee, W. C.; Wang, S.; Ohshiro, T.; Izumi, Y.; Ohtsuka, J.; Tanokura, M., Crystal structures of apo-DszC and FMN-bound DszC from Rhodococcus erythropolis D-1. *Febs J* **2015**, *282*, 3126-35.

2. Hino, T.; Hamamoto, H.; Suzuki, H.; Yagi, H.; Ohshiro, T.; Nagano, S., Crystal structures of TdsC, a dibenzothiophene monooxygenase from the thermophile Paenibacillus sp A11-2, reveal potential for expanding its substrate selectivity. *J Biol Chem* **2017**, *292*, 15804-15813.

3. Sondergaard, C. R.; Olsson, M. H. M.; Rostkowski, M.; Jensen, J. H., Improved Treatment of Ligands and Coupling Effects in Empirical Calculation and Rationalization of pK_a Values. *J Chem Theor Comp* **2011**, *7*, 2284-2295.

4. Zgarbova, M.; Otyepka, M.; Sponer, J.; Mladek, A.; Banas, P.; Cheatham, T. E.; Jurecka, P., Refinement of the Cornell et al. Nucleic Acids Force Field Based on Reference Quantum Chemical Calculations of Glycosidic Torsion Profiles. *J Chem Theor Comp* **2011**, *7*, 2886-2902.

5. Wang, J. M.; Wolf, R. M.; Caldwell, J. W.; Kollman, P. A.; Case, D. A., Development and testing of a general amber force field. *J Comput Chem* **2004**, *25*, 1157-1174.

6. Frisch, M. J.; Trucks, G. W.; Schlegel, H. B.; Scuseria, G. E.; Robb, M. A.; Cheeseman, J. R.; Scalmani, G.; Barone, V.; Mennucci, B.; Petersson, G. A.; Nakatsuji, H.; Caricato, M.; Li, X.; Hratchian, H. P.; Izmaylov, A. F.; Bloino, J.; Zheng, G.; Sonnenberg, J. L.; Hada, M.; Ehara, M.; Toyota, K.; Fukuda, R.; Hasegawa, J.; Ishida, M.; Nakajima, T.; Honda, Y.; Kitao, O.; Nakai, H.; Vreven, T.; Montgomery Jr., J. A.; Peralta, J. E.; Ogliaro, F.; Bearpark, M. J.; Heyd, J.; Brothers, E. N.; Kudin, K. N.; Staroverov, V. N.; Kobayashi, R.; Normand, J.; Raghavachari, K.; Rendell, A. P.; Burant, J. C.; Iyengar, S. S.; Tomasi, J.; Cossi, M.; Rega, N.; Millam, N. J.; Klene, M.; Knox, J. E.; Cross, J. B.; Bakken, V.; Adamo, C.; Jaramillo, J.; Gomperts, R.; Stratmann, R. E.; Yazyev, O.; Austin, A. J.; Cammi, R.; Pomelli, C.; Ochterski, J. W.; Martin, R. L.; Morokuma, K.; Zakrzewski, V. G.; Voth, G. A.; Salvador, P.; Dannenberg, J. J.; Dapprich, S.; Daniels, A. D.; Farkas, Ö.; Foresman, J. B.; Ortiz, J. V.; Cioslowski, J.; Fox, D. J. *Gaussian 09*, Gaussian, Inc.: Wallingford, CT, USA: Gaussian, Inc., 2009.

7. Jorgensen, W. L.; Chandrasekhar, J.; Madura, J. D.; Impey, R. W.; Klein, M. L., Comparison of Simple Potential Functions for Simulating Liquid Water. *J Chem Phys* **1983**, *79*, 926-935.

8. Becke, A. D., Density-Functional Exchange-Energy Approximation with Correct Asymptotic-Behavior. *Phys Rev A* **1988**, *38*, 3098-3100.

9. Becke, A. D., Density-functional thermochemistry. III. The role of exact exchange. *J Chem Phys* **1993**, *98*, 5648-5652.

10. Becke, A. D., Density-functional thermochemistry. IV. A new dynamical correlation functional and implications for exact-exchange mixing. *J Chem Phys* **1996**, *104*, 1040-1046.

11. Harihara.Pc; Pople, J. A., Influence of Polarization Functions on Molecular-Orbital Hydrogenation Energies. *Theoret Chim Acta* **1973**, *28*, 213-222.

12. Hariharan, P. C.; Pople, J. A., Accuracy of Ah Equilibrium Geometries by Single Determinant Molecular-Orbital Theory. *Mol Phys* **1974**, *27*, 209-214.

13. Hehre, W. J.; Ditchfield, R.; Pople, J. A., Self-Consistent Molecular-Orbital Methods .12. Further Extensions of Gaussian-Type Basis Sets for Use in Molecular-Orbital Studies of Organic-Molecules. *J Chem Phys* **1972**, *56*, 2257-+.

14. Lee, C.; Yang, W.; Parr, R. G., Development of the Colle-Salvetti correlation-energy formula into a functional of the electron density. *Phys Rev B* **1988**, *37*, 785-789.

15. Miehlich, B.; Savin, A.; Stoll, H.; Preuss, H., Results Obtained with the Correlation-Energy Density Functionals of Becke and Lee, Yang and Parr. *Chem Phys Lett* **1989**, *157*, 200-206.

16. Vosko, S. H.; Wilk, L.; Nusair, M., Accurate Spin-Dependent Electron Liquid Correlation Energies for Local Spin-Density Calculations - a Critical Analysis. *Can J Phys* **1980**, *58*, 1200-1211.

17. Calixto, A. R.; Brás, N. F.; Fernandes, P. A.; Ramos, M. J., Reaction Mechanism of Human Renin Studied by Quantum Mechanics/Molecular Mechanics (QM/MM) Calculations. *ACS Catal* **2014**, *4*, 3869-3876.

18. Neves, R. P. P.; Fernandes, P. A.; Ramos, M. J., Unveiling the Catalytic Mechanism of $NADP^+$-Dependent Isocitrate Dehydrogenase with QM/MM Calculations. *ACS Catal* **2016**, *6*, 357-368.

19. Ribeiro, A. J. M.; Yang, L.; Ramos, M. J.; Fernandes, P. A.; Liang, Z.-X.; Hirao, H., Insight into Enzymatic Nitrile Reduction: QM/MM Study of the Catalytic Mechanism of QueF Nitrile Reductase. *ACS Catal* **2015**, *5*, 3740-3751.

20. Gesto, D. S.; Cerqueira, N. M. F. S. A.; Fernandes, P. A.; Ramos, M. J., Unraveling the Enigmatic Mechanism of L-Asparaginase II with QM/QM Calculations. *J Am Chem Soc* **2013**, *135*, 7146-7158.

21. Seeger, R.; Pople, J. A., Self-Consistent Molecular-Orbital Methods .18. Constraints and Stability in Hartree-Fock Theory. *J Chem Phys* **1977**, *66*, 3045-3050.

22. Bauernschmitt, R.; Ahlrichs, R., Stability analysis for solutions of the closed shell Kohn-Sham equation. *J Chem Phys* **1996**, *104*, 9047-9052.

23. Schlegel, H. B.; McDouall, J. J. Do You Have SCF Stability and Convergence Problems? In *Computational Advances in Organic Chemistry*, Ögretir, C.; Csizmadia, I. G., Eds.; Kluwer Academic: The Netherlands, 1991; Vol. *330*, pp 167-185.

24. Maseras, F.; Morokuma, K., Imomm - a New Integrated Ab-Initio Plus Molecular Mechanics Geometry Optimization Scheme of Equilibrium Structures and Transition-States. *J Comput Chem* **1995**, *16*, 1170-1179.

25. Singh, U. C.; Kollman, P. A., A Combined *Ab initio* Quantum-Mechanical and Molecular Mechanical Method for Carrying out Simulations on Complex Molecular-Systems - Applications to the CH_3Cl + Cl^- Exchange-Reaction and Gas-Phase Protonation of Polyethers. *J Comput Chem* **1986**, *7*, 718-730.

26. Field, M. J.; Bash, P. A.; Karplus, M., A Combined Quantum-Mechanical and Molecular Mechanical Potential for Molecular-Dynamics Simulations. *J Comput Chem* **1990**, *11*, 700-733.

27. Das, D.; Eurenius, K. P.; Billings, E. M.; Sherwood, P.; Chatfield, D. C.; Hodoscek, M.; Brooks, B. R., Optimization of quantum mechanical molecular mechanical partitioning schemes: Gaussian delocalization of molecular mechanical charges and the double link atom method. *J Chem Phys* **2002**, *117*, 10534-10547.
