# Supplementary material for: Engineering DszC Mutants from Transition State Macrodipole Considerations and Evolutionary Sequence Analysis
Source: J Chem Inf Model. 2022 Dec 19;63(1):20–6. doi: 10.1021/acs.jcim.2c01337 (PMC9832474; doi:10.1021/acs.jcim.2c01337)
Supplement: Supplementary file 3 — ci2c01337_si_003.zip [file ci2c01337_si_003.zip › Multicolored_MSA.html]

ConSurf Color-Coded MSA

# ConSurf Color-Coded MSA

|  |  |  |  |  |  |  |  |  |  |  |  |  |  |  |  |  |  |  |  |  |  |  |  |  |  |  |  |  |  |  |  |  |  |  |  |  |  |  |  |  |  |  |  |  |  |  |  |  |  |  |
| --- | --- | --- | --- | --- | --- | --- | --- | --- | --- | --- | --- | --- | --- | --- | --- | --- | --- | --- | --- | --- | --- | --- | --- | --- | --- | --- | --- | --- | --- | --- | --- | --- | --- | --- | --- | --- | --- | --- | --- | --- | --- | --- | --- | --- | --- | --- | --- | --- | --- | --- |
| **001 Input\_pdb\_SEQRES\_A** | M | T | L | S | P | E | K | Q | H | - | - | V | R | P | R | D | A | A | D | N | D | - | P | V | - | - | - | A | V | A | R | G | L | A | E | K | W | R | A | T | A | V | E | R | D | R | A | G | G | S |
| 002 UniRef90\_Q1W1G3\_1\_416 | M | T | L | S | V | E | K | Q | H | - | - | V | R | P | G | D | - | A | D | N | D | - | P | V | - | - | - | A | V | A | R | G | L | A | E | K | W | R | A | T | A | V | E | R | D | R | A | G | G | S |
| 003 UniRef90\_T0BM21\_7\_392 | - | - | - | - | - | - | - | - | - | - | - | - | - | - | - | - | - | V | Q | D | D | - | I | W | - | - | - | A | S | V | Q | R | L | A | E | A | F | A | V | D | A | V | E | R | E | Q | A | G | G | N |
| 004 UniRef90\_UPI0002AC58FE\_11\_391 | - | - | - | - | - | - | - | - | - | - | - | - | - | - | - | - | - | - | - | - | D | - | Y | L | - | - | - | T | L | A | T | A | L | A | E | E | F | A | I | T | A | V | E | R | D | A | K | G | G | T |
| 005 UniRef90\_A0A3D5CW51\_20\_401 | - | - | - | - | - | - | - | - | - | - | - | - | - | - | - | - | - | - | - | - | - | - | A | L | - | - | - | A | V | A | K | Q | L | A | E | S | F | S | L | T | A | V | E | R | D | H | Q | G | G | T |
| 006 UniRef90\_A0A2V4SPP5\_16\_400 | - | - | - | - | - | - | - | - | - | - | - | - | - | - | - | T | A | A | R | S | G | - | P | L | - | - | - | A | I | A | A | D | L | A | E | R | L | A | A | T | A | V | E | R | D | R | A | G | G | H |
| 007 UniRef90\_E0UIV5\_8\_393 | - | - | - | - | - | - | - | - | - | - | - | - | - | - | - | - | - | T | T | N | D | - | H | L | - | - | - | N | L | A | A | S | L | S | Q | E | F | A | A | T | A | I | E | R | D | E | R | G | G | T |
| 008 UniRef90\_A0A1Z4S897\_9\_391 | - | - | - | - | - | - | - | - | - | - | - | - | - | - | - | - | - | - | - | T | D | - | F | L | - | - | - | A | I | A | T | T | L | A | E | E | F | A | K | T | A | V | L | R | D | A | K | G | G | T |
| 009 UniRef90\_UPI00045E9273\_21\_396 | - | - | - | - | - | - | - | - | - | - | - | - | - | - | - | - | - | - | - | - | - | - | - | - | - | - | - | - | - | A | E | Q | L | A | A | E | F | A | A | T | A | V | E | R | D | R | R | G | G | T |
| 010 UniRef90\_A0A353Y4M9\_17\_384 | - | - | - | - | - | - | - | - | - | - | - | - | - | - | - | - | - | - | - | - | - | - | - | - | - | - | - | - | - | - | - | - | - | - | - | S | L | A | A | T | A | V | E | R | D | R | H | G | G | H |
| 011 UniRef90\_F3KR10\_17\_384 | - | - | - | - | - | - | - | - | - | - | - | - | - | - | - | - | - | - | - | - | - | - | - | - | - | - | - | - | - | - | - | - | - | - | - | S | L | A | A | T | A | V | E | R | D | R | R | G | G | H |
| 012 UniRef90\_A0A2D8NW56\_20\_404 | - | - | - | - | - | - | - | - | - | - | - | - | - | - | - | - | - | D | G | A | D | H | W | L | - | - | - | T | V | A | D | S | V | A | E | R | L | A | E | T | A | V | E | R | D | R | R | G | G | H |
| 013 UniRef90\_A0A2W7M9P1\_16\_386 | - | - | - | - | - | - | - | - | - | - | - | - | - | - | - | - | - | - | - | - | - | - | - | - | - | - | - | - | - | - | - | - | L | I | A | E | I | S | K | T | A | S | E | R | D | K | L | G | G | T |
| 014 UniRef90\_A0A398AYR9\_12\_386 | - | - | - | - | - | - | - | - | - | - | - | - | - | - | - | - | - | - | - | - | - | - | - | - | - | - | - | - | I | V | K | S | L | T | E | E | F | A | K | T | A | S | E | R | D | K | R | G | G | T |
| 015 UniRef90\_A0A0T6UXN9\_11\_394 | - | - | - | - | - | - | - | - | - | - | - | - | - | - | - | - | - | - | - | - | A | - | P | L | - | - | - | Q | I | A | R | E | L | A | A | Q | F | A | E | T | A | V | E | R | D | A | R | G | G | T |
| 016 UniRef90\_A0A252E884\_11\_390 | - | - | - | - | - | - | - | - | - | - | - | - | - | - | - | - | - | - | - | Q | D | - | W | I | - | - | - | A | I | A | S | S | L | A | A | E | L | T | A | T | A | V | E | R | D | Q | K | A | G | L |
| 017 UniRef90\_A0A1Z4BZ71\_24\_403 | - | - | - | - | - | - | - | - | - | - | - | - | - | - | - | - | - | - | - | - | - | - | - | - | - | - | - | A | Q | A | E | Q | L | S | A | Y | F | Y | E | T | A | A | E | R | D | K | Q | G | G | T |
| 018 UniRef90\_A0A1Z4IGC5\_10\_387 | - | - | - | - | - | - | - | - | - | - | - | - | - | - | - | - | - | - | - | - | D | - | Y | I | - | - | - | K | L | A | A | S | L | V | P | V | F | A | Q | T | A | V | E | R | D | K | Q | G | G | T |
| 019 UniRef90\_A0A1W9JD13\_18\_401 | - | - | - | - | - | - | - | - | - | - | - | - | - | - | - | - | - | - | - | K | T | - | P | L | - | - | - | E | I | A | H | D | L | S | A | E | F | A | K | T | V | A | E | R | D | Y | L | G | G | T |
| 020 UniRef90\_A0A0B6S5D2\_8\_393 | - | - | - | - | - | - | - | - | - | - | - | - | - | T | A | A | A | A | D | P | D | A | P | - | - | - | - | R | T | F | G | E | L | I | V | A | L | R | A | S | A | A | E | R | D | R | R | G | G | H |
| 021 UniRef90\_A0A1Y3C786\_14\_396 | - | - | - | - | - | - | - | - | - | - | - | - | - | - | - | - | - | - | - | S | D | - | V | I | - | - | - | P | S | A | Q | F | L | A | Q | Q | F | A | Q | T | A | I | H | R | D | R | T | G | G | T |
| 022 UniRef90\_A0A318KD41\_20\_397 | - | - | - | - | - | - | - | - | - | - | - | - | - | - | - | - | - | - | - | - | - | - | - | - | - | - | - | - | - | A | A | R | L | A | A | R | F | A | E | T | A | V | E | R | D | Q | R | G | G | T |
| 023 UniRef90\_A0A352JDP6\_15\_389 | - | - | - | - | - | - | - | - | - | - | - | - | - | - | - | - | - | - | - | - | - | - | - | - | - | - | - | A | S | V | K | K | L | A | D | E | F | T | T | T | A | I | E | R | D | R | V | G | G | T |
| 024 UniRef90\_A0A329B538\_13\_401 | - | - | - | - | - | - | - | - | - | - | - | - | - | - | A | R | P | R | H | D | D | - | I | F | - | - | - | A | I | A | D | E | L | A | Q | R | F | A | A | T | A | V | A | R | D | Q | A | G | G | T |
| 025 UniRef90\_A0A2N8QAF3\_13\_394 | - | - | - | - | - | - | - | - | - | - | - | - | - | - | - | L | P | T | D | A | A | - | P | V | V | - | - | R | D | L | A | G | L | L | K | A | L | R | A | S | A | A | Q | R | D | R | D | G | G | H |
| 026 UniRef90\_A0A2N7XWT8\_11\_394 | - | - | - | - | - | - | - | - | - | - | - | - | - | - | - | - | - | - | - | - | T | - | P | L | - | - | - | E | I | A | R | D | L | A | T | E | F | A | R | T | A | V | D | R | D | A | S | G | G | T |
| 027 UniRef90\_A0A2A4HLB2\_33\_412 | - | - | - | - | - | - | - | - | - | - | - | - | - | - | - | - | - | - | - | - | - | - | - | L | - | - | - | A | I | T | D | Q | L | A | A | R | L | A | E | S | A | V | E | R | D | R | L | G | G | H |
| 028 UniRef90\_A0A4R3HWL3\_41\_416 | - | - | - | - | - | - | - | - | - | - | - | - | - | - | - | - | - | - | - | - | - | - | - | - | - | - | - | - | - | - | - | - | - | A | S | V | L | A | L | T | A | P | E | R | D | R | Q | G | G | T |
| 029 UniRef90\_A0A1M7NXP6\_14\_394 | - | - | - | - | - | - | - | - | - | - | - | - | - | - | - | - | - | - | - | Y | N | - | P | L | - | - | - | S | V | A | A | E | L | A | Q | R | L | A | A | T | A | N | T | R | D | Q | A | G | G | H |
| 030 UniRef90\_A0A1B4ESK2\_22\_397 | - | - | - | - | - | - | - | - | - | - | - | - | - | - | - | - | - | - | - | - | - | - | - | - | - | - | - | D | A | L | A | R | A | I | D | A | L | R | A | T | A | V | A | R | D | R | A | G | G | H |
| 031 UniRef90\_A0A1H6NKV2\_11\_394 | - | - | - | - | - | - | - | - | - | - | - | - | - | - | - | - | - | - | - | - | D | - | P | L | - | - | - | Q | T | A | R | L | L | A | A | E | F | A | E | T | A | V | E | R | D | E | R | G | G | T |
| 032 UniRef90\_A0A0D0KUF8\_11\_394 | - | - | - | - | - | - | - | - | - | - | - | - | - | - | - | - | - | - | - | - | S | - | P | L | - | - | - | Q | T | A | R | K | L | A | A | D | F | A | E | T | A | V | E | R | D | E | A | G | G | T |
| 033 UniRef90\_A0A0F3K7B4\_20\_401 | - | - | - | - | - | - | - | - | - | - | - | - | - | - | - | - | - | - | - | - | - | - | A | L | - | - | - | L | L | A | Q | Q | L | A | A | Q | F | A | A | T | A | A | E | R | D | H | A | G | G | T |
| 034 UniRef90\_A0A381IMG6\_13\_394 | - | - | - | - | - | - | - | - | - | - | - | - | - | - | - | - | E | A | D | R | E | - | P | - | - | - | - | R | T | L | A | E | L | I | A | A | L | R | A | S | A | P | E | R | D | R | A | G | G | H |
| 035 UniRef90\_A0A2S9K1G1\_26\_400 | - | - | - | - | - | - | - | - | - | - | - | - | - | - | - | - | - | - | - | - | - | - | - | - | - | - | - | - | - | - | E | A | L | A | Q | A | L | A | A | N | A | V | E | R | D | R | A | G | G | H |
| 036 UniRef90\_A0A1W6L7W5\_17\_392 | - | - | - | - | - | - | - | - | - | - | - | - | - | - | - | - | - | - | - | - | - | - | - | - | - | - | - | - | M | A | D | R | L | A | L | R | L | A | T | T | A | V | A | R | D | Q | A | G | G | H |
| 037 UniRef90\_A0A255HJE3\_14\_398 | - | - | - | - | - | - | - | - | - | - | - | - | - | - | - | P | A | A | P | P | D | - | A | I | - | - | - | A | V | A | T | F | L | A | A | R | L | A | A | T | A | N | A | R | D | Q | A | G | G | H |
| 038 UniRef90\_A0A0R3AD45\_11\_394 | - | - | - | - | - | - | - | - | - | - | - | - | - | - | - | - | - | - | - | - | S | - | P | L | - | - | - | Q | T | A | R | Q | L | A | A | E | F | A | L | T | A | V | E | R | D | E | R | G | G | T |
| 039 UniRef90\_A0A1W6ZB60\_16\_389 | - | - | - | - | - | - | - | - | - | - | - | - | - | - | - | - | - | - | - | - | - | - | - | - | - | - | - | - | - | - | E | A | L | A | A | Q | L | A | A | T | A | V | E | R | D | R | A | G | G | H |
| 040 UniRef90\_A0A2X1DPB7\_14\_397 | - | - | - | - | - | - | - | - | - | - | - | - | - | - | - | - | - | - | - | T | D | A | P | A | V | D | R | A | A | L | A | R | A | I | D | A | L | R | V | S | A | A | E | R | D | R | A | G | G | H |
| 041 UniRef90\_A0A0S9M2D1\_4\_380 | - | - | - | - | - | - | - | - | - | - | - | - | - | - | - | - | - | - | - | - | - | - | - | - | - | - | - | - | - | A | Q | A | L | A | T | R | F | A | A | T | A | A | E | R | D | R | R | G | G | T |
| 042 UniRef90\_A0A238ZKW1\_13\_401 | - | - | - | - | - | - | - | - | - | - | - | - | - | - | P | A | D | T | L | K | S | - | S | L | - | - | - | E | I | A | R | E | L | A | A | E | F | R | L | T | A | V | E | R | D | Q | A | G | G | T |
| 043 UniRef90\_A0A0D1P8V6\_10\_394 | - | - | - | - | - | - | - | - | - | - | - | - | - | - | - | - | - | - | - | S | T | - | P | L | - | - | - | Q | I | A | R | D | L | A | Q | T | F | S | R | S | V | V | E | R | D | R | K | G | G | T |
| 044 UniRef90\_A0A261SP68\_11\_387 | - | - | - | - | - | - | - | - | - | - | - | - | - | - | - | - | - | - | - | - | - | - | - | - | - | - | - | A | V | V | D | D | L | V | K | K | L | A | A | S | A | V | E | R | D | R | Q | G | G | H |
| 045 UniRef90\_A0A2N6MRB2\_11\_390 | - | - | - | - | - | - | - | - | - | - | - | - | - | - | - | - | - | - | - | K | D | - | W | I | - | - | - | A | I | A | S | S | L | S | A | E | L | T | T | T | A | V | E | R | D | T | K | A | G | L |
| 046 UniRef90\_A0A212BVA1\_31\_405 | - | - | - | - | - | - | - | - | - | - | - | - | - | - | - | - | - | - | - | - | - | - | - | - | - | - | - | - | K | V | E | R | L | A | R | K | L | A | E | N | A | V | A | R | D | R | Q | G | G | H |
| 047 UniRef90\_A0A1A9KE82\_11\_394 | - | - | - | - | - | - | - | - | - | - | - | - | - | - | - | - | - | - | - | - | N | - | P | L | - | - | - | Q | I | A | R | Q | L | A | A | G | F | A | E | N | A | A | E | R | D | E | R | G | G | T |
| 048 UniRef90\_A0A4Q5PVB9\_26\_400 | - | - | - | - | - | - | - | - | - | - | - | - | - | - | - | - | - | - | - | - | - | - | - | - | - | - | - | - | - | - | E | K | V | A | E | E | L | A | R | T | A | A | T | R | D | K | T | G | G | T |
| 049 UniRef90\_A0A178GPM9\_9\_392 | - | - | - | - | - | - | - | - | - | - | - | - | - | - | - | - | - | - | N | Q | S | - | P | V | - | - | - | D | I | A | Q | K | L | A | E | N | F | A | L | T | A | A | E | R | D | K | Q | G | G | N |
| 050 UniRef90\_A0A1P9YC32\_3\_390 | - | - | - | - | - | - | - | - | - | - | - | - | - | - | - | - | - | R | T | Q | D | A | P | W | - | - | N | E | P | L | E | A | V | L | R | E | W | A | R | T | A | A | A | R | D | K | T | G | G | T |
| 051 UniRef90\_UPI000A1773A9\_40\_416 | - | - | - | - | - | - | - | - | - | - | - | - | - | - | - | - | - | - | - | - | - | - | - | - | - | - | - | - | - | - | D | E | L | V | A | R | F | A | E | T | A | P | S | R | D | L | L | G | G | T |
| 052 UniRef90\_A0A158L201\_25\_395 | - | - | - | - | - | - | - | - | - | - | - | - | - | - | - | - | - | - | - | - | - | - | - | - | - | - | - | - | - | - | - | - | - | V | A | A | L | R | E | T | A | E | A | R | D | R | E | G | G | H |
| 053 UniRef90\_A0A4Q4GT15\_13\_391 | - | - | - | - | - | - | - | - | - | - | - | - | - | - | - | - | - | - | - | - | - | - | - | - | - | - | - | E | I | A | Q | Q | L | A | H | T | F | A | K | T | A | A | E | R | D | K | A | G | G | N |
| 054 UniRef90\_A0A1C0YC62\_17\_381 | - | - | - | - | - | - | - | - | - | - | - | - | - | - | - | - | - | - | - | - | - | - | - | - | - | - | - | - | - | - | - | - | - | - | T | T | F | A | K | T | A | A | K | R | D | K | Q | G | G | T |
| 055 UniRef90\_A0A1H7FE04\_32\_405 | - | - | - | - | - | - | - | - | - | - | - | - | - | - | - | - | - | - | - | - | - | - | - | - | - | - | - | - | - | - | - | - | L | A | Q | R | L | D | E | T | A | V | A | R | D | Q | A | G | G | H |
| 056 UniRef90\_A0A401MWC4\_5\_385 | - | - | - | - | - | - | - | - | - | - | - | - | - | - | - | - | - | - | - | - | D | - | W | I | - | - | - | A | T | A | R | A | V | A | A | D | L | A | Q | D | A | A | A | R | D | K | A | N | K | P |
| 057 UniRef90\_A0A1G6HFW7\_16\_395 | - | - | - | - | - | - | - | - | - | - | - | - | - | - | - | - | - | - | - | - | - | - | - | - | - | - | - | P | V | V | D | S | V | A | E | E | L | E | R | T | A | V | A | R | D | R | A | G | G | T |
| 058 UniRef90\_A0A0P9B999\_30\_403 | - | - | - | - | - | - | - | - | - | - | - | - | - | - | - | - | - | - | - | - | - | - | - | - | - | - | - | - | - | - | - | A | L | A | N | T | L | A | A | S | A | A | E | R | D | R | A | G | G | T |
| 059 UniRef90\_A0A140K6F0\_14\_392 | - | - | - | - | - | - | - | - | - | - | - | - | - | - | - | - | - | - | - | - | - | - | - | Q | - | - | - | V | V | A | E | E | L | S | E | A | I | A | A | S | A | L | E | R | D | Q | K | A | A | I |
| 060 UniRef90\_A0A1P8EKI7\_5\_392 | - | - | - | - | - | - | - | - | - | - | - | - | - | - | Y | F | A | Q | T | F | D | - | P | L | - | - | - | V | Q | A | Q | S | L | A | T | A | F | E | K | T | A | A | V | R | D | K | Q | G | G | T |
| 061 UniRef90\_A0A2U3MYZ3\_7\_392 | - | - | - | - | - | - | - | - | - | - | - | - | - | - | - | - | S | S | E | F | N | - | A | I | - | - | - | Q | T | A | E | Q | L | A | E | Q | F | A | Q | T | A | A | E | R | D | K | K | G | G | N |
| 062 UniRef90\_A0A1H2EPX5\_7\_397 | - | - | - | - | - | - | - | - | - | - | - | - | - | - | - | L | S | T | A | E | T | - | - | - | - | R | S | A | A | V | G | A | L | A | E | T | F | A | R | T | A | A | E | R | D | R | L | G | G | T |
| 063 UniRef90\_UPI00041EF43F\_25\_400 | - | - | - | - | - | - | - | - | - | - | - | - | - | - | - | - | - | - | - | - | - | - | - | - | - | - | - | - | - | T | E | A | L | A | R | A | L | E | E | S | A | V | A | R | D | Q | R | G | G | H |
| 064 UniRef90\_D0IW93\_17\_384 | - | - | - | - | - | - | - | - | - | - | - | - | - | - | - | - | - | - | - | - | - | - | - | - | - | - | - | - | - | - | - | - | - | - | - | - | - | - | K | T | A | V | A | R | D | R | Q | G | G | H |
| 065 UniRef90\_A0A395D1I6\_11\_388 | - | - | - | - | - | - | - | - | - | - | - | - | - | - | - | - | - | - | - | - | - | - | P | N | - | - | - | A | A | V | E | A | L | V | A | D | F | S | A | T | A | A | V | R | D | A | A | G | G | T |
| 066 UniRef90\_A0A1H0TK45\_27\_400 | - | - | - | - | - | - | - | - | - | - | - | - | - | - | - | - | - | - | - | - | - | - | - | - | - | - | - | - | - | - | - | - | - | T | Q | A | L | A | A | T | A | V | A | R | D | Q | R | G | G | T |
| 067 UniRef90\_A0A1H8MYU5\_23\_403 | - | - | - | - | - | - | - | - | - | - | - | - | - | - | - | - | - | - | - | N | P | - | Y | L | - | - | - | A | K | A | I | E | L | R | E | I | F | A | V | D | A | V | E | R | D | Q | Q | G | G | R |
| 068 UniRef90\_A0A1B1M254\_5\_403 | - | - | - | T | P | T | H | W | Q | - | - | T | G | P | A | P | R | T | A | Q | D | - | W | I | - | - | - | A | R | A | A | E | V | A | A | V | L | A | T | D | A | A | E | R | D | H | A | G | A | T |
| 069 UniRef90\_A0A484THM3\_28\_401 | - | - | - | - | - | - | - | - | - | - | - | - | - | - | - | - | - | - | - | - | - | - | - | - | - | - | - | A | K | V | E | A | L | A | V | A | L | A | S | T | A | A | Q | R | D | R | E | G | G | S |
| 070 UniRef90\_A0A0Q8Q8X6\_8\_377 | - | - | - | - | - | - | - | - | - | - | - | - | - | - | - | - | - | - | - | - | - | - | - | - | - | - | - | - | - | - | - | - | L | L | A | T | L | R | A | T | A | V | E | R | D | R | T | G | G | H |
| 071 UniRef90\_A0A0Q5QDL2\_32\_410 | - | - | - | - | - | - | - | - | - | - | - | - | - | - | - | - | - | - | - | - | - | - | - | - | - | - | - | - | - | A | H | E | V | A | S | I | L | A | A | D | A | V | E | R | D | R | A | G | A | T |
| 072 UniRef90\_A0A1A5XM62\_22\_406 | - | - | - | - | - | - | - | - | - | - | - | - | - | - | - | - | - | - | - | - | - | - | - | - | - | - | - | - | - | - | - | T | L | A | A | T | L | R | E | S | A | A | R | R | D | L | A | G | G | H |
| 073 UniRef90\_A0A239EI90\_21\_397 | - | - | - | - | - | - | - | - | - | - | - | - | - | - | - | - | - | - | - | - | - | - | - | - | - | - | - | - | - | - | - | E | V | A | Q | L | L | A | V | D | A | V | A | R | D | K | G | D | A | P |
| 074 UniRef90\_S5SWW2\_23\_403 | - | - | - | - | - | - | - | - | - | - | - | - | - | - | - | - | - | - | - | N | P | - | Y | L | - | - | - | A | K | A | V | E | L | R | E | I | F | A | R | D | A | A | E | R | D | R | L | G | G | R |
| 075 UniRef90\_A0A3A5JJY9\_31\_413 | - | - | - | - | - | - | - | - | - | - | - | - | - | - | - | - | - | - | - | A | D | - | W | L | - | - | - | A | I | A | D | D | V | A | A | R | L | D | I | T | A | A | E | R | D | A | V | G | G | H |
| 076 UniRef90\_A0A315ZU85\_7\_384 | - | T | - | - | - | - | - | - | - | - | - | - | - | - | - | - | - | - | - | - | - | - | - | - | - | - | - | L | A | A | R | D | A | A | A | K | L | A | V | D | A | L | E | R | D | R | A | N | A | E |
| 077 UniRef90\_A0A3R9U899\_5\_381 | - | - | - | T | P | P | D | F | T | - | - | T | R | P | T | P | R | T | A | A | D | - | W | I | - | - | - | A | R | A | G | E | V | A | A | V | L | A | T | D | A | V | E | R | D | R | A | G | A | T |
| 078 UniRef90\_UPI000DD53D91\_7\_389 | - | - | - | - | - | - | - | - | - | - | - | - | - | - | - | - | - | A | D | D | T | - | L | L | - | - | - | A | K | A | I | E | L | R | Q | A | F | H | R | D | A | V | E | R | D | K | A | G | G | R |
| 079 UniRef90\_UPI000DE4BE09\_13\_396 | - | - | - | - | - | - | - | - | - | - | - | - | - | - | - | N | S | S | R | E | D | - | L | F | - | - | - | S | R | A | E | A | I | S | A | D | L | A | T | R | A | A | E | L | D | R | Q | G | R | P |
| 080 UniRef90\_A0A2T0R7P7\_10\_381 | - | - | - | - | - | - | - | - | - | - | - | - | - | - | - | - | - | - | - | - | - | - | - | - | - | - | - | - | A | A | R | D | V | A | N | H | L | A | L | D | A | L | E | R | D | R | A | N | A | E |
| 081 UniRef90\_A0A4R1HYS3\_27\_404 | - | - | - | - | - | - | - | - | - | - | - | - | - | - | - | - | - | - | - | - | - | - | - | - | - | - | - | - | - | - | R | E | V | R | R | A | L | E | T | D | A | A | A | R | D | R | A | G | E | T |
| 082 UniRef90\_UPI000D1537B0\_7\_385 | - | - | - | - | - | - | - | - | - | - | - | - | - | - | - | - | - | - | - | - | - | - | - | L | - | - | - | A | R | A | R | D | L | A | D | T | L | A | P | S | A | A | A | R | D | R | Q | G | G | R |
| 083 UniRef90\_A0A1G6ZNM7\_17\_409 | - | - | - | - | - | - | - | - | - | - | - | - | - | - | - | - | A | T | S | A | A | - | W | V | - | - | - | A | R | A | R | E | V | A | A | R | L | A | V | D | A | V | E | R | D | R | E | G | A | T |
| 084 UniRef90\_UPI0003765980\_18\_389 | - | - | - | - | - | - | - | - | - | - | - | - | - | - | - | - | - | - | - | - | - | - | - | - | - | - | - | - | - | - | - | - | L | R | A | V | F | H | L | D | A | V | D | R | D | K | Q | G | G | R |
| 085 UniRef90\_A0A071ICK8\_25\_398 | - | - | - | - | - | - | - | - | - | - | - | - | - | - | - | - | - | - | - | - | - | - | - | - | - | - | - | - | - | - | - | E | I | S | A | D | F | A | K | R | A | A | E | L | D | R | E | G | L | P |
| 086 UniRef90\_A0A2L0WNE5\_33\_396 | - | - | - | - | - | - | - | - | - | - | - | - | - | - | - | - | - | - | - | - | - | - | - | - | - | - | - | - | - | - | - | - | - | - | - | - | - | - | - | R | A | A | A | L | D | R | E | G | R | P |
| 087 UniRef90\_A0A1Q8CFJ2\_9\_380 | - | - | - | - | - | - | - | - | - | - | - | - | - | - | - | - | - | - | - | - | - | - | - | - | - | - | - | - | - | A | D | Q | L | A | R | T | L | R | A | D | A | P | H | R | E | Q | A | A | A | E |
| 088 UniRef90\_UPI000835F605\_21\_394 | - | - | - | - | - | - | - | - | - | - | - | - | - | - | - | - | - | - | - | - | - | - | - | - | - | - | - | - | - | - | - | S | A | A | A | E | L | A | A | T | A | L | E | R | D | R | A | N | Q | D |
| 089 UniRef90\_UPI000374AB86\_31\_398 | - | - | - | - | - | - | - | - | - | - | - | - | - | - | - | - | - | - | - | - | - | - | - | - | - | - | - | - | - | - | - | - | - | - | - | - | - | A | L | R | A | A | T | L | D | R | E | G | R | P |
| 090 UniRef90\_A0A267RVL6\_22\_399 | - | - | - | - | - | - | - | - | - | - | - | - | - | - | - | - | - | - | - | - | - | - | - | - | - | - | - | - | V | A | Q | E | V | A | D | R | L | Y | A | T | L | L | E | R | D | R | A | N | K | H |
| 091 UniRef90\_M3VA40\_23\_395 | - | - | - | - | - | - | - | - | - | - | - | - | - | - | - | - | - | - | - | - | - | - | - | - | - | - | - | - | - | - | D | L | V | A | A | E | L | R | A | T | A | A | E | R | D | R | A | N | A | A |
| 092 UniRef90\_A0A395GID9\_32\_425 | - | - | - | - | - | - | - | - | - | - | - | - | - | - | - | - | - | - | - | - | - | - | - | L | - | - | - | A | R | A | R | E | V | A | Q | V | L | G | Q | D | A | A | Q | R | D | Q | E | N | K | S |
| 093 UniRef90\_UPI0008268C2F\_35\_406 | - | - | - | - | - | - | - | - | - | - | - | - | - | - | - | - | - | - | - | - | - | - | - | - | - | - | - | - | - | - | - | - | V | A | A | Q | L | R | A | T | A | A | E | R | D | R | A | N | K | A |
| 094 UniRef90\_A0A0S1XV73\_27\_397 | - | - | - | - | - | - | - | - | - | - | - | - | - | - | - | - | - | - | - | - | - | - | - | - | - | - | - | - | - | A | V | A | L | R | P | L | L | Q | A | D | A | V | Q | R | D | K | A | G | G | R |
| 095 UniRef90\_A0A0F5N116\_19\_398 | - | - | - | - | - | - | - | - | - | - | - | - | - | - | - | - | - | - | - | - | - | - | - | L | - | - | - | A | T | A | D | R | V | A | D | E | L | R | Q | T | A | A | A | R | D | K | A | N | A | A |
| 096 UniRef90\_A0A1N6Z7J9\_40\_406 | - | - | - | - | - | - | - | - | - | - | - | - | - | - | - | - | - | - | - | - | - | - | - | - | - | - | - | - | - | - | - | - | - | - | - | - | - | - | - | - | - | A | K | L | D | A | E | G | R | P |
| 097 UniRef90\_A0A2V4UWQ2\_31\_411 | - | - | - | - | - | - | - | - | - | - | - | - | - | - | - | - | - | - | - | - | - | - | - | - | - | - | - | A | R | A | T | A | I | A | A | D | L | R | A | R | G | A | A | L | D | R | D | G | R | P |
| 098 UniRef90\_A0A4P8KLT1\_5\_401 | - | T | L | T | P | A | R | T | H | F | Q | G | T | A | D | A | A | E | L | A | H | - | W | D | - | - | - | A | V | A | A | S | V | A | E | R | L | A | L | D | A | L | A | R | D | R | A | N | A | Q |
| 099 UniRef90\_UPI0003068598\_3\_292 | - | - | - | - | - | - | - | - | - | - | - | - | - | - | - | - | - | - | - | - | - | - | - | - | - | - | - | - | - | - | - | - | - | - | - | - | - | - | - | - | - | - | - | - | - | - | - | - | - | - |
| 100 UniRef90\_UPI000DD585C3\_27\_403 | - | - | - | - | - | - | - | - | - | - | - | - | - | - | - | - | - | - | - | - | - | - | - | - | - | - | - | - | Q | A | S | E | V | S | A | K | F | A | R | R | G | P | E | L | D | R | L | G | L | P |
| 101 UniRef90\_A0A1E3SMN8\_25\_404 | - | - | - | - | - | - | - | - | - | - | - | - | - | - | - | - | - | - | - | - | - | - | - | L | - | - | - | A | T | A | D | R | V | A | D | E | L | R | R | T | A | A | E | R | D | K | A | N | A | T |
| 102 UniRef90\_UPI00045EBD18\_12\_396 | - | - | - | - | - | - | - | - | S | - | - | L | A | P | A | A | P | A | R | P | D | - | W | I | - | - | - | A | R | A | Q | S | V | A | D | A | L | T | P | D | A | A | E | R | D | R | I | G | A | S |
| 103 UniRef90\_A0A1Y1ZNX6\_42\_434 | - | - | - | - | - | - | - | - | - | - | - | - | - | - | - | - | - | - | - | - | - | - | - | - | - | - | - | - | - | A | Q | E | V | A | D | V | L | A | V | D | A | V | L | R | D | K | E | N | K | S |
| 104 UniRef90\_A0A0D2FPI6\_31\_423 | - | - | - | - | - | - | - | - | - | - | - | - | - | - | - | - | - | - | - | - | - | - | W | I | - | - | - | A | R | A | Q | D | V | G | Q | I | L | A | A | D | A | P | K | R | D | I | E | N | K | S |
| 105 UniRef90\_A0A2S0KGS2\_30\_403 | - | - | - | - | - | - | - | - | - | - | - | - | - | - | - | - | - | - | - | - | - | - | - | - | - | - | - | - | - | - | D | L | V | A | A | E | L | R | A | S | A | A | E | R | D | R | A | N | A | A |
| 106 UniRef90\_A0A1A7MGQ0\_54\_442 | - | - | - | - | - | - | - | - | - | - | - | - | - | - | - | - | - | - | - | - | - | - | - | - | - | - | - | - | - | A | R | D | V | A | S | I | L | A | T | N | A | A | A | R | D | I | D | N | K | S |
| 107 UniRef90\_UPI000413001D\_18\_392 | - | - | - | - | - | - | - | - | - | - | - | - | - | - | - | - | - | - | - | - | - | - | - | - | - | - | - | A | A | A | G | Q | V | A | E | A | L | R | A | D | A | A | A | R | D | H | A | G | L | A |
| 108 UniRef90\_G7H5P9\_11\_400 | - | - | - | - | - | - | - | - | - | - | N | V | A | D | Q | Q | G | R | F | D | D | - | A | L | - | - | - | R | R | A | D | L | V | A | A | E | L | R | S | T | A | A | E | R | D | R | A | N | A | D |
| 109 UniRef90\_A0A010YHQ5\_11\_404 | - | T | - | - | - | - | - | - | - | - | - | D | R | P | D | P | A | E | H | A | R | - | W | V | - | - | - | A | V | A | D | R | V | A | A | Q | L | S | S | D | V | V | A | R | D | R | S | G | A | A |
| 110 UniRef90\_B0RBU7\_24\_404 | - | - | - | - | - | - | - | - | - | - | - | - | - | - | - | - | - | E | L | A | H | - | W | R | - | - | - | G | I | A | E | H | V | A | A | T | L | A | E | D | A | L | A | R | D | R | A | G | L | D |
| 111 UniRef90\_A0A167RW81\_42\_429 | - | - | - | - | - | - | - | - | - | - | - | - | - | - | - | - | - | - | - | - | - | - | - | - | - | - | - | - | - | - | - | - | - | - | - | V | L | A | R | D | A | G | V | R | E | R | E | N | K | S |
| 112 UniRef90\_A0A1X0J8A0\_26\_405 | - | - | - | - | - | - | - | - | - | - | - | - | - | - | - | - | - | - | - | - | - | - | - | - | - | - | - | - | T | A | Q | R | V | A | D | Q | L | A | A | T | A | L | D | R | D | R | A | N | Q | N |
| 113 UniRef90\_A0A2J6Q859\_43\_430 | - | - | - | - | - | - | - | - | - | - | - | - | - | - | - | - | - | - | - | - | - | - | - | - | - | - | - | - | - | - | - | E | V | A | E | I | L | A | V | D | V | V | Q | R | D | R | E | N | K | S |
| 114 UniRef90\_UPI0003828F02\_7\_389 | - | - | - | - | - | - | - | - | - | - | - | - | - | - | - | - | - | - | - | - | - | - | - | V | - | - | - | A | V | A | R | E | V | A | A | R | L | V | A | D | A | V | A | R | E | R | E | T | A | E |
| 115 UniRef90\_A0A1B2HDJ6\_6\_361 | - | - | - | - | - | - | - | - | - | - | - | - | - | - | - | - | - | - | - | - | - | - | - | - | - | - | - | D | V | A | R | D | V | A | A | T | L | R | S | D | A | A | D | R | D | R | A | N | Q | P |
| 116 UniRef90\_A0A2N3N0U1\_47\_427 | - | - | - | - | - | - | - | - | - | - | - | - | - | - | - | - | - | - | - | - | - | - | - | - | - | - | - | - | - | - | - | - | - | - | - | - | - | - | V | D | A | A | Q | R | E | K | E | N | K | T |
| 117 UniRef90\_S5Y4X6\_32\_408 | - | - | - | - | - | - | - | - | - | - | - | - | - | - | - | - | - | - | - | - | - | - | - | - | - | - | - | - | - | A | R | A | I | A | A | E | L | A | A | T | I | S | A | R | D | R | A | N | L | D |
| 118 UniRef90\_A0A3R2WQN2\_17\_411 | - | - | - | - | - | - | - | - | - | - | - | - | K | P | R | P | A | D | A | P | Q | - | W | L | - | - | - | A | R | A | A | E | V | A | A | I | L | A | V | D | Q | V | A | R | D | R | Q | Q | A | V |
| 119 UniRef90\_A0A3N1X255\_20\_392 | - | - | - | - | - | - | - | - | - | - | - | - | - | - | - | - | - | - | - | - | - | - | - | - | - | - | - | - | - | A | A | A | L | R | E | V | L | H | A | D | A | V | A | R | D | E | A | G | G | Q |
| 120 UniRef90\_A0A1S1LA29\_28\_404 | - | - | - | - | - | - | - | - | - | - | - | - | - | - | - | - | - | - | - | - | - | - | - | - | - | - | - | - | - | - | E | R | V | A | G | Q | L | A | A | T | A | L | T | R | D | R | A | N | Q | N |
| 121 UniRef90\_A0A081GNL4\_19\_392 | - | - | - | - | - | - | - | - | - | - | - | - | - | - | - | - | - | - | - | - | - | - | - | - | - | - | - | - | - | - | - | D | L | C | D | H | L | T | D | D | A | A | A | R | D | R | L | G | G | V |
| 122 UniRef90\_A0A1I6U758\_27\_419 | - | - | - | - | - | - | - | - | - | - | - | - | - | P | T | P | S | T | P | E | E | - | W | I | - | - | - | A | R | A | R | A | V | A | R | V | L | A | A | D | A | V | Q | R | D | R | A | G | T | P |
| 123 UniRef90\_A0A0F5VZP9\_7\_388 | - | - | - | - | - | - | - | - | - | - | - | - | - | - | - | - | A | D | D | T | E | - | A | L | - | - | - | A | V | A | A | A | L | A | E | E | F | R | A | G | A | T | A | R | D | A | E | R | R | L |
| 124 UniRef90\_A0A1H4GIM8\_20\_411 | - | - | - | - | - | - | - | - | - | - | - | - | - | - | R | P | H | D | S | A | G | - | W | I | - | - | - | R | R | A | Q | E | V | A | D | I | L | S | V | D | A | L | E | R | D | R | A | N | A | T |
| 125 UniRef90\_A0A0M8TTT1\_8\_388 | - | - | - | - | - | - | - | - | - | - | - | - | - | - | - | - | - | D | D | V | E | - | A | L | - | - | - | T | V | A | R | S | L | A | D | E | F | R | A | G | A | S | Q | R | D | A | E | R | R | L |
| 126 UniRef90\_A0A1Y2MDA3\_48\_437 | - | - | - | - | - | - | - | - | - | - | - | - | - | - | - | - | - | - | - | - | - | - | - | - | - | - | - | - | - | A | Q | D | V | A | D | V | L | A | V | D | A | A | V | R | D | Q | E | N | K | S |
| 127 UniRef90\_UPI000562F0C5\_9\_388 | - | - | - | - | - | - | - | - | - | - | - | - | - | - | - | - | - | - | E | A | E | - | A | L | - | - | - | A | V | A | A | A | L | A | D | E | F | R | P | G | A | C | E | R | D | A | R | R | R | L |
| 128 UniRef90\_A0A506Y8Z1\_43\_442 | - | - | - | - | - | - | - | - | - | - | - | - | S | A | R | P | S | D | A | A | G | - | W | I | - | - | - | A | R | A | R | E | V | A | D | I | L | A | A | D | A | V | D | R | D | R | A | G | A | P |
| 129 UniRef90\_A0A021VVW0\_28\_409 | - | - | - | - | - | - | - | - | - | - | - | - | - | - | - | - | - | - | - | - | - | - | - | - | - | - | - | A | R | A | E | E | V | A | A | I | L | A | V | D | A | V | E | R | D | R | A | L | R | T |
| 130 UniRef90\_E3QTS2\_46\_428 | - | - | - | - | - | - | - | - | - | - | - | - | - | - | - | - | - | - | - | - | - | - | - | - | - | - | - | - | - | - | - | - | - | - | - | - | - | - | R | D | A | A | L | R | E | R | E | N | K | S |
| 131 UniRef90\_A0A1D8SMV7\_7\_388 | - | - | - | - | - | - | - | - | - | - | - | - | - | - | - | - | A | D | D | A | E | - | A | L | - | - | - | A | V | A | T | A | L | A | D | E | F | R | P | G | A | S | A | R | D | S | E | R | R | L |
| 132 UniRef90\_A9BUV8\_38\_419 | - | - | - | - | - | - | - | - | - | - | - | - | - | - | - | - | - | - | - | - | E | - | A | L | - | - | - | E | A | A | R | S | F | A | A | L | I | A | P | G | A | L | A | R | D | R | D | R | L | L |
| 133 UniRef90\_A0A1N6TB45\_32\_407 | - | - | - | - | - | - | - | - | - | - | - | - | - | - | - | - | - | - | - | - | - | - | - | - | - | - | - | - | - | A | A | A | L | R | P | L | F | A | R | D | A | A | E | R | D | R | A | G | G | R |
| 134 UniRef90\_A0A381IE41\_24\_406 | - | - | - | - | - | - | - | - | - | - | - | - | - | - | - | - | A | S | D | A | E | - | A | L | - | - | - | D | V | A | R | A | L | A | A | R | L | A | Q | G | A | A | E | R | D | R | E | R | R | L |
| 135 UniRef90\_A0A3M8TJ33\_18\_397 | - | - | - | - | - | - | - | - | - | - | - | - | - | - | - | - | - | - | - | - | - | - | - | - | - | - | - | Q | V | A | H | E | A | A | D | D | L | A | T | D | A | V | V | R | D | Q | A | G | K | T |
| 136 UniRef90\_A0A1H3JL41\_4\_373 | - | - | - | - | - | - | - | - | - | - | - | - | - | - | - | - | - | - | - | - | - | - | - | - | - | - | - | A | S | A | K | A | V | A | D | R | L | R | A | D | A | A | D | R | D | R | A | N | R | R |
| 137 UniRef90\_A0A507AW89\_49\_427 | - | - | - | - | - | - | - | - | - | - | - | - | - | - | - | - | - | - | - | - | - | - | - | - | - | - | - | - | - | - | - | - | - | - | - | - | - | - | - | - | - | G | L | R | E | R | E | N | K | S |
| 138 UniRef90\_A0A1X1SEN2\_20\_406 | - | - | - | - | - | - | - | - | - | - | - | - | - | - | - | - | - | - | - | - | - | - | - | - | - | - | - | - | - | - | - | - | V | R | Q | E | L | A | R | T | A | V | A | R | D | R | A | A | G | T |
| 139 UniRef90\_A0A2D3UFX4\_26\_403 | - | - | - | - | - | - | - | - | - | - | - | - | - | - | - | - | - | - | - | - | - | - | - | - | - | - | - | R | V | A | R | E | T | A | D | D | L | A | T | D | A | V | A | R | E | Q | A | G | K | A |
| 140 UniRef90\_A0A089X0S9\_26\_403 | - | - | - | - | - | - | - | - | - | - | - | - | - | - | - | - | - | - | - | - | - | - | - | - | - | - | - | R | V | I | R | E | T | A | D | D | L | A | T | D | A | V | E | R | E | Q | A | G | K | A |
| 141 UniRef90\_A0A2W5T1D0\_29\_413 | - | - | - | - | - | - | - | - | - | - | - | - | - | - | - | - | - | - | - | - | - | - | - | L | - | - | - | A | R | A | E | E | V | S | Q | I | L | A | A | D | A | V | E | R | D | R | A | G | A | S |
| 142 UniRef90\_A0A1C4QGR9\_7\_388 | - | - | - | - | - | - | - | - | - | - | - | - | - | - | - | - | A | D | D | Q | E | - | A | L | - | - | - | A | V | A | R | A | L | A | E | E | F | R | A | G | A | S | E | R | D | R | E | R | R | L |
| 143 UniRef90\_A0A101R4L6\_7\_388 | - | - | - | - | - | - | - | - | - | - | - | - | - | - | - | - | A | D | D | A | E | - | A | L | - | - | - | A | V | A | A | E | L | A | D | A | F | R | A | G | A | A | R | R | D | A | E | R | V | L |
| 144 UniRef90\_A0A4D4LHU8\_7\_388 | - | - | - | - | - | - | - | - | - | - | - | - | - | - | - | - | A | D | D | A | E | - | A | L | - | - | - | S | V | A | A | A | L | A | D | E | F | R | A | G | A | S | G | R | D | A | E | R | R | L |
| 145 UniRef90\_L1KK35\_6\_389 | - | - | - | - | - | - | - | - | - | - | - | - | - | - | - | - | - | D | D | A | E | - | A | L | - | - | - | A | V | A | A | E | L | A | A | D | F | R | K | G | A | A | E | R | D | A | R | R | R | L |
| 146 UniRef90\_UPI0005601C6B\_13\_390 | - | - | - | - | - | - | - | - | - | - | - | - | - | - | - | - | - | - | - | - | E | - | A | L | - | - | - | D | V | A | A | R | L | A | A | G | F | R | V | D | A | D | T | R | D | A | E | R | R | L |
| 147 UniRef90\_A0A1H9UQJ9\_6\_361 | - | - | - | - | - | - | - | - | - | - | - | - | - | - | - | - | - | - | - | - | - | - | - | - | - | - | - | D | V | A | R | D | V | A | A | T | L | R | S | D | A | A | D | R | D | R | A | N | Q | P |
| 148 UniRef90\_A0A3D9JKY9\_12\_401 | - | - | - | - | - | - | - | - | - | - | - | - | - | - | - | G | A | G | Q | A | D | - | W | L | - | - | - | H | I | A | R | E | M | A | D | D | L | A | T | D | A | V | E | R | E | Q | A | G | K | A |
| 149 UniRef90\_D9X7Q2\_26\_403 | - | - | - | - | - | - | - | - | - | - | - | - | - | - | - | - | - | - | - | - | - | - | - | - | - | - | - | R | V | A | R | E | T | A | D | D | L | A | T | D | A | V | T | R | E | Q | A | G | K | P |
| 150 UniRef90\_A0A1X1DXJ3\_20\_398 | - | - | - | - | - | - | - | - | - | - | - | - | - | - | - | - | - | - | - | D | Q | - | A | I | - | - | - | Q | A | A | H | S | L | A | E | Q | A | K | P | G | A | V | E | R | D | Q | Q | R | I | Y |

  
  

|  |  |  |  |  |  |  |  |  |  |  |  |  |  |  |  |  |  |  |  |  |  |  |  |  |  |  |  |  |  |  |  |  |  |  |  |  |  |  |  |  |  |  |  |  |  |  |  |  |  |  |
| --- | --- | --- | --- | --- | --- | --- | --- | --- | --- | --- | --- | --- | --- | --- | --- | --- | --- | --- | --- | --- | --- | --- | --- | --- | --- | --- | --- | --- | --- | --- | --- | --- | --- | --- | --- | --- | --- | --- | --- | --- | --- | --- | --- | --- | --- | --- | --- | --- | --- | --- |
| **001 Input\_pdb\_SEQRES\_A** | A | T | A | E | R | E | D | L | R | A | S | G | L | L | S | L | L | V | P | R | E | Y | G | G | - | - | - | - | - | - | - | - | - | - | - | - | - | W | G | A | D | W | P | T | A | I | E | V | V | R |
| 002 UniRef90\_Q1W1G3\_1\_416 | A | T | V | E | R | E | D | L | R | A | S | G | L | L | S | L | L | I | P | R | Q | Y | G | G | - | - | - | - | - | - | - | - | - | - | - | - | - | W | G | A | D | W | P | T | A | I | E | V | V | R |
| 003 UniRef90\_T0BM21\_7\_392 | A | K | R | Q | R | D | M | L | R | E | S | G | L | L | T | L | L | I | P | E | R | Y | G | G | - | - | - | - | - | - | - | - | - | - | - | - | - | G | G | E | T | W | S | T | V | L | R | I | V | R |
| 004 UniRef90\_UPI0002AC58FE\_11\_391 | P | K | Q | E | R | D | R | L | R | Q | S | G | L | L | K | L | I | I | P | K | E | Y | G | G | - | - | - | - | - | - | - | - | - | - | - | - | - | L | G | E | T | W | I | T | T | L | K | I | S | R |
| 005 UniRef90\_A0A3D5CW51\_20\_401 | P | K | L | E | R | D | A | I | R | N | S | G | L | L | S | L | I | I | P | R | Q | Y | G | G | - | - | - | - | - | - | - | - | - | - | - | - | - | Y | G | A | N | W | Q | E | T | L | Q | V | V | R |
| 006 UniRef90\_A0A2V4SPP5\_16\_400 | P | K | H | E | R | E | L | I | R | D | S | G | L | L | T | L | S | V | P | A | E | F | G | G | - | - | - | - | - | - | - | - | - | - | - | - | - | Q | G | A | D | W | A | T | V | M | A | A | V | R |
| 007 UniRef90\_E0UIV5\_8\_393 | P | K | P | E | R | D | R | L | R | E | S | G | L | L | K | L | I | I | P | K | E | Y | G | G | - | - | - | - | - | - | - | - | - | - | - | - | - | L | A | E | T | W | L | T | L | F | K | I | T | R |
| 008 UniRef90\_A0A1Z4S897\_9\_391 | P | T | E | E | L | K | K | L | R | E | S | G | L | L | N | L | V | I | P | K | E | Y | G | G | - | - | - | - | - | - | - | - | - | - | - | - | - | I | G | E | T | W | P | N | V | L | K | V | V | R |
| 009 UniRef90\_UPI00045E9273\_21\_396 | A | K | N | E | R | D | S | L | R | Q | S | G | L | L | N | L | I | I | P | G | E | Y | G | G | - | - | - | - | - | - | - | - | - | - | - | - | - | H | G | L | D | W | H | D | T | L | Q | I | V | R |
| 010 UniRef90\_A0A353Y4M9\_17\_384 | A | A | A | E | R | A | L | L | R | D | S | G | L | L | A | L | A | V | P | A | C | F | G | G | - | - | - | - | - | - | - | - | - | - | - | - | - | Q | G | A | D | W | P | T | V | L | R | A | V | R |
| 011 UniRef90\_F3KR10\_17\_384 | A | A | A | E | R | A | L | L | R | D | S | G | L | L | T | L | A | V | P | A | K | F | G | G | - | - | - | - | - | - | - | - | - | - | - | - | - | Q | G | E | R | W | P | V | I | L | R | A | I | R |
| 012 UniRef90\_A0A2D8NW56\_20\_404 | A | A | E | A | R | E | L | L | R | Q | Q | G | L | L | R | L | S | V | P | A | W | A | G | G | - | - | - | - | - | - | - | - | - | - | - | - | - | V | G | A | S | W | S | L | I | Y | H | V | V | R |
| 013 UniRef90\_A0A2W7M9P1\_16\_386 | A | K | K | E | R | D | L | I | R | K | S | G | L | L | R | L | A | S | P | K | A | Y | G | G | - | - | - | - | - | - | - | - | - | - | - | - | - | E | K | A | D | W | S | T | I | L | H | I | T | R |
| 014 UniRef90\_A0A398AYR9\_12\_386 | A | K | E | E | R | D | L | I | R | H | S | G | L | L | R | L | T | A | P | E | Q | Y | G | G | - | - | - | - | - | - | - | - | - | - | - | - | - | Y | G | E | N | W | K | R | V | L | H | I | T | R |
| 015 UniRef90\_A0A0T6UXN9\_11\_394 | P | K | A | E | R | D | A | I | R | A | S | G | L | L | S | L | I | I | P | T | Q | Y | G | G | - | - | - | - | - | - | - | - | - | - | - | - | - | L | G | A | T | W | S | E | T | F | E | V | V | R |
| 016 UniRef90\_A0A252E884\_11\_390 | P | D | I | E | I | Q | R | L | R | E | S | G | L | L | P | L | V | V | P | K | E | Y | G | G | - | - | - | - | - | - | - | - | - | - | - | - | - | T | G | A | T | W | A | E | A | F | K | V | I | Q |
| 017 UniRef90\_A0A1Z4BZ71\_24\_403 | P | L | A | Q | R | Q | A | I | R | D | S | G | L | L | K | L | S | I | A | K | E | F | G | G | - | - | - | - | - | - | - | - | - | - | - | - | - | Y | G | L | A | W | P | D | I | Y | K | I | I | R |
| 018 UniRef90\_A0A1Z4IGC5\_10\_387 | A | K | H | E | R | S | H | L | R | Q | S | G | L | L | K | L | I | I | P | K | E | Y | G | G | - | - | - | - | - | - | - | - | - | - | - | - | - | L | G | E | T | W | I | T | T | L | Q | I | V | R |
| 019 UniRef90\_A0A1W9JD13\_18\_401 | P | K | A | E | R | D | M | L | R | E | S | G | L | L | S | L | T | I | P | K | E | Y | G | G | - | - | - | - | - | - | - | - | - | - | - | - | - | Q | G | A | S | W | H | E | T | L | L | V | I | R |
| 020 UniRef90\_A0A0B6S5D2\_8\_393 | A | A | R | E | K | Q | W | M | A | A | V | G | L | Q | T | L | A | V | P | R | A | F | G | G | - | - | - | - | - | - | - | - | - | - | - | - | - | Q | E | A | E | W | P | I | V | Y | Q | T | I | R |
| 021 UniRef90\_A0A1Y3C786\_14\_396 | P | K | Y | E | R | D | L | I | R | Q | S | G | L | L | A | L | S | I | P | Q | E | F | G | G | - | - | - | - | - | - | - | - | - | - | - | - | - | F | S | G | T | W | K | D | T | F | D | V | V | R |
| 022 UniRef90\_A0A318KD41\_20\_397 | P | K | A | E | R | D | A | L | R | D | S | G | L | L | T | L | I | I | P | R | E | Y | G | G | - | - | - | - | - | - | - | - | - | - | - | - | - | L | G | A | G | W | Q | D | T | L | N | V | V | R |
| 023 UniRef90\_A0A352JDP6\_15\_389 | P | K | Y | E | R | D | R | L | R | D | L | G | L | L | K | I | A | I | P | K | A | Y | G | G | - | - | - | - | - | - | - | - | - | - | - | - | - | W | E | L | P | W | H | E | V | L | R | I | S | R |
| 024 UniRef90\_A0A329B538\_13\_401 | P | K | A | E | R | D | A | L | R | R | S | G | L | L | T | L | A | I | P | R | E | F | G | G | - | - | - | - | - | - | - | - | - | - | - | - | - | L | G | A | S | W | S | E | M | L | T | I | V | R |
| 025 UniRef90\_A0A2N8QAF3\_13\_394 | A | A | Q | E | K | Q | W | I | A | D | A | G | L | L | T | L | A | V | P | R | E | L | G | G | - | - | - | - | - | - | - | - | - | - | - | - | - | L | G | A | R | W | P | E | I | Y | E | T | I | R |
| 026 UniRef90\_A0A2N7XWT8\_11\_394 | P | K | A | E | R | D | K | L | R | S | S | G | L | L | S | L | S | I | P | G | Q | F | G | G | - | - | - | - | - | - | - | - | - | - | - | - | - | L | D | A | N | W | S | E | T | F | E | V | V | R |
| 027 UniRef90\_A0A2A4HLB2\_33\_412 | A | S | F | E | R | E | L | I | R | D | S | G | L | L | T | L | A | V | P | T | E | Y | G | G | - | - | - | - | - | - | - | - | - | - | - | - | - | Q | G | Q | P | W | S | L | V | Y | H | V | V | R |
| 028 UniRef90\_A0A4R3HWL3\_41\_416 | A | W | A | E | R | Q | A | L | R | D | S | G | L | L | T | L | A | V | P | A | A | F | G | G | - | - | - | - | - | - | - | - | - | - | - | - | - | P | E | A | S | W | P | F | I | Y | R | V | I | R |
| 029 UniRef90\_A0A1M7NXP6\_14\_394 | A | A | Q | E | R | E | W | I | R | E | S | G | L | L | T | L | S | I | P | T | A | F | G | G | - | - | - | - | - | - | - | - | - | - | - | - | - | Q | G | A | D | W | P | T | V | Y | Q | V | I | R |
| 030 UniRef90\_A0A1B4ESK2\_22\_397 | A | A | D | E | K | Q | R | L | A | D | A | G | L | L | T | L | A | V | P | R | E | F | G | G | - | - | - | - | - | - | - | - | - | - | - | - | - | Q | E | A | E | W | P | A | I | Y | D | T | I | R |
| 031 UniRef90\_A0A1H6NKV2\_11\_394 | P | K | A | Q | R | D | A | L | R | H | S | G | L | L | A | L | S | I | P | T | Q | Y | G | G | - | - | - | - | - | - | - | - | - | - | - | - | - | L | G | A | R | W | S | E | T | L | S | V | V | R |
| 032 UniRef90\_A0A0D0KUF8\_11\_394 | P | K | A | E | R | D | A | I | R | Q | S | G | L | L | A | L | S | I | P | T | Q | F | G | G | - | - | - | - | - | - | - | - | - | - | - | - | - | L | G | A | S | W | T | E | T | L | G | V | V | R |
| 033 UniRef90\_A0A0F3K7B4\_20\_401 | P | K | A | E | R | D | A | I | R | A | S | G | L | L | G | L | S | I | A | S | E | L | G | G | - | - | - | - | - | - | - | - | - | - | - | - | - | L | G | A | N | W | Q | Q | T | L | L | V | V | R |
| 034 UniRef90\_A0A381IMG6\_13\_394 | A | A | R | E | K | R | W | I | A | D | A | G | L | L | T | L | A | V | P | R | E | F | G | G | - | - | - | - | - | - | - | - | - | - | - | - | - | Q | E | A | G | W | P | V | I | Y | H | T | I | R |
| 035 UniRef90\_A0A2S9K1G1\_26\_400 | A | A | A | E | R | E | L | I | R | A | S | G | L | L | D | L | T | T | P | C | A | Y | G | G | - | - | - | - | - | - | - | - | - | - | - | - | - | W | G | Q | S | W | Q | T | F | Y | A | G | L | R |
| 036 UniRef90\_A0A1W6L7W5\_17\_392 | A | A | E | E | R | Q | W | I | R | D | S | G | L | L | S | L | T | I | P | A | A | H | G | G | - | - | - | - | - | - | - | - | - | - | - | - | - | Q | G | A | D | W | P | T | F | Y | R | T | L | R |
| 037 UniRef90\_A0A255HJE3\_14\_398 | A | A | E | E | R | E | W | I | R | D | S | G | L | L | T | L | S | I | P | A | Q | F | G | G | - | - | - | - | - | - | - | - | - | - | - | - | - | Q | G | A | P | W | P | L | V | Y | Q | V | I | R |
| 038 UniRef90\_A0A0R3AD45\_11\_394 | P | K | T | E | R | D | A | L | R | Q | S | G | L | L | A | L | S | I | P | T | Q | Y | G | G | - | - | - | - | - | - | - | - | - | - | - | - | - | L | G | A | R | W | S | D | T | L | A | I | V | R |
| 039 UniRef90\_A0A1W6ZB60\_16\_389 | A | A | H | E | R | E | L | I | R | A | S | G | L | L | A | L | S | V | P | T | E | F | G | G | - | - | - | - | - | - | - | - | - | - | - | - | - | A | Q | A | P | W | S | V | T | L | A | A | V | R |
| 040 UniRef90\_A0A2X1DPB7\_14\_397 | A | A | Q | E | K | Q | W | L | A | D | A | G | L | L | T | L | A | V | P | R | A | F | G | G | - | - | - | - | - | - | - | - | - | - | - | - | - | Q | E | A | A | W | P | A | I | Y | D | V | I | R |
| 041 UniRef90\_A0A0S9M2D1\_4\_380 | P | K | A | E | R | D | A | L | R | D | S | G | L | L | A | L | S | I | P | R | A | Y | G | G | - | - | - | - | - | - | - | - | - | - | - | - | - | H | G | G | D | W | Q | L | T | L | R | V | V | R |
| 042 UniRef90\_A0A238ZKW1\_13\_401 | P | K | I | E | R | D | L | I | R | N | S | G | L | L | A | L | S | I | P | G | T | E | G | G | - | - | - | - | - | - | - | - | - | - | - | - | - | L | G | A | N | W | Q | E | T | M | Q | I | V | R |
| 043 UniRef90\_A0A0D1P8V6\_10\_394 | P | K | A | E | R | D | A | L | R | A | S | G | L | L | A | L | S | I | P | R | E | F | G | G | - | - | - | - | - | - | - | - | - | - | - | - | - | L | N | A | T | W | S | Q | T | F | D | V | V | R |
| 044 UniRef90\_A0A261SP68\_11\_387 | A | A | A | E | R | E | L | I | R | A | S | G | L | L | L | L | S | I | P | R | E | H | G | G | - | - | - | - | - | - | - | - | - | - | - | - | - | L | E | T | D | W | S | T | I | L | G | I | V | R |
| 045 UniRef90\_A0A2N6MRB2\_11\_390 | P | D | V | E | I | Q | L | L | R | E | S | G | L | L | P | L | V | V | P | K | E | Y | G | G | - | - | - | - | - | - | - | - | - | - | - | - | - | T | G | A | T | W | I | E | A | L | R | I | V | Q |
| 046 UniRef90\_A0A212BVA1\_31\_405 | A | R | A | E | R | E | L | I | R | E | S | G | L | L | A | L | A | I | P | Q | R | F | D | G | - | - | - | - | - | - | - | - | - | - | - | - | - | L | E | K | P | W | P | Q | I | Y | R | I | V | R |
| 047 UniRef90\_A0A1A9KE82\_11\_394 | P | K | A | E | R | D | A | I | R | H | S | G | L | L | A | L | S | I | P | G | A | F | G | G | - | - | - | - | - | - | - | - | - | - | - | - | - | H | G | A | D | W | S | L | T | L | E | V | V | R |
| 048 UniRef90\_A0A4Q5PVB9\_26\_400 | A | L | A | E | R | R | L | L | R | E | S | G | L | L | T | L | A | V | P | G | E | H | G | G | - | - | - | - | - | - | - | - | - | - | - | - | - | H | G | A | G | W | P | L | I | F | R | I | V | R |
| 049 UniRef90\_A0A178GPM9\_9\_392 | P | K | A | E | R | D | L | I | R | Q | S | G | L | L | G | L | S | I | P | K | Q | Y | G | G | - | - | - | - | - | - | - | - | - | - | - | - | - | Q | E | A | D | W | Q | T | I | F | K | T | I | Q |
| 050 UniRef90\_A0A1P9YC32\_3\_390 | A | F | H | E | R | R | L | L | R | E | S | G | L | L | R | L | S | V | P | V | E | H | G | G | - | - | - | - | - | - | - | - | - | - | - | - | - | D | G | A | P | W | S | V | T | L | D | V | V | R |
| 051 UniRef90\_UPI000A1773A9\_40\_416 | P | K | L | Q | R | D | Q | L | R | R | S | G | L | L | A | L | S | I | P | T | A | F | G | G | - | - | - | - | - | - | - | - | - | - | - | - | - | L | G | G | D | W | K | L | V | L | D | V | V | R |
| 052 UniRef90\_A0A158L201\_25\_395 | A | A | R | E | K | Q | L | I | A | D | A | G | L | L | T | I | A | V | P | R | E | F | G | G | - | - | - | - | - | - | - | - | - | - | - | - | - | E | G | A | R | W | V | D | V | Y | E | T | I | R |
| 053 UniRef90\_A0A4Q4GT15\_13\_391 | A | K | V | E | R | D | L | I | R | Q | S | G | L | L | S | L | S | I | P | K | Q | Y | G | G | - | - | - | - | - | - | - | - | - | - | - | - | - | Q | G | A | D | W | A | T | I | F | Q | T | V | R |
| 054 UniRef90\_A0A1C0YC62\_17\_381 | A | Y | E | E | R | Q | L | L | R | Q | S | G | L | L | Y | L | - | - | - | - | - | F | Q | D | - | - | - | - | - | - | - | - | - | - | - | - | - | G | D | I | P | W | Q | T | V | L | R | I | T | R |
| 055 UniRef90\_A0A1H7FE04\_32\_405 | A | A | A | E | R | E | L | I | R | A | S | G | L | L | A | L | T | V | P | Q | A | L | G | G | - | - | - | - | - | - | - | - | - | - | - | - | - | L | G | G | G | V | T | E | L | F | H | A | V | R |
| 056 UniRef90\_A0A401MWC4\_5\_385 | P | F | E | E | A | E | R | L | R | A | A | G | L | L | T | L | L | V | P | A | E | Y | G | G | - | - | - | - | - | - | - | - | - | - | - | - | - | G | G | A | D | W | R | T | A | Y | A | I | I | R |
| 057 UniRef90\_A0A1G6HFW7\_16\_395 | A | L | H | E | R | R | V | L | R | D | S | G | L | L | G | L | S | V | P | R | A | Y | G | G | - | - | - | - | - | - | - | - | - | - | - | - | - | H | G | A | S | W | S | D | T | L | A | V | V | R |
| 058 UniRef90\_A0A0P9B999\_30\_403 | A | L | A | E | R | A | L | L | R | R | S | G | L | L | T | L | A | I | P | A | A | H | G | G | - | - | - | - | - | - | - | - | - | - | - | - | - | Q | Q | A | A | W | P | L | I | F | R | I | L | R |
| 059 UniRef90\_A0A140K6F0\_14\_392 | P | E | A | E | I | N | L | F | K | K | S | G | L | L | L | L | S | I | P | K | K | Y | G | G | - | - | - | - | - | - | - | - | - | - | - | - | - | M | E | A | S | W | L | E | V | Y | R | V | I | Q |
| 060 UniRef90\_A0A1P8EKI7\_5\_392 | A | K | I | E | R | D | L | I | R | A | S | G | L | L | S | L | S | I | P | K | A | Y | G | G | - | - | - | - | - | - | - | - | - | - | - | - | - | Q | G | A | D | W | E | T | I | F | K | T | I | Q |
| 061 UniRef90\_A0A2U3MYZ3\_7\_392 | P | K | F | E | R | D | L | I | R | Q | S | G | L | L | A | L | S | I | P | E | Q | Y | G | G | - | - | - | - | - | - | - | - | - | - | - | - | - | Q | G | A | N | W | D | T | I | F | Q | T | I | R |
| 062 UniRef90\_A0A1H2EPX5\_7\_397 | A | Q | A | E | R | D | L | L | R | A | S | G | L | L | T | L | A | I | P | E | A | L | G | G | - | - | - | - | - | - | - | - | - | - | - | - | - | Q | G | A | P | W | P | E | I | L | G | H | V | R |
| 063 UniRef90\_UPI00041EF43F\_25\_400 | A | H | E | E | R | E | L | I | R | G | S | G | L | L | D | M | T | T | P | H | A | F | G | G | - | - | - | - | - | - | - | - | - | - | - | - | - | W | G | H | S | W | Q | L | F | F | Q | G | L | R |
| 064 UniRef90\_D0IW93\_17\_384 | A | A | A | E | K | A | L | L | R | D | A | G | L | L | R | L | A | I | P | R | E | H | G | G | - | - | - | - | - | - | - | - | - | - | - | - | - | D | A | L | S | W | P | D | I | Y | R | H | V | R |
| 065 UniRef90\_A0A395D1I6\_11\_388 | A | K | R | E | R | D | L | I | R | E | S | G | L | L | R | L | S | I | P | V | E | L | G | G | - | - | - | - | - | - | - | - | - | - | - | - | - | A | G | A | D | W | V | E | T | L | G | V | V | R |
| 066 UniRef90\_A0A1H0TK45\_27\_400 | A | K | A | E | R | D | A | I | R | A | S | G | L | L | G | L | S | V | P | T | A | L | G | G | - | - | - | - | - | - | - | - | - | - | - | - | - | L | G | A | S | W | Q | Q | T | L | E | V | V | R |
| 067 UniRef90\_A0A1H8MYU5\_23\_403 | P | S | D | Q | I | R | R | L | K | E | S | G | L | V | N | L | L | I | P | R | E | F | G | G | - | - | - | - | - | - | - | - | - | - | - | - | - | E | G | Q | P | Y | S | T | A | L | R | I | V | R |
| 068 UniRef90\_A0A1B1M254\_5\_403 | P | Y | A | E | V | Q | L | L | K | D | S | G | L | V | T | L | L | G | P | T | E | H | G | G | - | - | - | - | - | - | - | - | - | - | - | - | - | A | G | Q | D | W | P | T | A | Y | R | V | V | R |
| 069 UniRef90\_A0A484THM3\_28\_401 | P | Q | A | E | R | Q | R | L | R | D | S | G | L | L | T | L | A | V | P | R | E | H | G | G | - | - | - | - | - | - | - | - | - | - | - | - | - | A | G | E | R | W | P | V | L | L | R | I | V | R |
| 070 UniRef90\_A0A0Q8Q8X6\_8\_377 | A | A | A | E | K | E | L | I | R | A | H | G | L | L | G | L | S | I | P | R | H | Y | G | G | - | - | - | - | - | - | - | - | - | - | - | - | - | D | G | L | P | W | P | V | I | Y | N | C | V | R |
| 071 UniRef90\_A0A0Q5QDL2\_32\_410 | P | H | A | E | V | A | L | L | K | E | S | G | L | V | T | L | L | G | P | V | E | H | G | G | - | - | - | - | - | - | - | - | - | - | - | - | - | G | G | Q | N | W | V | T | A | Y | E | V | I | R |
| 072 UniRef90\_A0A1A5XM62\_22\_406 | A | A | A | E | K | Q | L | I | A | D | A | G | L | L | A | L | A | V | P | R | E | F | G | G | A | F | D | D | A | F | D | D | A | T | G | A | Q | N | G | A | R | W | P | E | I | Y | R | T | I | R |
| 073 UniRef90\_A0A239EI90\_21\_397 | P | V | D | E | V | Q | L | L | K | D | S | G | L | V | T | L | L | G | P | V | E | H | G | G | - | - | - | - | - | - | - | - | - | - | - | - | - | G | G | Q | D | W | P | T | A | Y | Q | V | I | R |
| 074 UniRef90\_S5SWW2\_23\_403 | P | R | E | Q | V | R | H | L | K | E | S | G | L | V | N | L | L | I | P | K | E | F | G | G | - | - | - | - | - | - | - | - | - | - | - | - | - | E | G | Q | P | Y | S | T | A | L | R | I | V | R |
| 075 UniRef90\_A0A3A5JJY9\_31\_413 | A | A | A | E | R | A | L | I | R | G | S | G | L | L | D | L | S | I | P | A | A | I | G | G | - | - | - | - | - | - | - | - | - | - | - | - | - | A | G | Q | S | W | A | T | I | H | G | V | V | R |
| 076 UniRef90\_A0A315ZU85\_7\_384 | P | L | A | E | A | Q | L | L | R | E | H | R | L | P | G | L | L | L | P | V | E | V | G | G | - | - | - | - | - | - | - | - | - | - | - | - | - | G | G | G | T | W | A | D | A | L | R | A | V | R |
| 077 UniRef90\_A0A3R9U899\_5\_381 | P | Y | A | E | V | Q | L | L | K | D | S | G | L | V | T | L | L | G | P | V | A | H | G | G | - | - | - | - | - | - | - | - | - | - | - | - | - | G | G | Q | D | W | R | T | A | Y | R | V | V | R |
| 078 UniRef90\_UPI000DD53D91\_7\_389 | P | V | D | Q | I | R | L | L | K | E | S | G | L | A | S | A | Q | I | P | R | D | Y | G | G | - | - | - | - | - | - | - | - | - | - | - | - | - | R | G | A | S | W | L | T | I | L | R | I | V | R |
| 079 UniRef90\_UPI000DE4BE09\_13\_396 | P | L | A | E | I | V | K | L | K | N | S | G | L | L | N | A | L | H | A | P | E | I | G | G | - | - | - | - | - | - | - | - | - | - | - | - | - | G | G | L | D | W | V | D | G | L | K | L | V | R |
| 080 UniRef90\_A0A2T0R7P7\_10\_381 | P | F | T | E | A | E | L | L | R | K | A | G | L | P | S | V | L | L | P | A | S | I | G | G | - | - | - | - | - | - | - | - | - | - | - | - | - | A | G | L | P | W | S | V | A | L | E | V | V | R |
| 081 UniRef90\_A0A4R1HYS3\_27\_404 | P | Y | D | E | I | A | L | L | K | R | S | G | L | V | T | L | L | G | P | V | E | H | G | G | - | - | - | - | - | - | - | - | - | - | - | - | - | A | G | L | E | W | P | A | A | Y | R | V | V | R |
| 082 UniRef90\_UPI000D1537B0\_7\_385 | P | C | A | E | I | A | L | L | K | R | S | G | L | L | E | A | M | V | P | Q | A | W | G | G | - | - | - | - | - | - | - | - | - | - | - | - | - | G | G | H | P | W | S | A | V | L | A | V | V | R |
| 083 UniRef90\_A0A1G6ZNM7\_17\_409 | P | Y | A | E | V | Q | L | L | K | D | A | G | L | V | P | L | L | A | P | A | E | H | G | G | - | - | - | - | - | - | - | - | - | - | - | - | - | G | G | Q | D | W | T | T | A | Y | R | V | I | R |
| 084 UniRef90\_UPI0003765980\_18\_389 | P | L | E | Q | I | R | I | L | K | E | S | G | L | P | S | A | Q | I | A | K | A | Y | G | G | - | - | - | - | - | - | - | - | - | - | - | - | - | Q | G | A | S | W | L | T | I | L | R | I | V | R |
| 085 UniRef90\_A0A071ICK8\_25\_398 | P | L | K | E | I | I | R | L | K | N | S | G | L | L | N | A | L | H | A | P | Q | I | G | G | - | - | - | - | - | - | - | - | - | - | - | - | - | G | G | L | N | W | V | D | A | L | K | L | V | R |
| 086 UniRef90\_A0A2L0WNE5\_33\_396 | P | L | A | E | I | I | K | L | K | K | A | G | L | L | N | A | L | H | S | R | E | I | G | G | - | - | - | - | - | - | - | - | - | - | - | - | - | G | G | L | D | W | V | D | G | L | R | L | V | R |
| 087 UniRef90\_A0A1Q8CFJ2\_9\_380 | P | E | A | E | V | A | L | L | R | A | S | G | L | L | T | L | L | L | P | A | S | A | G | G | - | - | - | - | - | - | - | - | - | - | - | - | - | R | G | A | G | W | L | A | A | N | E | T | A | R |
| 088 UniRef90\_UPI000835F605\_21\_394 | P | F | E | E | I | E | L | L | R | K | A | G | L | L | G | L | A | V | P | R | A | L | G | G | - | - | - | - | - | - | - | - | - | - | - | - | - | A | G | A | N | L | A | Q | A | L | E | I | S | R |
| 089 UniRef90\_UPI000374AB86\_31\_398 | P | L | N | E | I | V | K | L | K | N | A | G | L | L | N | A | L | H | A | P | A | L | G | G | - | - | - | - | - | - | - | - | - | - | - | - | - | G | G | L | D | W | V | D | G | L | K | L | V | R |
| 090 UniRef90\_A0A267RVL6\_22\_399 | P | F | E | E | I | D | W | L | R | Q | S | G | L | L | K | L | A | V | P | K | S | L | G | G | - | - | - | - | - | - | - | - | - | - | - | - | - | G | G | A | N | L | V | Q | A | L | E | I | G | R |
| 091 UniRef90\_M3VA40\_23\_395 | P | T | A | E | I | E | L | L | R | S | H | D | L | L | Q | V | G | E | P | V | E | L | G | G | - | - | - | - | - | - | - | - | - | - | - | - | - | S | G | L | N | Y | A | Q | T | Q | Q | I | T | R |
| 092 UniRef90\_A0A395GID9\_32\_425 | P | R | A | E | V | V | L | L | K | H | S | G | L | L | K | L | L | G | P | K | K | Y | G | G | - | - | - | - | - | - | - | - | - | - | - | - | - | G | E | Q | P | W | D | I | G | Y | K | A | I | R |
| 093 UniRef90\_UPI0008268C2F\_35\_406 | P | L | A | E | V | A | L | L | R | E | A | D | L | L | Q | V | G | E | P | V | A | Y | G | G | - | - | - | - | - | - | - | - | - | - | - | - | - | S | G | L | D | Y | A | Q | S | Q | Q | I | T | R |
| 094 UniRef90\_A0A0S1XV73\_27\_397 | P | V | A | Q | I | A | L | L | K | Q | Q | K | L | N | A | A | A | I | A | P | Q | Y | G | G | - | - | - | - | - | - | - | - | - | - | - | - | - | E | G | A | S | W | V | S | I | L | R | V | V | R |
| 095 UniRef90\_A0A0F5N116\_19\_398 | P | R | A | E | I | E | L | L | R | R | N | D | L | L | H | V | Q | E | P | V | E | Y | G | G | - | - | - | - | - | - | - | - | - | - | - | - | - | S | G | L | S | Y | P | Q | A | S | Q | V | T | R |
| 096 UniRef90\_A0A1N6Z7J9\_40\_406 | P | L | E | D | I | T | T | L | K | A | A | G | L | L | T | A | L | H | P | T | E | I | G | G | - | - | - | - | - | - | - | - | - | - | - | - | - | G | G | L | D | W | I | D | G | L | R | L | V | R |
| 097 UniRef90\_A0A2V4UWQ2\_31\_411 | P | F | S | E | I | T | E | L | K | A | A | G | L | L | N | A | L | H | P | P | E | I | G | G | - | - | - | - | - | - | - | - | - | - | - | - | - | G | G | L | D | W | V | D | G | L | R | L | V | R |
| 098 UniRef90\_A0A4P8KLT1\_5\_401 | P | F | A | E | A | R | L | L | K | D | A | G | L | T | T | L | L | D | P | A | E | Y | G | G | - | - | - | - | - | - | - | - | - | - | - | - | - | G | G | A | H | W | E | S | A | F | R | A | V | R |
| 099 UniRef90\_UPI0003068598\_3\_292 | - | - | - | - | - | - | - | - | - | - | - | - | - | - | - | - | - | - | - | - | - | - | - | - | - | - | - | - | - | - | - | - | - | - | - | - | - | - | - | - | - | - | - | - | - | - | - | - | - | - |
| 100 UniRef90\_UPI000DD585C3\_27\_403 | P | R | D | E | I | E | A | L | R | Q | A | G | L | L | S | A | L | H | A | P | E | V | G | G | - | - | - | - | - | - | - | - | - | - | - | - | - | A | G | L | S | W | V | D | G | L | R | L | V | R |
| 101 UniRef90\_A0A1E3SMN8\_25\_404 | P | R | A | E | I | E | L | L | R | H | N | D | L | L | Q | V | Q | E | P | A | E | Y | G | G | - | - | - | - | - | - | - | - | - | - | - | - | - | S | G | L | N | F | A | Q | A | V | Q | I | V | R |
| 102 UniRef90\_UPI00045EBD18\_12\_396 | P | R | R | E | L | A | L | L | R | D | A | G | L | L | E | L | L | F | S | P | E | H | G | G | - | - | - | - | - | - | - | - | - | - | - | - | - | G | G | G | S | F | T | D | A | L | L | A | V | R |
| 103 UniRef90\_A0A1Y1ZNX6\_42\_434 | P | R | A | E | V | E | L | L | K | H | S | G | L | L | K | L | L | G | P | K | K | Y | G | G | - | - | - | - | - | - | - | - | - | - | - | - | - | G | E | Q | P | W | S | V | G | Y | K | A | I | R |
| 104 UniRef90\_A0A0D2FPI6\_31\_423 | P | F | S | E | I | Q | L | L | K | A | S | G | L | T | K | L | L | G | P | K | N | Y | G | G | - | - | - | - | - | - | - | - | - | - | - | - | - | A | G | Q | D | W | A | I | A | Y | K | A | I | R |
| 105 UniRef90\_A0A2S0KGS2\_30\_403 | P | V | A | E | V | E | L | L | R | T | H | D | L | L | Q | V | G | E | P | V | E | L | G | G | - | - | - | - | - | - | - | - | - | - | - | - | - | D | G | L | N | Y | A | Q | S | Q | Q | L | T | R |
| 106 UniRef90\_A0A1A7MGQ0\_54\_442 | P | F | A | E | I | S | L | L | K | S | S | G | L | L | K | V | L | G | P | T | E | Y | G | G | - | - | - | - | - | - | - | - | - | - | - | - | - | G | G | Q | E | W | E | I | G | Y | K | V | I | R |
| 107 UniRef90\_UPI000413001D\_18\_392 | P | R | R | E | L | E | L | L | R | A | S | G | L | L | A | L | L | N | P | P | A | H | G | G | - | - | - | - | - | - | - | - | - | - | - | - | - | G | G | G | S | F | G | D | A | F | R | A | V | R |
| 108 UniRef90\_G7H5P9\_11\_400 | P | V | A | E | V | D | L | L | R | Q | A | D | L | L | Q | V | G | E | P | V | E | F | G | G | - | - | - | - | - | - | - | - | - | - | - | - | - | S | G | L | N | Y | A | Q | S | Q | Q | L | T | R |
| 109 UniRef90\_A0A010YHQ5\_11\_404 | P | H | K | E | V | E | L | L | R | D | A | G | L | L | P | L | L | I | P | A | R | N | G | G | - | - | - | - | - | - | - | - | - | - | - | - | - | H | G | G | S | W | L | T | A | F | E | V | V | T |
| 110 UniRef90\_B0RBU7\_24\_404 | P | T | A | E | L | D | L | L | R | D | S | G | L | V | N | L | L | D | P | A | E | H | G | G | - | - | - | - | - | - | - | - | - | - | - | - | - | G | G | G | H | W | E | S | A | V | L | A | I | R |
| 111 UniRef90\_A0A167RW81\_42\_429 | P | R | A | E | V | A | L | L | K | H | A | G | L | L | T | V | L | G | P | T | R | Y | G | G | - | - | - | - | - | - | - | - | - | - | - | - | - | G | G | Q | P | W | R | V | G | Y | K | V | V | R |
| 112 UniRef90\_A0A1X0J8A0\_26\_405 | P | S | G | E | I | G | L | L | R | E | Y | G | L | L | S | F | A | T | A | R | E | F | G | G | - | - | - | - | - | - | - | - | - | - | - | - | - | A | G | G | S | L | T | Q | A | L | Q | L | S | R |
| 113 UniRef90\_A0A2J6Q859\_43\_430 | P | R | A | E | I | V | L | L | K | H | A | G | L | T | K | V | L | G | P | K | K | Y | G | G | - | - | - | - | - | - | - | - | - | - | - | - | - | G | G | Q | P | W | A | L | A | Y | K | L | I | R |
| 114 UniRef90\_UPI0003828F02\_7\_389 | P | V | A | E | V | A | L | V | R | D | S | G | L | L | P | L | M | I | P | A | D | H | G | G | - | - | - | - | - | - | - | - | - | - | - | - | - | H | G | E | D | W | A | T | A | H | A | V | L | A |
| 115 UniRef90\_A0A1B2HDJ6\_6\_361 | P | T | K | E | V | E | L | L | R | Q | S | G | L | L | N | V | - | - | - | - | - | - | - | - | - | - | - | - | - | - | - | - | - | - | - | - | - | - | - | S | D | W | A | T | Q | Q | R | I | N | R |
| 116 UniRef90\_A0A2N3N0U1\_47\_427 | P | R | A | E | V | A | L | L | K | Y | A | G | L | L | K | V | L | G | P | K | K | Y | G | G | - | - | - | - | - | - | - | - | - | - | - | - | - | G | E | Q | P | L | S | V | G | Y | K | V | I | R |
| 117 UniRef90\_S5Y4X6\_32\_408 | P | H | A | E | I | E | L | L | R | Q | A | G | L | L | G | L | A | A | P | R | D | F | G | G | - | - | - | - | - | - | - | - | - | - | - | - | - | G | G | A | S | L | L | Q | A | M | E | I | V | R |
| 118 UniRef90\_A0A3R2WQN2\_17\_411 | P | T | A | E | V | Q | L | L | K | D | A | G | L | T | T | L | L | G | P | V | Q | H | G | G | - | - | - | - | - | - | - | - | - | - | - | - | - | A | G | Q | P | W | D | T | A | Y | K | V | I | R |
| 119 UniRef90\_A0A3N1X255\_20\_392 | P | L | D | A | V | R | A | L | K | A | S | G | L | N | A | A | W | I | G | R | A | Y | G | G | - | - | - | - | - | - | - | - | - | - | - | - | - | E | G | A | S | W | T | S | I | L | R | V | V | R |
| 120 UniRef90\_A0A1S1LA29\_28\_404 | P | V | A | E | I | G | L | L | R | H | H | G | L | L | S | F | A | T | A | R | E | F | G | G | - | - | - | - | - | - | - | - | - | - | - | - | - | A | G | G | S | L | A | Q | A | L | Q | L | S | R |
| 121 UniRef90\_A0A081GNL4\_19\_392 | P | L | G | A | R | Q | R | I | R | Q | G | G | L | L | S | L | S | I | P | V | P | W | G | G | - | - | - | - | - | - | - | - | - | - | - | - | - | G | G | W | S | W | S | R | L | S | S | L | V | R |
| 122 UniRef90\_A0A1I6U758\_27\_419 | P | T | A | E | V | R | L | L | K | E | S | G | L | V | T | L | L | G | P | A | E | H | G | G | - | - | - | - | - | - | - | - | - | - | - | - | - | A | E | Q | H | W | T | T | A | C | R | V | V | R |
| 123 UniRef90\_A0A0F5VZP9\_7\_388 | P | R | A | E | L | D | R | L | S | A | S | G | L | L | A | V | T | V | P | A | E | H | G | G | - | - | - | - | - | - | - | - | - | - | - | - | - | A | D | V | G | P | Q | T | L | A | E | T | F | R |
| 124 UniRef90\_A0A1H4GIM8\_20\_411 | P | Y | T | E | V | Q | L | L | K | E | S | G | L | V | T | L | L | G | P | R | E | H | G | G | - | - | - | - | - | - | - | - | - | - | - | - | - | A | G | E | S | W | D | T | A | Y | K | V | I | R |
| 125 UniRef90\_A0A0M8TTT1\_8\_388 | P | R | Q | E | L | D | R | I | T | A | S | G | L | L | A | V | T | V | P | A | E | H | G | G | - | - | - | - | - | - | - | - | - | - | - | - | - | A | D | V | R | Q | E | T | L | A | E | I | F | R |
| 126 UniRef90\_A0A1Y2MDA3\_48\_437 | P | R | A | E | I | A | L | L | K | H | A | G | L | L | K | I | L | G | P | K | K | Y | G | G | - | - | - | - | - | - | - | - | - | - | - | - | - | G | E | Q | P | W | G | V | G | Y | K | A | I | R |
| 127 UniRef90\_UPI000562F0C5\_9\_388 | P | R | P | E | L | D | R | L | S | A | S | G | L | L | A | V | S | V | P | A | E | H | G | G | - | - | - | - | - | - | - | - | - | - | - | - | - | A | D | V | G | A | G | T | L | A | E | I | F | R |
| 128 UniRef90\_A0A506Y8Z1\_43\_442 | P | R | S | E | V | A | L | L | K | A | S | G | L | V | T | L | L | G | P | T | A | H | G | G | - | - | - | - | - | - | - | - | - | - | - | - | - | A | G | Q | S | W | E | T | A | Y | R | V | I | R |
| 129 UniRef90\_A0A021VVW0\_28\_409 | P | H | D | E | V | R | L | L | K | Q | A | G | L | V | T | L | L | G | P | R | A | A | G | G | - | - | - | - | - | - | - | - | - | - | - | - | - | A | G | Q | S | W | A | T | A | Y | Q | V | I | R |
| 130 UniRef90\_E3QTS2\_46\_428 | P | R | A | E | I | A | L | L | K | H | S | G | L | L | K | V | L | G | W | K | K | Y | G | G | - | - | - | - | - | - | - | - | - | - | - | - | - | G | E | Q | P | W | S | V | G | Y | K | V | I | R |
| 131 UniRef90\_A0A1D8SMV7\_7\_388 | P | R | A | E | L | D | R | L | S | A | S | G | L | L | A | V | T | V | P | A | D | H | G | G | - | - | - | - | - | - | - | - | - | - | - | - | - | A | D | V | R | A | T | T | L | A | E | V | F | R |
| 132 UniRef90\_A9BUV8\_38\_419 | P | W | Q | E | V | E | Q | F | S | Q | S | G | L | W | G | I | T | I | P | R | E | Y | G | G | - | - | - | - | - | - | - | - | - | - | - | - | - | A | G | V | S | T | A | T | L | T | R | A | I | A |
| 133 UniRef90\_A0A1N6TB45\_32\_407 | P | S | A | Q | I | A | L | L | R | Q | E | G | L | L | A | L | L | L | P | T | A | V | G | G | - | - | - | - | - | - | - | - | - | - | - | - | - | E | A | Q | P | W | S | T | A | L | R | I | T | R |
| 134 UniRef90\_A0A381IE41\_24\_406 | P | Y | D | E | V | D | W | F | S | Q | S | G | L | W | A | I | T | V | P | N | A | Y | G | G | - | - | - | - | - | - | - | - | - | - | - | - | - | A | G | V | S | H | V | T | L | T | E | V | A | K |
| 135 UniRef90\_A0A3M8TJ33\_18\_397 | P | F | D | E | V | S | R | L | R | E | A | G | L | L | S | L | L | I | P | V | E | S | G | G | - | - | - | - | - | - | - | - | - | - | - | - | - | G | G | A | D | W | P | T | A | H | A | V | I | R |
| 136 UniRef90\_A0A1H3JL41\_4\_373 | P | V | E | E | V | E | L | L | R | G | A | G | L | L | - | - | V | I | P | P | D | - | - | - | - | - | - | - | - | - | - | - | - | - | - | - | - | - | - | - | D | H | V | T | T | H | A | V | T | R |
| 137 UniRef90\_A0A507AW89\_49\_427 | P | R | A | E | V | A | L | L | K | H | S | G | L | L | K | V | L | G | L | K | K | Y | G | G | - | - | - | - | - | - | - | - | - | - | - | - | - | G | E | Q | P | W | S | V | G | Y | K | V | I | R |
| 138 UniRef90\_A0A1X1SEN2\_20\_406 | P | T | F | E | I | G | L | L | K | E | A | G | L | L | G | I | F | I | P | E | E | H | G | G | - | - | - | - | - | - | - | - | - | - | - | - | - | G | G | A | D | F | S | Q | A | A | A | V | V | G |
| 139 UniRef90\_A0A2D3UFX4\_26\_403 | P | F | D | E | V | S | R | L | R | E | A | G | L | L | T | L | L | I | P | A | E | L | G | G | - | - | - | - | - | - | - | - | - | - | - | - | - | G | G | E | D | W | P | T | A | Y | A | V | V | R |
| 140 UniRef90\_A0A089X0S9\_26\_403 | P | F | D | E | V | S | R | L | R | E | A | G | L | L | T | L | L | I | P | A | E | L | G | G | - | - | - | - | - | - | - | - | - | - | - | - | - | G | G | A | D | W | P | T | A | Y | A | A | V | R |
| 141 UniRef90\_A0A2W5T1D0\_29\_413 | P | V | D | E | V | A | L | L | K | N | S | G | L | V | T | L | L | G | P | A | E | H | G | G | - | - | - | - | - | - | - | - | - | - | - | - | - | G | G | Q | Q | W | A | T | A | Y | R | V | I | R |
| 142 UniRef90\_A0A1C4QGR9\_7\_388 | P | R | A | E | L | E | R | L | S | A | S | G | L | L | A | V | T | V | P | A | A | H | G | G | - | - | - | - | - | - | - | - | - | - | - | - | - | A | D | V | G | A | G | T | L | A | E | I | F | R |
| 143 UniRef90\_A0A101R4L6\_7\_388 | P | R | A | E | L | D | R | L | S | A | S | G | L | L | A | V | T | V | P | A | E | H | G | G | - | - | - | - | - | - | - | - | - | - | - | - | - | A | D | V | G | A | A | T | L | A | E | V | F | R |
| 144 UniRef90\_A0A4D4LHU8\_7\_388 | P | R | A | E | L | D | R | L | S | A | S | G | L | L | A | V | T | V | P | A | E | H | G | G | - | - | - | - | - | - | - | - | - | - | - | - | - | A | D | V | R | Q | E | T | L | A | E | I | F | R |
| 145 UniRef90\_L1KK35\_6\_389 | P | H | A | E | L | E | R | L | S | A | S | G | L | L | A | V | T | V | P | A | E | F | G | G | - | - | - | - | - | - | - | - | - | - | - | - | - | A | D | V | R | A | E | T | L | A | E | I | F | R |
| 146 UniRef90\_UPI0005601C6B\_13\_390 | P | R | A | E | L | E | K | L | S | A | S | G | L | L | G | I | T | V | P | A | E | Y | G | G | - | - | - | - | - | - | - | - | - | - | - | - | - | A | G | V | S | A | R | T | L | A | E | V | L | R |
| 147 UniRef90\_A0A1H9UQJ9\_6\_361 | P | A | K | E | V | E | L | L | R | H | S | G | L | L | D | V | - | - | - | - | - | - | - | - | - | - | - | - | - | - | - | - | - | - | - | - | - | - | - | A | D | W | D | L | Q | Q | R | I | G | R |
| 148 UniRef90\_A0A3D9JKY9\_12\_401 | P | L | D | E | V | A | R | L | R | E | S | G | L | L | T | L | L | A | P | A | E | H | G | G | - | - | - | - | - | - | - | - | - | - | - | - | - | G | G | A | D | W | R | T | A | Y | A | V | V | R |
| 149 UniRef90\_D9X7Q2\_26\_403 | P | F | D | E | V | S | R | L | R | E | S | G | L | L | T | L | L | V | P | A | G | L | G | G | - | - | - | - | - | - | - | - | - | - | - | - | - | G | G | G | D | W | S | T | A | Y | A | V | V | R |
| 150 UniRef90\_A0A1X1DXJ3\_20\_398 | P | V | D | L | L | N | E | F | T | R | L | G | L | G | S | I | S | V | P | R | R | F | G | G | - | - | - | - | - | - | - | - | - | - | - | - | - | G | G | L | D | Y | Q | T | L | A | E | V | F | R |

  
  

|  |  |  |  |  |  |  |  |  |  |  |  |  |  |  |  |  |  |  |  |  |  |  |  |  |  |  |  |  |  |  |  |  |  |  |  |  |  |  |  |  |  |  |  |  |  |  |  |  |  |  |
| --- | --- | --- | --- | --- | --- | --- | --- | --- | --- | --- | --- | --- | --- | --- | --- | --- | --- | --- | --- | --- | --- | --- | --- | --- | --- | --- | --- | --- | --- | --- | --- | --- | --- | --- | --- | --- | --- | --- | --- | --- | --- | --- | --- | --- | --- | --- | --- | --- | --- | --- |
| **001 Input\_pdb\_SEQRES\_A** | E | I | A | A | A | D | G | S | L | G | H | L | F | G | Y | H | L | T | N | A | P | M | I | E | L | I | G | S | Q | - | - | - | E | Q | E | E | H | L | Y | T | Q | I | A | Q | N | N | W | W | T | G |
| 002 UniRef90\_Q1W1G3\_1\_416 | E | I | A | A | A | D | G | S | L | G | H | L | L | G | Y | H | L | S | S | A | P | M | I | E | L | F | G | S | Q | - | - | - | E | Q | E | Q | R | L | Y | R | Q | I | A | Q | N | D | W | W | T | G |
| 003 UniRef90\_T0BM21\_7\_392 | E | L | A | K | V | D | G | S | L | A | H | L | Y | G | Y | H | F | L | Q | L | V | A | P | H | L | V | G | T | P | - | - | - | E | Q | K | R | Y | F | Y | T | E | S | A | K | H | N | W | F | W | G |
| 004 UniRef90\_UPI0002AC58FE\_11\_391 | E | L | A | K | A | D | S | S | I | A | H | V | F | S | Y | H | H | L | G | V | I | I | P | H | I | F | G | S | E | - | - | - | A | Q | K | E | Q | Y | Y | S | K | T | I | E | N | N | W | F | W | C |
| 005 UniRef90\_A0A3D5CW51\_20\_401 | E | F | A | K | V | D | S | S | I | A | H | V | Y | G | F | Q | H | L | M | L | A | T | V | R | L | F | S | R | P | - | - | - | D | Q | W | E | R | W | Y | E | Q | T | A | Q | S | N | W | F | W | G |
| 006 UniRef90\_A0A2V4SPP5\_16\_400 | I | L | A | K | A | D | S | A | L | A | H | L | F | G | F | H | H | L | Q | L | A | G | V | Q | L | Y | G | T | E | - | - | - | L | Q | Q | R | S | L | L | T | A | T | V | R | Q | K | L | F | W | G |
| 007 UniRef90\_E0UIV5\_8\_393 | E | F | A | K | V | D | S | S | I | A | H | I | F | S | Y | H | H | L | G | V | I | I | P | H | I | F | G | T | E | - | - | - | H | Q | K | K | Q | S | Y | L | N | T | I | K | N | N | W | F | W | C |
| 008 UniRef90\_A0A1Z4S897\_9\_391 | E | I | A | K | T | D | G | S | V | G | Q | L | L | G | Y | H | Y | F | N | S | A | I | P | R | F | F | G | T | P | - | - | - | E | Q | Y | A | E | F | S | K | A | S | A | R | H | N | W | F | W | S |
| 009 UniRef90\_UPI00045E9273\_21\_396 | I | I | S | R | A | D | S | S | L | G | H | L | F | G | F | Q | H | L | L | L | A | T | L | R | L | F | G | - | - | - | - | - | D | Q | W | P | H | Y | Y | R | E | T | V | K | N | S | W | F | W | G |
| 010 UniRef90\_A0A353Y4M9\_17\_384 | R | I | A | A | V | D | S | A | L | A | H | L | F | A | F | Q | H | L | Q | V | A | S | I | T | F | F | G | S | E | - | - | - | E | Q | Q | A | D | L | L | T | R | T | V | Q | E | R | W | F | W | G |
| 011 UniRef90\_F3KR10\_17\_384 | R | I | A | A | V | D | S | S | M | A | H | L | F | A | F | Q | H | L | Q | V | A | S | L | S | F | F | G | S | E | - | - | - | A | Q | Q | Q | E | W | L | T | R | T | V | R | E | R | W | F | W | G |
| 012 UniRef90\_A0A2D8NW56\_20\_404 | R | I | A | R | T | D | S | A | L | A | H | L | L | A | F | H | H | L | Q | V | G | S | V | L | L | Y | G | S | R | - | - | - | A | Q | Q | S | R | L | L | G | G | T | A | R | E | N | W | F | W | G |
| 013 UniRef90\_A0A2W7M9P1\_16\_386 | E | I | A | K | V | D | S | S | V | A | H | L | F | G | Y | H | F | L | C | L | A | S | V | E | L | Y | G | T | A | - | - | - | E | Q | V | S | H | F | T | K | E | T | A | E | N | D | N | F | W | G |
| 014 UniRef90\_A0A398AYR9\_12\_386 | E | M | A | K | V | D | S | S | V | A | H | L | F | G | Y | H | F | L | C | L | A | S | V | E | L | Y | G | T | P | - | - | - | E | Q | V | D | Y | F | T | K | E | T | V | E | N | N | Y | F | W | G |
| 015 UniRef90\_A0A0T6UXN9\_11\_394 | E | F | A | R | V | D | S | S | V | A | H | V | F | G | F | Q | H | L | M | L | A | T | V | R | L | F | A | S | P | - | - | - | A | Q | W | E | P | W | L | E | L | T | A | R | K | N | W | F | W | G |
| 016 UniRef90\_A0A252E884\_11\_390 | Q | L | S | T | A | D | G | S | I | G | Q | L | Y | G | N | H | L | N | L | T | T | L | A | H | V | S | G | T | P | - | - | - | E | Q | K | E | R | Y | Y | R | Q | T | A | E | K | N | L | F | W | A |
| 017 UniRef90\_A0A1Z4BZ71\_24\_403 | I | I | A | R | A | D | S | S | L | A | H | V | F | A | F | Q | F | L | M | L | A | S | I | R | L | Y | G | S | G | - | - | - | E | Q | W | Q | S | L | F | K | E | T | A | A | Q | N | L | W | W | G |
| 018 UniRef90\_A0A1Z4IGC5\_10\_387 | Q | F | A | Q | V | D | S | S | I | A | H | L | F | G | Y | H | H | L | Q | V | I | T | P | Y | L | Y | G | T | P | - | - | - | E | Q | A | Q | N | Y | Y | T | W | T | A | R | H | N | W | F | W | G |
| 019 UniRef90\_A0A1W9JD13\_18\_401 | E | I | A | K | T | D | S | S | L | A | H | L | F | G | F | Q | H | L | M | L | A | T | V | R | L | F | S | K | P | - | - | - | E | Q | W | Q | R | W | Y | E | Q | T | A | E | L | S | W | F | W | G |
| 020 UniRef90\_A0A0B6S5D2\_8\_393 | A | L | A | R | V | D | S | A | L | A | H | L | V | G | F | Q | V | L | Q | I | V | S | V | E | V | W | G | S | D | - | - | - | A | Q | R | E | R | Y | L | R | G | T | V | E | Q | G | W | W | W | G |
| 021 UniRef90\_A0A1Y3C786\_14\_396 | I | F | A | Q | T | D | S | S | I | A | H | V | F | G | F | H | H | L | L | L | A | T | I | R | L | F | G | N | A | - | - | - | Q | Q | W | Q | R | W | Y | K | V | T | A | E | K | S | W | F | W | G |
| 022 UniRef90\_A0A318KD41\_20\_397 | Q | F | A | R | V | D | S | S | V | A | H | V | F | A | F | Q | H | L | L | L | A | T | V | R | L | F | G | R | P | - | - | - | A | Q | W | Q | P | W | F | E | A | T | V | R | E | R | W | F | W | G |
| 023 UniRef90\_A0A352JDP6\_15\_389 | E | F | A | K | V | D | S | A | I | A | H | V | Y | S | Y | H | H | L | G | V | T | I | P | H | I | F | G | S | K | - | - | - | E | Q | K | E | K | F | Y | T | E | T | V | N | N | N | W | F | W | C |
| 024 UniRef90\_A0A329B538\_13\_401 | R | F | A | R | V | D | A | S | I | A | H | V | F | G | F | Q | H | L | M | L | A | T | V | R | L | F | A | G | P | - | - | - | A | Q | W | Q | P | W | F | E | H | T | A | R | R | Q | W | F | W | G |
| 025 UniRef90\_A0A2N8QAF3\_13\_394 | Q | I | A | R | V | D | S | A | L | A | H | L | L | G | F | T | C | L | Q | V | V | S | V | N | V | W | G | N | A | - | - | - | E | Q | R | A | R | Y | L | S | G | T | V | E | G | R | W | W | W | G |
| 026 UniRef90\_A0A2N7XWT8\_11\_394 | E | F | A | R | V | D | S | S | I | A | H | V | F | G | F | H | H | L | M | L | A | T | V | R | L | F | A | T | P | - | - | - | A | Q | W | Q | T | W | F | S | L | T | A | S | N | N | W | F | W | G |
| 027 UniRef90\_A0A2A4HLB2\_33\_412 | R | L | A | R | V | D | S | A | L | A | H | V | F | A | F | H | H | L | Q | V | G | S | V | L | L | Y | G | N | Q | - | - | - | H | Q | Q | Q | R | L | L | T | A | T | A | Q | E | H | L | F | W | G |
| 028 UniRef90\_A0A4R3HWL3\_41\_416 | R | F | A | E | V | D | S | S | L | A | H | L | F | A | F | Q | H | L | Q | V | A | S | I | I | L | F | A | N | P | - | - | - | V | Q | K | A | R | Y | L | G | G | T | V | S | E | G | W | F | W | G |
| 029 UniRef90\_A0A1M7NXP6\_14\_394 | I | L | A | R | A | D | S | A | L | A | H | V | F | G | F | H | H | L | Q | L | A | G | I | Q | L | Y | G | S | A | - | - | - | Q | Q | Q | R | R | L | L | T | Q | T | V | E | Q | N | L | F | W | G |
| 030 UniRef90\_A0A1B4ESK2\_22\_397 | R | I | A | R | V | D | S | A | L | A | H | L | V | G | F | Q | I | L | Q | V | V | S | V | D | V | W | G | S | A | - | - | - | A | Q | R | E | R | F | L | R | G | T | V | E | H | R | W | W | W | G |
| 031 UniRef90\_A0A1H6NKV2\_11\_394 | E | F | A | K | V | D | S | S | I | A | H | V | F | G | F | H | H | L | M | L | A | T | V | R | L | F | S | R | P | - | - | - | E | Q | W | Q | P | W | F | E | Q | T | A | R | K | N | W | F | W | G |
| 032 UniRef90\_A0A0D0KUF8\_11\_394 | E | F | A | K | V | D | S | S | I | A | H | V | F | G | F | Q | H | L | M | L | A | T | V | R | L | F | S | R | P | - | - | - | E | Q | W | Q | P | W | Y | E | Q | T | A | R | K | N | W | F | W | G |
| 033 UniRef90\_A0A0F3K7B4\_20\_401 | E | F | A | K | A | D | S | S | V | A | H | V | F | A | F | H | H | L | M | L | A | T | V | E | L | F | G | Q | P | - | - | - | E | Q | W | Q | P | W | H | E | I | T | T | R | Q | N | W | F | W | G |
| 034 UniRef90\_A0A381IMG6\_13\_394 | A | L | A | R | V | D | S | A | L | A | H | L | L | G | F | Q | C | L | Q | I | V | S | V | D | V | W | G | S | A | - | - | - | A | Q | R | E | R | Y | L | R | G | T | V | E | H | D | W | W | W | G |
| 035 UniRef90\_A0A2S9K1G1\_26\_400 | R | I | A | Q | A | D | S | S | L | A | H | L | Y | A | F | H | H | L | Q | V | A | T | I | L | L | Y | G | K | P | - | - | - | A | Q | H | E | A | L | L | R | S | T | V | E | Q | R | L | F | W | G |
| 036 UniRef90\_A0A1W6L7W5\_17\_392 | R | L | A | E | A | D | S | A | L | A | H | V | Y | G | F | H | H | L | Q | L | A | S | V | Q | L | Y | G | S | P | - | - | - | E | Q | H | A | R | Y | L | R | G | T | I | E | H | G | W | F | W | G |
| 037 UniRef90\_A0A255HJE3\_14\_398 | I | L | A | R | A | D | S | A | L | A | H | V | F | G | F | H | H | L | Q | L | A | G | L | Q | L | Y | G | S | A | - | - | - | Q | Q | Q | R | R | L | L | T | L | T | V | D | E | R | L | F | W | G |
| 038 UniRef90\_A0A0R3AD45\_11\_394 | E | F | A | K | V | D | S | S | I | A | H | V | F | G | F | H | H | L | M | L | A | T | V | R | L | F | S | R | P | - | - | - | D | Q | W | Q | P | W | F | E | Q | T | A | R | K | N | W | F | W | G |
| 039 UniRef90\_A0A1W6ZB60\_16\_389 | R | L | A | Q | A | D | S | A | L | A | H | L | F | G | F | H | H | L | Q | V | A | G | V | L | L | F | G | R | P | - | - | - | E | Q | H | E | Y | F | L | A | P | T | V | R | R | K | L | F | W | G |
| 040 UniRef90\_A0A2X1DPB7\_14\_397 | Q | V | A | R | V | D | S | A | L | A | H | L | V | G | F | Q | C | L | Q | V | V | S | V | D | V | W | G | N | A | - | - | - | A | Q | R | E | R | Y | L | R | G | T | V | D | G | R | W | W | W | G |
| 041 UniRef90\_A0A0S9M2D1\_4\_380 | I | L | A | A | A | D | S | S | L | A | H | V | F | G | F | H | H | L | M | L | A | T | V | R | L | F | G | Q | R | - | - | - | A | Q | W | E | P | W | F | A | Q | T | A | R | H | A | W | F | W | G |
| 042 UniRef90\_A0A238ZKW1\_13\_401 | E | F | A | R | V | D | S | S | I | A | H | V | F | A | F | H | H | L | M | L | A | T | L | R | M | F | G | R | P | - | - | - | D | Q | W | Q | P | W | F | R | H | T | A | R | L | N | W | F | W | G |
| 043 UniRef90\_A0A0D1P8V6\_10\_394 | E | F | A | R | V | D | S | S | I | A | H | V | F | G | F | H | H | L | M | L | A | T | V | R | L | F | G | A | P | - | - | - | S | Q | W | Q | P | W | Y | E | L | T | A | R | K | N | W | F | W | G |
| 044 UniRef90\_A0A261SP68\_11\_387 | R | L | A | R | V | D | S | A | L | A | H | V | F | G | F | H | H | L | Q | I | A | G | V | L | L | S | G | G | P | - | - | - | A | Q | H | A | H | F | L | P | P | T | V | E | G | N | L | F | W | G |
| 045 UniRef90\_A0A2N6MRB2\_11\_390 | E | L | S | K | A | D | G | S | I | G | Q | L | Y | G | N | H | L | N | L | T | A | L | G | H | V | S | G | T | S | - | - | - | E | Q | K | E | R | Y | Y | R | E | T | A | T | H | N | L | F | W | A |
| 046 UniRef90\_A0A212BVA1\_31\_405 | H | L | A | A | A | D | S | S | L | A | H | L | F | A | F | N | H | L | Q | V | A | T | I | L | L | H | G | S | T | - | - | - | E | Q | Q | R | H | W | L | S | R | A | A | H | E | R | W | F | W | G |
| 047 UniRef90\_A0A1A9KE82\_11\_394 | E | F | A | R | A | D | S | S | V | A | H | V | F | G | F | H | H | L | M | L | A | T | V | R | L | F | S | R | P | - | - | - | E | Q | W | Q | P | W | F | E | L | T | A | R | K | N | W | F | W | G |
| 048 UniRef90\_A0A4Q5PVB9\_26\_400 | R | F | A | Q | A | D | S | S | L | A | H | L | F | A | F | Q | Q | H | Q | V | A | A | V | I | L | F | G | S | P | - | - | - | A | Q | Q | E | N | Y | L | G | K | T | V | S | E | R | W | F | W | G |
| 049 UniRef90\_A0A178GPM9\_9\_392 | I | M | A | Q | V | D | S | S | L | A | H | V | Y | G | F | H | H | L | L | I | A | T | V | Q | L | F | S | Q | A | - | - | - | E | Q | Y | G | P | W | F | E | Q | T | A | Q | N | N | L | F | W | G |
| 050 UniRef90\_A0A1P9YC32\_3\_390 | R | M | A | R | V | D | S | S | L | A | H | L | F | A | F | H | H | L | Q | L | A | T | I | R | L | F | G | S | D | - | - | - | A | Q | I | R | A | W | L | G | E | T | V | T | Q | G | W | F | W | G |
| 051 UniRef90\_UPI000A1773A9\_40\_416 | R | F | A | R | V | D | S | S | V | A | H | V | F | A | F | H | H | L | M | L | A | T | T | Q | L | F | A | R | P | - | - | - | E | Q | W | E | Q | W | F | R | H | T | A | R | H | D | W | F | W | G |
| 052 UniRef90\_A0A158L201\_25\_395 | A | L | A | R | V | D | S | A | L | A | H | L | L | G | F | T | C | L | Q | V | V | S | V | T | V | W | G | N | E | - | - | - | A | Q | R | E | R | Y | L | R | G | T | V | A | H | R | W | W | W | G |
| 053 UniRef90\_A0A4Q4GT15\_13\_391 | T | I | A | Q | V | D | S | S | V | A | H | I | Y | A | F | H | H | L | L | I | A | T | V | Q | L | F | A | Q | P | - | - | - | T | Q | Y | A | K | W | F | E | D | T | A | K | N | N | L | F | W | G |
| 054 UniRef90\_A0A1C0YC62\_17\_381 | Q | L | A | T | V | D | S | S | L | A | H | L | F | G | Y | H | Y | L | C | I | A | T | I | E | L | Y | G | T | Q | - | - | - | A | H | Y | E | R | A | I | T | Q | G | T | Q | H | Q | H | F | Y | G |
| 055 UniRef90\_A0A1H7FE04\_32\_405 | V | V | A | R | V | D | S | A | L | A | H | V | L | G | F | H | H | L | Q | L | Y | G | V | S | L | Y | G | G | P | A | A | L | E | H | G | A | R | H | L | R | E | T | A | E | Q | R | L | F | W | G |
| 056 UniRef90\_A0A401MWC4\_5\_385 | E | I | A | A | G | D | G | S | I | G | Q | L | L | G | Y | H | Y | L | L | S | W | N | P | R | F | F | G | E | P | - | - | - | A | T | V | E | R | L | E | R | A | A | A | G | G | Q | W | L | W | G |
| 057 UniRef90\_A0A1G6HFW7\_16\_395 | R | F | A | Q | V | D | S | S | L | A | H | L | F | A | F | Q | H | L | H | L | A | T | V | R | L | F | G | R | P | - | - | - | R | Q | W | E | P | W | F | E | Q | T | I | A | Q | R | W | F | W | G |
| 058 UniRef90\_A0A0P9B999\_30\_403 | R | L | A | Q | A | D | S | S | L | A | H | L | F | G | F | Q | H | L | Q | V | A | S | V | L | L | F | G | S | E | - | - | - | A | Q | Q | A | R | F | L | G | E | A | V | E | K | R | W | F | W | G |
| 059 UniRef90\_A0A140K6F0\_14\_392 | T | I | S | K | A | D | G | S | S | G | Q | L | Y | A | N | H | I | T | L | V | T | L | G | E | V | M | G | R | L | - | - | - | G | Q | A | E | H | F | Y | R | R | T | C | E | N | N | L | F | W | A |
| 060 UniRef90\_A0A1P8EKI7\_5\_392 | L | I | A | Q | V | D | S | S | L | A | H | V | Y | G | F | H | H | L | L | I | A | T | V | Q | L | F | A | R | P | - | - | - | E | Q | Y | E | N | W | F | R | Q | T | A | Q | Q | Q | L | F | W | G |
| 061 UniRef90\_A0A2U3MYZ3\_7\_392 | A | I | A | R | V | D | S | S | V | A | H | V | Y | G | F | H | H | L | L | I | A | T | V | Q | L | F | A | Q | P | - | - | - | E | Q | Y | K | K | W | F | E | D | T | A | K | N | N | L | F | W | G |
| 062 UniRef90\_A0A1H2EPX5\_7\_397 | R | F | A | A | V | D | G | S | L | A | H | L | F | G | F | Q | H | L | L | L | A | T | V | A | L | F | G | S | E | - | - | - | A | Q | Q | E | S | F | F | R | R | T | V | E | G | R | W | F | W | G |
| 063 UniRef90\_UPI00041EF43F\_25\_400 | R | L | A | Q | V | D | S | A | L | A | H | V | Y | A | F | H | H | L | Q | V | A | T | V | L | L | Y | G | T | P | - | - | - | E | Q | H | E | F | F | L | R | P | T | V | G | E | R | L | F | W | G |
| 064 UniRef90\_D0IW93\_17\_384 | A | L | A | A | V | D | S | A | L | A | H | V | L | A | F | H | Q | L | Q | V | A | T | V | L | I | Y | G | S | R | - | - | - | Q | Q | Q | R | L | W | L | R | R | T | I | D | E | N | G | W | W | G |
| 065 UniRef90\_A0A395D1I6\_11\_388 | R | L | A | S | V | D | S | S | L | A | H | V | F | A | F | H | H | L | M | L | A | T | F | Q | F | F | G | S | P | - | - | - | A | Q | A | Q | E | F | L | R | R | T | A | Q | D | G | W | F | W | G |
| 066 UniRef90\_A0A1H0TK45\_27\_400 | G | F | A | R | V | D | S | A | L | A | H | L | F | A | F | H | H | L | L | L | A | T | T | Q | L | F | G | R | P | - | - | - | A | Q | W | Q | P | W | I | E | Q | T | L | A | Q | R | W | F | W | G |
| 067 UniRef90\_A0A1H8MYU5\_23\_403 | E | F | A | K | V | D | G | S | L | A | H | L | F | G | Y | H | F | S | P | I | Q | N | A | V | T | S | G | R | D | - | - | - | E | R | S | A | A | I | L | R | R | S | A | E | G | R | W | F | W | G |
| 068 UniRef90\_A0A1B1M254\_5\_403 | E | V | A | K | A | D | G | S | I | G | Q | L | L | G | Y | H | Y | L | W | F | W | A | A | R | L | V | G | T | R | - | - | - | E | Q | W | E | H | V | E | A | E | A | S | R | N | R | W | F | F | G |
| 069 UniRef90\_A0A484THM3\_28\_401 | R | L | A | Q | A | D | S | S | L | A | H | L | L | A | F | Q | H | L | Q | V | A | S | V | I | L | F | G | S | A | - | - | - | A | Q | Q | D | T | Y | L | R | A | T | V | S | Q | G | W | F | W | G |
| 070 UniRef90\_A0A0Q8Q8X6\_8\_377 | E | I | A | T | V | D | S | A | L | A | H | V | L | A | F | H | H | L | Q | I | V | T | V | L | I | Y | G | S | D | - | - | - | A | Q | K | E | R | W | L | V | D | T | M | A | R | R | C | W | W | G |
| 071 UniRef90\_A0A0Q5QDL2\_32\_410 | A | V | A | A | G | D | G | S | I | G | Q | L | L | G | Y | H | L | L | W | F | W | A | A | R | L | V | G | T | R | - | - | - | E | Q | I | E | A | V | E | T | A | A | T | Q | N | K | W | F | F | G |
| 072 UniRef90\_A0A1A5XM62\_22\_406 | T | L | A | R | A | D | S | A | L | A | H | L | L | G | F | Q | F | L | Q | I | V | S | V | D | L | W | G | S | A | - | - | - | A | Q | R | A | D | W | L | R | G | T | V | E | H | R | W | W | W | G |
| 073 UniRef90\_A0A239EI90\_21\_397 | R | V | A | A | A | D | G | S | I | G | Q | L | L | G | Y | H | L | L | W | F | W | A | A | R | L | V | G | T | P | - | - | - | E | Q | I | E | A | V | E | A | D | A | T | S | N | K | W | F | F | G |
| 074 UniRef90\_S5SWW2\_23\_403 | E | F | A | K | V | D | G | S | L | G | H | L | Y | G | Y | H | F | G | P | L | Q | N | A | A | T | A | E | A | D | - | - | - | P | R | S | R | D | I | L | R | R | F | A | A | G | R | W | F | W | G |
| 075 UniRef90\_A0A3A5JJY9\_31\_413 | R | L | A | R | A | D | S | A | V | A | H | V | L | A | F | H | H | L | Q | I | A | T | V | M | L | Y | A | T | P | - | - | - | D | Q | Q | R | R | L | L | Q | R | T | V | R | E | N | L | F | W | G |
| 076 UniRef90\_A0A315ZU85\_7\_384 | E | V | A | V | A | D | G | S | I | A | Q | L | L | G | Y | H | Y | V | N | Q | A | N | L | D | W | V | A | D | A | - | - | - | A | T | R | E | R | W | Q | R | R | S | G | A | A | Q | W | L | W | G |
| 077 UniRef90\_A0A3R9U899\_5\_381 | E | I | S | K | A | D | G | S | I | G | Q | L | L | G | Y | H | Y | L | W | N | W | A | A | R | L | V | G | T | R | - | - | - | E | Q | W | E | H | I | E | A | E | A | A | R | N | R | W | F | F | G |
| 078 UniRef90\_UPI000DD53D91\_7\_389 | E | F | A | R | T | D | G | S | L | A | H | L | F | G | Y | H | H | L | P | L | N | H | I | L | F | R | G | S | T | - | - | - | A | Q | R | Q | R | W | L | T | G | S | V | A | G | N | W | F | W | S |
| 079 UniRef90\_UPI000DE4BE09\_13\_396 | I | L | A | R | G | E | S | S | I | G | Q | L | L | G | Y | H | F | V | N | S | Q | Y | V | D | W | A | F | D | E | - | - | - | A | R | A | R | A | L | G | S | E | T | V | A | K | S | L | Y | W | G |
| 080 UniRef90\_A0A2T0R7P7\_10\_381 | E | I | A | R | T | D | G | S | I | A | Q | L | I | A | Y | H | Y | V | N | A | H | N | L | V | W | V | A | D | D | - | - | - | A | G | R | A | R | W | G | V | P | S | V | A | N | Q | W | L | W | G |
| 081 UniRef90\_A0A4R1HYS3\_27\_404 | E | V | A | A | G | D | G | S | I | G | Q | L | L | G | Y | H | Y | L | W | F | W | A | A | R | L | V | G | T | P | - | - | - | E | Q | I | E | A | V | E | A | D | A | T | R | N | Q | W | F | F | G |
| 082 UniRef90\_UPI000D1537B0\_7\_385 | E | F | A | R | V | D | G | S | I | A | H | L | Y | G | Y | H | F | L | L | M | N | M | P | V | I | A | G | Q | L | - | - | - | A | Q | A | E | A | L | Q | R | A | S | V | R | H | R | W | L | W | G |
| 083 UniRef90\_A0A1G6ZNM7\_17\_409 | A | V | A | A | G | D | G | S | I | G | Q | L | L | G | Y | H | Y | L | W | A | W | A | A | R | L | V | A | T | P | - | - | - | G | Q | I | E | A | V | E | K | Q | A | A | E | Q | E | W | F | F | G |
| 084 UniRef90\_UPI0003765980\_18\_389 | E | F | A | K | T | D | G | S | L | A | H | L | F | G | Y | H | H | L | P | L | N | L | I | L | F | R | G | S | N | - | - | - | A | Q | K | E | K | W | L | S | G | S | A | A | G | N | W | I | W | S |
| 085 UniRef90\_A0A071ICK8\_25\_398 | I | I | S | R | G | E | S | S | L | G | Q | L | L | G | Y | H | F | V | N | S | Q | Y | I | Y | W | A | L | D | D | A | - | - | A | R | A | H | A | L | G | A | E | T | V | A | K | N | Y | Y | W | G |
| 086 UniRef90\_A0A2L0WNE5\_33\_396 | I | L | A | R | G | E | S | S | I | G | Q | L | L | G | Y | H | F | V | N | S | Q | Y | I | Y | W | A | L | D | P | - | - | - | V | R | A | N | A | L | G | E | E | T | V | A | E | N | L | Y | W | G |
| 087 UniRef90\_A0A1Q8CFJ2\_9\_380 | T | V | A | A | A | D | A | S | V | G | H | L | L | G | Y | H | Y | L | Q | L | W | R | T | E | L | F | G | R | P | - | - | - | D | L | V | E | R | L | H | R | D | T | V | A | G | N | L | F | W | A |
| 088 UniRef90\_UPI000835F605\_21\_394 | I | I | S | A | A | D | G | S | I | G | Q | L | I | A | Y | H | Y | S | N | G | V | W | S | Y | I | L | G | T | P | - | - | - | E | Q | W | Q | A | T | A | R | G | V | G | E | E | G | W | F | Q | G |
| 089 UniRef90\_UPI000374AB86\_31\_398 | I | L | A | R | G | E | S | S | I | G | Q | L | L | G | Y | H | F | V | N | S | Q | Y | I | D | W | A | L | D | D | R | - | - | A | R | A | H | A | L | S | A | E | T | V | A | N | N | L | Y | W | G |
| 090 UniRef90\_A0A267RVL6\_22\_399 | I | I | S | A | A | D | G | S | I | G | Q | L | I | T | Y | H | Y | S | N | G | V | W | S | Y | I | L | G | N | R | - | - | - | E | Q | W | E | F | I | A | R | G | V | A | N | E | G | W | F | Q | S |
| 091 UniRef90\_M3VA40\_23\_395 | R | I | A | R | G | D | T | S | I | A | H | L | L | G | Y | H | Y | A | Q | Q | R | I | A | H | L | F | G | T | P | - | - | - | E | Q | A | E | A | L | S | R | R | N | A | Q | E | K | L | F | W | G |
| 092 UniRef90\_A0A395GID9\_32\_425 | E | V | A | K | G | D | G | S | I | G | M | L | L | G | Y | H | L | L | W | S | T | T | A | N | I | V | G | T | P | - | - | - | E | Q | A | E | R | I | Q | E | W | I | I | S | N | N | Y | F | V | G |
| 093 UniRef90\_UPI0008268C2F\_35\_406 | R | I | A | R | G | D | T | S | I | A | H | L | L | G | Y | H | Y | A | Q | Q | R | I | P | H | L | F | G | T | P | - | - | - | E | Q | A | D | Q | L | S | R | R | N | A | E | F | R | P | F | W | G |
| 094 UniRef90\_A0A0S1XV73\_27\_397 | E | L | A | R | S | D | G | S | L | A | H | L | Y | G | Y | Q | H | L | P | L | H | T | V | L | A | R | G | T | D | - | - | - | E | Q | Q | Q | R | W | F | S | Q | A | A | T | E | Q | W | L | W | S |
| 095 UniRef90\_A0A0F5N116\_19\_398 | R | I | A | R | G | D | T | S | I | A | H | L | V | G | Y | H | Y | A | Q | T | R | I | A | G | L | F | G | T | P | - | - | - | E | Q | A | D | A | L | S | R | R | N | A | S | E | K | L | F | W | G |
| 096 UniRef90\_A0A1N6Z7J9\_40\_406 | I | L | A | R | G | E | S | S | I | G | Q | L | L | G | Y | H | Y | V | N | S | Q | Y | P | Y | W | A | V | S | E | S | - | - | Q | K | A | H | D | L | G | V | E | T | V | Q | K | S | L | Y | W | G |
| 097 UniRef90\_A0A2V4UWQ2\_31\_411 | I | L | A | R | G | E | S | S | I | G | Q | L | L | G | Y | H | F | V | N | S | Q | Y | I | S | W | A | A | D | D | S | - | - | D | L | A | W | R | L | S | S | E | T | V | A | K | S | L | Y | W | G |
| 098 UniRef90\_A0A4P8KLT1\_5\_401 | I | L | A | R | A | D | A | S | I | A | Q | V | L | G | Y | H | Y | V | N | E | A | N | I | A | L | V | A | P | A | - | - | - | A | E | R | E | R | W | F | R | A | T | I | A | G | R | W | V | W | G |
| 099 UniRef90\_UPI0003068598\_3\_292 | - | - | - | - | - | - | - | - | - | - | - | - | - | - | - | - | - | - | - | - | - | - | - | - | - | - | G | S | A | - | - | - | A | Q | R | E | R | Y | L | R | G | T | V | E | H | D | W | W | W | G |
| 100 UniRef90\_UPI000DD585C3\_27\_403 | I | V | A | R | G | E | S | S | I | G | Q | L | L | G | Y | H | Y | V | N | S | Q | Y | V | Y | W | A | A | A | D | P | - | - | R | Q | A | W | V | F | G | A | E | T | V | E | R | Q | L | Y | W | G |
| 101 UniRef90\_A0A1E3SMN8\_25\_404 | R | I | A | R | G | D | T | S | I | A | H | L | I | G | Y | H | Y | A | Q | T | R | V | A | G | L | F | G | T | A | - | - | - | A | Q | A | D | A | Q | S | R | R | N | A | S | E | K | L | F | W | G |
| 102 UniRef90\_UPI00045EBD18\_12\_396 | V | I | G | R | A | D | A | S | I | A | Q | L | L | A | Y | H | Y | L | H | L | T | N | A | L | W | R | A | R | P | - | - | - | E | Q | A | A | A | L | A | R | A | S | V | A | G | R | W | L | W | G |
| 103 UniRef90\_A0A1Y1ZNX6\_42\_434 | K | V | A | E | A | D | G | S | I | G | M | L | L | G | Y | H | L | L | W | S | I | T | A | N | V | V | G | S | P | - | - | - | E | Q | A | D | R | Y | Q | K | L | I | I | E | N | N | Y | F | I | G |
| 104 UniRef90\_A0A0D2FPI6\_31\_423 | E | V | A | K | G | D | G | S | I | G | M | L | L | G | Y | H | L | L | W | S | T | T | A | N | V | V | G | T | D | - | - | - | E | Q | K | H | R | I | Q | K | L | I | L | D | N | D | W | F | V | G |
| 105 UniRef90\_A0A2S0KGS2\_30\_403 | R | I | A | R | G | D | T | S | V | A | H | L | L | G | Y | H | Y | A | Q | Q | R | I | P | H | L | F | G | T | R | - | - | - | E | Q | A | E | A | L | S | R | R | N | A | S | E | K | L | F | W | G |
| 106 UniRef90\_A0A1A7MGQ0\_54\_442 | E | V | A | K | G | D | G | S | I | G | M | L | L | G | Y | H | L | L | W | S | K | T | A | D | I | V | G | T | D | - | - | - | E | Q | K | E | R | F | Q | K | L | I | I | E | N | N | Y | F | V | G |
| 107 UniRef90\_UPI000413001D\_18\_392 | R | I | A | R | V | D | T | S | V | A | Q | L | L | S | Y | H | Y | L | H | L | I | N | A | L | W | R | A | G | A | - | - | - | G | Q | G | E | Q | L | S | R | A | S | V | S | G | R | W | F | W | G |
| 108 UniRef90\_G7H5P9\_11\_400 | R | I | A | R | G | D | T | S | I | A | H | L | L | G | Y | H | Y | A | Q | Q | R | I | P | H | L | F | G | T | P | - | - | - | E | Q | A | Q | E | I | S | R | R | N | A | E | E | K | T | F | W | G |
| 109 UniRef90\_A0A010YHQ5\_11\_404 | R | V | G | R | A | D | T | S | I | G | H | V | L | G | Y | H | Y | L | H | N | W | R | T | R | L | A | R | R | L | - | - | - | D | V | V | E | R | L | A | T | E | T | A | A | N | N | W | L | W | G |
| 110 UniRef90\_B0RBU7\_24\_404 | V | L | A | R | A | D | A | S | I | A | Q | V | L | A | Y | H | Y | I | N | S | G | N | L | G | F | T | A | T | G | - | - | - | D | V | R | A | D | G | Y | R | R | T | I | A | G | R | W | V | W | G |
| 111 UniRef90\_A0A167RW81\_42\_429 | E | V | A | K | A | D | G | S | L | G | M | L | L | G | Y | H | L | T | W | S | T | T | A | N | I | V | G | S | P | - | - | - | E | Q | A | D | R | F | Q | K | L | I | V | T | N | N | Y | F | V | G |
| 112 UniRef90\_A0A1X0J8A0\_26\_405 | I | I | A | A | A | D | G | S | I | G | Q | V | L | L | Y | H | Y | S | N | G | V | W | T | H | I | L | G | S | P | - | - | - | S | Q | R | E | H | I | A | R | G | V | G | E | L | G | W | F | Q | G |
| 113 UniRef90\_A0A2J6Q859\_43\_430 | E | V | A | K | V | D | G | S | I | G | M | L | L | G | Y | H | L | L | W | T | T | T | A | N | V | V | G | T | P | - | - | - | E | Q | A | D | R | W | Q | E | K | I | L | S | N | N | Y | F | V | G |
| 114 UniRef90\_UPI0003828F02\_7\_389 | V | V | A | A | A | D | A | S | V | G | H | L | L | G | Y | H | Y | L | H | V | W | R | T | S | L | F | D | V | P | - | - | - | A | T | A | A | A | L | D | R | A | T | V | E | H | G | W | F | W | A |
| 115 UniRef90\_A0A1B2HDJ6\_6\_361 | I | I | G | A | A | D | A | N | A | G | H | L | L | G | Y | H | Y | L | Q | I | W | R | S | G | L | F | D | T | A | - | - | - | - | - | - | - | - | - | - | - | F | S | R | H | D | D | Q | F | W | A |
| 116 UniRef90\_A0A2N3N0U1\_47\_427 | E | V | A | K | A | D | G | S | L | G | M | L | L | G | Y | H | L | L | W | S | L | T | A | S | I | V | G | T | T | - | - | - | E | Q | A | E | R | W | Q | E | I | I | I | T | N | N | Y | F | V | G |
| 117 UniRef90\_S5Y4X6\_32\_408 | L | I | S | A | G | D | G | S | I | G | Q | L | I | A | Y | H | Y | S | N | G | V | W | T | Y | I | L | G | T | P | - | - | - | A | Q | W | A | E | T | T | R | H | V | A | E | R | G | W | F | Q | G |
| 118 UniRef90\_A0A3R2WQN2\_17\_411 | I | V | A | S | G | D | G | S | I | G | Q | L | L | G | Y | H | Y | L | W | A | W | A | V | H | L | V | G | T | S | - | - | - | E | Q | I | D | A | V | E | A | L | Y | T | A | N | D | L | F | F | G |
| 119 UniRef90\_A0A3N1X255\_20\_392 | E | F | A | R | T | D | G | S | I | A | H | L | Y | G | Y | Q | H | L | P | L | H | I | T | A | A | R | A | T | P | - | - | - | A | Q | L | A | N | W | F | P | A | S | V | R | E | N | W | L | W | S |
| 120 UniRef90\_A0A1S1LA29\_28\_404 | I | I | A | A | A | D | G | S | I | G | Q | L | L | V | Y | H | Y | S | N | G | V | W | T | Y | I | L | G | S | P | - | - | - | A | Q | R | E | Y | I | A | R | G | V | G | E | H | G | W | F | Q | G |
| 121 UniRef90\_A0A081GNL4\_19\_392 | R | I | A | R | L | D | S | S | V | A | H | L | L | S | Y | H | Y | L | G | L | T | I | P | V | I | F | G | E | G | - | - | - | D | L | A | E | T | H | L | R | A | T | A | A | G | H | L | F | W | C |
| 122 UniRef90\_A0A1I6U758\_27\_419 | E | I | S | A | G | D | G | S | I | G | Q | L | L | G | H | H | Y | V | W | A | W | A | M | R | L | V | G | T | P | - | - | - | E | Q | I | A | E | T | D | E | L | Y | T | T | R | A | H | F | F | G |
| 123 UniRef90\_A0A0F5VZP9\_7\_388 | L | L | A | S | A | D | A | S | L | A | Q | I | P | Q | S | H | F | A | Y | V | N | V | I | R | R | Q | G | T | E | - | - | - | E | Q | R | K | F | F | F | A | E | L | L | S | G | R | R | L | - | G |
| 124 UniRef90\_A0A1H4GIM8\_20\_411 | A | V | A | R | G | D | G | S | I | G | Q | V | L | G | Y | H | Y | L | W | A | W | A | A | R | L | V | A | T | E | - | - | - | E | Q | I | A | A | V | E | E | L | Y | T | T | N | N | F | V | F | G |
| 125 UniRef90\_A0A0M8TTT1\_8\_388 | L | L | A | S | A | D | A | S | L | A | Q | I | P | Q | S | H | F | V | Y | V | N | V | I | H | R | Q | G | T | P | - | - | - | E | Q | Q | E | F | F | F | G | E | V | L | A | G | R | R | F | - | G |
| 126 UniRef90\_A0A1Y2MDA3\_48\_437 | K | V | A | E | A | D | G | S | I | G | M | L | L | G | Y | H | L | L | W | S | T | T | A | N | V | V | G | N | E | - | - | - | E | Q | A | E | R | Y | Q | K | L | I | V | E | N | N | Y | F | I | G |
| 127 UniRef90\_UPI000562F0C5\_9\_388 | L | L | A | S | A | D | G | S | L | A | Q | I | P | Q | S | H | F | V | Y | V | N | V | I | R | R | Q | G | A | P | - | - | - | E | Q | Q | K | F | F | F | A | E | L | L | A | G | R | R | L | - | G |
| 128 UniRef90\_A0A506Y8Z1\_43\_442 | E | V | A | R | G | D | G | S | I | G | Q | L | L | G | Y | H | Y | L | W | A | W | A | A | R | L | V | A | T | E | - | - | - | E | Q | I | A | A | V | E | E | L | A | T | S | S | T | A | F | Y | G |
| 129 UniRef90\_A0A021VVW0\_28\_409 | R | V | A | E | G | D | G | S | I | G | Q | L | L | G | Y | H | Y | L | W | S | Q | L | P | A | F | F | G | T | P | - | - | - | D | Q | A | R | D | I | I | G | E | A | T | R | E | A | W | F | F | G |
| 130 UniRef90\_E3QTS2\_46\_428 | T | V | A | K | G | D | G | S | I | G | M | L | L | G | Y | H | L | L | W | S | T | T | A | N | V | L | G | N | P | - | - | - | E | Q | A | D | R | F | Q | D | L | I | I | S | N | N | Y | F | V | G |
| 131 UniRef90\_A0A1D8SMV7\_7\_388 | L | L | A | S | A | D | G | S | L | A | Q | I | P | Q | S | H | F | A | Y | V | N | V | I | R | R | Q | G | T | E | - | - | - | E | Q | R | T | F | F | F | A | E | L | L | A | G | R | R | F | - | G |
| 132 UniRef90\_A9BUV8\_38\_419 | L | I | A | A | A | D | G | N | F | G | H | I | P | Q | N | H | Y | Y | S | L | E | V | L | R | V | G | G | S | P | - | - | - | A | Q | K | A | F | F | Y | D | R | V | L | R | G | E | R | L | - | G |
| 133 UniRef90\_A0A1N6TB45\_32\_407 | L | L | S | Q | V | D | S | S | V | G | H | L | Y | G | Y | H | Y | V | S | L | L | G | L | G | L | R | Q | V | P | - | - | - | A | Q | V | E | P | L | W | R | R | S | A | Q | A | Q | W | F | W | G |
| 134 UniRef90\_A0A381IE41\_24\_406 | I | V | A | A | A | D | P | S | L | G | Q | L | P | Q | N | H | F | G | L | V | D | V | I | A | L | T | G | T | D | - | - | - | E | Q | K | R | F | F | F | G | E | I | L | K | G | K | R | F | - | G |
| 135 UniRef90\_A0A3M8TJ33\_18\_397 | T | I | A | A | A | D | G | A | I | G | Q | V | L | G | C | H | Y | L | L | S | S | S | A | R | F | F | G | G | P | - | - | - | G | L | A | A | R | V | E | R | E | S | A | A | G | Q | W | C | W | G |
| 136 UniRef90\_A0A1H3JL41\_4\_373 | I | V | A | A | A | D | P | S | I | G | H | L | L | G | Y | H | Y | L | H | L | W | R | A | G | L | F | G | N | P | - | - | - | E | A | A | A | R | M | R | R | R | T | A | E | Q | G | L | F | W | A |
| 137 UniRef90\_A0A507AW89\_49\_427 | K | V | A | E | G | D | G | S | L | G | M | L | L | G | Y | H | L | L | W | S | T | T | A | N | V | V | G | T | A | - | - | - | E | Q | A | D | R | I | Q | K | L | I | I | S | N | N | Y | F | V | G |
| 138 UniRef90\_A0A1X1SEN2\_20\_406 | E | I | A | R | A | D | S | S | V | A | H | I | L | L | Y | H | Y | F | G | S | I | A | G | T | R | G | E | N | G | - | - | - | F | L | G | P | R | R | A | Q | R | I | A | R | E | N | L | F | H | G |
| 139 UniRef90\_A0A2D3UFX4\_26\_403 | E | I | A | T | A | D | G | A | I | G | Q | L | L | G | C | H | Y | F | L | S | W | S | A | R | F | F | I | E | P | - | - | - | A | L | A | A | Q | V | E | R | K | S | T | R | E | Q | W | C | W | G |
| 140 UniRef90\_A0A089X0S9\_26\_403 | E | I | A | A | A | D | G | A | V | A | Q | L | L | G | S | H | T | F | L | S | W | S | A | R | F | L | G | E | P | - | - | - | A | L | A | A | R | I | G | R | R | S | A | A | E | Q | W | C | W | G |
| 141 UniRef90\_A0A2W5T1D0\_29\_413 | I | I | A | R | G | D | G | S | I | G | Q | L | L | G | Y | H | Y | L | W | A | W | A | A | R | L | V | A | T | D | - | - | - | A | Q | I | A | A | V | E | K | L | Y | T | E | N | R | Y | L | F | G |
| 142 UniRef90\_A0A1C4QGR9\_7\_388 | L | L | A | S | A | D | P | S | L | A | Q | I | P | Q | S | H | F | V | Y | V | N | V | I | R | R | Q | G | T | P | - | - | - | E | Q | R | R | F | F | F | G | E | L | L | A | G | R | R | L | - | G |
| 143 UniRef90\_A0A101R4L6\_7\_388 | L | L | G | A | A | D | P | S | L | A | Q | I | P | Q | S | H | F | V | Y | V | N | V | I | R | R | Q | G | T | T | - | - | - | E | Q | R | T | F | F | F | A | E | V | L | A | G | R | R | F | - | G |
| 144 UniRef90\_A0A4D4LHU8\_7\_388 | L | L | A | S | A | D | A | S | L | A | Q | I | P | Q | N | H | F | V | Y | V | N | V | I | R | R | Q | G | I | E | - | - | - | E | Q | R | K | F | F | F | A | E | V | L | A | G | R | R | F | - | G |
| 145 UniRef90\_L1KK35\_6\_389 | L | L | A | A | V | D | A | S | L | A | Q | I | P | Q | S | H | F | V | Y | V | N | V | L | R | R | Q | G | T | H | - | - | - | E | Q | Q | E | F | F | L | G | E | V | L | R | G | K | R | F | - | G |
| 146 UniRef90\_UPI0005601C6B\_13\_390 | L | L | A | T | A | D | G | S | L | A | Q | I | P | Q | N | H | F | V | Y | V | N | V | L | R | R | Q | G | T | A | - | - | - | A | Q | R | R | F | F | F | A | E | V | L | A | G | R | R | F | - | G |
| 147 UniRef90\_A0A1H9UQJ9\_6\_361 | I | V | G | A | A | D | A | N | V | G | H | L | L | G | Y | H | Y | L | Q | I | W | R | S | G | L | F | D | A | P | - | - | - | - | - | - | - | - | - | - | - | F | S | A | R | P | G | Q | F | W | A |
| 148 UniRef90\_A0A3D9JKY9\_12\_401 | T | V | A | A | A | D | G | A | I | G | H | L | L | G | N | H | Y | F | L | S | F | S | A | R | F | F | A | D | P | - | - | - | T | R | T | A | R | I | E | R | E | S | T | A | A | L | W | C | W | G |
| 149 UniRef90\_D9X7Q2\_26\_403 | E | I | A | A | A | D | G | A | I | G | Q | L | L | G | C | H | Y | V | M | S | W | S | A | R | F | L | T | E | P | - | - | - | D | L | A | V | R | L | E | Q | R | S | T | A | E | Q | W | C | W | G |
| 150 UniRef90\_A0A1X1DXJ3\_20\_398 | I | I | S | A | S | D | P | S | L | G | Q | I | P | Q | N | H | F | G | L | I | Q | F | I | L | G | E | G | E | P | - | - | - | E | Q | Q | E | T | L | L | Q | A | V | V | N | G | H | R | L | - | G |

  
  

|  |  |  |  |  |  |  |  |  |  |  |  |  |  |  |  |  |  |  |  |  |  |  |  |  |  |  |  |  |  |  |  |  |  |  |  |  |  |  |  |  |  |  |  |  |  |  |  |  |  |  |
| --- | --- | --- | --- | --- | --- | --- | --- | --- | --- | --- | --- | --- | --- | --- | --- | --- | --- | --- | --- | --- | --- | --- | --- | --- | --- | --- | --- | --- | --- | --- | --- | --- | --- | --- | --- | --- | --- | --- | --- | --- | --- | --- | --- | --- | --- | --- | --- | --- | --- | --- |
| **001 Input\_pdb\_SEQRES\_A** | N | A | S | - | - | - | - | - | - | S | E | N | N | S | H | V | L | D | W | K | V | - | R | A | - | - | - | - | T | P | T | - | - | - | E | D | G | G | Y | V | L | N | G | T | K | H | F | C | S | G |
| 002 UniRef90\_Q1W1G3\_1\_416 | N | A | S | - | - | - | - | - | - | S | E | N | N | S | H | V | L | D | W | K | V | - | S | A | - | - | - | - | S | P | T | - | - | - | E | D | G | G | Y | L | L | N | G | T | K | H | F | C | S | G |
| 003 UniRef90\_T0BM21\_7\_392 | N | A | F | - | - | - | - | - | - | N | P | - | - | - | - | - | L | D | Q | R | L | - | R | G | - | - | - | - | N | R | E | - | - | - | - | D | G | R | V | V | L | N | G | R | K | S | F | C | T | G |
| 004 UniRef90\_UPI0002AC58FE\_11\_391 | N | A | L | - | - | - | - | - | - | N | P | - | - | - | - | - | L | D | R | R | T | - | T | L | - | - | - | - | T | P | D | - | - | - | - | G | N | N | F | R | L | N | G | V | K | S | F | C | S | G |
| 005 UniRef90\_A0A3D5CW51\_20\_401 | N | A | L | - | - | - | - | - | - | N | P | - | - | - | - | - | L | D | E | R | T | - | L | C | - | - | - | - | K | S | Y | - | - | - | - | D | H | W | H | E | F | S | G | K | K | S | F | C | S | G |
| 006 UniRef90\_A0A2V4SPP5\_16\_400 | N | A | L | - | - | - | - | - | - | N | P | - | - | - | - | - | L | D | K | R | T | - | R | A | - | - | - | - | V | A | S | - | - | - | - | P | A | G | Y | I | L | D | G | V | K | S | F | A | S | G |
| 007 UniRef90\_E0UIV5\_8\_393 | N | A | L | - | - | - | - | - | - | N | P | - | - | - | - | - | L | D | R | R | T | - | T | L | - | - | - | - | T | P | E | - | - | - | - | K | N | A | F | R | L | K | G | N | K | S | F | C | S | G |
| 008 UniRef90\_A0A1Z4S897\_9\_391 | D | A | A | - | - | - | - | - | - | N | P | - | - | - | - | - | R | D | P | D | S | - | I | L | - | - | - | - | T | P | D | - | - | - | - | G | E | N | F | R | L | N | G | L | K | N | F | A | T | G |
| 009 UniRef90\_UPI00045E9273\_21\_396 | N | T | L | - | - | - | - | - | - | N | P | - | - | - | - | - | L | D | T | R | A | - | K | I | - | - | - | - | S | A | D | - | - | - | - | G | Q | D | W | L | V | H | G | T | K | S | F | C | S | G |
| 010 UniRef90\_A0A353Y4M9\_17\_384 | N | A | L | - | - | - | - | - | - | N | P | - | - | - | - | - | A | D | R | R | T | - | T | A | - | - | - | - | A | E | F | - | - | - | - | D | E | G | L | V | L | N | G | V | K | S | F | C | S | G |
| 011 UniRef90\_F3KR10\_17\_384 | N | A | N | - | - | - | - | - | - | N | P | - | - | - | - | - | A | D | K | R | V | - | T | A | - | - | - | - | V | E | F | - | - | - | - | D | E | G | L | V | L | N | G | V | K | S | F | C | S | G |
| 012 UniRef90\_A0A2D8NW56\_20\_404 | N | T | L | - | - | - | - | - | - | N | P | - | - | - | - | - | L | D | R | R | T | - | L | A | - | - | - | - | S | D | R | - | - | - | A | A | G | G | F | L | F | H | G | D | K | G | F | C | S | G |
| 013 UniRef90\_A0A2W7M9P1\_16\_386 | N | A | F | - | - | - | - | - | - | N | P | - | - | - | - | - | L | D | I | H | V | - | K | A | - | - | - | - | K | K | T | - | - | - | - | A | N | G | W | L | V | Q | G | K | K | S | F | C | S | G |
| 014 UniRef90\_A0A398AYR9\_12\_386 | N | A | F | - | - | - | - | - | - | N | P | - | - | - | - | - | L | D | T | H | V | - | T | A | - | - | - | - | T | K | G | - | - | - | - | D | N | G | W | V | I | N | G | K | K | S | F | C | S | G |
| 015 UniRef90\_A0A0T6UXN9\_11\_394 | N | A | L | - | - | - | - | - | - | N | P | - | - | - | - | - | L | D | T | R | T | - | V | M | - | - | - | - | K | K | F | - | - | - | - | D | G | Y | Y | E | F | S | G | K | K | S | F | C | S | G |
| 016 UniRef90\_A0A252E884\_11\_390 | N | A | I | - | - | - | - | - | - | N | T | - | - | - | - | - | R | D | T | R | L | - | K | I | - | - | - | - | T | P | D | - | - | - | - | G | E | N | F | R | V | D | G | A | K | S | F | G | T | G |
| 017 UniRef90\_A0A1Z4BZ71\_24\_403 | N | A | L | - | - | - | - | - | - | N | P | - | - | - | - | - | L | D | T | R | T | - | I | A | - | - | - | - | S | E | A | - | - | - | - | A | E | H | L | L | F | N | G | F | K | S | F | C | S | G |
| 018 UniRef90\_A0A1Z4IGC5\_10\_387 | N | A | L | - | - | - | - | - | - | N | P | - | - | - | - | - | L | D | K | R | L | - | T | L | - | - | - | - | S | S | D | - | - | - | - | G | K | N | Y | R | L | N | G | L | K | S | F | C | S | G |
| 019 UniRef90\_A0A1W9JD13\_18\_401 | N | A | L | - | - | - | - | - | - | N | P | - | - | - | - | - | L | D | D | R | T | - | I | S | - | - | - | - | K | S | F | - | - | - | - | N | G | W | H | E | F | S | G | K | K | S | F | C | S | G |
| 020 UniRef90\_A0A0B6S5D2\_8\_393 | N | A | V | - | - | - | - | - | - | N | S | - | - | - | - | - | L | D | T | R | L | - | V | A | - | - | - | - | R | Q | E | - | - | - | A | D | G | G | Y | R | L | D | G | I | K | S | F | C | S | G |
| 021 UniRef90\_A0A1Y3C786\_14\_396 | N | A | L | - | - | - | - | - | - | N | P | - | - | - | - | - | L | D | K | R | T | - | I | V | - | - | - | - | K | H | Q | - | - | - | - | N | G | W | Y | E | F | S | G | K | K | S | F | C | S | G |
| 022 UniRef90\_A0A318KD41\_20\_397 | N | A | L | - | - | - | - | - | - | N | P | - | - | - | - | - | L | D | G | R | T | - | E | A | - | - | - | - | Q | P | D | - | - | - | - | G | D | G | Y | V | F | H | G | S | K | S | F | C | S | G |
| 023 UniRef90\_A0A352JDP6\_15\_389 | N | A | L | - | - | - | - | - | - | N | P | - | - | - | - | - | L | D | R | R | S | - | T | L | - | - | - | - | T | R | D | - | - | - | - | G | D | I | W | I | L | N | G | I | K | S | F | C | S | G |
| 024 UniRef90\_A0A329B538\_13\_401 | N | A | L | - | - | - | - | - | - | N | P | - | - | - | - | - | L | D | P | R | T | - | Q | A | - | - | - | - | R | P | R | - | - | - | - | P | G | H | Y | L | F | A | G | Q | K | R | F | C | S | G |
| 025 UniRef90\_A0A2N8QAF3\_13\_394 | N | A | V | - | - | - | - | - | - | N | P | - | - | - | - | - | L | D | T | R | L | - | V | A | - | - | - | - | G | A | T | - | - | - | R | D | G | G | Y | R | L | D | G | Q | K | G | F | C | S | G |
| 026 UniRef90\_A0A2N7XWT8\_11\_394 | N | T | L | - | - | - | - | - | - | N | P | - | - | - | - | - | L | D | T | R | T | - | V | V | - | - | - | - | K | R | H | - | - | - | - | T | G | W | R | E | F | S | G | Q | K | N | F | C | S | G |
| 027 UniRef90\_A0A2A4HLB2\_33\_412 | N | T | L | - | - | - | - | - | - | N | P | - | - | - | - | - | L | D | K | R | T | - | V | A | - | - | - | - | T | E | R | - | - | - | G | N | G | G | F | I | L | Q | G | K | K | G | F | C | S | G |
| 028 UniRef90\_A0A4R3HWL3\_41\_416 | N | G | T | - | - | - | - | - | - | N | S | - | - | - | - | - | L | G | N | Q | V | - | R | L | V | W | - | - | R | E | E | - | - | - | - | G | G | Y | Y | E | L | S | G | T | K | T | F | C | S | G |
| 029 UniRef90\_A0A1M7NXP6\_14\_394 | N | A | L | - | - | - | - | - | - | N | P | - | - | - | - | - | L | D | K | R | T | - | T | A | - | - | - | - | T | D | A | - | - | - | - | D | Y | G | F | Q | L | D | G | I | K | S | F | S | S | G |
| 030 UniRef90\_A0A1B4ESK2\_22\_397 | N | A | V | - | - | - | - | - | - | N | P | - | - | - | - | - | L | D | T | R | L | - | V | A | - | - | - | - | T | A | T | - | - | - | P | D | G | G | Y | R | L | D | G | V | K | G | F | C | S | G |
| 031 UniRef90\_A0A1H6NKV2\_11\_394 | N | A | L | - | - | - | - | - | - | N | P | - | - | - | - | - | L | D | T | R | T | - | V | V | - | - | - | - | R | K | L | - | - | - | - | D | G | W | R | E | F | S | G | K | K | S | F | C | S | G |
| 032 UniRef90\_A0A0D0KUF8\_11\_394 | N | A | L | - | - | - | - | - | - | N | P | - | - | - | - | - | L | D | T | R | T | - | I | V | - | - | - | - | K | K | F | - | - | - | - | D | G | W | R | E | F | S | G | K | K | S | F | C | S | G |
| 033 UniRef90\_A0A0F3K7B4\_20\_401 | N | A | L | - | - | - | - | - | - | N | P | - | - | - | - | - | L | D | E | R | T | - | V | S | - | - | - | - | R | R | F | - | - | - | - | D | G | W | H | E | F | S | G | Q | K | S | F | C | S | G |
| 034 UniRef90\_A0A381IMG6\_13\_394 | N | A | V | - | - | - | - | - | - | N | P | - | - | - | - | - | L | D | A | R | L | - | V | A | - | - | - | - | H | A | T | - | - | - | G | D | G | G | Y | R | L | D | G | V | K | G | F | C | S | G |
| 035 UniRef90\_A0A2S9K1G1\_26\_400 | N | A | L | - | - | - | - | - | - | N | P | - | - | - | - | - | N | D | K | R | A | - | L | A | - | - | - | - | T | E | D | - | - | - | - | G | T | G | W | R | I | S | G | P | K | S | Y | C | S | G |
| 036 UniRef90\_A0A1W6L7W5\_17\_392 | N | A | L | - | - | - | - | - | - | N | P | - | - | - | - | - | L | D | K | R | L | - | V | A | - | - | - | - | T | E | V | - | - | - | - | D | G | G | Y | R | L | D | G | L | K | G | F | A | S | G |
| 037 UniRef90\_A0A255HJE3\_14\_398 | N | A | L | - | - | - | - | - | - | N | P | - | - | - | - | - | L | D | K | R | V | - | T | A | - | - | - | - | T | D | S | - | - | - | - | G | D | G | F | V | L | D | G | I | K | S | F | S | S | G |
| 038 UniRef90\_A0A0R3AD45\_11\_394 | N | A | L | - | - | - | - | - | - | N | P | - | - | - | - | - | L | D | T | R | T | - | Q | V | - | - | - | - | K | D | F | - | - | - | - | G | G | W | R | E | F | S | G | K | K | S | F | C | S | G |
| 039 UniRef90\_A0A1W6ZB60\_16\_389 | N | A | L | - | - | - | - | - | - | N | P | - | - | - | - | - | L | D | R | R | V | - | L | A | - | - | - | - | A | N | E | - | - | - | - | G | E | G | F | R | L | D | G | V | K | S | F | S | S | G |
| 040 UniRef90\_A0A2X1DPB7\_14\_397 | N | A | V | - | - | - | - | - | - | N | P | - | - | - | - | - | L | D | T | R | L | - | V | A | - | - | - | - | T | A | T | - | - | - | P | D | G | G | Y | R | L | D | G | V | K | G | F | C | S | G |
| 041 UniRef90\_A0A0S9M2D1\_4\_380 | N | A | L | - | - | - | - | - | - | N | P | - | - | - | - | - | L | D | A | R | T | - | V | A | - | - | - | - | T | P | Q | - | - | - | - | D | G | W | H | A | F | T | G | Q | K | S | F | C | S | G |
| 042 UniRef90\_A0A238ZKW1\_13\_401 | N | A | L | - | - | - | - | - | - | N | P | - | - | - | - | - | L | D | A | R | T | - | T | S | - | - | - | - | R | Q | V | - | - | - | - | K | H | W | H | E | F | S | G | K | K | S | F | C | S | G |
| 043 UniRef90\_A0A0D1P8V6\_10\_394 | N | A | L | - | - | - | - | - | - | N | P | - | - | - | - | - | L | D | T | R | T | - | I | V | - | - | - | - | K | H | H | - | - | - | - | G | S | W | R | E | F | S | G | K | K | S | F | C | S | G |
| 044 UniRef90\_A0A261SP68\_11\_387 | N | A | L | - | - | - | - | - | - | N | P | - | - | - | - | - | L | D | R | R | A | - | I | A | - | - | - | - | A | E | E | - | - | - | - | P | G | G | F | R | L | D | G | V | K | S | F | S | S | G |
| 045 UniRef90\_A0A2N6MRB2\_11\_390 | N | A | I | - | - | - | - | - | - | N | T | - | - | - | - | - | R | D | S | R | L | - | K | I | - | - | - | - | T | P | D | - | - | - | - | G | E | H | F | R | V | N | G | I | K | S | F | G | T | G |
| 046 UniRef90\_A0A212BVA1\_31\_405 | N | A | T | - | - | - | - | - | - | N | G | - | - | - | - | - | R | D | L | G | L | - | Q | L | - | - | - | - | Q | K | R | - | - | - | - | E | E | H | F | E | L | N | G | R | K | S | F | C | S | G |
| 047 UniRef90\_A0A1A9KE82\_11\_394 | N | A | L | - | - | - | - | - | - | N | P | - | - | - | - | - | L | D | T | R | T | - | Q | V | - | - | - | - | K | W | F | - | - | - | - | D | G | W | C | E | F | S | G | K | K | S | F | C | S | G |
| 048 UniRef90\_A0A4Q5PVB9\_26\_400 | N | A | V | - | - | - | - | - | - | N | A | - | - | - | - | - | R | D | T | R | L | - | A | A | - | - | - | - | T | R | T | - | - | - | - | D | G | G | W | R | L | D | G | V | K | A | F | C | S | G |
| 049 UniRef90\_A0A178GPM9\_9\_392 | N | T | L | - | - | - | - | - | - | N | P | - | - | - | - | - | L | D | R | R | T | - | T | A | - | - | - | - | T | K | V | - | - | - | S | E | N | E | Y | I | F | H | G | D | K | S | F | C | S | G |
| 050 UniRef90\_A0A1P9YC32\_3\_390 | N | A | L | - | - | - | - | - | - | N | P | - | - | - | - | - | L | D | K | S | T | - | R | A | - | - | - | - | L | P | D | - | - | - | G | A | G | G | Y | V | F | D | G | R | K | S | F | C | S | G |
| 051 UniRef90\_UPI000A1773A9\_40\_416 | N | A | L | - | - | - | - | - | - | N | P | - | - | - | - | - | L | D | R | R | T | - | V | C | - | - | - | - | R | R | L | - | - | - | - | D | G | W | S | E | F | S | G | R | K | S | F | C | S | G |
| 052 UniRef90\_A0A158L201\_25\_395 | N | A | V | - | - | - | - | - | - | N | P | - | - | - | - | - | L | D | T | R | L | - | I | A | - | - | - | - | H | A | T | - | - | - | G | D | G | G | Y | R | L | S | G | Q | K | G | F | C | S | G |
| 053 UniRef90\_A0A4Q4GT15\_13\_391 | N | T | L | - | - | - | - | - | - | N | P | - | - | - | - | - | L | D | R | R | T | - | K | A | - | - | - | - | T | Q | V | - | - | - | S | E | Q | E | F | I | F | H | G | D | K | S | F | C | S | G |
| 054 UniRef90\_A0A1C0YC62\_17\_381 | N | A | F | - | - | - | - | - | - | N | P | - | - | - | - | - | L | D | T | H | V | - | T | A | - | - | - | - | T | K | Q | - | - | - | A | D | G | Q | W | L | L | Q | G | T | K | Y | F | C | S | G |
| 055 UniRef90\_A0A1H7FE04\_32\_405 | N | A | L | - | - | - | - | - | - | N | P | - | - | - | - | - | A | D | T | R | L | - | R | A | - | - | - | - | T | L | A | - | - | - | - | P | G | G | W | R | L | D | G | A | K | S | F | C | S | G |
| 056 UniRef90\_A0A401MWC4\_5\_385 | G | A | F | - | - | - | - | - | - | N | P | - | - | - | - | - | R | D | P | D | V | - | T | L | - | - | - | - | T | P | D | - | - | - | - | G | D | G | F | R | L | D | G | R | K | S | F | A | T | G |
| 057 UniRef90\_A0A1G6HFW7\_16\_395 | N | A | L | - | - | - | - | - | - | N | P | - | - | - | - | - | L | D | Q | G | T | - | R | A | - | - | - | - | V | P | D | - | - | - | R | A | G | G | W | Y | F | V | G | R | K | S | F | C | S | G |
| 058 UniRef90\_A0A0P9B999\_30\_403 | N | A | V | - | - | - | - | - | - | N | A | - | - | - | - | - | R | D | T | R | L | - | Q | A | - | - | - | - | A | R | T | - | - | - | - | D | D | G | I | E | I | D | G | V | K | G | F | C | S | G |
| 059 UniRef90\_A0A140K6F0\_14\_392 | N | A | V | - | - | - | - | - | - | N | A | - | - | - | - | - | R | D | A | R | L | - | K | I | - | - | - | - | T | P | D | - | - | - | - | G | N | N | F | L | V | N | G | I | K | S | F | G | T | G |
| 060 UniRef90\_A0A1P8EKI7\_5\_392 | N | A | L | - | - | - | - | - | - | N | P | - | - | - | - | - | L | D | R | R | T | - | Q | V | - | - | - | - | Q | P | L | - | - | - | N | D | D | T | F | I | F | Q | G | E | K | S | F | C | S | G |
| 061 UniRef90\_A0A2U3MYZ3\_7\_392 | N | T | L | - | - | - | - | - | - | N | P | - | - | - | - | - | L | D | R | R | T | - | T | A | - | - | - | - | Q | Q | I | - | - | - | S | E | N | E | F | I | F | H | G | D | K | S | F | C | S | G |
| 062 UniRef90\_A0A1H2EPX5\_7\_397 | N | A | L | - | - | - | - | - | - | N | P | - | - | - | - | - | L | D | P | G | T | - | L | A | - | - | - | - | S | A | T | - | - | - | - | E | G | G | R | R | I | N | G | R | K | S | F | S | S | G |
| 063 UniRef90\_UPI00041EF43F\_25\_400 | N | A | L | - | - | - | - | - | - | N | P | - | - | - | - | - | N | D | K | R | T | - | L | A | - | - | - | - | H | D | D | - | - | - | - | G | D | G | F | I | V | H | G | P | K | S | Y | C | S | G |
| 064 UniRef90\_D0IW93\_17\_384 | N | A | L | - | - | - | - | - | - | N | P | - | - | - | - | - | R | D | T | R | L | - | Q | A | L | P | R | S | S | E | L | - | - | - | - | A | G | G | Y | V | L | D | G | L | K | G | F | C | S | G |
| 065 UniRef90\_A0A395D1I6\_11\_388 | N | A | L | - | - | - | - | - | - | N | P | - | - | - | - | - | R | D | P | R | T | - | Q | L | - | - | - | - | Q | A | V | - | - | - | - | E | G | G | Y | R | L | D | G | H | K | S | F | C | S | G |
| 066 UniRef90\_A0A1H0TK45\_27\_400 | N | A | L | - | - | - | - | - | - | N | P | - | - | - | - | - | L | D | K | S | T | - | H | A | - | - | - | - | L | Q | Q | - | - | - | A | D | G | S | Y | T | F | T | G | R | K | S | F | C | S | G |
| 067 UniRef90\_A0A1H8MYU5\_23\_403 | N | T | T | - | - | - | - | - | - | N | S | - | - | - | - | - | F | S | K | S | L | - | F | G | - | - | - | - | R | K | V | - | - | - | - | E | G | G | V | L | L | N | G | D | R | P | F | A | S | G |
| 068 UniRef90\_A0A1B1M254\_5\_403 | G | A | V | - | - | - | - | - | - | N | P | - | - | - | - | - | R | D | K | D | V | - | I | V | - | - | - | - | T | E | D | - | - | - | - | G | D | D | L | V | F | T | G | R | K | S | F | S | T | G |
| 069 UniRef90\_A0A484THM3\_28\_401 | N | A | V | - | - | - | - | - | - | N | A | - | - | - | - | - | R | D | T | R | L | - | E | V | - | - | - | - | T | R | T | - | - | - | - | A | D | G | Y | R | L | D | G | I | K | G | F | C | S | G |
| 070 UniRef90\_A0A0Q8Q8X6\_8\_377 | N | A | M | - | - | - | - | - | - | N | P | - | - | - | - | - | L | D | L | R | L | - | T | A | - | - | - | - | E | D | R | - | - | - | - | G | D | G | L | V | L | N | G | Q | K | G | F | C | S | G |
| 071 UniRef90\_A0A0Q5QDL2\_32\_410 | G | A | V | - | - | - | - | - | - | N | P | - | - | - | - | - | R | D | S | D | V | - | T | I | - | - | - | - | T | D | N | - | - | - | - | G | D | T | I | S | Y | T | G | R | K | S | F | S | T | G |
| 072 UniRef90\_A0A1A5XM62\_22\_406 | N | A | V | - | - | - | - | - | - | N | P | - | - | - | - | - | L | D | T | R | L | - | V | A | - | - | - | - | Q | A | D | - | - | - | G | E | G | G | W | R | L | H | G | K | K | G | F | C | S | G |
| 073 UniRef90\_A0A239EI90\_21\_397 | G | A | V | - | - | - | - | - | - | N | P | - | - | - | - | - | R | D | S | D | V | - | V | V | - | - | - | - | T | D | E | - | - | - | - | G | D | T | I | V | F | E | G | A | K | T | F | S | T | G |
| 074 UniRef90\_S5SWW2\_23\_403 | N | T | T | - | - | - | - | - | - | N | S | - | - | - | - | - | F | S | N | S | L | - | F | G | - | - | - | - | K | R | D | - | - | - | - | A | K | G | V | V | L | N | G | V | R | P | F | A | S | G |
| 075 UniRef90\_A0A3A5JJY9\_31\_413 | N | A | L | - | - | - | - | - | - | N | P | - | - | - | - | - | R | D | R | R | C | - | R | A | - | - | - | - | Y | R | H | - | - | - | P | S | G | G | Y | V | F | N | G | D | K | S | F | C | S | G |
| 076 UniRef90\_A0A315ZU85\_7\_384 | D | A | V | - | - | - | - | - | - | N | P | - | - | - | - | - | V | D | P | D | L | - | T | L | - | - | - | - | T | P | A | - | - | - | G | Q | G | G | Y | R | L | N | G | R | K | S | F | A | T | G |
| 077 UniRef90\_A0A3R9U899\_5\_381 | G | A | V | - | - | - | - | - | - | N | P | - | - | - | - | - | R | D | K | D | V | - | V | V | - | - | - | - | T | E | D | - | - | - | - | G | D | D | L | V | F | T | G | H | K | T | F | S | T | G |
| 078 UniRef90\_UPI000DD53D91\_7\_389 | N | S | G | - | - | - | - | - | - | N | A | - | - | - | - | - | L | S | K | T | S | - | S | G | - | - | - | - | E | R | S | - | - | - | - | A | T | G | W | I | I | N | G | S | R | P | F | S | S | G |
| 079 UniRef90\_UPI000DE4BE09\_13\_396 | A | A | V | - | - | - | - | - | - | N | P | - | - | - | - | - | R | D | P | G | L | - | V | L | - | - | - | - | S | R | R | - | - | - | - | G | N | G | Y | V | L | N | G | R | K | S | F | S | T | A |
| 080 UniRef90\_A0A2T0R7P7\_10\_381 | D | S | V | - | - | - | - | - | - | N | P | - | - | - | - | - | V | D | P | D | L | - | R | L | - | - | - | - | A | R | D | - | - | - | - | G | E | D | W | V | L | S | G | T | K | N | F | S | T | G |
| 081 UniRef90\_A0A4R1HYS3\_27\_404 | G | A | V | - | - | - | - | - | - | N | P | - | - | - | - | - | R | D | D | D | V | - | T | I | - | - | - | - | R | D | T | - | - | - | - | G | D | E | I | V | Y | N | G | R | K | S | F | S | T | G |
| 082 UniRef90\_UPI000D1537B0\_7\_385 | N | A | V | - | - | - | - | - | - | S | S | - | - | - | - | - | R | D | P | T | L | - | K | G | - | - | - | - | R | E | S | - | - | - | - | G | T | G | H | V | L | D | G | F | R | P | F | A | T | G |
| 083 UniRef90\_A0A1G6ZNM7\_17\_409 | G | A | V | - | - | - | - | - | - | N | P | - | - | - | - | - | R | D | D | D | L | - | V | I | - | - | - | - | R | E | E | - | - | - | - | D | G | E | L | V | F | N | G | R | K | T | F | S | T | G |
| 084 UniRef90\_UPI0003765980\_18\_389 | N | S | G | - | - | - | - | - | - | N | A | - | - | - | - | - | M | S | K | T | S | - | T | G | - | - | - | - | E | R | I | - | - | - | - | K | G | G | W | T | I | N | G | S | R | P | F | S | S | G |
| 085 UniRef90\_A0A071ICK8\_25\_398 | A | A | V | - | - | - | - | - | - | N | P | - | - | - | - | - | R | D | P | G | L | - | E | L | - | - | - | - | T | R | R | - | - | - | - | G | S | A | Y | V | L | N | G | R | K | S | F | S | T | A |
| 086 UniRef90\_A0A2L0WNE5\_33\_396 | A | A | V | - | - | - | - | - | - | N | P | - | - | - | - | - | R | D | P | G | L | - | V | L | - | - | - | - | T | R | R | - | - | - | - | G | N | G | Y | V | L | N | G | R | K | T | F | S | T | A |
| 087 UniRef90\_A0A1Q8CFJ2\_9\_380 | G | V | S | - | - | - | - | - | - | N | P | - | - | - | - | - | L | D | A | A | L | - | E | L | - | - | - | - | T | P | V | - | - | - | - | E | G | G | F | R | V | E | G | R | K | S | F | A | T | G |
| 088 UniRef90\_UPI000835F605\_21\_394 | G | V | S | - | - | - | - | - | - | N | P | - | - | - | - | - | R | D | P | K | A | - | E | L | - | - | - | - | E | K | T | - | - | - | - | E | K | G | Y | L | I | S | G | R | R | T | F | A | T | G |
| 089 UniRef90\_UPI000374AB86\_31\_398 | A | A | V | - | - | - | - | - | - | N | P | - | - | - | - | - | R | D | P | G | L | - | M | L | - | - | - | - | T | R | R | - | - | - | - | G | N | G | Y | V | L | N | G | K | K | S | F | S | T | A |
| 090 UniRef90\_A0A267RVL6\_22\_399 | S | I | S | - | - | - | - | - | - | N | P | - | - | - | - | - | R | D | P | R | L | - | K | L | - | - | - | - | E | W | D | - | - | - | - | G | N | D | L | Y | V | T | G | R | R | T | F | A | T | G |
| 091 UniRef90\_M3VA40\_23\_395 | G | V | Q | - | - | - | - | - | - | N | P | - | - | - | - | - | R | G | G | S | G | L | V | L | - | - | - | - | T | R | D | - | - | - | - | E | D | G | F | R | L | N | G | R | R | T | F | A | S | G |
| 092 UniRef90\_A0A395GID9\_32\_425 | G | A | V | - | - | - | - | - | - | N | P | - | - | - | - | - | R | D | S | D | L | - | K | I | - | - | - | - | T | S | D | - | - | - | - | G | E | D | I | V | F | N | G | A | K | F | F | N | T | G |
| 093 UniRef90\_UPI0008268C2F\_35\_406 | G | V | Q | - | - | - | - | - | - | N | P | - | - | - | - | - | R | G | A | A | G | L | T | L | - | - | - | - | T | R | D | - | - | - | - | G | A | G | F | R | L | N | G | A | R | T | F | A | S | G |
| 094 UniRef90\_A0A0S1XV73\_27\_397 | N | S | G | - | - | - | - | - | - | N | A | - | - | - | - | - | M | S | K | T | S | - | A | A | - | - | - | - | E | R | V | - | - | - | - | P | G | G | W | I | V | D | G | F | R | P | F | S | S | G |
| 095 UniRef90\_A0A0F5N116\_19\_398 | G | I | Q | - | - | - | - | - | - | N | P | - | - | - | - | - | R | G | G | S | D | L | V | L | - | - | - | - | T | R | D | - | - | - | - | G | D | G | F | R | L | N | G | S | R | T | F | A | S | G |
| 096 UniRef90\_A0A1N6Z7J9\_40\_406 | A | A | V | - | - | - | - | - | - | N | P | - | - | - | - | - | R | D | P | G | L | - | T | L | - | - | - | - | T | K | R | - | - | - | - | G | D | Q | Y | L | L | N | G | R | K | T | F | S | T | G |
| 097 UniRef90\_A0A2V4UWQ2\_31\_411 | A | A | V | - | - | - | - | - | - | N | P | - | - | - | - | - | R | D | P | G | L | - | V | L | - | - | - | - | T | R | Q | - | - | - | - | D | D | H | Y | V | L | N | G | R | K | T | F | S | T | G |
| 098 UniRef90\_A0A4P8KLT1\_5\_401 | D | S | V | - | - | - | - | - | - | N | P | - | - | - | - | - | V | D | P | N | L | - | T | L | - | - | - | - | T | V | D | - | - | - | - | G | D | G | Y | R | L | N | G | F | K | R | Y | S | T | G |
| 099 UniRef90\_UPI0003068598\_3\_292 | N | A | V | - | - | - | - | - | - | N | P | - | - | - | - | - | L | D | T | R | L | - | V | A | - | - | - | - | R | A | T | - | - | - | G | D | G | G | Y | R | L | D | G | V | K | G | F | C | S | G |
| 100 UniRef90\_UPI000DD585C3\_27\_403 | A | A | V | - | - | - | - | - | - | N | P | - | - | - | - | - | R | D | P | G | L | - | V | L | - | - | - | - | T | R | R | - | - | - | - | G | G | H | Y | V | L | N | G | R | K | T | F | S | T | G |
| 101 UniRef90\_A0A1E3SMN8\_25\_404 | G | I | Q | - | - | - | - | - | - | N | P | - | - | - | - | - | R | G | G | S | A | L | V | L | - | - | - | - | T | R | D | - | - | - | - | G | D | G | F | R | L | N | G | Y | R | S | F | A | S | G |
| 102 UniRef90\_UPI00045EBD18\_12\_396 | G | A | S | - | - | - | - | - | - | N | P | - | - | - | - | - | R | D | P | E | S | - | R | L | - | - | - | - | A | P | V | - | - | - | - | E | D | F | Y | R | L | T | G | R | K | T | F | A | S | N |
| 103 UniRef90\_A0A1Y1ZNX6\_42\_434 | G | V | V | - | - | - | - | - | - | N | P | - | - | - | - | - | R | D | N | D | L | - | K | I | - | - | - | - | T | S | E | - | - | - | - | G | D | N | I | V | F | N | G | F | K | H | F | N | T | G |
| 104 UniRef90\_A0A0D2FPI6\_31\_423 | G | A | V | - | - | - | - | - | - | N | P | - | - | - | - | - | R | N | A | D | L | - | K | I | - | - | - | - | T | S | D | - | - | - | - | G | E | E | I | V | F | N | G | F | K | S | F | N | T | G |
| 105 UniRef90\_A0A2S0KGS2\_30\_403 | G | V | Q | - | - | - | - | - | - | N | P | - | - | - | - | - | R | G | G | S | A | L | E | L | - | - | - | - | T | R | D | - | - | - | - | G | D | G | F | R | L | N | G | R | R | T | F | A | S | G |
| 106 UniRef90\_A0A1A7MGQ0\_54\_442 | G | A | V | - | - | - | - | - | - | N | P | - | - | - | - | - | R | D | N | D | L | - | A | I | - | - | - | - | S | D | H | - | - | - | - | G | D | H | L | V | F | S | G | S | K | H | F | N | T | G |
| 107 UniRef90\_UPI000413001D\_18\_392 | G | A | S | - | - | - | - | - | - | N | P | - | - | - | - | - | R | D | P | E | S | - | Q | L | - | - | - | - | T | A | D | - | - | - | - | G | P | D | F | R | L | N | G | R | K | T | F | A | S | N |
| 108 UniRef90\_G7H5P9\_11\_400 | G | V | Q | - | - | - | - | - | - | N | P | - | - | - | - | - | R | G | G | S | A | L | E | L | - | - | - | - | T | R | D | - | - | - | - | G | D | G | F | R | L | N | G | R | R | T | F | A | S | G |
| 109 UniRef90\_A0A010YHQ5\_11\_404 | G | A | G | - | - | - | - | - | - | N | P | - | - | - | - | - | R | D | A | G | L | - | E | L | - | - | - | - | E | P | T | - | - | - | - | S | G | G | Y | L | V | R | G | K | K | F | F | A | T | G |
| 110 UniRef90\_B0RBU7\_24\_404 | D | S | V | - | - | - | - | - | - | N | P | - | - | - | - | - | T | D | P | D | L | - | R | L | - | - | - | - | T | P | D | - | - | - | - | G | D | G | Y | Q | L | D | G | L | K | R | F | S | T | G |
| 111 UniRef90\_A0A167RW81\_42\_429 | G | A | V | - | - | - | - | - | - | N | P | - | - | - | - | - | R | D | S | D | L | - | R | I | - | - | - | - | T | L | D | D | A | A | G | G | D | H | L | V | Y | N | G | A | K | H | F | N | T | G |
| 112 UniRef90\_A0A1X0J8A0\_26\_405 | S | V | S | - | - | - | - | - | - | N | P | - | - | - | - | - | R | D | P | G | I | - | R | V | - | - | - | - | T | R | T | - | - | - | - | D | E | G | Y | R | V | D | G | K | R | T | F | A | T | G |
| 113 UniRef90\_A0A2J6Q859\_43\_430 | G | A | V | - | - | - | - | - | - | N | P | - | - | - | - | - | R | N | S | D | L | - | K | I | - | - | - | - | T | S | D | - | - | - | - | G | E | D | I | V | F | N | G | T | K | F | F | N | T | G |
| 114 UniRef90\_UPI0003828F02\_7\_389 | G | V | A | - | - | - | - | - | - | N | P | - | - | - | - | - | R | D | D | A | L | - | T | V | - | - | - | - | S | D | V | - | - | - | - | D | G | G | F | A | V | A | G | R | K | F | F | A | T | G |
| 115 UniRef90\_A0A1B2HDJ6\_6\_361 | G | V | S | - | - | - | - | - | - | N | P | - | - | - | - | - | L | D | A | A | L | - | E | L | - | - | - | - | T | P | T | - | - | - | - | G | T | G | F | L | L | N | G | R | K | T | F | A | T | G |
| 116 UniRef90\_A0A2N3N0U1\_47\_427 | G | A | V | - | - | - | - | - | - | N | P | - | - | - | - | - | R | D | G | D | L | - | K | I | - | - | - | - | T | E | D | - | - | - | - | G | D | N | L | V | F | N | G | F | K | N | F | S | T | G |
| 117 UniRef90\_S5Y4X6\_32\_408 | G | V | S | - | - | - | - | - | - | N | P | - | - | - | - | - | R | D | Q | W | S | - | E | I | - | - | - | - | E | T | E | - | - | - | - | G | T | R | R | F | I | S | G | K | R | S | F | A | T | G |
| 118 UniRef90\_A0A3R2WQN2\_17\_411 | G | A | V | - | - | - | - | - | - | N | P | - | - | - | - | - | R | D | G | D | L | - | V | I | - | - | - | - | T | E | V | - | - | - | - | D | G | Q | L | A | Y | N | G | R | K | S | F | S | T | G |
| 119 UniRef90\_A0A3N1X255\_20\_392 | N | S | G | - | - | - | - | - | - | N | V | - | - | - | - | - | M | S | R | T | S | - | T | A | - | - | - | - | R | R | V | - | - | - | - | G | T | H | W | V | L | D | G | H | R | P | F | S | S | G |
| 120 UniRef90\_A0A1S1LA29\_28\_404 | S | V | S | - | - | - | - | - | - | N | P | - | - | - | - | - | R | D | P | G | I | - | T | V | - | - | - | - | T | R | T | - | - | - | - | E | E | G | Y | R | V | N | G | K | R | T | F | A | T | G |
| 121 UniRef90\_A0A081GNL4\_19\_392 | N | A | L | - | - | - | - | - | - | N | P | - | - | - | - | - | L | D | R | R | S | - | R | L | - | - | - | - | R | R | D | - | - | - | - | G | D | G | W | R | L | E | G | S | K | S | F | C | S | G |
| 122 UniRef90\_A0A1I6U758\_27\_419 | G | V | V | - | - | - | - | - | - | N | P | - | - | - | - | - | R | D | R | D | L | - | I | V | - | - | - | - | T | D | E | - | - | - | - | G | E | T | L | L | Y | R | G | K | K | S | F | S | T | G |
| 123 UniRef90\_A0A0F5VZP9\_7\_388 | N | A | Q | S | E | A | G | T | R | H | V | - | - | - | - | - | Q | D | I | R | T | - | R | L | - | - | - | - | A | P | R | - | - | - | P | D | G | S | Y | V | L | D | G | V | K | H | Y | S | T | G |
| 124 UniRef90\_A0A1H4GIM8\_20\_411 | G | A | V | - | - | - | - | - | - | N | P | - | - | - | - | - | R | D | S | D | L | - | T | I | - | - | - | - | R | E | D | - | - | - | - | G | D | E | L | V | Y | S | G | R | K | S | F | S | T | G |
| 125 UniRef90\_A0A0M8TTT1\_8\_388 | N | A | Q | S | E | A | G | T | K | H | V | - | - | - | - | - | Q | D | I | R | T | - | R | L | - | - | - | - | A | P | R | - | - | - | P | D | G | S | Y | T | L | T | G | V | K | H | Y | S | T | G |
| 126 UniRef90\_A0A1Y2MDA3\_48\_437 | G | A | V | - | - | - | - | - | - | N | P | - | - | - | - | - | R | D | N | D | L | - | K | I | - | - | - | - | T | P | E | - | - | - | - | G | D | K | L | V | F | N | G | F | K | H | F | N | T | G |
| 127 UniRef90\_UPI000562F0C5\_9\_388 | N | A | Q | S | E | A | G | T | R | H | V | - | - | - | - | - | Q | D | I | R | T | - | R | L | - | - | - | - | I | P | R | - | - | - | P | D | G | S | Y | V | L | S | G | V | K | H | Y | A | T | G |
| 128 UniRef90\_A0A506Y8Z1\_43\_442 | G | A | V | - | - | - | - | - | - | N | P | - | - | - | - | - | R | D | A | D | L | - | T | V | - | - | - | - | R | D | E | - | - | - | - | G | D | T | L | V | F | S | G | R | K | S | F | S | T | G |
| 129 UniRef90\_A0A021VVW0\_28\_409 | G | A | V | - | - | - | - | - | - | N | P | - | - | - | - | - | R | D | A | D | L | - | V | A | - | - | - | - | V | D | Q | - | - | - | - | G | D | H | L | V | V | S | G | H | K | T | F | S | T | G |
| 130 UniRef90\_E3QTS2\_46\_428 | G | A | V | - | - | - | - | - | - | N | P | - | - | - | - | - | R | D | S | D | H | - | K | I | - | - | - | - | T | S | D | - | - | - | - | G | D | K | I | V | F | N | G | L | K | H | F | N | T | G |
| 131 UniRef90\_A0A1D8SMV7\_7\_388 | N | A | Q | S | E | A | G | T | K | H | V | - | - | - | - | - | Q | D | I | R | T | - | R | L | - | - | - | - | T | R | R | - | - | - | P | D | G | S | Y | A | L | D | G | V | K | H | Y | S | T | G |
| 132 UniRef90\_A9BUV8\_38\_419 | N | A | L | A | E | I | G | H | R | D | - | - | - | - | - | - | F | Q | R | R | T | - | R | L | - | - | - | - | L | R | D | - | - | - | - | S | G | G | W | F | V | Q | G | R | K | F | Y | C | T | G |
| 133 UniRef90\_A0A1N6TB45\_32\_407 | N | T | A | - | - | - | - | - | - | N | S | - | - | - | - | - | F | S | R | S | L | - | F | G | - | - | - | - | R | R | E | - | - | - | - | G | D | H | F | V | L | D | G | F | R | P | F | T | S | G |
| 134 UniRef90\_A0A381IE41\_24\_406 | N | G | F | S | E | K | G | T | K | N | V | - | - | - | - | - | L | D | L | K | T | - | K | V | - | - | - | - | I | R | D | - | - | - | - | G | D | G | Y | R | V | D | G | T | K | F | Y | S | T | G |
| 135 UniRef90\_A0A3M8TJ33\_18\_397 | G | G | L | - | - | - | - | - | - | A | S | - | - | - | - | - | V | E | P | R | L | - | T | L | - | - | - | - | T | P | G | - | - | - | - | P | G | G | Y | V | L | N | G | R | Q | G | Y | A | T | G |
| 136 UniRef90\_A0A1H3JL41\_4\_373 | G | V | S | - | - | - | S | P | P | N | P | - | - | - | - | - | A | D | A | G | L | - | S | M | - | - | - | - | T | T | V | - | - | - | - | D | G | G | F | L | V | N | G | R | R | T | F | V | T | G |
| 137 UniRef90\_A0A507AW89\_49\_427 | G | A | V | - | - | - | - | - | - | N | P | - | - | - | - | - | R | D | Q | D | L | - | R | I | - | - | - | - | T | S | D | - | - | - | - | G | D | D | I | V | F | N | G | F | K | F | F | N | T | G |
| 138 UniRef90\_A0A1X1SEN2\_20\_406 | T | V | A | - | - | - | - | - | - | Q | A | - | - | - | - | - | A | Y | P | P | L | I | S | A | - | - | - | - | D | P | T | - | - | - | - | P | G | G | F | V | L | N | G | S | K | P | F | T | S | G |
| 139 UniRef90\_A0A2D3UFX4\_26\_403 | G | G | F | - | - | - | - | - | - | A | R | - | - | - | - | - | Q | E | L | P | L | - | T | L | - | - | - | - | A | R | T | - | - | - | - | A | D | G | Y | V | L | D | G | R | Q | S | Y | A | T | G |
| 140 UniRef90\_A0A089X0S9\_26\_403 | G | G | L | - | - | - | - | - | - | A | R | - | - | - | - | - | Q | E | P | A | L | - | A | L | - | - | - | - | T | P | S | - | - | - | - | A | G | G | Q | V | L | D | G | R | Q | S | Y | V | T | G |
| 141 UniRef90\_A0A2W5T1D0\_29\_413 | G | A | V | - | - | - | - | - | - | N | P | - | - | - | - | - | R | D | A | D | L | - | T | V | - | - | - | - | R | D | E | - | - | - | - | G | D | E | L | V | F | T | G | R | K | S | F | S | T | G |
| 142 UniRef90\_A0A1C4QGR9\_7\_388 | N | A | Q | S | E | A | G | T | R | H | V | - | - | - | - | - | Q | D | I | G | T | - | R | L | - | - | - | - | T | P | R | - | - | - | P | D | G | S | L | L | L | T | G | E | K | H | Y | S | T | G |
| 143 UniRef90\_A0A101R4L6\_7\_388 | N | A | Q | S | E | A | G | T | K | H | V | - | - | - | - | - | Q | D | I | R | T | - | R | L | - | - | - | - | T | P | R | - | - | - | P | D | G | S | Y | R | L | D | G | V | K | H | Y | S | T | G |
| 144 UniRef90\_A0A4D4LHU8\_7\_388 | N | A | Q | S | E | A | G | T | R | H | V | - | - | - | - | - | Q | D | I | R | T | - | R | L | - | - | - | - | A | P | R | - | - | - | P | D | G | S | Y | L | L | T | G | V | K | H | Y | S | T | G |
| 145 UniRef90\_L1KK35\_6\_389 | N | A | Q | S | E | A | G | T | Q | H | V | - | - | - | - | - | Q | D | I | R | T | - | R | L | - | - | - | - | T | R | L | - | - | - | P | D | G | S | Y | A | L | D | G | V | K | H | Y | S | T | G |
| 146 UniRef90\_UPI0005601C6B\_13\_390 | N | A | Q | S | E | A | G | T | K | H | V | - | - | - | - | - | Q | D | I | R | T | - | R | L | - | - | - | - | E | S | R | - | - | - | P | D | G | S | Y | L | L | D | G | E | K | H | Y | S | T | G |
| 147 UniRef90\_A0A1H9UQJ9\_6\_361 | G | V | S | - | - | - | - | - | - | N | P | - | - | - | - | - | L | D | A | A | L | - | E | L | - | - | - | - | T | P | A | - | - | - | - | G | D | G | Y | V | L | N | G | R | K | T | F | A | T | G |
| 148 UniRef90\_A0A3D9JKY9\_12\_401 | G | G | I | - | - | - | - | - | - | A | S | - | - | - | - | - | H | E | P | P | L | - | V | L | - | - | - | - | T | P | T | - | - | - | - | G | D | G | Y | L | L | D | G | Y | Q | R | Y | A | A | G |
| 149 UniRef90\_D9X7Q2\_26\_403 | G | G | L | - | - | - | - | - | - | A | R | - | - | - | - | - | Q | E | P | A | L | - | T | I | - | - | - | - | A | R | N | - | - | - | - | A | T | G | Y | V | L | N | G | R | Q | S | Y | A | A | G |
| 150 UniRef90\_A0A1X1DXJ3\_20\_398 | N | G | G | P | E | K | N | T | R | H | T | - | - | - | - | - | R | D | V | Q | A | - | R | L | - | - | - | - | S | G | K | - | - | - | - | G | D | S | R | F | L | T | G | E | K | F | Y | S | T | G |

  
  

|  |  |  |  |  |  |  |  |  |  |  |  |  |  |  |  |  |  |  |  |  |  |  |  |  |  |  |  |  |  |  |  |  |  |  |  |  |  |  |  |  |  |  |  |  |  |  |  |  |  |  |
| --- | --- | --- | --- | --- | --- | --- | --- | --- | --- | --- | --- | --- | --- | --- | --- | --- | --- | --- | --- | --- | --- | --- | --- | --- | --- | --- | --- | --- | --- | --- | --- | --- | --- | --- | --- | --- | --- | --- | --- | --- | --- | --- | --- | --- | --- | --- | --- | --- | --- | --- |
| **001 Input\_pdb\_SEQRES\_A** | A | K | G | S | D | L | L | F | V | F | G | V | V | Q | - | - | - | - | - | D | D | S | P | Q | - | - | - | - | - | Q | G | A | - | - | - | - | - | - | I | I | A | A | A | I | P | - | - | T | S | R |
| 002 UniRef90\_Q1W1G3\_1\_416 | A | K | G | S | D | L | L | L | V | F | G | V | I | Q | - | - | - | - | - | D | D | S | P | Q | - | - | - | - | - | Q | G | A | - | - | - | - | - | - | I | I | A | A | V | I | P | - | - | T | S | R |
| 003 UniRef90\_T0BM21\_7\_392 | A | Q | D | S | D | R | L | L | V | S | W | V | - | - | - | - | - | - | - | - | - | E | G | D | - | - | - | - | D | P | A | A | - | - | - | - | - | - | L | Y | T | A | V | I | P | - | - | T | R | R |
| 004 UniRef90\_UPI0002AC58FE\_11\_391 | S | K | D | S | D | I | L | P | I | T | A | T | - | - | - | - | - | - | - | - | - | N | N | D | - | - | - | - | - | - | - | E | - | - | - | - | - | - | I | T | I | L | A | I | P | - | - | T | Q | R |
| 005 UniRef90\_A0A3D5CW51\_20\_401 | A | M | D | S | E | M | L | I | A | S | A | I | - | - | - | - | - | - | - | - | - | K | K | T | - | - | - | - | - | D | G | Q | - | - | - | - | - | - | L | V | I | A | A | I | P | - | - | T | L | R |
| 006 UniRef90\_A0A2V4SPP5\_16\_400 | S | V | G | S | D | W | L | T | V | S | A | W | - | - | - | - | - | - | - | - | - | D | S | E | - | - | - | - | - | A | D | A | - | - | - | - | - | - | A | L | I | A | V | V | P | - | - | T | E | Q |
| 007 UniRef90\_E0UIV5\_8\_393 | A | K | D | S | D | I | L | P | L | T | A | I | - | - | - | - | - | - | - | - | - | H | Q | E | - | - | - | - | - | T | G | E | - | - | - | - | - | - | L | T | V | L | A | I | P | - | - | T | S | R |
| 008 UniRef90\_A0A1Z4S897\_9\_391 | T | K | C | S | D | V | V | L | V | G | G | R | - | - | - | - | - | - | - | - | - | R | E | D | - | - | - | - | - | L | G | N | - | - | - | - | - | - | V | V | Y | A | V | I | P | - | - | S | D | R |
| 009 UniRef90\_UPI00045E9273\_21\_396 | A | S | D | S | D | H | L | I | V | S | A | L | - | - | - | - | - | - | - | - | - | T | E | A | - | - | - | - | - | G | G | K | - | - | - | - | - | - | L | V | I | A | A | I | P | - | - | S | D | R |
| 010 UniRef90\_A0A353Y4M9\_17\_384 | A | V | G | A | D | V | L | L | V | S | A | Y | - | - | - | - | - | - | - | - | - | L | E | - | - | - | - | - | - | C | G | R | - | - | - | - | - | - | F | I | V | A | A | I | P | - | - | A | H | R |
| 011 UniRef90\_F3KR10\_17\_384 | A | V | G | A | D | V | L | L | V | S | A | H | - | - | - | - | - | - | - | - | - | L | E | - | - | - | - | - | - | C | G | R | - | - | - | - | - | - | L | I | A | A | T | V | P | - | - | T | D | R |
| 012 UniRef90\_A0A2D8NW56\_20\_404 | A | L | G | S | D | Y | L | T | A | S | A | W | - | - | - | - | - | - | - | - | - | Y | E | A | - | - | - | - | - | S | Q | S | - | - | - | - | - | - | L | V | V | A | A | I | P | - | - | T | G | R |
| 013 UniRef90\_A0A2W7M9P1\_16\_386 | A | T | D | S | D | R | L | L | I | S | A | Q | - | - | - | - | - | - | - | - | - | K | E | D | - | - | - | - | - | G | S | G | - | - | - | - | - | - | V | V | I | A | V | I | P | - | - | S | I | R |
| 014 UniRef90\_A0A398AYR9\_12\_386 | A | V | D | S | D | R | L | L | I | S | A | Q | - | - | - | - | - | - | - | - | - | K | D | D | - | - | - | - | - | G | S | G | - | - | - | - | - | - | V | L | V | A | V | I | P | - | - | T | N | R |
| 015 UniRef90\_A0A0T6UXN9\_11\_394 | A | T | D | S | E | M | L | I | A | S | A | V | - | - | - | - | - | - | - | - | - | D | E | N | - | - | - | - | A | S | G | K | - | - | - | - | - | - | L | V | I | A | A | I | P | - | - | T | G | R |
| 016 UniRef90\_A0A252E884\_11\_390 | V | A | I | A | D | L | R | V | F | S | A | L | - | - | - | - | - | - | - | - | - | Q | D | G | - | - | - | - | - | V | E | A | - | - | - | - | - | - | P | W | L | F | V | I | P | - | - | K | D | R |
| 017 UniRef90\_A0A1Z4BZ71\_24\_403 | A | T | D | S | D | R | L | I | V | S | A | I | - | - | - | - | - | - | - | - | - | T | A | D | - | - | - | - | - | T | K | K | - | - | - | - | - | - | F | I | V | A | A | V | P | - | - | T | Q | R |
| 018 UniRef90\_A0A1Z4IGC5\_10\_387 | A | Q | D | S | D | M | L | T | V | S | A | L | - | - | - | - | - | - | - | - | - | P | I | G | - | - | - | - | - | E | S | Q | - | - | - | - | - | - | P | V | V | V | A | I | P | - | - | T | F | S |
| 019 UniRef90\_A0A1W9JD13\_18\_401 | A | I | D | S | E | M | L | I | A | S | A | I | - | - | - | - | - | - | - | - | - | K | Q | K | - | - | - | - | - | N | G | K | - | - | - | - | - | - | L | V | I | A | A | I | P | - | - | T | S | R |
| 020 UniRef90\_A0A0B6S5D2\_8\_393 | T | R | G | S | S | R | M | T | I | S | A | H | - | - | - | - | - | - | - | - | - | D | P | A | - | - | - | - | - | T | G | K | - | - | - | - | - | - | P | V | F | A | V | V | P | - | - | T | A | R |
| 021 UniRef90\_A0A1Y3C786\_14\_396 | A | L | D | S | E | M | L | I | A | S | G | I | - | - | - | - | - | - | - | - | - | D | E | N | - | - | - | - | - | N | G | K | - | - | - | - | - | - | L | L | I | A | A | V | P | - | - | T | S | R |
| 022 UniRef90\_A0A318KD41\_20\_397 | A | L | D | S | D | M | L | V | V | S | A | R | - | - | - | - | - | - | - | - | - | Q | P | G | - | - | - | - | - | A | D | K | - | - | - | - | - | - | L | L | I | A | A | V | P | - | - | S | R | R |
| 023 UniRef90\_A0A352JDP6\_15\_389 | S | K | D | S | D | I | L | P | I | T | A | V | - | - | - | - | - | - | - | - | - | N | Q | E | - | - | - | - | - | T | S | E | - | - | - | - | - | - | L | L | A | L | A | I | P | - | - | T | N | R |
| 024 UniRef90\_A0A329B538\_13\_401 | A | L | D | S | E | M | L | I | V | S | A | R | - | - | - | - | - | - | - | - | - | H | A | D | - | - | - | - | - | D | Q | R | - | - | - | - | - | - | L | L | I | A | A | V | P | - | - | T | A | R |
| 025 UniRef90\_A0A2N8QAF3\_13\_394 | T | R | G | S | H | M | M | T | V | S | A | L | - | - | - | - | - | - | - | - | - | D | P | T | - | - | - | - | - | T | G | K | - | - | - | - | - | - | A | V | F | A | V | V | P | - | - | T | T | R |
| 026 UniRef90\_A0A2N7XWT8\_11\_394 | A | N | D | S | E | M | L | I | A | S | A | I | - | - | - | - | - | - | - | - | - | D | E | S | - | - | - | - | A | G | G | K | - | - | - | - | - | - | L | L | I | A | A | I | P | - | - | S | A | R |
| 027 UniRef90\_A0A2A4HLB2\_33\_412 | A | R | G | S | D | Y | L | T | V | S | A | W | - | - | - | - | - | - | - | - | - | H | E | P | - | - | - | - | - | T | Q | S | - | - | - | - | - | - | L | V | I | A | A | V | P | - | - | T | H | R |
| 028 UniRef90\_A0A4R3HWL3\_41\_416 | A | T | G | S | D | M | L | N | V | T | A | P | - | - | - | - | - | - | - | - | - | H | P | E | - | - | - | - | R | P | D | E | - | - | - | - | - | - | R | V | F | F | A | I | P | - | - | T | K | R |
| 029 UniRef90\_A0A1M7NXP6\_14\_394 | S | V | G | S | D | W | L | T | I | S | A | W | - | - | - | - | - | - | - | - | - | H | A | E | - | - | - | - | - | T | Q | S | - | - | - | - | - | - | A | L | I | G | V | V | P | - | - | A | R | Q |
| 030 UniRef90\_A0A1B4ESK2\_22\_397 | T | R | G | S | Q | R | M | T | V | S | A | H | - | - | - | - | - | - | - | - | - | D | P | E | - | - | - | - | - | T | G | R | - | - | - | - | - | - | A | V | F | G | V | V | P | - | - | T | D | R |
| 031 UniRef90\_A0A1H6NKV2\_11\_394 | A | S | D | S | Q | M | L | I | A | S | A | V | - | - | - | - | - | - | - | - | - | D | E | S | - | - | - | - | A | G | G | K | - | - | - | - | - | - | L | L | I | A | A | I | P | - | - | S | G | R |
| 032 UniRef90\_A0A0D0KUF8\_11\_394 | A | T | D | S | E | M | L | I | A | S | A | V | - | - | - | - | - | - | - | - | - | D | E | S | - | - | - | - | A | G | G | K | - | - | - | - | - | - | L | L | I | A | A | I | P | - | - | S | G | R |
| 033 UniRef90\_A0A0F3K7B4\_20\_401 | A | L | D | S | Q | M | L | V | V | S | A | R | - | - | - | - | - | - | - | - | - | Q | Q | H | - | - | - | - | - | S | G | Q | - | - | - | - | - | - | L | V | V | A | A | I | P | - | - | T | G | R |
| 034 UniRef90\_A0A381IMG6\_13\_394 | T | R | G | S | Q | R | M | T | I | S | A | H | - | - | - | - | - | - | - | - | - | D | P | D | - | - | - | - | - | T | G | K | - | - | - | - | - | - | P | V | F | A | V | V | P | - | - | T | E | R |
| 035 UniRef90\_A0A2S9K1G1\_26\_400 | S | V | G | S | D | R | L | T | L | S | A | W | - | - | - | - | - | - | - | - | - | H | E | P | - | - | - | - | - | S | Q | S | - | - | - | - | - | - | L | L | I | G | A | L | P | - | - | S | D | R |
| 036 UniRef90\_A0A1W6L7W5\_17\_392 | A | L | G | S | D | Q | L | V | V | S | A | W | - | - | - | - | - | - | - | - | - | L | P | D | - | - | - | - | - | R | E | A | - | - | - | - | - | - | L | L | V | G | V | V | P | - | - | T | G | R |
| 037 UniRef90\_A0A255HJE3\_14\_398 | S | V | G | S | D | W | L | T | V | S | A | W | - | - | - | - | - | - | - | - | - | H | A | P | - | - | - | - | - | T | Q | T | - | - | - | - | - | - | A | L | I | A | A | L | P | - | - | T | R | Q |
| 038 UniRef90\_A0A0R3AD45\_11\_394 | A | S | D | S | E | M | L | I | A | S | A | V | - | - | - | - | - | - | - | - | - | D | E | S | - | - | - | - | A | G | G | K | - | - | - | - | - | - | L | L | I | A | A | I | P | - | - | S | G | R |
| 039 UniRef90\_A0A1W6ZB60\_16\_389 | S | V | G | S | D | I | L | T | L | S | A | W | - | - | - | - | - | - | - | - | - | H | K | P | - | - | - | - | - | T | E | T | - | - | - | - | - | - | A | L | I | A | A | L | P | - | - | T | D | A |
| 040 UniRef90\_A0A2X1DPB7\_14\_397 | T | R | G | S | Q | R | M | T | V | S | A | H | - | - | - | - | - | - | - | - | - | D | P | A | - | - | - | - | - | I | G | R | - | - | - | - | - | - | T | V | F | G | V | V | P | - | - | T | D | R |
| 041 UniRef90\_A0A0S9M2D1\_4\_380 | A | L | D | S | Q | M | L | V | A | S | A | L | - | - | - | - | - | - | - | - | - | H | A | R | - | - | - | - | - | T | R | Q | - | - | - | - | - | - | L | L | V | A | A | V | P | - | - | T | A | R |
| 042 UniRef90\_A0A238ZKW1\_13\_401 | A | M | D | S | E | M | L | I | V | S | A | Y | - | - | - | - | - | - | - | - | - | A | Q | D | - | - | - | - | - | T | K | E | - | - | - | - | - | - | L | L | I | A | A | L | P | - | - | T | A | R |
| 043 UniRef90\_A0A0D1P8V6\_10\_394 | A | S | D | S | Q | M | L | I | A | S | A | V | - | - | - | - | - | - | - | - | - | D | E | A | - | - | - | - | A | G | G | K | - | - | - | - | - | - | L | L | I | A | A | I | P | - | - | S | G | R |
| 044 UniRef90\_A0A261SP68\_11\_387 | S | I | G | S | D | V | L | T | I | S | A | W | - | - | - | - | - | - | - | - | - | H | R | A | - | - | - | - | - | S | G | T | - | - | - | - | - | - | A | L | V | A | A | L | P | - | - | T | D | T |
| 045 UniRef90\_A0A2N6MRB2\_11\_390 | V | A | I | A | D | L | R | V | F | S | A | V | - | - | - | - | - | - | - | - | - | Q | D | G | - | - | - | - | - | I | E | V | - | - | - | - | - | - | P | W | L | F | I | I | P | - | - | K | D | R |
| 046 UniRef90\_A0A212BVA1\_31\_405 | A | L | G | A | D | A | L | V | V | S | A | P | - | - | - | - | - | - | - | - | - | R | G | G | - | - | - | - | S | T | S | E | - | - | - | - | - | - | R | A | F | L | V | L | P | - | - | T | Q | R |
| 047 UniRef90\_A0A1A9KE82\_11\_394 | A | N | D | S | E | M | L | I | A | S | A | V | - | - | - | - | - | - | - | - | - | D | E | S | - | - | - | - | A | G | G | K | - | - | - | - | - | - | L | L | I | A | A | L | P | - | - | S | G | R |
| 048 UniRef90\_A0A4Q5PVB9\_26\_400 | A | K | D | S | D | V | M | N | V | S | I | A | T | - | - | - | - | - | - | - | - | G | P | V | - | - | - | - | - | P | A | D | - | - | - | - | - | - | R | L | Y | A | V | V | P | - | - | T | G | R |
| 049 UniRef90\_A0A178GPM9\_9\_392 | S | I | D | S | D | M | L | L | C | S | G | Y | - | - | - | - | - | - | - | - | - | N | D | - | - | - | - | - | - | A | G | K | - | - | - | - | - | - | L | L | I | G | V | I | P | - | - | T | Q | R |
| 050 UniRef90\_A0A1P9YC32\_3\_390 | A | R | D | S | D | R | L | L | A | S | A | F | - | - | - | - | - | - | - | - | - | D | G | - | - | - | - | - | - | D | G | R | - | - | - | - | - | - | L | L | I | G | V | T | P | - | - | T | N | R |
| 051 UniRef90\_UPI000A1773A9\_40\_416 | A | L | D | S | E | M | L | I | V | S | G | H | - | - | - | - | - | - | - | - | - | R | E | T | - | - | - | - | - | D | G | S | - | - | - | - | - | - | L | V | V | A | A | V | P | - | - | T | G | R |
| 052 UniRef90\_A0A158L201\_25\_395 | T | R | G | S | Q | M | M | T | L | S | A | H | - | - | - | - | - | - | - | - | - | D | P | A | - | - | - | - | - | T | G | K | - | - | - | - | - | - | P | I | F | A | V | V | P | - | - | T | T | R |
| 053 UniRef90\_A0A4Q4GT15\_13\_391 | S | I | D | S | D | I | L | L | C | S | G | F | - | - | - | - | - | - | - | - | - | N | E | - | - | - | - | - | - | A | G | K | - | - | - | - | - | - | L | L | I | G | V | I | P | - | - | T | T | R |
| 054 UniRef90\_A0A1C0YC62\_17\_381 | A | S | D | A | D | C | L | L | V | S | A | Q | - | - | - | - | - | - | - | - | - | K | K | D | - | - | - | - | - | G | S | G | - | - | - | - | - | - | L | L | M | A | V | I | P | - | - | A | N | R |
| 055 UniRef90\_A0A1H7FE04\_32\_405 | A | L | G | S | D | R | L | T | V | S | A | T | - | - | - | - | - | - | - | - | - | T | P | - | - | - | - | - | - | D | G | G | - | - | - | - | - | - | F | L | T | G | V | V | P | - | - | T | R | R |
| 056 UniRef90\_A0A401MWC4\_5\_385 | A | R | V | A | D | R | L | V | V | G | A | T | - | - | - | - | - | - | - | - | - | R | T | H | - | - | - | - | - | T | G | E | - | - | - | - | - | - | P | V | V | V | F | L | D | - | - | P | A | H |
| 057 UniRef90\_A0A1G6HFW7\_16\_395 | A | A | D | S | D | R | L | L | A | S | A | F | - | - | - | - | - | - | - | - | - | D | E | - | - | - | - | - | - | Q | G | R | - | - | - | - | - | - | L | L | I | G | V | V | P | - | - | T | S | R |
| 058 UniRef90\_A0A0P9B999\_30\_403 | A | P | D | S | D | V | L | N | V | S | V | V | L | - | - | - | - | - | - | - | - | G | P | E | - | - | - | - | - | P | T | D | - | - | - | - | - | - | R | L | F | A | V | V | P | - | - | T | S | R |
| 059 UniRef90\_A0A140K6F0\_14\_392 | V | A | V | G | D | L | N | V | I | G | A | S | - | - | - | - | - | - | - | - | - | M | E | G | - | - | - | - | - | V | E | T | - | - | - | - | - | - | P | I | V | F | I | L | P | - | - | K | N | R |
| 060 UniRef90\_A0A1P8EKI7\_5\_392 | S | I | D | S | D | M | L | L | C | S | G | Y | - | - | - | - | - | - | - | - | - | T | Q | - | - | - | - | - | - | E | D | K | - | - | - | - | - | - | L | L | I | A | V | V | P | - | - | T | Q | R |
| 061 UniRef90\_A0A2U3MYZ3\_7\_392 | S | I | D | S | D | I | L | L | C | S | G | Y | - | - | - | - | - | - | - | - | - | D | S | - | - | - | - | - | - | A | S | K | - | - | - | - | - | - | L | L | I | G | V | I | P | - | - | T | Q | R |
| 062 UniRef90\_A0A1H2EPX5\_7\_397 | S | V | D | A | D | A | L | V | V | S | A | S | - | - | - | - | - | - | - | - | - | D | E | A | - | - | - | - | H | G | G | K | - | - | - | - | - | - | L | L | V | G | V | L | P | L | A | E | R | R |
| 063 UniRef90\_UPI00041EF43F\_25\_400 | S | V | G | S | D | Q | L | T | F | S | A | W | - | - | - | - | - | - | - | - | - | H | E | A | - | - | - | - | - | S | Q | S | - | - | - | - | - | - | L | L | I | A | A | L | P | - | - | S | R | H |
| 064 UniRef90\_D0IW93\_17\_384 | T | R | G | S | H | Y | L | T | V | S | A | S | - | - | - | - | - | - | - | - | - | V | A | G | - | - | - | - | - | H | A | Q | - | - | - | - | - | - | P | V | L | G | L | L | E | - | - | T | A | S |
| 065 UniRef90\_A0A395D1I6\_11\_388 | A | S | D | S | D | M | I | I | V | S | A | L | - | - | - | - | - | - | - | - | - | D | R | E | - | - | - | - | - | T | K | A | - | - | - | - | - | - | L | R | V | A | A | I | P | - | - | T | R | R |
| 066 UniRef90\_A0A1H0TK45\_27\_400 | A | S | D | S | D | M | L | L | A | S | A | L | - | - | - | - | - | - | - | - | - | D | V | - | - | - | - | - | - | H | G | R | - | - | - | - | - | - | L | L | I | S | V | V | P | - | - | T | A | R |
| 067 UniRef90\_A0A1H8MYU5\_23\_403 | S | H | V | A | D | Y | L | M | V | A | W | E | - | - | - | - | - | - | - | - | - | D | E | K | - | - | - | - | - | T | N | A | - | - | - | - | - | - | R | R | F | A | Y | I | P | - | - | A | N | R |
| 068 UniRef90\_A0A1B1M254\_5\_403 | S | K | V | S | D | V | T | V | L | E | G | V | - | - | - | - | - | - | - | - | - | L | D | G | - | - | - | - | - | T | D | Q | - | - | - | - | - | - | H | V | F | A | I | V | P | - | - | S | D | S |
| 069 UniRef90\_A0A484THM3\_28\_401 | A | L | G | S | D | V | L | N | V | S | V | A | L | - | - | - | - | - | - | - | - | G | P | A | - | - | - | - | - | P | T | D | - | - | - | - | - | - | R | L | F | L | V | V | P | - | - | T | S | R |
| 070 UniRef90\_A0A0Q8Q8X6\_8\_377 | T | R | G | S | S | Y | M | T | L | S | A | V | - | - | - | - | - | - | - | - | - | H | V | S | - | - | - | - | - | S | R | R | - | - | - | - | - | - | T | V | L | G | V | V | P | - | - | T | G | H |
| 071 UniRef90\_A0A0Q5QDL2\_32\_410 | S | H | V | S | D | V | T | V | L | E | G | V | - | - | - | - | - | - | - | - | - | L | E | T | - | - | - | - | - | T | G | A | - | - | - | - | - | - | H | I | F | A | I | V | P | - | - | S | N | S |
| 072 UniRef90\_A0A1A5XM62\_22\_406 | T | Y | G | S | Q | M | M | T | V | S | A | H | - | - | - | - | - | - | - | - | - | D | P | A | - | - | - | - | - | T | G | Q | - | - | - | - | - | - | P | V | F | A | V | V | P | - | - | T | T | R |
| 073 UniRef90\_A0A239EI90\_21\_397 | S | R | V | S | D | V | T | V | L | E | G | A | - | - | - | - | - | - | - | - | - | L | S | G | - | - | - | - | - | S | D | A | - | - | - | - | - | - | H | V | F | A | I | V | P | - | - | S | N | I |
| 074 UniRef90\_S5SWW2\_23\_403 | S | H | V | A | D | Y | L | L | V | A | W | E | - | - | - | - | - | - | - | - | - | N | E | E | - | - | - | - | - | T | N | E | - | - | - | - | - | - | R | S | F | A | H | I | P | - | - | A | N | R |
| 075 UniRef90\_A0A3A5JJY9\_31\_413 | S | V | D | A | D | Y | L | T | V | S | A | Y | - | - | - | - | - | - | - | - | - | D | E | A | - | - | - | - | - | S | D | A | - | - | - | - | - | - | L | V | I | A | A | I | P | - | - | G | D | R |
| 076 UniRef90\_A0A315ZU85\_7\_384 | V | S | S | G | D | V | S | V | V | S | G | Q | - | - | - | - | - | - | - | - | - | D | S | A | - | - | - | - | - | T | G | R | - | - | - | - | - | - | H | V | L | V | V | V | E | - | - | R | G | A |
| 077 UniRef90\_A0A3R9U899\_5\_381 | S | K | V | S | D | V | T | V | L | E | G | V | - | - | - | - | - | - | - | - | - | F | E | G | - | - | - | - | - | T | D | R | - | - | - | - | - | - | H | V | F | A | V | V | P | - | - | S | D | S |
| 078 UniRef90\_UPI000DD53D91\_7\_389 | S | H | V | A | D | Y | I | Q | I | S | W | E | - | - | - | - | - | - | - | - | - | N | - | A | - | - | - | - | - | D | G | A | - | - | - | - | - | - | R | L | T | A | A | V | P | - | - | A | D | R |
| 079 UniRef90\_UPI000DE4BE09\_13\_396 | A | H | V | S | D | Y | I | N | A | N | A | S | - | - | - | - | - | - | - | - | - | - | - | I | - | - | - | - | - | G | D | R | - | - | - | - | - | - | I | V | G | F | A | V | P | - | - | T | S | R |
| 080 UniRef90\_A0A2T0R7P7\_10\_381 | A | S | V | G | D | V | T | L | V | G | G | L | - | - | - | - | - | - | - | - | - | - | T | D | - | - | - | - | - | D | G | R | - | - | - | - | - | - | D | L | L | V | L | V | P | - | - | R | E | T |
| 081 UniRef90\_A0A4R1HYS3\_27\_404 | S | R | V | S | D | V | T | V | L | E | G | V | - | - | - | - | - | - | - | - | - | L | E | G | - | - | - | - | - | T | D | T | - | - | - | - | - | - | H | V | F | A | I | V | P | - | - | S | A | Q |
| 082 UniRef90\_UPI000D1537B0\_7\_385 | S | H | V | A | D | M | L | Y | I | G | W | E | - | - | - | - | - | - | - | - | - | D | E | A | - | - | - | - | - | G | A | G | - | - | - | - | - | - | K | R | F | A | A | I | P | - | - | A | S | R |
| 083 UniRef90\_A0A1G6ZNM7\_17\_409 | G | K | V | S | D | V | T | V | L | E | G | I | - | - | - | - | - | - | - | - | - | L | E | G | - | - | - | - | - | T | D | R | - | - | - | - | - | - | H | V | F | A | I | V | P | - | - | T | D | Q |
| 084 UniRef90\_UPI0003765980\_18\_389 | S | H | I | A | D | Y | I | Q | I | S | F | E | - | - | - | - | - | - | - | - | - | N | - | S | - | - | - | - | - | A | G | E | - | - | - | - | - | - | R | L | T | A | A | V | P | - | - | A | D | R |
| 085 UniRef90\_A0A071ICK8\_25\_398 | A | H | V | S | D | Y | I | N | A | N | A | A | - | - | - | - | - | - | - | - | - | - | - | L | - | - | - | - | - | D | G | K | - | - | - | - | - | - | V | A | S | F | A | V | P | - | - | T | N | R |
| 086 UniRef90\_A0A2L0WNE5\_33\_396 | A | H | V | S | D | Y | I | N | A | N | A | A | - | - | - | - | - | - | - | - | - | - | - | L | - | - | - | - | - | D | D | K | - | - | - | - | - | - | I | V | G | F | T | I | R | - | - | T | N | R |
| 087 UniRef90\_A0A1Q8CFJ2\_9\_380 | A | S | V | A | D | R | L | V | V | S | A | T | - | - | - | - | - | - | - | - | - | R | V | D | - | - | - | - | - | S | G | Q | - | - | - | - | - | - | K | L | T | F | V | V | D | - | - | A | H | A |
| 088 UniRef90\_UPI000835F605\_21\_394 | A | S | I | A | Q | I | L | T | V | T | V | W | - | - | - | - | - | - | - | - | - | D | G | - | - | - | - | - | - | - | E | K | - | - | - | - | - | - | R | V | H | F | Q | V | P | - | - | P | S | R |
| 089 UniRef90\_UPI000374AB86\_31\_398 | A | R | I | S | D | Y | I | N | A | N | A | T | - | - | - | - | - | - | - | - | - | - | - | L | - | - | - | - | - | A | G | R | - | - | - | - | - | - | I | A | S | F | A | V | P | - | - | T | N | R |
| 090 UniRef90\_A0A267RVL6\_22\_399 | A | A | V | S | Q | V | M | T | I | A | V | W | - | - | - | - | - | - | - | - | - | I | E | - | - | - | - | - | - | - | D | R | - | - | - | - | - | - | L | V | Q | Y | Q | V | T | - | - | A | D | R |
| 091 UniRef90\_M3VA40\_23\_395 | A | G | V | G | D | Q | L | S | V | T | A | T | - | - | - | - | - | - | - | - | - | - | - | F | - | - | - | - | - | G | D | A | - | - | - | - | - | - | L | V | F | L | S | L | P | - | - | A | G | K |
| 092 UniRef90\_A0A395GID9\_32\_425 | G | V | V | S | D | L | T | V | L | E | G | V | - | - | - | - | - | - | - | - | - | L | D | N | - | - | - | - | - | T | G | E | - | - | - | - | - | - | H | I | F | A | L | V | E | - | - | T | R | Q |
| 093 UniRef90\_UPI0008268C2F\_35\_406 | A | S | I | S | D | Q | L | S | V | T | A | A | - | - | - | - | - | - | - | - | - | - | - | L | - | - | - | - | - | D | G | D | - | - | - | - | - | - | L | V | F | L | S | I | P | - | - | T | R | R |
| 094 UniRef90\_A0A0S1XV73\_27\_397 | S | H | V | A | D | V | I | H | I | A | W | Q | - | - | - | - | - | - | - | - | - | D | - | S | - | - | - | - | - | T | G | A | - | - | - | - | - | - | R | W | S | A | L | I | P | - | - | A | D | R |
| 095 UniRef90\_A0A0F5N116\_19\_398 | A | S | T | G | D | Q | L | S | V | T | A | S | - | - | - | - | - | - | - | - | - | - | - | L | - | - | - | - | - | D | G | S | - | - | - | - | - | - | L | V | F | L | S | L | D | V | R | G | G | R |
| 096 UniRef90\_A0A1N6Z7J9\_40\_406 | A | H | V | S | D | R | I | N | A | N | A | A | - | - | - | - | - | - | - | - | - | - | - | Y | - | - | - | - | - | R | N | Q | - | - | - | - | - | - | V | A | N | L | V | L | P | - | - | T | D | R |
| 097 UniRef90\_A0A2V4UWQ2\_31\_411 | A | R | I | S | D | R | I | N | A | S | A | A | - | - | - | - | - | - | - | - | - | - | - | F | - | - | - | - | - | G | D | Q | - | - | - | - | - | - | I | A | S | L | V | L | P | - | - | T | D | R |
| 098 UniRef90\_A0A4P8KLT1\_5\_401 | S | G | V | G | E | A | L | L | I | N | A | V | V | E | - | - | - | - | - | - | D | G | P | D | - | - | - | - | - | A | G | A | - | - | - | - | - | - | F | A | F | L | V | L | P | - | - | Y | G | H |
| 099 UniRef90\_UPI0003068598\_3\_292 | T | R | G | S | Q | R | M | T | V | S | A | H | - | - | - | - | - | - | - | - | - | D | S | V | - | - | - | - | - | T | G | K | - | - | - | - | - | - | P | V | F | A | V | V | P | - | - | T | Q | R |
| 100 UniRef90\_UPI000DD585C3\_27\_403 | A | H | V | S | D | R | I | N | V | N | A | T | - | - | - | - | - | - | - | - | - | - | - | L | - | - | - | - | - | D | D | K | - | - | - | - | - | - | V | A | N | F | V | V | P | - | - | T | D | R |
| 101 UniRef90\_A0A1E3SMN8\_25\_404 | A | S | T | G | D | Q | L | S | V | T | A | A | - | - | - | - | - | - | - | - | - | - | - | L | - | - | - | - | - | D | G | E | - | - | - | - | - | - | L | V | F | L | S | L | D | V | R | G | G | R |
| 102 UniRef90\_UPI00045EBD18\_12\_396 | A | A | L | A | D | R | I | T | L | R | A | Q | L | - | - | - | - | - | - | - | - | - | - | - | - | - | - | - | - | G | D | G | - | - | - | - | - | - | F | A | V | L | V | V | P | - | - | G | D | R |
| 103 UniRef90\_A0A1Y1ZNX6\_42\_434 | G | V | I | S | D | L | T | V | L | E | G | V | - | - | - | - | - | - | - | - | - | L | D | G | - | - | - | - | - | T | T | D | - | - | - | - | - | - | H | I | F | A | F | A | P | - | - | T | N | Q |
| 104 UniRef90\_A0A0D2FPI6\_31\_423 | G | V | V | S | D | A | T | I | L | E | G | V | - | - | - | - | - | - | - | - | - | L | D | G | - | - | - | - | - | T | E | D | - | - | - | - | - | - | H | I | F | T | I | V | P | - | - | T | K | Q |
| 105 UniRef90\_A0A2S0KGS2\_30\_403 | A | S | I | A | D | Q | L | S | V | T | A | V | - | - | - | - | - | - | - | - | - | - | - | F | - | - | - | - | - | E | G | D | - | - | - | - | - | - | L | V | F | L | S | L | P | - | - | A | G | V |
| 106 UniRef90\_A0A1A7MGQ0\_54\_442 | G | V | I | S | D | L | T | V | L | E | G | V | - | - | - | - | - | - | - | - | - | L | S | G | - | - | - | - | - | T | S | N | - | - | - | - | - | - | H | I | F | A | I | A | P | - | - | T | S | Q |
| 107 UniRef90\_UPI000413001D\_18\_392 | A | S | L | A | D | R | I | T | L | R | A | Q | I | - | - | - | - | - | - | - | - | - | - | - | - | - | - | - | - | G | G | G | - | - | - | - | - | - | F | A | V | L | T | V | P | - | - | G | D | R |
| 108 UniRef90\_G7H5P9\_11\_400 | A | S | V | A | D | W | L | S | V | T | A | V | - | - | - | - | - | - | - | - | - | - | - | F | - | - | - | - | - | E | G | D | - | - | - | - | - | - | L | V | F | L | A | L | P | - | - | R | D | V |
| 109 UniRef90\_A0A010YHQ5\_11\_404 | A | E | V | S | D | R | V | I | A | S | G | T | - | - | - | - | - | - | - | - | - | D | T | V | - | - | - | - | - | T | G | Q | - | - | - | - | - | - | K | Y | G | F | A | L | P | - | - | T | R | T |
| 110 UniRef90\_B0RBU7\_24\_404 | A | S | A | G | D | V | I | L | V | N | A | V | V | A | - | - | - | - | - | - | G | G | E | L | - | - | - | - | - | D | G | R | - | - | - | - | - | - | I | V | V | F | A | L | D | - | - | H | D | R |
| 111 UniRef90\_A0A167RW81\_42\_429 | G | V | V | S | D | L | T | V | L | E | G | V | - | - | - | - | - | - | - | - | - | L | A | G | - | - | - | - | - | T | N | D | - | - | - | - | - | - | H | I | F | A | I | V | P | - | - | T | Q | Q |
| 112 UniRef90\_A0A1X0J8A0\_26\_405 | V | A | L | A | D | L | I | T | V | L | L | Y | - | - | - | - | - | - | - | - | - | E | - | - | - | - | - | - | - | - | D | E | - | - | - | - | - | - | P | I | N | A | V | I | P | - | - | R | D | R |
| 113 UniRef90\_A0A2J6Q859\_43\_430 | G | V | V | S | D | V | T | V | L | E | G | V | - | - | - | - | - | - | - | - | - | L | D | G | - | - | - | - | - | T | Q | D | - | - | - | - | - | - | H | I | F | T | I | V | K | - | - | T | Q | Q |
| 114 UniRef90\_UPI0003828F02\_7\_389 | A | S | V | A | D | R | L | V | V | S | G | Q | - | - | - | - | - | - | - | - | - | H | A | T | - | - | - | - | - | T | G | R | - | - | - | - | - | - | K | L | T | F | T | L | D | - | - | A | R | A |
| 115 UniRef90\_A0A1B2HDJ6\_6\_361 | A | S | V | A | D | R | L | V | V | S | A | T | - | - | - | - | - | - | - | - | - | R | T | D | - | - | - | - | - | N | G | E | - | - | - | - | - | - | K | L | T | F | L | L | D | - | - | G | K | A |
| 116 UniRef90\_A0A2N3N0U1\_47\_427 | G | V | I | S | D | L | T | V | L | E | G | V | - | - | - | - | - | - | - | - | - | L | E | G | - | - | - | - | - | T | E | N | - | - | - | - | - | - | H | I | F | A | F | V | K | - | - | S | D | Q |
| 117 UniRef90\_S5Y4X6\_32\_408 | T | A | I | S | Q | I | I | T | V | S | L | W | - | - | - | - | - | - | - | - | - | D | K | - | - | - | - | - | - | - | G | R | - | - | - | - | - | - | R | V | H | Y | Q | I | P | - | - | T | D | R |
| 118 UniRef90\_A0A3R2WQN2\_17\_411 | S | K | V | S | D | L | T | V | L | E | G | V | - | - | - | - | - | - | - | - | - | L | Q | G | - | - | - | - | - | T | D | K | - | - | - | - | - | - | H | I | F | A | I | V | P | - | - | S | K | Q |
| 119 UniRef90\_A0A3N1X255\_20\_392 | T | H | V | A | D | V | I | Q | V | S | W | D | - | - | - | - | - | - | - | - | - | - | - | G | - | - | - | - | - | D | G | G | - | - | - | - | - | - | R | Y | T | A | V | I | P | - | - | A | D | R |
| 120 UniRef90\_A0A1S1LA29\_28\_404 | V | A | L | A | D | L | I | T | V | L | L | Y | - | - | - | - | - | - | - | - | - | E | - | - | - | - | - | - | - | - | A | E | - | - | - | - | - | - | P | I | N | A | I | I | P | - | - | G | D | R |
| 121 UniRef90\_A0A081GNL4\_19\_392 | S | V | D | S | D | V | I | P | T | T | A | I | - | - | - | - | - | - | - | - | - | L | E | D | - | - | - | - | - | T | G | E | - | - | - | - | - | - | V | V | I | V | I | L | P | - | - | T | A | S |
| 122 UniRef90\_A0A1I6U758\_27\_419 | A | K | I | A | D | L | L | V | V | E | G | T | - | - | - | - | - | - | - | - | - | L | E | P | - | - | - | - | - | G | D | Q | - | - | - | - | - | - | S | V | F | A | V | V | P | - | - | A | R | Q |
| 123 UniRef90\_A0A0F5VZP9\_7\_388 | A | L | F | A | D | W | I | P | V | L | A | R | - | - | - | - | - | - | - | - | - | A | E | E | - | - | - | - | - | - | D | K | - | - | - | - | - | - | L | H | V | A | Y | V | P | - | - | R | D | A |
| 124 UniRef90\_A0A1H4GIM8\_20\_411 | G | Q | I | S | D | L | T | V | L | E | G | V | - | - | - | - | - | - | - | - | - | L | E | G | - | - | - | - | - | A | E | T | - | - | - | - | - | - | H | I | F | A | I | V | P | - | - | T | A | Q |
| 125 UniRef90\_A0A0M8TTT1\_8\_388 | A | L | F | A | D | W | I | P | V | L | A | R | - | - | - | - | - | - | - | - | - | A | E | D | - | - | - | - | - | - | D | N | - | - | - | - | - | - | L | H | V | A | Y | V | P | - | - | R | G | A |
| 126 UniRef90\_A0A1Y2MDA3\_48\_437 | G | V | I | S | D | L | T | V | L | E | G | D | - | - | - | - | - | - | - | - | - | F | N | G | - | - | - | - | - | T | G | D | - | - | - | - | - | - | H | I | F | A | F | V | P | - | - | T | D | Q |
| 127 UniRef90\_UPI000562F0C5\_9\_388 | A | L | F | A | D | W | I | P | V | L | A | R | - | - | - | - | - | - | - | - | - | A | E | D | - | - | - | - | - | - | D | T | - | - | - | - | - | - | L | H | V | A | C | V | P | - | - | R | D | A |
| 128 UniRef90\_A0A506Y8Z1\_43\_442 | G | V | I | S | D | L | T | V | L | E | G | V | - | - | - | - | - | - | - | - | - | I | E | G | - | - | - | - | - | S | D | A | D | G | G | P | G | I | H | V | F | A | I | V | P | - | - | T | A | Q |
| 129 UniRef90\_A0A021VVW0\_28\_409 | S | K | V | S | D | V | T | W | L | E | A | A | - | - | - | - | - | - | - | - | - | V | D | G | - | - | - | - | - | H | E | D | - | - | - | - | - | - | H | V | F | A | L | V | A | - | - | S | D | D |
| 130 UniRef90\_E3QTS2\_46\_428 | G | V | I | S | D | L | T | V | L | E | G | V | - | - | - | - | - | - | - | - | - | L | E | G | - | - | - | - | - | T | E | D | - | - | - | - | - | - | H | I | Y | A | I | V | K | - | - | T | D | Q |
| 131 UniRef90\_A0A1D8SMV7\_7\_388 | A | L | F | A | D | W | I | P | V | L | A | R | - | - | - | - | - | - | - | - | - | T | E | D | - | - | - | - | - | - | D | N | - | - | - | - | - | - | L | H | V | A | Y | V | P | - | - | R | D | A |
| 132 UniRef90\_A9BUV8\_38\_419 | A | L | F | A | H | W | I | P | T | L | V | S | - | - | - | - | - | - | - | - | - | A | Q | E | E | A | P | G | Q | G | E | R | - | - | - | - | - | - | M | Y | L | V | F | I | P | - | - | R | D | A |
| 133 UniRef90\_A0A1N6TB45\_32\_407 | S | H | V | A | D | W | L | S | I | A | W | E | - | - | - | - | - | - | - | - | - | D | A | A | - | - | - | - | - | T | G | E | - | - | - | - | - | - | R | R | T | A | A | I | E | - | - | A | G | R |
| 134 UniRef90\_A0A381IE41\_24\_406 | A | L | F | A | H | Y | V | P | V | L | G | I | - | - | - | - | - | - | - | - | - | D | D | D | - | - | - | - | - | - | R | K | - | - | - | - | - | - | G | W | L | A | Y | I | P | - | - | K | G | T |
| 135 UniRef90\_A0A3M8TJ33\_18\_397 | V | L | V | A | D | R | L | A | V | R | A | D | - | - | - | - | - | - | - | - | - | H | S | E | - | - | - | - | - | T | G | E | - | - | - | - | - | - | P | L | A | V | L | V | D | - | - | A | R | R |
| 136 UniRef90\_A0A1H3JL41\_4\_373 | A | A | V | A | D | R | L | V | A | S | A | P | - | - | - | - | - | - | - | - | - | D | G | - | - | - | - | - | - | S | G | E | - | - | - | - | - | - | I | R | T | F | L | V | D | - | - | A | R | A |
| 137 UniRef90\_A0A507AW89\_49\_427 | G | V | V | S | D | L | T | V | L | E | G | S | - | - | - | - | - | - | - | - | - | Y | E | D | - | - | - | - | - | T | N | D | - | - | - | - | - | - | H | I | F | A | F | V | K | - | - | T | E | Q |
| 138 UniRef90\_A0A1X1SEN2\_20\_406 | A | A | V | S | D | V | L | L | A | W | V | Q | F | G | T | G | S | V | L | N | G | A | D | V | - | - | - | - | - | S | G | Q | - | - | - | - | - | - | L | A | T | V | H | I | E | - | - | G | G | A |
| 139 UniRef90\_A0A2D3UFX4\_26\_403 | V | L | V | A | D | R | L | A | V | R | A | V | - | - | - | - | - | - | - | - | - | R | A | D | - | - | - | - | - | T | G | E | - | - | - | - | - | - | P | L | A | V | V | V | D | - | - | P | T | R |
| 140 UniRef90\_A0A089X0S9\_26\_403 | V | L | V | A | D | R | V | A | V | R | A | V | - | - | - | - | - | - | - | - | - | R | A | D | - | - | - | - | - | S | A | E | - | - | - | - | - | - | P | V | A | V | L | V | D | - | - | P | A | A |
| 141 UniRef90\_A0A2W5T1D0\_29\_413 | G | V | V | S | D | L | T | V | L | E | G | V | - | - | - | - | - | - | - | - | - | L | E | G | - | - | - | - | - | T | D | T | - | - | - | - | - | - | H | V | F | A | I | V | P | - | - | T | D | Q |
| 142 UniRef90\_A0A1C4QGR9\_7\_388 | A | L | F | A | D | W | I | P | V | L | A | R | - | - | - | - | - | - | - | - | - | A | A | D | - | - | - | - | - | - | D | A | - | - | - | - | - | - | L | H | V | A | Y | V | P | - | - | R | D | A |
| 143 UniRef90\_A0A101R4L6\_7\_388 | A | L | F | A | D | W | I | P | V | L | A | R | - | - | - | - | - | - | - | - | - | A | D | D | - | - | - | - | - | - | D | D | - | - | - | - | - | - | L | H | V | A | Y | V | P | - | - | R | D | A |
| 144 UniRef90\_A0A4D4LHU8\_7\_388 | A | L | F | A | D | W | I | P | V | L | A | R | - | - | - | - | - | - | - | - | - | A | E | N | - | - | - | - | - | - | D | D | - | - | - | - | - | - | L | H | V | A | Y | V | P | - | - | R | D | A |
| 145 UniRef90\_L1KK35\_6\_389 | A | L | F | A | H | W | I | P | V | L | A | R | - | - | - | - | - | - | - | - | - | T | E | D | - | - | - | - | - | - | D | T | - | - | - | - | - | - | L | H | V | A | Y | V | P | - | - | R | D | A |
| 146 UniRef90\_UPI0005601C6B\_13\_390 | A | L | F | A | H | W | I | P | V | L | A | R | - | - | - | - | - | - | - | - | - | A | A | D | - | - | - | - | - | - | D | A | - | - | - | - | - | - | L | H | V | A | Y | V | A | - | - | A | D | T |
| 147 UniRef90\_A0A1H9UQJ9\_6\_361 | A | A | V | A | D | R | L | V | V | S | A | T | - | - | - | - | - | - | - | - | - | R | T | D | - | - | - | - | - | N | G | E | - | - | - | - | - | - | K | L | T | F | L | L | D | - | - | G | K | A |
| 148 UniRef90\_A0A3D9JKY9\_12\_401 | A | G | I | A | D | R | L | V | V | R | A | V | - | - | - | - | - | - | - | - | - | G | Y | G | - | - | - | - | - | T | G | E | - | - | - | - | - | - | P | L | A | V | L | V | D | - | - | P | T | R |
| 149 UniRef90\_D9X7Q2\_26\_403 | A | L | V | A | D | R | L | T | V | R | A | V | - | - | - | - | - | - | - | - | - | R | A | D | - | - | - | - | - | T | G | E | - | - | - | - | - | - | P | L | A | L | V | V | D | - | - | P | A | L |
| 150 UniRef90\_A0A1X1DXJ3\_20\_398 | A | L | F | A | D | I | L | V | T | T | A | L | - | - | - | - | - | - | - | - | - | Q | D | D | - | - | - | - | - | - | D | R | - | - | - | - | - | - | P | V | M | A | F | I | P | - | - | L | P | A |

  
  

|  |  |  |  |  |  |  |  |  |  |  |  |  |  |  |  |  |  |  |  |  |  |  |  |  |  |  |  |  |  |  |  |  |  |  |  |  |  |  |  |  |  |  |  |  |  |  |  |  |  |  |
| --- | --- | --- | --- | --- | --- | --- | --- | --- | --- | --- | --- | --- | --- | --- | --- | --- | --- | --- | --- | --- | --- | --- | --- | --- | --- | --- | --- | --- | --- | --- | --- | --- | --- | --- | --- | --- | --- | --- | --- | --- | --- | --- | --- | --- | --- | --- | --- | --- | --- | --- |
| **001 Input\_pdb\_SEQRES\_A** | A | G | V | T | P | N | D | D | - | W | A | A | I | G | M | R | Q | T | D | S | G | S | T | D | F | H | N | V | K | V | E | P | D | E | V | L | G | A | P | - | - | - | - | N | - | - | - | - | - | - |
| 002 UniRef90\_Q1W1G3\_1\_416 | H | G | V | Q | V | N | D | D | - | W | A | A | I | G | M | R | Q | T | D | S | G | S | T | D | F | H | S | V | K | V | E | P | D | E | V | L | G | E | P | - | - | - | - | N | - | - | - | - | - | - |
| 003 UniRef90\_T0BM21\_7\_392 | E | G | V | L | V | H | D | D | - | W | D | G | L | G | Q | R | Q | T | D | S | G | T | V | E | F | Q | D | V | V | V | E | P | H | E | I | I | A | V | P | - | - | - | - | L | - | - | - | - | - | - |
| 004 UniRef90\_UPI0002AC58FE\_11\_391 | Q | G | I | N | I | Q | D | D | - | W | D | N | M | G | Q | R | Q | T | D | S | G | S | I | V | F | D | N | V | V | V | Y | E | D | E | I | L | A | L | R | - | - | - | - | D | - | - | - | - | - | - |
| 005 UniRef90\_A0A3D5CW51\_20\_401 | T | G | V | T | I | L | D | D | - | W | D | N | I | G | Q | R | Q | T | D | S | G | S | V | N | F | E | K | V | R | V | E | E | N | E | L | L | T | E | P | - | - | - | - | G | - | - | - | - | - | - |
| 006 UniRef90\_A0A2V4SPP5\_16\_400 | A | G | V | H | V | E | A | D | - | W | D | A | F | G | Q | R | Q | T | D | S | G | N | V | H | F | E | K | V | Y | L | P | T | A | L | V | L | Q | A | P | - | - | - | - | G | - | - | - | - | - | - |
| 007 UniRef90\_E0UIV5\_8\_393 | E | G | I | Q | I | N | D | D | - | W | D | N | I | G | Q | R | Q | T | D | S | G | S | I | S | F | N | N | V | L | V | Y | P | D | E | I | F | K | N | K | - | - | - | - | E | - | - | - | - | - | - |
| 008 UniRef90\_A0A1Z4S897\_9\_391 | E | G | L | Q | I | N | D | D | - | W | N | Y | I | G | Q | R | Q | T | E | S | G | S | V | V | F | H | N | V | L | I | R | R | D | E | I | L | G | N | P | - | - | - | - | D | - | - | - | - | - | - |
| 009 UniRef90\_UPI00045E9273\_21\_396 | P | G | I | R | I | H | S | D | - | W | D | N | M | G | Q | R | Q | T | D | S | G | T | V | D | F | D | R | V | R | I | Y | D | Q | E | I | L | S | Q | P | - | - | - | - | G | - | - | - | - | - | - |
| 010 UniRef90\_A0A353Y4M9\_17\_384 | E | G | V | Q | I | E | G | D | - | W | N | A | I | G | Q | R | Q | T | D | S | G | N | V | I | F | T | D | V | L | V | E | P | H | E | L | L | Q | - | P | - | - | - | - | G | - | - | - | - | - | - |
| 011 UniRef90\_F3KR10\_17\_384 | E | G | L | Q | I | Q | G | D | - | W | N | A | I | G | Q | R | Q | T | D | S | G | N | V | V | F | E | D | V | L | V | K | P | E | E | V | L | N | - | P | - | - | - | - | G | - | - | - | - | - | - |
| 012 UniRef90\_A0A2D8NW56\_20\_404 | R | G | I | T | V | H | D | D | - | W | D | A | M | G | Q | R | Q | T | D | S | G | T | V | T | F | D | S | V | V | V | E | P | E | E | V | L | I | A | P | - | - | - | - | G | - | - | - | - | - | - |
| 013 UniRef90\_A0A2W7M9P1\_16\_386 | E | G | V | I | I | G | N | D | - | W | D | S | F | G | Q | R | Q | T | D | S | G | S | V | E | F | H | N | V | E | V | K | H | S | E | V | L | E | A | F | - | - | - | - | Q | - | I | - | - | - | - |
| 014 UniRef90\_A0A398AYR9\_12\_386 | G | G | V | L | L | G | H | D | - | W | D | S | F | G | Q | R | Q | T | D | S | G | S | V | T | F | E | Q | V | I | V | H | D | A | E | V | L | D | A | Y | - | - | - | - | E | - | A | - | - | - | - |
| 015 UniRef90\_A0A0T6UXN9\_11\_394 | T | G | I | T | L | H | G | D | - | W | D | N | I | G | Q | R | Q | T | D | S | G | S | A | T | F | E | R | V | R | V | E | H | A | E | L | L | L | D | P | - | - | - | - | G | - | - | - | - | - | - |
| 016 UniRef90\_A0A252E884\_11\_390 | P | G | V | V | S | N | Q | D | - | W | D | N | I | G | Q | R | R | T | D | S | D | T | F | T | F | H | D | V | L | V | K | K | D | E | I | L | G | Y | P | - | - | - | - | H | - | - | - | - | - | - |
| 017 UniRef90\_A0A1Z4BZ71\_24\_403 | A | G | I | Y | V | H | D | D | - | W | D | N | M | G | Q | R | Q | T | D | S | G | S | V | E | F | K | G | L | R | V | E | K | Q | E | L | L | I | N | P | - | - | - | - | G | - | - | - | - | - | - |
| 018 UniRef90\_A0A1Z4IGC5\_10\_387 | H | G | I | T | V | H | D | D | - | W | D | N | I | G | Q | R | Q | T | D | S | G | S | V | S | F | A | N | V | L | V | K | E | F | E | I | L | A | N | P | - | - | - | - | E | - | - | - | - | - | - |
| 019 UniRef90\_A0A1W9JD13\_18\_401 | T | G | V | C | V | L | G | D | - | W | A | N | I | G | Q | R | Q | T | D | S | G | S | V | N | F | E | K | V | R | V | E | E | N | E | L | L | N | D | P | - | - | - | - | G | - | - | - | - | - | - |
| 020 UniRef90\_A0A0B6S5D2\_8\_393 | D | G | I | T | V | H | D | D | - | W | D | P | I | G | Q | R | Q | T | D | S | G | S | V | S | F | N | G | V | R | V | E | P | D | E | V | L | H | R | S | - | - | - | - | E | - | - | - | - | - | - |
| 021 UniRef90\_A0A1Y3C786\_14\_396 | S | G | I | T | L | Y | H | D | - | W | D | N | I | G | Q | R | Q | T | D | S | G | S | S | I | F | E | R | V | R | V | E | A | K | D | M | L | L | D | P | - | - | - | - | G | - | - | - | - | - | - |
| 022 UniRef90\_A0A318KD41\_20\_397 | E | G | I | T | L | F | H | D | - | W | D | N | M | G | Q | R | Q | T | D | S | G | S | A | D | F | K | Q | V | K | V | A | H | N | E | L | L | L | D | P | - | - | - | - | G | - | - | - | - | - | - |
| 023 UniRef90\_A0A352JDP6\_15\_389 | E | G | V | T | I | H | D | D | - | W | D | N | I | G | Q | R | Q | T | D | S | G | S | I | S | F | N | Q | V | E | V | F | P | H | E | I | F | G | N | R | - | - | - | - | S | - | - | - | - | - | - |
| 024 UniRef90\_A0A329B538\_13\_401 | S | G | I | T | L | L | D | D | - | W | D | S | I | G | Q | R | Q | T | D | S | G | T | V | Q | F | E | Q | V | R | V | E | N | S | E | I | L | A | D | P | - | - | - | - | G | - | - | - | - | - | - |
| 025 UniRef90\_A0A2N8QAF3\_13\_394 | E | G | I | T | V | H | E | D | - | W | N | P | I | G | Q | R | Q | T | D | S | G | S | V | S | F | A | S | V | R | V | E | P | H | E | V | L | A | R | A | - | - | - | - | D | - | - | - | - | - | - |
| 026 UniRef90\_A0A2N7XWT8\_11\_394 | S | G | I | I | L | H | N | D | - | W | N | N | M | G | Q | R | Q | T | D | S | G | S | V | T | F | K | R | V | R | V | E | E | S | D | L | L | L | D | P | - | - | - | - | G | - | - | - | - | - | - |
| 027 UniRef90\_A0A2A4HLB2\_33\_412 | P | G | I | T | V | H | D | D | - | W | D | A | I | G | Q | R | Q | T | D | S | G | T | V | S | F | E | Q | V | E | V | L | E | S | E | V | L | L | P | P | - | - | - | - | G | - | - | - | - | - | - |
| 028 UniRef90\_A0A4R3HWL3\_41\_416 | A | G | I | H | V | L | D | D | - | W | D | N | L | G | Q | R | Q | T | D | S | G | S | V | I | F | D | N | V | R | V | E | R | D | E | V | L | G | P | P | - | - | - | - | G | - | - | - | - | - | - |
| 029 UniRef90\_A0A1M7NXP6\_14\_394 | H | G | V | S | V | Q | A | D | - | W | D | A | F | G | Q | K | Q | T | D | S | G | N | V | H | F | N | Q | V | S | L | P | S | T | Q | V | L | Q | A | P | - | - | - | - | G | - | - | - | - | - | - |
| 030 UniRef90\_A0A1B4ESK2\_22\_397 | D | G | I | T | V | N | D | D | - | W | D | P | V | G | Q | R | Q | T | D | S | G | S | V | R | F | D | G | V | T | L | A | P | D | D | V | L | H | R | S | - | - | - | - | E | - | - | - | - | - | - |
| 031 UniRef90\_A0A1H6NKV2\_11\_394 | S | G | I | T | L | H | N | D | - | W | H | N | M | G | Q | R | Q | T | D | S | G | S | A | S | F | E | R | V | R | V | E | E | S | E | L | L | L | E | P | - | - | - | - | G | - | - | - | - | - | - |
| 032 UniRef90\_A0A0D0KUF8\_11\_394 | T | G | I | T | L | H | D | D | - | W | N | N | M | G | Q | R | Q | T | D | S | G | S | A | T | F | E | R | V | R | V | E | E | E | E | L | L | L | D | P | - | - | - | - | G | - | - | - | - | - | - |
| 033 UniRef90\_A0A0F3K7B4\_20\_401 | S | G | I | S | V | Q | Q | D | - | W | N | N | I | G | Q | R | Q | T | D | S | G | S | V | N | F | E | K | V | R | I | E | N | N | E | V | L | D | N | P | - | - | - | - | G | - | - | - | - | - | - |
| 034 UniRef90\_A0A381IMG6\_13\_394 | E | G | I | V | V | R | D | D | - | W | D | P | I | G | Q | R | Q | T | D | S | G | S | V | S | F | D | G | V | R | V | E | P | D | E | V | L | H | C | S | - | - | - | - | E | - | - | - | - | - | - |
| 035 UniRef90\_A0A2S9K1G1\_26\_400 | A | G | V | T | I | R | A | D | - | W | D | A | F | G | Q | K | Q | T | D | S | G | T | V | T | F | D | Q | V | R | L | E | P | H | E | V | L | V | Q | P | - | - | - | - | G | - | - | - | - | - | - |
| 036 UniRef90\_A0A1W6L7W5\_17\_392 | D | G | V | A | V | Q | G | D | - | W | D | A | F | G | Q | R | Q | T | D | S | G | T | V | T | F | D | Q | V | A | L | A | H | A | E | V | L | Q | A | P | - | - | - | - | G | - | - | - | - | - | - |
| 037 UniRef90\_A0A255HJE3\_14\_398 | P | G | I | T | V | Q | P | D | - | W | D | A | F | G | Q | R | Q | T | D | S | G | N | V | H | F | E | Q | V | A | L | P | A | E | L | V | L | Q | S | P | - | - | - | - | A | - | - | - | - | - | - |
| 038 UniRef90\_A0A0R3AD45\_11\_394 | S | G | I | T | L | H | N | D | - | W | N | N | I | G | Q | R | Q | T | D | S | G | S | A | S | F | E | R | V | R | V | E | E | S | E | L | L | L | D | P | - | - | - | - | G | - | - | - | - | - | - |
| 039 UniRef90\_A0A1W6ZB60\_16\_389 | D | G | I | T | V | N | P | D | - | W | D | A | F | G | Q | R | Q | T | D | S | G | T | V | R | F | D | H | V | R | L | D | P | V | Q | I | L | Q | A | P | - | - | - | - | G | - | - | - | - | - | - |
| 040 UniRef90\_A0A2X1DPB7\_14\_397 | A | G | I | T | V | N | D | D | - | W | D | P | V | G | Q | R | Q | T | D | S | G | S | V | R | F | D | G | V | T | L | A | A | D | E | V | L | H | R | S | - | - | - | - | E | - | - | - | - | - | - |
| 041 UniRef90\_A0A0S9M2D1\_4\_380 | S | G | I | Q | A | A | P | D | - | W | D | N | M | G | Q | R | Q | T | D | S | G | S | V | T | F | E | K | V | R | V | E | D | A | E | L | L | T | D | P | - | - | - | - | G | - | - | - | - | - | - |
| 042 UniRef90\_A0A238ZKW1\_13\_401 | T | G | I | N | V | L | Q | D | - | W | H | N | I | G | Q | R | Q | T | D | S | G | S | V | N | F | E | R | V | R | V | E | K | D | D | L | L | Q | E | P | - | - | - | - | G | - | - | - | - | - | - |
| 043 UniRef90\_A0A0D1P8V6\_10\_394 | T | G | I | T | L | H | D | D | - | W | H | N | M | G | Q | R | Q | T | D | S | G | S | A | T | F | E | R | V | R | V | E | E | G | D | L | L | L | D | P | - | - | - | - | G | - | - | - | - | - | - |
| 044 UniRef90\_A0A261SP68\_11\_387 | Q | G | V | T | V | N | P | D | - | W | D | A | F | G | Q | R | Q | T | D | S | G | T | V | R | F | D | H | V | R | L | E | F | L | Q | V | L | Q | A | P | - | - | - | - | G | - | - | - | - | - | - |
| 045 UniRef90\_A0A2N6MRB2\_11\_390 | T | G | V | V | S | E | Q | D | - | W | D | N | I | G | Q | R | R | T | D | S | D | T | F | T | F | H | N | V | L | V | K | K | D | E | I | L | G | Y | P | - | - | - | - | H | - | - | - | - | - | - |
| 046 UniRef90\_A0A212BVA1\_31\_405 | E | G | L | A | I | N | D | D | - | W | D | A | F | G | Q | R | Q | T | D | S | G | T | V | Q | F | E | N | V | F | V | D | R | G | E | L | L | V | S | G | - | - | - | - | G | - | - | - | - | - | - |
| 047 UniRef90\_A0A1A9KE82\_11\_394 | S | G | I | G | V | H | D | D | - | W | D | N | M | G | Q | R | Q | T | D | S | G | S | V | T | F | E | R | V | R | V | E | R | S | E | L | L | L | D | P | - | - | - | - | G | - | - | - | - | - | - |
| 048 UniRef90\_A0A4Q5PVB9\_26\_400 | D | G | I | T | V | N | D | D | - | W | D | N | M | G | Q | R | Q | T | D | S | G | S | V | T | F | S | N | V | F | V | G | E | A | E | I | L | G | P | P | - | - | - | - | G | - | - | - | - | - | - |
| 049 UniRef90\_A0A178GPM9\_9\_392 | K | G | V | S | F | L | G | D | - | W | N | N | M | G | Q | R | Q | T | D | S | G | T | S | H | F | E | Q | V | K | I | H | Q | N | E | L | L | L | N | P | - | - | - | - | G | - | - | - | - | - | - |
| 050 UniRef90\_A0A1P9YC32\_3\_390 | A | G | I | A | V | L | D | D | - | W | D | N | M | G | Q | R | Q | T | D | S | G | T | V | V | F | E | R | V | P | I | A | K | D | E | L | L | L | Q | P | - | - | - | - | G | - | - | - | - | - | - |
| 051 UniRef90\_UPI000A1773A9\_40\_416 | S | G | I | A | V | I | Q | D | - | W | D | N | I | G | Q | R | Q | T | D | S | G | S | V | D | F | E | R | V | R | V | D | D | A | E | L | L | L | D | P | - | - | - | - | G | - | - | - | - | - | - |
| 052 UniRef90\_A0A158L201\_25\_395 | A | G | I | T | V | H | D | D | - | W | D | P | V | G | Q | R | Q | T | D | S | G | S | V | S | F | D | D | V | E | L | R | D | D | E | V | L | H | R | S | - | - | - | - | E | - | - | - | - | - | - |
| 053 UniRef90\_A0A4Q4GT15\_13\_391 | E | G | V | S | F | L | G | D | - | W | N | N | M | G | Q | R | Q | T | D | S | G | T | S | H | F | E | Q | V | K | I | S | K | D | E | L | L | L | N | P | - | - | - | - | G | - | - | - | - | - | - |
| 054 UniRef90\_A0A1C0YC62\_17\_381 | A | G | V | H | I | H | N | D | - | W | D | S | F | G | Q | R | Q | T | D | S | G | A | V | T | F | N | D | V | S | I | L | P | H | E | C | L | A | L | H | - | - | - | - | T | - | - | - | - | - | - |
| 055 UniRef90\_A0A1H7FE04\_32\_405 | Q | G | V | A | V | E | Q | D | - | W | D | A | F | G | Q | R | Q | T | D | S | G | T | V | R | F | D | N | V | S | L | A | D | E | E | L | L | Q | V | P | - | - | - | - | G | - | - | - | - | - | - |
| 056 UniRef90\_A0A401MWC4\_5\_385 | P | G | V | V | R | N | D | D | - | W | D | N | F | G | Q | R | L | S | A | S | G | S | V | E | F | D | A | V | P | V | A | A | D | D | V | F | G | S | L | - | - | - | - | A | - | - | - | - | - | - |
| 057 UniRef90\_A0A1G6HFW7\_16\_395 | K | G | I | G | V | L | D | D | - | W | D | N | M | G | Q | R | Q | T | D | S | G | T | V | V | F | D | Q | V | A | V | A | G | N | E | V | L | A | D | P | - | - | - | - | G | - | - | - | - | - | - |
| 058 UniRef90\_A0A0P9B999\_30\_403 | A | G | I | T | V | L | G | D | - | W | D | N | M | G | Q | R | Q | T | D | S | G | N | V | E | F | R | R | V | R | I | A | H | D | E | I | L | G | P | P | - | - | - | - | G | - | - | - | - | - | - |
| 059 UniRef90\_A0A140K6F0\_14\_392 | E | G | I | S | Y | N | Y | D | - | W | N | N | M | G | Q | R | C | T | A | S | G | S | Y | T | F | N | N | V | Q | V | F | P | K | E | I | V | G | P | P | - | - | - | - | P | - | - | - | - | - | - |
| 060 UniRef90\_A0A1P8EKI7\_5\_392 | D | G | I | R | F | L | G | D | - | W | N | N | M | G | Q | R | Q | T | D | S | G | T | T | H | F | E | Q | V | K | I | L | K | H | E | L | L | L | N | P | - | - | - | - | G | - | - | - | - | - | - |
| 061 UniRef90\_A0A2U3MYZ3\_7\_392 | D | G | V | S | F | L | G | D | - | W | N | N | M | G | Q | R | Q | T | D | S | G | T | S | H | F | E | Q | V | K | I | Y | K | D | E | L | L | L | N | P | - | - | - | - | G | - | - | - | - | - | - |
| 062 UniRef90\_A0A1H2EPX5\_7\_397 | D | G | V | R | V | L | A | D | - | W | D | A | F | G | Q | R | Q | T | D | S | G | A | V | L | F | E | N | V | L | L | R | D | D | E | I | L | R | T | P | - | - | - | - | G | - | - | - | - | - | - |
| 063 UniRef90\_UPI00041EF43F\_25\_400 | P | G | I | S | I | Q | G | D | - | W | D | A | F | G | Q | K | Q | T | D | S | G | T | V | T | F | N | Q | V | R | V | Q | A | H | Q | V | L | V | R | P | - | - | - | - | G | - | - | - | - | - | - |
| 064 UniRef90\_D0IW93\_17\_384 | P | G | I | A | V | K | D | D | - | W | N | P | M | G | Q | R | Q | T | D | S | G | S | V | Q | F | N | R | V | Q | L | P | A | S | A | V | M | R | D | E | - | - | - | - | S | - | - | - | - | - | - |
| 065 UniRef90\_A0A395D1I6\_11\_388 | D | G | V | L | V | H | G | D | - | W | D | N | M | G | Q | R | Q | T | D | S | G | T | V | S | F | D | N | V | F | V | A | E | S | E | L | L | L | S | P | - | - | - | - | G | - | - | - | - | - | - |
| 066 UniRef90\_A0A1H0TK45\_27\_400 | A | G | I | R | V | L | N | D | - | W | D | N | M | G | Q | R | Q | T | D | S | G | S | V | I | F | D | Q | V | A | V | A | A | D | E | L | L | T | D | P | - | - | - | - | G | - | - | - | - | - | - |
| 067 UniRef90\_A0A1H8MYU5\_23\_403 | P | G | I | T | I | A | D | D | - | W | D | G | I | G | Q | R | Q | T | G | S | G | R | V | F | Y | K | D | V | R | V | R | D | D | E | I | L | S | E | - | - | - | - | - | - | - | - | - | - | - | - |
| 068 UniRef90\_A0A1B1M254\_5\_403 | E | G | L | T | F | L | D | D | - | W | D | N | I | G | Q | R | L | T | E | S | G | G | V | T | L | D | G | V | R | T | P | W | S | S | A | A | G | Y | V | - | - | - | - | D | - | - | - | - | - | - |
| 069 UniRef90\_A0A484THM3\_28\_401 | A | G | I | V | V | N | D | D | - | W | D | N | M | G | Q | R | Q | T | D | S | G | T | V | V | F | N | G | V | Q | V | G | L | D | E | A | L | G | P | P | - | - | - | - | G | - | - | - | - | - | - |
| 070 UniRef90\_A0A0Q8Q8X6\_8\_377 | H | G | I | S | I | L | D | D | - | W | D | P | I | G | Q | R | Q | T | D | S | N | S | V | S | F | A | N | V | P | L | S | G | G | N | L | L | R | R | H | - | - | - | - | D | - | - | - | - | - | - |
| 071 UniRef90\_A0A0Q5QDL2\_32\_410 | E | G | L | T | F | H | H | D | - | W | D | N | L | G | Q | R | L | T | E | S | G | S | V | T | I | D | N | V | T | T | E | W | A | S | A | A | G | F | V | - | - | - | - | D | - | - | - | - | - | - |
| 072 UniRef90\_A0A1A5XM62\_22\_406 | A | G | I | T | V | H | D | D | - | W | D | P | I | G | Q | R | Q | T | D | S | G | T | V | S | F | D | A | V | E | V | R | A | H | E | V | L | K | R | P | - | - | - | - | D | - | - | - | - | - | - |
| 073 UniRef90\_A0A239EI90\_21\_397 | E | G | L | T | F | H | D | D | - | W | D | N | I | G | Q | R | R | T | E | S | G | S | V | T | I | D | K | V | R | V | D | W | S | S | A | A | G | F | V | - | - | - | - | D | - | - | - | - | - | - |
| 074 UniRef90\_S5SWW2\_23\_403 | E | G | I | V | I | A | D | D | - | W | D | G | I | G | Q | R | Q | T | G | S | G | Q | V | F | Y | H | N | V | R | I | E | A | G | E | I | L | P | E | - | - | - | - | - | - | - | - | - | - | - | - |
| 075 UniRef90\_A0A3A5JJY9\_31\_413 | A | G | L | D | I | R | G | D | - | W | D | A | I | G | Q | R | Q | T | D | S | G | S | I | G | F | N | R | V | R | I | T | A | E | D | V | L | L | A | A | - | - | - | - | D | - | - | - | - | - | - |
| 076 UniRef90\_A0A315ZU85\_7\_384 | A | G | V | Q | P | G | G | D | - | W | D | N | L | G | Q | R | L | S | A | S | G | S | V | T | F | T | D | V | E | V | P | A | E | N | V | L | G | F | L | - | - | - | - | D | - | - | - | - | - | - |
| 077 UniRef90\_A0A3R9U899\_5\_381 | E | G | L | T | F | H | D | D | - | W | D | N | I | G | Q | R | L | T | E | S | G | S | V | T | L | D | G | V | R | T | P | W | S | S | A | A | G | Y | V | - | - | - | - | N | - | - | - | - | - | - |
| 078 UniRef90\_UPI000DD53D91\_7\_389 | D | G | I | V | I | E | N | D | - | W | D | G | I | G | Q | R | Q | T | G | S | G | T | V | R | F | V | N | L | E | I | D | D | D | E | L | I | G | S | P | - | - | - | - | D | - | - | - | - | - | - |
| 079 UniRef90\_UPI000DE4BE09\_13\_396 | A | G | Y | T | A | N | D | D | - | W | D | N | F | G | Q | R | L | S | D | S | G | S | V | E | F | H | D | F | P | V | Y | E | E | D | F | V | T | S | P | - | - | - | - | T | - | - | - | - | - | - |
| 080 UniRef90\_A0A2T0R7P7\_10\_381 | P | G | F | V | K | G | G | D | - | W | D | N | L | G | Q | R | L | S | A | S | G | S | V | R | F | D | D | V | R | I | T | P | D | A | V | L | G | S | A | - | - | - | - | S | - | - | - | - | - | - |
| 081 UniRef90\_A0A4R1HYS3\_27\_404 | D | G | I | V | F | G | D | D | - | W | D | N | I | G | Q | R | L | T | E | S | G | S | V | T | I | S | D | V | T | V | P | W | A | S | A | A | G | F | V | - | - | - | - | D | - | - | - | - | - | - |
| 082 UniRef90\_UPI000D1537B0\_7\_385 | E | G | V | Q | I | M | D | D | - | W | D | G | F | G | Q | T | Q | T | G | S | G | S | V | R | F | H | G | V | R | V | L | P | E | E | V | V | D | M | A | - | - | - | - | P | - | - | - | - | - | - |
| 083 UniRef90\_A0A1G6ZNM7\_17\_409 | D | G | I | V | F | A | G | D | - | W | D | N | L | G | Q | R | L | T | E | S | G | S | A | L | I | E | G | V | R | V | P | W | E | A | A | A | G | Y | V | A | T | E | Q | G | - | - | - | - | - | - |
| 084 UniRef90\_UPI0003765980\_18\_389 | P | G | I | V | I | V | D | D | - | W | D | G | I | G | Q | R | Q | T | G | S | G | T | V | H | F | H | D | L | R | I | D | D | D | E | M | I | S | A | P | - | - | - | - | S | - | - | - | - | - | - |
| 085 UniRef90\_A0A071ICK8\_25\_398 | P | G | Y | V | A | N | D | D | - | W | D | N | I | G | Q | R | L | S | D | S | G | T | V | E | F | R | D | F | P | V | Y | E | E | D | F | I | A | P | P | - | - | - | - | S | - | - | - | - | - | - |
| 086 UniRef90\_A0A2L0WNE5\_33\_396 | P | G | Y | I | A | N | D | D | - | W | D | N | F | G | Q | R | L | S | D | S | G | S | V | D | F | R | D | F | P | V | Y | T | E | D | F | V | G | Q | P | - | - | - | - | V | - | - | - | - | - | - |
| 087 UniRef90\_A0A1Q8CFJ2\_9\_380 | P | G | L | S | Y | L | H | D | - | W | D | N | L | G | Q | R | L | T | A | S | G | G | V | V | F | R | D | V | H | V | P | D | E | D | V | L | G | V | H | - | - | - | - | D | - | - | - | - | - | - |
| 088 UniRef90\_UPI000835F605\_21\_394 | K | G | I | S | F | E | G | D | - | W | D | N | L | G | Q | R | L | T | A | S | G | S | V | I | F | D | R | V | E | A | T | D | A | D | L | L | S | G | L | - | - | - | - | D | - | - | - | - | - | - |
| 089 UniRef90\_UPI000374AB86\_31\_398 | P | G | Y | V | A | N | D | D | - | W | D | N | I | G | Q | R | L | S | D | S | G | S | V | E | F | H | D | F | P | V | Y | N | E | D | F | I | G | A | P | - | - | - | - | V | - | - | - | - | - | - |
| 090 UniRef90\_A0A267RVL6\_22\_399 | P | G | I | V | F | N | D | D | - | W | D | N | L | G | Q | R | L | T | A | S | G | T | L | E | F | N | R | V | R | L | Q | E | K | D | R | L | T | G | L | - | - | - | - | E | - | - | - | - | - | - |
| 091 UniRef90\_M3VA40\_23\_395 | E | G | F | T | P | L | G | D | - | W | D | N | I | G | Q | R | L | T | D | S | G | G | V | E | F | V | N | T | P | I | S | R | D | E | I | L | G | - | D | - | - | - | - | P | - | - | - | - | - | - |
| 092 UniRef90\_A0A395GID9\_32\_425 | P | G | I | Q | F | A | H | N | - | W | H | N | I | G | L | R | L | T | E | S | G | G | V | K | I | E | N | V | R | V | P | W | A | D | A | L | G | W | D | - | - | - | - | T | - | - | - | - | - | - |
| 093 UniRef90\_UPI0008268C2F\_35\_406 | D | G | F | T | P | Q | G | D | - | W | D | N | I | G | Q | R | L | T | D | S | G | G | V | V | F | D | N | T | P | V | E | R | S | E | V | L | G | D | D | - | - | - | - | P | - | - | - | - | - | - |
| 094 UniRef90\_A0A0S1XV73\_27\_397 | A | G | V | V | I | E | D | D | - | W | D | G | I | G | Q | T | Q | T | G | S | G | T | V | S | F | H | G | V | R | V | R | D | D | E | V | L | G | D | A | - | - | - | - | G | - | - | - | - | - | - |
| 095 UniRef90\_A0A0F5N116\_19\_398 | Q | G | F | T | F | L | D | D | - | W | D | N | I | G | Q | R | L | T | D | S | G | G | V | R | I | V | D | A | R | I | E | H | R | E | V | L | G | E | E | - | - | - | - | P | - | - | - | - | - | - |
| 096 UniRef90\_A0A1N6Z7J9\_40\_406 | A | G | Y | I | A | Y | D | D | - | W | D | N | I | G | Q | R | L | S | D | S | G | S | V | E | F | K | D | Y | V | V | Y | E | S | D | F | L | F | P | L | - | - | - | - | V | - | - | - | - | - | - |
| 097 UniRef90\_A0A2V4UWQ2\_31\_411 | P | G | Y | I | A | H | D | D | - | W | D | N | I | G | Q | R | L | S | D | S | G | S | V | E | F | K | D | F | P | V | H | D | S | D | F | L | L | P | L | - | - | - | - | A | - | - | - | - | - | - |
| 098 UniRef90\_A0A4P8KLT1\_5\_401 | P | G | V | E | L | V | D | D | - | W | D | N | L | G | Q | R | L | S | A | S | N | T | V | T | Y | R | D | V | R | V | E | P | E | H | V | L | G | F | G | - | - | - | - | T | - | - | - | - | - | - |
| 099 UniRef90\_UPI0003068598\_3\_292 | E | G | I | A | V | R | D | D | - | W | D | P | I | G | Q | R | Q | T | D | S | G | S | V | A | F | D | G | V | R | V | A | P | D | E | V | L | H | R | S | - | - | - | - | E | - | - | - | - | - | - |
| 100 UniRef90\_UPI000DD585C3\_27\_403 | P | G | Y | V | A | H | D | D | - | W | D | N | I | G | Q | R | L | S | D | S | G | S | V | E | F | Q | D | F | P | V | Y | E | R | D | F | I | L | P | L | - | - | - | - | A | - | - | - | - | - | - |
| 101 UniRef90\_A0A1E3SMN8\_25\_404 | Q | G | F | T | F | L | D | D | - | W | D | N | I | G | Q | R | L | T | D | S | G | G | V | R | V | V | D | A | R | I | E | H | H | E | V | L | G | E | E | - | - | - | - | P | - | - | - | - | - | - |
| 102 UniRef90\_UPI00045EBD18\_12\_396 | E | G | V | T | H | G | N | D | - | W | D | A | F | G | Q | R | L | S | E | S | G | T | I | T | F | A | D | V | K | V | E | A | D | E | I | L | G | D | P | - | - | - | - | A | - | - | - | - | - | - |
| 103 UniRef90\_A0A1Y1ZNX6\_42\_434 | D | G | I | K | F | S | H | D | - | W | N | N | V | G | L | R | L | T | E | S | G | S | V | K | L | E | N | I | V | V | P | W | S | D | A | L | G | W | D | - | - | - | - | T | - | - | - | - | - | - |
| 104 UniRef90\_A0A0D2FPI6\_31\_423 | P | G | I | Q | F | G | H | D | - | W | D | N | I | G | M | R | L | T | E | S | G | S | V | K | I | E | N | V | R | V | P | W | T | D | A | F | G | W | D | - | - | - | - | S | - | - | - | - | - | - |
| 105 UniRef90\_A0A2S0KGS2\_30\_403 | E | G | F | T | P | L | G | D | - | W | D | N | I | G | Q | R | L | T | D | S | G | G | V | E | F | D | D | T | P | V | A | R | D | Q | I | L | G | S | D | - | - | - | - | P | - | - | - | - | - | - |
| 106 UniRef90\_A0A1A7MGQ0\_54\_442 | P | G | F | E | F | A | H | N | - | W | N | N | I | G | L | R | L | T | E | S | G | S | V | K | I | N | D | I | K | V | P | W | S | D | A | L | G | W | D | - | - | - | - | A | - | - | - | - | - | - |
| 107 UniRef90\_UPI000413001D\_18\_392 | A | G | V | T | H | G | N | D | - | W | D | A | F | G | Q | R | L | T | E | S | G | T | I | A | F | D | N | V | R | I | G | R | G | E | I | L | G | E | A | - | - | - | - | E | - | - | - | - | - | - |
| 108 UniRef90\_G7H5P9\_11\_400 | E | G | F | R | P | L | G | D | - | W | D | N | I | G | Q | R | L | T | D | S | G | G | V | E | F | V | N | T | P | V | S | R | D | Q | I | L | G | A | D | - | - | - | - | P | - | - | - | - | - | - |
| 109 UniRef90\_A0A010YHQ5\_11\_404 | A | G | V | V | H | G | E | D | - | W | D | S | L | G | Q | R | A | S | A | S | G | S | I | A | F | D | G | A | F | L | A | E | E | D | I | L | G | P | G | - | - | - | - | E | - | - | - | - | - | - |
| 110 UniRef90\_B0RBU7\_24\_404 | P | G | I | A | Y | L | G | D | - | W | D | A | L | G | Q | R | L | S | A | S | G | S | V | R | F | T | D | V | R | V | E | P | D | D | V | L | G | V | G | - | - | - | - | S | - | - | - | - | - | - |
| 111 UniRef90\_A0A167RW81\_42\_429 | P | G | I | Q | F | A | H | N | - | W | D | N | I | G | L | R | L | T | E | S | G | S | V | R | I | E | N | V | H | V | P | W | A | D | A | L | G | W | N | - | - | - | - | P | - | - | - | - | - | - |
| 112 UniRef90\_A0A1X0J8A0\_26\_405 | E | G | L | R | F | N | D | D | - | W | D | N | L | G | Q | R | L | T | A | S | G | S | V | E | F | D | S | V | L | L | R | H | D | E | V | L | T | G | L | - | - | - | - | A | - | - | - | - | - | - |
| 113 UniRef90\_A0A2J6Q859\_43\_430 | P | G | I | Q | F | A | H | D | - | W | D | N | V | G | L | R | L | T | E | S | G | G | V | K | L | E | N | V | R | A | S | W | K | D | A | F | G | W | D | - | - | - | - | P | - | - | - | - | - | - |
| 114 UniRef90\_UPI0003828F02\_7\_389 | P | G | I | R | Y | L | G | D | - | W | D | N | L | G | Q | R | R | S | A | S | G | G | V | E | F | A | N | V | A | L | A | P | D | A | V | L | G | W | T | - | - | - | - | P | - | - | - | - | - | - |
| 115 UniRef90\_A0A1B2HDJ6\_6\_361 | T | G | I | T | C | L | D | D | - | W | D | N | I | G | Q | R | L | T | A | S | G | G | V | V | F | E | N | V | E | V | T | - | - | E | V | L | G | V | Q | - | - | - | - | P | - | - | - | - | - | - |
| 116 UniRef90\_A0A2N3N0U1\_47\_427 | P | G | V | Q | F | Q | R | N | - | W | N | N | I | G | L | R | L | T | E | S | G | S | V | K | I | E | N | V | T | A | P | W | S | D | A | L | G | W | D | - | - | - | - | V | - | - | - | - | - | - |
| 117 UniRef90\_S5Y4X6\_32\_408 | A | G | I | S | F | G | N | D | - | W | D | N | L | G | Q | R | L | T | A | S | G | S | V | T | F | D | R | V | E | L | F | E | H | E | R | L | S | A | L | - | - | - | - | D | - | - | - | - | - | - |
| 118 UniRef90\_A0A3R2WQN2\_17\_411 | D | G | I | R | Y | L | D | D | - | W | D | H | L | G | Q | R | L | S | E | S | G | G | V | I | I | E | D | V | R | I | D | W | A | S | A | A | G | Y | V | - | - | - | - | D | - | - | - | - | - | - |
| 119 UniRef90\_A0A3N1X255\_20\_392 | D | G | V | G | I | A | H | D | - | W | D | G | I | G | Q | R | Q | T | G | S | G | T | V | L | F | Q | G | V | R | I | A | D | E | E | L | L | G | R | P | - | - | - | - | G | - | - | - | - | - | - |
| 120 UniRef90\_A0A1S1LA29\_28\_404 | A | G | L | R | F | N | D | D | - | W | D | N | L | G | Q | R | L | T | A | S | G | S | V | E | F | D | D | V | L | L | R | H | D | E | V | L | T | G | I | - | - | - | - | A | - | - | - | - | - | - |
| 121 UniRef90\_A0A081GNL4\_19\_392 | A | G | V | K | V | I | D | D | - | W | D | N | I | G | Q | R | Q | T | S | S | G | T | V | Q | F | E | Q | V | A | V | R | S | D | Q | I | L | F | P | V | - | - | - | - | R | - | - | - | - | - | - |
| 122 UniRef90\_A0A1I6U758\_27\_419 | D | A | I | T | F | G | D | D | - | W | D | N | L | G | Q | R | L | T | V | S | G | S | V | E | I | T | D | L | R | V | P | W | S | A | A | A | G | Y | V | - | - | - | - | D | - | - | - | - | - | - |
| 123 UniRef90\_A0A0F5VZP9\_7\_388 | P | G | L | T | V | I | D | D | - | W | D | G | L | G | Q | R | T | T | A | S | G | T | V | R | L | E | G | V | E | V | P | A | D | R | V | L | P | H | H | - | - | - | - | L | - | - | - | - | - | - |
| 124 UniRef90\_A0A1H4GIM8\_20\_411 | E | G | I | V | F | A | G | D | - | W | D | S | L | G | Q | R | L | T | E | S | G | S | V | E | I | R | D | V | R | V | P | W | T | A | A | A | G | F | V | - | - | - | - | D | - | - | - | - | - | - |
| 125 UniRef90\_A0A0M8TTT1\_8\_388 | P | G | L | T | V | I | D | D | - | W | D | G | M | G | Q | R | T | T | A | S | G | T | V | R | L | E | E | V | P | V | P | A | D | R | V | L | P | H | H | - | - | - | - | L | - | - | - | - | - | - |
| 126 UniRef90\_A0A1Y2MDA3\_48\_437 | P | G | I | Q | F | G | H | D | - | W | K | N | I | G | L | R | L | T | E | S | G | S | V | K | I | N | D | V | T | V | P | W | T | D | A | L | G | W | D | - | - | - | - | A | - | - | - | - | - | - |
| 127 UniRef90\_UPI000562F0C5\_9\_388 | P | G | L | T | V | I | D | D | - | W | D | G | L | G | Q | R | T | T | A | S | G | T | V | R | L | D | D | V | P | V | P | A | D | R | V | L | P | H | H | - | - | - | - | L | - | - | - | - | - | - |
| 128 UniRef90\_A0A506Y8Z1\_43\_442 | E | G | I | R | F | A | H | D | - | W | D | N | L | G | Q | R | L | T | E | S | G | S | V | E | I | R | D | V | R | V | P | W | A | A | A | A | G | F | V | - | - | - | - | D | - | - | - | - | - | - |
| 129 UniRef90\_A0A021VVW0\_28\_409 | P | A | I | T | F | H | D | G | - | W | D | A | L | G | Q | R | L | T | E | S | G | S | V | T | V | Q | D | A | V | V | P | W | T | A | A | L | G | W | V | - | - | - | - | D | - | - | - | - | - | - |
| 130 UniRef90\_E3QTS2\_46\_428 | A | G | V | R | F | L | H | N | - | W | D | N | V | G | L | R | L | S | E | S | G | S | V | I | I | E | N | V | T | A | P | W | A | D | A | L | G | W | D | - | - | - | - | A | - | - | - | - | - | - |
| 131 UniRef90\_A0A1D8SMV7\_7\_388 | P | G | V | T | V | T | D | D | - | W | D | G | L | G | Q | R | T | T | A | S | G | T | V | R | L | A | D | V | A | V | P | A | D | R | V | L | P | H | H | - | - | - | - | L | - | - | - | - | - | - |
| 132 UniRef90\_A9BUV8\_38\_419 | A | G | V | T | V | T | D | D | - | W | D | G | F | G | Q | R | V | T | G | S | G | S | V | Q | F | D | R | V | R | V | E | P | E | W | V | V | P | F | T | - | - | - | - | S | - | - | - | - | - | - |
| 133 UniRef90\_A0A1N6TB45\_32\_407 | E | G | V | V | I | E | D | D | - | W | D | G | I | G | Q | R | Q | T | G | S | G | R | V | S | F | H | G | V | R | V | H | A | D | E | V | L | G | P | P | - | - | - | - | A | P | L | D | P | T | R |
| 134 UniRef90\_A0A381IE41\_24\_406 | P | G | L | S | V | I | D | D | - | W | S | G | F | G | Q | R | T | T | A | S | G | T | V | R | L | D | N | V | R | V | P | A | S | H | V | F | P | A | H | - | - | - | - | R | - | - | - | - | - | - |
| 135 UniRef90\_A0A3M8TJ33\_18\_397 | P | G | V | V | A | G | G | D | G | G | D | T | F | G | Q | R | L | A | A | G | G | S | V | E | F | D | A | V | A | V | E | S | D | A | V | L | G | P | L | - | - | - | - | S | - | - | - | - | - | - |
| 136 UniRef90\_A0A1H3JL41\_4\_373 | P | G | I | G | H | P | D | D | - | W | D | N | I | G | Q | R | L | S | A | S | G | S | I | V | F | D | D | V | L | I | D | A | A | D | I | L | G | S | P | - | - | - | - | P | - | - | - | - | - | - |
| 137 UniRef90\_A0A507AW89\_49\_427 | P | G | I | Q | F | S | H | D | - | W | N | N | V | G | L | R | L | T | E | S | G | S | V | K | I | D | K | V | K | A | P | W | A | D | A | L | G | W | D | - | - | - | - | A | - | - | - | - | - | - |
| 138 UniRef90\_A0A1X1SEN2\_20\_406 | A | G | V | S | F | G | D | D | - | W | D | N | V | G | Q | R | L | T | V | S | G | T | T | T | L | T | G | V | E | I | P | V | D | A | V | I | G | Y | G | - | - | - | Y | G | - | V | - | - | - | - |
| 139 UniRef90\_A0A2D3UFX4\_26\_403 | H | G | V | G | I | D | D | D | - | A | D | T | F | G | Q | R | L | A | A | G | G | S | V | E | F | D | A | V | P | V | A | A | D | D | V | L | G | S | L | - | - | - | - | S | - | - | - | - | - | - |
| 140 UniRef90\_A0A089X0S9\_26\_403 | P | G | V | R | V | D | N | D | - | A | E | A | F | G | Q | R | L | A | A | G | G | S | V | E | F | D | A | V | P | V | A | A | D | D | I | L | G | S | L | - | - | - | - | S | - | - | - | - | - | - |
| 141 UniRef90\_A0A2W5T1D0\_29\_413 | P | G | I | V | F | G | R | D | - | W | D | S | L | G | Q | R | L | T | E | S | G | S | V | R | I | E | G | V | R | V | P | W | A | D | A | A | G | F | V | - | - | - | - | D | - | - | - | - | - | - |
| 142 UniRef90\_A0A1C4QGR9\_7\_388 | P | G | L | T | V | V | D | D | - | W | D | G | M | G | Q | R | T | T | A | S | G | T | V | R | L | T | G | V | V | V | P | A | D | R | V | L | P | H | H | - | - | - | - | L | - | - | - | - | - | - |
| 143 UniRef90\_A0A101R4L6\_7\_388 | P | G | L | T | V | V | D | D | - | W | D | G | M | G | Q | R | T | T | A | S | G | T | V | H | L | E | G | V | E | V | P | A | D | R | V | L | P | H | H | - | - | - | - | L | - | - | - | - | - | - |
| 144 UniRef90\_A0A4D4LHU8\_7\_388 | D | G | V | T | V | V | D | D | - | W | D | G | M | G | Q | R | T | T | A | S | G | T | V | R | L | E | A | V | S | V | P | A | D | R | V | L | P | H | H | - | - | - | - | L | - | - | - | - | - | - |
| 145 UniRef90\_L1KK35\_6\_389 | P | G | L | T | V | V | D | D | - | W | D | G | M | G | Q | R | T | T | A | S | G | T | V | R | L | E | S | V | P | V | P | A | D | R | V | V | P | H | H | - | - | - | - | L | - | - | - | - | - | - |
| 146 UniRef90\_UPI0005601C6B\_13\_390 | P | G | V | T | V | V | D | D | - | W | D | G | M | G | Q | R | T | T | A | S | G | T | V | R | L | A | G | V | A | V | P | A | D | R | V | V | P | H | H | - | - | - | - | L | - | - | - | - | - | - |
| 147 UniRef90\_A0A1H9UQJ9\_6\_361 | D | G | I | T | Y | L | D | D | - | W | D | N | I | G | Q | R | L | T | A | S | G | G | V | S | F | E | D | V | H | V | T | - | - | E | V | L | G | V | Q | - | - | - | - | P | - | - | - | - | - | - |
| 148 UniRef90\_A0A3D9JKY9\_12\_401 | P | G | V | V | R | G | S | G | - | G | D | T | F | G | Q | R | L | A | A | E | G | G | V | E | F | D | S | V | L | V | G | A | D | A | V | L | G | F | L | - | - | - | - | S | - | - | - | - | - | - |
| 149 UniRef90\_D9X7Q2\_26\_403 | P | G | V | L | T | D | G | D | - | A | D | P | F | G | Q | R | L | A | A | G | G | S | V | E | F | D | A | V | P | V | S | A | H | D | V | L | G | S | L | - | - | - | - | A | - | - | - | - | - | - |
| 150 UniRef90\_A0A1X1DXJ3\_20\_398 | A | G | I | E | I | V | D | D | - | W | S | G | M | G | Q | R | T | T | A | S | G | T | V | R | L | N | Q | V | A | V | D | P | A | W | I | I | P | L | P | - | - | - | - | - | - | - | - | - | - | - |

  
  

|  |  |  |  |  |  |  |  |  |  |  |  |  |  |  |  |  |  |  |  |  |  |  |  |  |  |  |  |  |  |  |  |  |  |  |  |  |  |  |  |  |  |  |  |  |  |  |  |  |  |  |
| --- | --- | --- | --- | --- | --- | --- | --- | --- | --- | --- | --- | --- | --- | --- | --- | --- | --- | --- | --- | --- | --- | --- | --- | --- | --- | --- | --- | --- | --- | --- | --- | --- | --- | --- | --- | --- | --- | --- | --- | --- | --- | --- | --- | --- | --- | --- | --- | --- | --- | --- |
| **001 Input\_pdb\_SEQRES\_A** | A | F | V | - | - | - | L | A | F | - | - | - | I | Q | S | E | R | G | S | L | F | A | P | I | A | Q | L | I | F | A | N | V | Y | L | G | I | A | H | G | A | L | D | A | A | R | E | Y | T | R | T |
| 002 UniRef90\_Q1W1G3\_1\_416 | A | F | I | - | - | - | V | A | F | - | - | - | I | Q | S | E | R | G | S | L | F | A | P | I | V | Q | L | I | L | A | N | L | Y | L | G | I | A | H | G | A | L | D | A | A | R | E | Y | T | R | T |
| 003 UniRef90\_T0BM21\_7\_392 | A | N | K | - | - | - | - | - | - | - | - | - | - | - | S | P | F | S | T | I | D | A | P | L | S | Q | M | I | L | A | N | I | F | A | G | S | A | T | G | A | L | E | A | A | K | E | Y | T | R | T |
| 004 UniRef90\_UPI0002AC58FE\_11\_391 | R | T | P | - | - | - | - | - | - | - | - | - | - | - | T | P | F | N | T | I | R | A | C | L | T | Q | L | N | L | A | N | I | Y | L | G | I | A | Q | G | A | L | A | A | A | K | Q | Y | T | R | T |
| 005 UniRef90\_A0A3D5CW51\_20\_401 | P | L | S | - | - | - | - | - | - | - | - | - | - | - | N | P | F | A | C | L | R | P | L | I | A | Q | L | I | L | T | N | I | Y | L | G | I | A | E | G | A | F | N | D | A | Q | H | Y | T | L | R |
| 006 UniRef90\_A0A2V4SPP5\_16\_400 | Q | A | P | - | - | - | - | - | - | - | - | - | - | - | T | A | R | S | T | L | R | S | Q | V | A | Q | L | V | M | A | N | L | Y | L | G | I | A | Q | G | A | F | D | A | A | R | D | Y | T | L | E |
| 007 UniRef90\_E0UIV5\_8\_393 | H | S | G | - | - | - | - | - | - | - | - | - | - | - | K | P | F | N | T | I | R | S | C | L | T | Q | L | N | L | A | N | I | Y | L | G | I | A | L | G | A | F | E | A | A | K | Q | Y | T | K | T |
| 008 UniRef90\_A0A1Z4S897\_9\_391 | S | N | D | - | - | - | D | - | - | - | - | - | - | P | R | P | F | A | T | L | F | T | P | L | G | Q | L | V | F | V | H | L | Y | L | G | I | A | L | G | A | F | A | E | A | K | K | Y | T | L | A |
| 009 UniRef90\_UPI00045E9273\_21\_396 | P | L | G | - | - | - | - | - | - | - | - | - | - | - | S | V | F | A | T | L | R | P | L | I | A | Q | L | V | L | I | N | I | Y | L | G | I | G | E | G | A | L | A | E | A | I | G | Y | T | R | S |
| 010 UniRef90\_A0A353Y4M9\_17\_384 | P | G | S | - | - | - | - | - | - | - | - | - | - | - | S | P | W | A | T | L | R | S | C | L | A | Q | S | I | L | V | N | I | Y | L | G | I | A | E | G | A | D | Q | S | A | R | E | H | T | T | R |
| 011 UniRef90\_F3KR10\_17\_384 | P | G | S | - | - | - | - | - | - | - | - | - | - | - | S | A | W | A | S | L | R | S | T | L | S | Q | S | M | L | V | N | I | Y | L | G | I | A | E | G | A | H | Q | A | A | R | D | H | T | R | Q |
| 012 UniRef90\_A0A2D8NW56\_20\_404 | A | P | W | - | - | - | - | - | - | - | - | - | - | - | T | P | A | T | Q | F | R | S | C | L | A | Q | L | V | L | V | N | L | Y | V | G | I | A | E | G | A | L | E | E | A | R | R | Y | T | L | E |
| 013 UniRef90\_A0A2W7M9P1\_16\_386 | N | D | D | - | - | - | - | - | - | - | - | - | - | - | N | V | F | A | T | V | R | T | H | I | A | Q | S | I | L | I | H | V | L | L | G | T | A | E | G | A | F | D | V | A | K | E | Y | T | K | T |
| 014 UniRef90\_A0A398AYR9\_12\_386 | T | D | S | - | - | - | - | - | - | - | - | - | - | - | N | L | F | A | T | V | R | T | H | I | A | Q | S | I | L | I | H | V | L | L | G | T | A | E | G | A | F | E | V | A | K | D | Y | T | K | T |
| 015 UniRef90\_A0A0T6UXN9\_11\_394 | P | L | S | - | - | - | - | - | - | - | - | - | - | - | T | P | F | A | C | L | R | P | L | I | A | Q | L | L | F | A | N | M | F | L | G | I | A | E | G | A | F | A | E | A | R | Q | Y | T | L | K |
| 016 UniRef90\_A0A252E884\_11\_390 | P | S | D | - | - | - | - | - | - | - | - | - | - | - | S | G | F | A | T | F | L | G | I | I | A | Q | L | T | K | T | Y | I | Y | L | G | I | A | E | G | A | L | A | A | A | K | E | Y | T | K | T |
| 017 UniRef90\_A0A1Z4BZ71\_24\_403 | P | L | S | - | - | - | - | - | - | - | - | - | - | - | S | P | F | S | S | L | R | S | M | V | A | Q | L | I | F | T | N | I | Y | I | G | L | A | E | G | A | L | Q | E | G | K | K | Y | T | Q | T |
| 018 UniRef90\_A0A1Z4IGC5\_10\_387 | S | I | G | - | - | - | - | - | - | - | - | - | - | - | T | P | F | A | A | L | R | I | Y | I | S | H | L | V | R | V | N | I | L | L | G | I | A | L | G | A | F | A | Q | A | K | E | Y | T | T | T |
| 019 UniRef90\_A0A1W9JD13\_18\_401 | P | L | S | - | - | - | - | - | - | - | - | - | - | - | T | P | F | S | C | L | R | P | L | V | A | Q | L | I | L | T | N | I | Y | L | G | I | A | E | G | A | F | A | D | A | K | Q | Y | T | L | K |
| 020 UniRef90\_A0A0B6S5D2\_8\_393 | V | P | P | - | - | - | - | - | - | - | - | - | - | - | T | P | R | A | T | L | R | T | L | V | S | Q | L | V | L | T | N | L | F | V | G | L | A | E | G | A | L | A | E | A | R | E | Y | V | G | T |
| 021 UniRef90\_A0A1Y3C786\_14\_396 | P | L | S | - | - | - | - | - | - | - | - | - | - | - | T | P | F | S | S | L | R | P | L | I | A | Q | L | V | F | V | S | M | F | L | G | V | A | E | G | A | F | T | E | A | K | H | Y | T | R | N |
| 022 UniRef90\_A0A318KD41\_20\_397 | P | L | S | - | - | - | - | - | - | - | - | - | - | - | T | P | F | S | A | L | R | P | L | I | A | Q | L | I | L | S | T | I | Y | L | G | I | A | E | G | A | L | S | E | A | R | R | Y | T | L | S |
| 023 UniRef90\_A0A352JDP6\_15\_389 | Q | S | D | - | - | - | - | - | - | - | - | - | - | - | R | P | F | K | T | I | R | A | C | L | T | Q | L | N | L | A | N | I | Y | L | G | I | A | I | G | A | F | E | T | A | R | N | Y | T | Q | T |
| 024 UniRef90\_A0A329B538\_13\_401 | P | L | T | - | - | - | - | - | - | - | - | - | - | - | T | P | F | S | C | L | R | P | L | I | A | Q | L | T | L | T | C | V | Y | L | G | I | A | E | G | A | F | D | E | A | R | H | F | S | L | H |
| 025 UniRef90\_A0A2N8QAF3\_13\_394 | - | - | - | - | - | - | - | - | - | - | - | - | - | - | T | P | Y | A | S | L | R | T | L | I | S | Q | Q | V | L | T | N | L | F | V | G | I | A | Q | G | A | L | E | E | A | R | E | Y | V | T | Q |
| 026 UniRef90\_A0A2N7XWT8\_11\_394 | P | L | S | - | - | - | - | - | - | - | - | - | - | - | T | P | F | A | C | L | R | P | L | I | A | Q | L | H | F | A | N | I | F | L | G | I | A | E | G | A | F | E | E | A | R | Q | Y | T | L | E |
| 027 UniRef90\_A0A2A4HLB2\_33\_412 | F | T | W | - | - | - | - | - | - | - | - | - | - | - | S | P | S | A | Q | F | R | A | C | L | A | Q | L | V | L | V | N | L | Y | V | G | I | A | E | G | A | F | E | E | A | K | R | F | T | L | E |
| 028 UniRef90\_A0A4R3HWL3\_41\_416 | T | A | G | - | - | - | - | - | - | - | - | - | - | - | T | P | R | A | T | L | R | T | L | V | S | Q | V | I | L | T | E | I | Y | I | G | N | A | Q | G | A | L | Q | N | A | W | K | Y | T | H | E |
| 029 UniRef90\_A0A1M7NXP6\_14\_394 | V | Q | A | - | - | - | - | - | - | - | - | - | - | - | T | P | Q | A | T | L | R | S | Q | V | A | Q | L | I | M | A | N | L | Y | L | G | I | G | E | G | A | F | E | A | A | R | E | Y | L | A | K |
| 030 UniRef90\_A0A1B4ESK2\_22\_397 | T | P | P | - | - | - | - | - | - | - | - | - | - | - | T | P | R | A | T | L | R | T | L | V | S | Q | L | V | L | T | N | L | F | V | G | L | A | E | G | A | L | A | E | A | R | E | Y | V | R | Q |
| 031 UniRef90\_A0A1H6NKV2\_11\_394 | P | L | S | - | - | - | - | - | - | - | - | - | - | - | T | P | F | A | C | L | R | P | L | I | A | Q | L | T | F | S | H | M | F | L | G | I | A | E | G | A | F | E | E | A | R | R | Y | T | L | T |
| 032 UniRef90\_A0A0D0KUF8\_11\_394 | P | L | S | - | - | - | - | - | - | - | - | - | - | - | T | P | F | A | C | L | R | P | L | I | A | Q | L | H | F | S | H | I | F | L | G | I | A | E | G | A | L | E | D | A | R | H | Y | T | L | K |
| 033 UniRef90\_A0A0F3K7B4\_20\_401 | P | L | T | - | - | - | - | - | - | - | - | - | - | - | T | P | R | S | C | L | R | P | L | L | A | Q | L | I | L | T | N | I | Y | L | G | I | A | E | G | A | F | E | E | A | R | H | Y | T | L | H |
| 034 UniRef90\_A0A381IMG6\_13\_394 | T | P | P | - | - | - | - | - | - | - | - | - | - | - | T | P | R | E | T | L | R | T | L | V | S | Q | L | V | L | T | N | L | F | V | G | I | A | E | G | A | L | A | E | A | R | D | Y | V | Q | R |
| 035 UniRef90\_A0A2S9K1G1\_26\_400 | Q | V | A | - | - | - | - | - | - | - | - | - | - | - | T | P | R | A | T | L | R | P | M | L | A | Q | L | I | L | T | N | L | Y | A | G | I | A | R | G | A | L | Q | E | G | L | R | Y | T | R | D |
| 036 UniRef90\_A0A1W6L7W5\_17\_392 | T | V | P | - | - | - | - | - | - | - | - | - | - | - | T | P | Q | A | T | L | R | S | Q | L | A | Q | L | I | L | V | N | L | Y | T | G | I | A | R | G | A | F | D | A | A | R | R | Y | T | V | E |
| 037 UniRef90\_A0A255HJE3\_14\_398 | Q | A | A | - | - | - | - | - | - | - | - | - | - | - | T | P | Q | T | T | V | R | S | Q | I | A | Q | L | I | M | T | N | L | Y | L | G | I | A | E | G | A | F | E | A | A | R | G | Y | T | S | E |
| 038 UniRef90\_A0A0R3AD45\_11\_394 | P | L | S | - | - | - | - | - | - | - | - | - | - | - | T | P | F | A | C | L | R | P | L | I | A | Q | L | T | F | T | H | M | F | L | G | I | A | E | G | A | F | D | E | A | R | N | Y | T | L | T |
| 039 UniRef90\_A0A1W6ZB60\_16\_389 | V | T | P | - | - | - | - | - | - | - | - | - | - | - | T | P | R | A | T | L | R | S | Q | V | A | Q | L | I | I | T | N | L | Y | L | G | I | A | Q | G | A | L | A | E | A | R | R | F | V | H | D |
| 040 UniRef90\_A0A2X1DPB7\_14\_397 | A | P | P | - | - | - | - | - | - | - | - | - | - | - | T | P | R | A | T | L | R | T | L | V | S | Q | L | V | L | T | N | L | F | V | G | L | A | E | G | A | L | A | E | A | R | D | Y | V | R | Q |
| 041 UniRef90\_A0A0S9M2D1\_4\_380 | P | L | S | - | - | - | - | - | - | - | - | - | - | - | T | P | F | A | C | L | R | P | L | V | A | Q | L | V | L | A | N | V | Y | L | G | I | A | E | N | A | F | E | D | A | R | R | Y | T | L | H |
| 042 UniRef90\_A0A238ZKW1\_13\_401 | P | L | S | - | - | - | - | - | - | - | - | - | - | - | T | P | F | S | C | L | R | P | L | L | A | Q | L | I | L | T | N | I | Y | L | G | I | A | E | G | A | F | E | D | A | R | H | Y | T | L | N |
| 043 UniRef90\_A0A0D1P8V6\_10\_394 | P | L | S | - | - | - | - | - | - | - | - | - | - | - | T | P | F | A | C | L | R | P | L | I | A | Q | L | H | F | A | N | I | C | L | G | I | A | E | G | A | F | E | E | A | R | Q | Y | T | L | N |
| 044 UniRef90\_A0A261SP68\_11\_387 | F | S | P | - | - | - | - | - | - | - | - | - | - | - | T | P | R | S | T | L | R | T | L | F | S | Q | A | I | M | A | N | L | Y | L | G | I | A | Q | G | A | L | A | E | A | C | R | F | V | L | E |
| 045 UniRef90\_A0A2N6MRB2\_11\_390 | P | P | N | - | - | - | - | - | - | - | - | - | - | - | H | P | F | S | T | F | L | G | I | I | A | Q | L | T | K | T | Y | V | Y | L | G | I | A | E | G | A | F | T | A | A | K | E | Y | T | K | T |
| 046 UniRef90\_A0A212BVA1\_31\_405 | Q | - | - | - | - | - | - | - | - | - | - | - | - | - | S | P | R | S | S | L | R | V | C | V | S | Q | L | I | L | T | Q | L | Y | L | G | N | S | Q | A | A | L | D | G | A | L | R | Y | V | R | E |
| 047 UniRef90\_A0A1A9KE82\_11\_394 | P | L | S | - | - | - | - | - | - | - | - | - | - | - | T | P | F | A | C | L | R | P | L | L | A | Q | L | I | F | A | H | M | F | L | G | I | A | E | G | A | F | E | E | F | R | G | Y | T | L | K |
| 048 UniRef90\_A0A4Q5PVB9\_26\_400 | N | A | T | - | - | - | - | - | - | - | - | - | - | - | S | P | R | A | S | L | R | N | M | L | G | Q | A | V | L | T | E | I | Y | L | G | N | A | Q | G | A | L | Q | Q | A | V | D | Y | T | R | T |
| 049 UniRef90\_A0A178GPM9\_9\_392 | P | L | S | - | - | - | - | - | - | - | - | - | - | - | T | P | Y | S | S | L | R | P | L | I | A | Q | L | I | F | V | H | L | F | L | G | V | A | E | G | A | F | D | V | A | K | Q | T | V | Q | - |
| 050 UniRef90\_A0A1P9YC32\_3\_390 | P | L | S | - | - | - | - | - | - | - | - | - | - | - | S | P | F | A | T | L | R | P | L | L | A | Q | A | I | L | S | H | I | Y | L | G | I | G | E | G | A | L | Q | A | A | R | E | A | T | L | A |
| 051 UniRef90\_UPI000A1773A9\_40\_416 | P | L | T | - | - | - | - | - | - | - | - | - | - | - | T | P | R | S | C | L | R | P | L | I | A | Q | L | I | L | T | N | I | Y | V | G | I | A | E | G | A | F | N | E | A | R | E | Y | T | L | R |
| 052 UniRef90\_A0A158L201\_25\_395 | V | P | P | - | - | - | - | - | - | - | - | - | - | - | T | P | R | A | T | L | R | T | L | V | S | Q | L | V | L | T | N | L | F | V | G | I | A | E | G | A | L | Q | E | A | R | D | Y | V | A | K |
| 053 UniRef90\_A0A4Q4GT15\_13\_391 | P | L | S | - | - | - | - | - | - | - | - | - | - | - | T | P | Y | S | S | L | R | P | L | I | A | Q | L | I | F | V | H | L | F | L | G | V | A | E | G | A | F | D | I | A | K | E | M | V | Q | - |
| 054 UniRef90\_A0A1C0YC62\_17\_381 | E | E | Q | - | - | - | - | - | - | - | - | - | - | - | H | Y | F | S | T | C | R | T | H | I | A | Q | T | I | L | L | H | V | L | L | G | T | A | E | G | A | F | Q | E | A | K | S | Y | T | K | T |
| 055 UniRef90\_A0A1H7FE04\_32\_405 | Q | A | P | - | - | - | - | - | - | - | - | - | - | - | T | P | R | A | T | L | R | S | Q | I | A | Q | L | V | M | T | H | L | Y | L | G | I | A | E | G | A | F | E | Q | A | R | R | Y | T | A | T |
| 056 UniRef90\_A0A401MWC4\_5\_385 | Q | D | E | - | - | - | G | T | - | - | - | - | - | L | S | P | F | A | T | L | V | T | P | A | I | Q | L | V | F | V | H | F | Y | L | G | I | A | E | G | A | L | A | A | A | A | D | Y | T | R | T |
| 057 UniRef90\_A0A1G6HFW7\_16\_395 | P | L | S | - | - | - | - | - | - | - | - | - | - | - | S | P | F | A | C | L | R | P | L | L | A | Q | L | I | L | A | H | I | Y | L | G | L | G | E | G | A | L | R | T | A | R | D | F | T | R | A |
| 058 UniRef90\_A0A0P9B999\_30\_403 | V | A | S | - | - | - | - | - | - | - | - | - | - | - | S | P | R | A | T | L | R | N | L | I | G | Q | I | V | L | T | E | I | Y | L | G | N | A | F | G | A | L | R | A | A | I | E | H | L | R | A |
| 059 UniRef90\_A0A140K6F0\_14\_392 | I | P | E | - | - | - | - | - | - | - | - | - | - | - | S | A | F | P | S | L | I | F | L | V | T | Q | L | S | K | T | F | V | Y | L | G | I | A | E | G | A | L | K | A | A | R | E | Y | T | L | T |
| 060 UniRef90\_A0A1P8EKI7\_5\_392 | P | L | S | - | - | - | - | - | - | - | - | - | - | - | T | P | Y | S | S | L | R | P | L | I | A | Q | L | I | F | V | H | L | F | L | G | V | A | E | G | A | F | K | V | A | H | Q | A | V | Q | - |
| 061 UniRef90\_A0A2U3MYZ3\_7\_392 | P | L | S | - | - | - | - | - | - | - | - | - | - | - | T | P | Y | S | G | L | R | P | L | I | A | Q | L | I | F | V | N | L | F | L | G | V | A | E | G | A | F | T | V | A | K | E | T | V | R | - |
| 062 UniRef90\_A0A1H2EPX5\_7\_397 | P | L | G | - | - | - | - | - | - | - | - | - | - | - | D | I | R | A | T | L | R | P | L | L | A | Q | S | I | L | V | H | V | Y | L | G | L | A | E | G | A | F | A | E | A | L | P | L | A | S | A |
| 063 UniRef90\_UPI00041EF43F\_25\_400 | T | V | P | - | - | - | - | - | - | - | - | - | - | - | R | P | R | A | T | L | R | S | Q | I | A | Q | L | V | L | V | N | L | Y | A | G | I | A | Q | G | A | L | S | Q | G | L | R | Y | T | R | D |
| 064 UniRef90\_D0IW93\_17\_384 | A | E | V | - | - | - | - | - | - | - | - | - | - | - | T | A | F | H | T | L | R | N | C | L | A | Q | L | V | L | V | N | L | F | V | G | V | A | Q | G | A | R | R | Q | A | R | D | Y | A | R | E |
| 065 UniRef90\_A0A395D1I6\_11\_388 | P | L | G | - | - | - | - | - | - | - | - | - | - | - | S | P | F | A | S | L | R | P | C | L | A | Q | L | V | L | T | N | I | Y | L | G | L | A | Q | G | A | L | E | Q | A | K | T | Y | L | H | G |
| 066 UniRef90\_A0A1H0TK45\_27\_400 | P | L | S | - | - | - | - | - | - | - | - | - | - | - | T | P | Y | A | C | L | R | P | L | L | A | Q | L | I | L | A | H | I | Y | Q | G | L | G | E | G | A | L | A | E | A | R | Q | F | T | L | Q |
| 067 UniRef90\_A0A1H8MYU5\_23\_403 | R | P | K | - | - | - | - | - | - | - | - | - | - | - | D | P | S | E | L | L | I | P | Q | Q | Q | Q | S | V | L | L | N | V | F | V | G | S | A | Q | G | A | L | N | A | A | R | D | Y | T | V | T |
| 068 UniRef90\_A0A1B1M254\_5\_403 | K | Q | F | - | - | - | K | P | - | - | - | - | - | - | R | V | Y | N | T | L | N | V | P | T | I | Q | L | V | F | V | N | F | Y | L | G | I | A | A | G | A | L | E | T | A | A | T | Y | T | R | E |
| 069 UniRef90\_A0A484THM3\_28\_401 | A | A | S | - | - | - | - | - | - | - | - | - | - | - | S | A | R | A | T | L | R | N | V | I | G | Q | L | V | L | T | E | L | Y | L | G | N | A | L | G | A | L | D | E | A | V | G | R | V | R | T |
| 070 UniRef90\_A0A0Q8Q8X6\_8\_377 | E | E | Q | - | - | - | - | - | - | - | - | - | - | - | T | F | Y | Q | S | L | R | T | C | F | A | Q | L | V | L | V | N | L | Y | L | G | I | A | M | G | A | F | E | E | A | R | R | Y | L | L | E |
| 071 UniRef90\_A0A0Q5QDL2\_32\_410 | K | E | F | - | - | - | Q | P | - | - | - | - | - | - | R | V | Y | N | T | L | N | V | P | I | I | Q | L | V | F | I | N | F | Y | L | G | I | A | R | G | A | L | Q | T | A | A | D | Y | T | R | D |
| 072 UniRef90\_A0A1A5XM62\_22\_406 | - | - | - | - | - | - | - | - | - | - | - | - | - | - | T | P | R | A | S | L | R | T | L | V | S | Q | L | V | L | T | N | L | F | V | G | I | A | E | G | A | L | E | E | A | R | E | Y | V | L | A |
| 073 UniRef90\_A0A239EI90\_21\_397 | K | Q | F | - | - | - | Q | P | - | - | - | - | - | - | R | V | Y | N | T | L | N | V | P | T | I | Q | L | V | F | V | N | F | Y | L | G | I | A | R | G | A | L | E | E | A | A | A | Y | T | K | D |
| 074 UniRef90\_S5SWW2\_23\_403 | R | P | K | - | - | - | - | - | - | - | - | - | - | - | R | P | A | A | L | L | G | P | Q | Q | Q | Q | S | V | L | L | N | V | F | V | G | S | A | Q | G | A | L | I | A | A | R | D | Y | T | V | T |
| 075 UniRef90\_A0A3A5JJY9\_31\_413 | A | P | T | - | - | - | - | - | - | - | - | - | - | - | T | T | R | H | Q | L | R | S | C | L | A | Q | L | V | L | V | N | L | Y | V | G | L | A | E | G | A | L | A | T | A | R | E | M | T | T | T |
| 076 UniRef90\_A0A315ZU85\_7\_384 | G | D | A | - | - | - | - | - | - | - | - | - | - | A | T | P | R | A | S | L | V | T | P | A | I | Q | A | V | F | G | N | F | Y | V | G | V | V | R | G | A | L | Q | T | A | A | E | Y | T | R | T |
| 077 UniRef90\_A0A3R9U899\_5\_381 | K | Q | F | - | - | - | Q | P | - | - | - | - | - | - | R | V | Y | N | T | L | N | V | P | T | I | Q | L | V | F | V | N | L | Y | L | G | I | A | G | G | A | L | E | T | A | A | A | Y | T | R | T |
| 078 UniRef90\_UPI000DD53D91\_7\_389 | L | P | L | - | - | - | - | - | - | - | - | - | - | - | T | P | F | S | S | L | T | S | L | L | Q | Q | S | V | L | L | N | V | F | V | G | S | A | Q | G | I | L | D | E | G | R | N | Y | T | T | T |
| 079 UniRef90\_UPI000DE4BE09\_13\_396 | A | P | D | - | - | - | T | A | - | - | - | - | - | P | S | V | L | S | T | F | N | T | P | L | I | Q | L | V | F | V | N | F | Y | L | G | T | A | E | G | A | L | Q | A | A | I | D | Y | V | R | T |
| 080 UniRef90\_A0A2T0R7P7\_10\_381 | S | - | - | - | - | - | - | - | - | - | - | - | - | S | G | A | F | G | S | L | V | T | P | S | I | Q | A | A | F | G | H | F | Y | L | G | V | T | R | G | A | L | E | A | A | S | E | Y | T | R | T |
| 081 UniRef90\_A0A4R1HYS3\_27\_404 | K | A | F | - | - | - | Q | P | - | - | - | - | - | - | R | V | Y | N | T | L | N | V | P | V | I | Q | L | V | F | V | S | F | Y | L | G | I | A | R | G | A | L | E | T | A | L | A | Y | T | R | T |
| 082 UniRef90\_UPI000D1537B0\_7\_385 | S | R | G | - | - | - | - | - | - | - | - | - | - | - | K | P | V | S | T | L | L | P | Q | M | S | Q | S | V | L | S | S | V | F | I | G | S | A | L | G | A | L | E | E | A | R | R | Y | A | M | T |
| 083 UniRef90\_A0A1G6ZNM7\_17\_409 | H | E | F | - | - | - | A | P | - | - | - | - | - | - | R | T | Y | N | T | L | N | V | P | L | I | Q | L | V | F | T | N | F | Y | L | G | I | A | Q | G | A | L | D | T | A | A | A | Y | T | R | E |
| 084 UniRef90\_UPI0003765980\_18\_389 | V | P | L | - | - | - | - | - | - | - | - | - | - | - | T | P | Y | T | S | L | T | S | L | L | Q | Q | S | V | L | L | N | V | F | V | G | S | A | Q | G | I | L | E | E | G | R | E | Y | T | V | G |
| 085 UniRef90\_A0A071ICK8\_25\_398 | E | P | D | - | - | - | A | P | - | - | - | - | - | P | P | V | L | S | T | F | N | T | P | L | I | Q | L | V | F | V | N | F | Y | L | G | T | A | E | G | A | L | E | A | A | L | D | Y | V | R | T |
| 086 UniRef90\_A0A2L0WNE5\_33\_396 | A | P | D | - | - | - | A | A | - | - | - | - | - | P | P | V | L | S | T | F | N | T | P | L | I | Q | L | V | F | V | N | F | Y | L | G | T | A | E | G | A | L | Q | A | A | L | D | Y | V | R | A |
| 087 UniRef90\_A0A1Q8CFJ2\_9\_380 | E | - | - | - | - | - | - | - | - | - | - | - | - | T | S | V | R | A | S | L | A | A | L | G | F | Q | L | M | L | A | Q | I | Y | V | G | L | A | E | G | A | L | T | E | A | A | E | Y | T | R | T |
| 088 UniRef90\_UPI000835F605\_21\_394 | H | Y | A | - | - | - | G | D | - | - | - | - | - | V | E | Q | R | D | G | L | R | V | L | F | S | Q | L | I | F | V | N | F | Y | L | G | I | A | E | G | A | L | K | A | A | S | A | Y | V | R | S |
| 089 UniRef90\_UPI000374AB86\_31\_398 | D | E | G | - | - | - | A | E | - | - | - | - | - | P | S | V | L | A | S | F | N | T | P | L | I | Q | L | V | F | V | N | F | Y | I | G | T | S | E | G | A | L | H | Q | A | L | D | Y | V | R | K |
| 090 UniRef90\_A0A267RVL6\_22\_399 | E | K | P | - | - | - | E | S | - | - | - | - | - | A | L | L | R | N | A | L | R | N | Q | F | S | Q | L | I | F | V | H | F | Y | L | G | I | A | E | G | A | L | K | Q | A | A | A | Y | F | R | D |
| 091 UniRef90\_M3VA40\_23\_395 | D | S | P | - | - | - | P | T | - | - | - | - | - | F | T | A | F | Q | Q | L | V | T | P | H | W | Q | L | A | F | V | N | F | Y | I | G | T | A | E | G | A | L | D | E | A | L | D | W | T | R | L |
| 092 UniRef90\_A0A395GID9\_32\_425 | A | S | K | - | - | - | T | P | R | D | D | V | L | K | V | P | F | A | S | L | L | L | P | T | I | Q | L | V | F | S | N | F | Y | L | G | I | A | Q | G | A | L | D | F | A | S | K | Y | T | V | T |
| 093 UniRef90\_UPI0008268C2F\_35\_406 | L | T | G | - | - | - | R | I | - | - | - | - | - | P | S | A | Y | Q | T | L | V | T | P | H | W | Q | L | A | F | V | N | F | Y | I | G | T | A | E | G | A | L | E | E | A | L | D | W | T | R | L |
| 094 UniRef90\_A0A0S1XV73\_27\_397 | S | P | L | - | - | - | - | - | - | - | - | - | - | - | S | P | F | E | S | V | V | S | L | L | Q | Q | S | V | L | A | N | V | F | I | G | S | A | V | G | A | L | D | E | A | R | E | Y | T | V | T |
| 095 UniRef90\_A0A0F5N116\_19\_398 | F | G | S | - | - | - | A | D | - | - | - | - | - | L | T | P | Y | Q | T | L | V | T | P | H | W | Q | L | A | F | V | N | F | Y | V | G | T | A | E | G | A | L | A | E | A | F | D | W | T | R | A |
| 096 UniRef90\_A0A1N6Z7J9\_40\_406 | E | K | G | - | - | - | A | A | - | - | - | - | - | P | T | V | L | A | T | F | N | T | P | L | I | Q | L | V | F | V | N | F | Y | I | G | T | A | E | G | A | L | S | A | A | T | D | Y | V | R | T |
| 097 UniRef90\_A0A2V4UWQ2\_31\_411 | A | P | D | - | - | - | A | A | - | - | - | - | - | P | K | V | Q | A | T | F | N | T | P | L | I | Q | L | V | F | A | N | F | Y | I | G | T | A | E | G | A | L | A | E | A | L | D | Y | V | R | D |
| 098 UniRef90\_A0A4P8KLT1\_5\_401 | D | - | - | - | - | - | - | - | - | - | - | - | - | - | E | P | I | Q | S | F | V | T | P | A | I | Q | L | V | F | G | N | L | Y | L | G | I | A | Q | G | A | L | A | Q | A | R | E | L | T | N | A |
| 099 UniRef90\_UPI0003068598\_3\_292 | A | P | P | - | - | - | - | - | - | - | - | - | - | - | T | P | R | A | T | L | R | A | L | V | S | Q | L | V | L | T | N | L | F | V | G | I | A | E | G | A | L | A | E | A | R | D | Y | V | Q | R |
| 100 UniRef90\_UPI000DD585C3\_27\_403 | A | P | S | - | - | - | D | P | - | - | - | - | - | P | P | V | Q | A | T | F | N | T | P | L | I | Q | L | V | F | V | N | F | Y | L | G | T | A | E | G | A | L | A | A | A | L | N | Y | V | S | T |
| 101 UniRef90\_A0A1E3SMN8\_25\_404 | F | I | G | - | - | - | N | T | - | - | - | - | - | L | T | P | H | Q | T | L | I | T | P | H | W | Q | L | A | F | V | N | F | Y | V | G | T | A | E | G | A | L | A | E | A | L | E | W | T | R | A |
| 102 UniRef90\_UPI00045EBD18\_12\_396 | A | P | P | - | - | - | - | - | - | - | - | - | - | - | P | P | R | T | S | L | V | V | P | F | H | Q | L | L | I | V | N | F | Y | I | G | I | A | K | G | A | L | A | E | A | N | R | Y | I | R | E |
| 103 UniRef90\_A0A1Y1ZNX6\_42\_434 | T | Q | R | - | - | - | K | P | D | P | A | I | L | G | I | P | F | G | S | L | L | L | P | T | I | Q | L | V | F | S | N | F | Y | L | G | I | A | L | G | A | Q | D | Y | A | S | K | Y | T | T | K |
| 104 UniRef90\_A0A0D2FPI6\_31\_423 | K | T | K | - | - | - | R | P | I | A | E | V | L | K | V | P | F | A | S | L | L | L | P | T | I | Q | L | V | F | S | N | F | Y | V | G | I | G | L | G | A | L | E | E | A | K | K | W | T | T | S |
| 105 UniRef90\_A0A2S0KGS2\_30\_403 | L | T | G | - | - | - | K | E | - | - | - | - | - | L | S | A | Y | Q | Q | L | V | T | P | H | W | Q | L | A | F | A | N | F | Y | I | G | T | A | E | G | A | L | D | E | A | L | D | W | T | R | L |
| 106 UniRef90\_A0A1A7MGQ0\_54\_442 | T | S | K | - | - | - | K | P | L | D | S | V | L | S | I | P | F | A | T | L | L | L | P | T | I | Q | L | V | F | A | N | F | Y | L | G | I | A | Q | G | S | L | E | F | A | R | G | Y | T | T | T |
| 107 UniRef90\_UPI000413001D\_18\_392 | P | G | S | - | - | - | A | A | - | - | - | - | P | L | S | P | R | V | S | L | V | V | P | L | H | Q | L | L | I | V | N | F | Y | V | G | T | A | E | G | A | L | A | E | A | N | A | Y | L | R | S |
| 108 UniRef90\_G7H5P9\_11\_400 | I | T | G | - | - | - | K | E | - | - | - | - | - | F | S | A | Y | Q | T | L | V | T | P | H | W | Q | L | A | F | V | N | F | Y | I | G | T | A | E | G | A | L | E | E | A | L | D | W | T | K | L |
| 109 UniRef90\_A0A010YHQ5\_11\_404 | Q | T | D | - | - | - | P | D | - | - | - | - | - | A | P | A | Y | P | S | L | S | A | L | G | F | Q | I | L | L | G | L | L | A | V | A | T | A | E | G | A | L | R | F | A | A | E | Y | T | R | T |
| 110 UniRef90\_B0RBU7\_24\_404 | D | - | - | - | - | - | - | - | - | - | - | - | - | - | E | P | F | S | T | L | V | T | P | A | I | Q | L | A | F | G | N | L | Y | L | G | I | A | E | G | A | L | A | Q | A | L | D | L | V | R | A |
| 111 UniRef90\_A0A167RW81\_42\_429 | Q | T | K | - | - | - | Q | P | D | P | A | I | L | S | I | P | F | A | S | L | L | L | P | T | I | Q | L | V | F | G | N | L | Y | L | G | I | A | Q | G | S | L | D | F | A | S | A | Y | T | T | K |
| 112 UniRef90\_A0A1X0J8A0\_26\_405 | E | L | S | A | S | D | G | S | - | - | - | - | - | R | E | R | R | D | G | L | R | A | L | F | S | Q | L | I | F | V | H | L | Y | L | G | I | A | E | G | A | L | A | A | G | V | T | Y | I | R | E |
| 113 UniRef90\_A0A2J6Q859\_43\_430 | S | S | K | - | - | - | K | P | I | P | E | I | L | T | I | P | F | A | T | L | L | L | P | T | I | Q | L | V | F | S | N | F | Y | L | G | I | A | L | G | G | L | D | F | A | S | K | Y | T | A | K |
| 114 UniRef90\_UPI0003828F02\_7\_389 | E | P | D | - | - | - | D | P | - | - | - | - | - | R | A | Q | R | D | S | L | A | A | L | G | F | Q | L | V | L | S | R | V | L | A | A | I | G | R | G | A | I | T | E | A | A | R | Y | T | R | E |
| 115 UniRef90\_A0A1B2HDJ6\_6\_361 | A | N | E | - | - | - | - | - | - | - | - | - | - | - | D | P | R | I | S | L | A | A | I | G | F | Q | L | V | L | A | Q | L | Y | V | A | I | A | E | G | A | L | D | E | A | A | D | Y | T | R | T |
| 116 UniRef90\_A0A2N3N0U1\_47\_427 | Q | K | K | - | - | - | E | P | D | L | A | Y | L | G | I | P | F | A | T | L | L | L | P | T | I | Q | L | V | F | S | N | F | Y | L | G | I | A | Q | G | A | L | A | T | A | S | K | Y | T | V | A |
| 117 UniRef90\_S5Y4X6\_32\_408 | H | W | P | - | - | - | G | E | - | - | - | - | - | S | A | E | R | D | G | L | R | G | L | F | S | Q | I | I | F | A | Q | F | Y | L | G | I | A | E | G | A | L | E | A | A | E | T | Y | I | R | E |
| 118 UniRef90\_A0A3R2WQN2\_17\_411 | K | V | Y | - | - | - | Q | S | - | - | - | - | - | - | R | T | Y | E | T | L | N | L | P | A | I | Q | L | V | F | A | N | F | Y | L | G | I | A | E | G | A | L | K | T | A | A | G | Y | T | R | E |
| 119 UniRef90\_A0A3N1X255\_20\_392 | D | E | V | - | - | - | - | - | - | - | - | - | - | - | T | P | F | V | S | L | N | S | L | L | Q | Q | L | V | L | A | Q | V | F | I | G | S | A | Q | G | A | L | A | E | G | R | D | Y | T | V | R |
| 120 UniRef90\_A0A1S1LA29\_28\_404 | E | Y | S | D | R | D | G | S | - | - | - | - | - | R | E | R | R | D | G | L | R | A | L | F | S | Q | L | I | F | V | H | L | Y | L | G | I | A | E | G | A | L | A | A | G | V | A | Y | V | R | D |
| 121 UniRef90\_A0A081GNL4\_19\_392 | Q | G | A | - | - | - | - | - | - | - | - | - | - | - | T | P | F | S | T | I | R | T | L | L | A | Q | L | N | L | A | N | L | Y | L | G | L | A | E | G | A | L | K | E | A | C | E | R | F | H | E |
| 122 UniRef90\_A0A1I6U758\_27\_419 | K | R | F | - | - | - | R | A | - | - | - | - | - | - | S | A | F | S | T | L | H | V | P | A | L | H | L | V | F | A | N | L | Y | L | G | I | A | Q | A | A | L | R | A | G | V | A | Y | T | R | G |
| 123 UniRef90\_A0A0F5VZP9\_7\_388 | - | - | - | - | - | - | - | - | - | - | - | - | - | T | F | D | G | P | Q | L | H | G | T | L | A | Q | L | L | H | A | A | I | D | A | G | I | A | G | G | A | L | A | E | A | A | E | F | V | R | T |
| 124 UniRef90\_A0A1H4GIM8\_20\_411 | R | E | F | - | - | - | Q | P | - | - | - | - | - | - | L | V | Y | N | T | L | N | V | P | T | I | Q | L | V | F | A | N | F | Y | L | G | I | A | Q | G | A | L | E | A | G | S | S | Y | T | R | S |
| 125 UniRef90\_A0A0M8TTT1\_8\_388 | - | - | - | - | - | - | - | - | - | - | - | - | - | T | F | R | G | P | Q | L | H | G | A | V | A | Q | L | L | H | A | A | I | D | A | G | I | A | G | G | A | L | A | A | A | A | E | F | V | R | T |
| 126 UniRef90\_A0A1Y2MDA3\_48\_437 | E | Q | K | - | - | - | K | P | D | P | K | V | L | Q | N | S | F | A | A | L | L | L | P | T | I | Q | L | V | F | S | N | F | Y | L | G | I | A | L | G | A | Q | Q | F | A | S | K | Y | T | V | K |
| 127 UniRef90\_UPI000562F0C5\_9\_388 | - | - | - | - | - | - | - | - | - | - | - | - | - | T | F | H | G | P | Q | L | H | G | A | V | A | Q | L | L | H | A | A | I | D | A | G | I | A | G | D | A | L | V | Q | A | V | E | F | V | R | T |
| 128 UniRef90\_A0A506Y8Z1\_43\_442 | K | T | F | - | - | - | Q | P | - | - | - | - | - | - | L | I | Y | N | T | L | N | V | P | T | I | Q | L | V | F | A | N | F | Y | L | G | I | A | Q | G | T | L | E | R | A | T | A | Y | T | R | E |
| 129 UniRef90\_A0A021VVW0\_28\_409 | K | A | F | - | - | - | R | P | - | - | - | - | - | - | H | V | Y | N | T | L | C | L | V | A | I | Q | L | V | F | T | S | F | Y | L | G | I | A | S | G | G | L | Q | T | A | L | E | Y | T | R | A |
| 130 UniRef90\_E3QTS2\_46\_428 | S | K | K | - | - | - | K | P | D | P | S | I | L | S | I | P | F | A | T | L | L | L | P | T | I | Q | L | V | F | S | N | F | Y | I | G | I | A | W | G | A | L | N | F | A | S | A | Y | T | K | R |
| 131 UniRef90\_A0A1D8SMV7\_7\_388 | - | - | - | - | - | - | - | - | - | - | - | - | - | T | F | Q | G | P | Q | L | H | G | A | V | A | Q | L | L | H | A | A | I | D | A | G | I | A | A | G | A | L | A | E | A | A | E | F | V | R | T |
| 132 UniRef90\_A9BUV8\_38\_419 | - | - | - | - | - | - | - | - | - | - | - | - | - | S | F | E | R | P | T | T | I | G | P | F | A | Q | I | I | H | A | A | L | D | A | G | I | G | H | G | A | L | Q | A | A | L | P | F | I | R | E |
| 133 UniRef90\_A0A1N6TB45\_32\_407 | A | P | A | - | - | - | - | - | - | - | - | - | - | - | E | P | W | R | T | V | V | P | M | L | Q | Q | S | V | L | L | N | V | F | V | G | S | A | Q | G | A | V | R | T | A | R | D | T | L | L | A |
| 134 UniRef90\_A0A381IE41\_24\_406 | - | - | - | - | - | - | - | - | - | - | - | - | - | V | S | D | Q | P | T | L | N | G | P | L | S | Q | I | I | Q | A | A | I | D | A | G | I | A | H | A | A | L | D | D | T | L | R | F | V | R | E |
| 135 UniRef90\_A0A3M8TJ33\_18\_397 | S | D | D | - | - | - | G | T | - | - | - | - | - | M | S | P | Y | A | G | L | S | A | P | T | G | R | L | V | S | A | Q | I | C | L | G | I | A | Q | G | V | L | A | E | A | R | E | Y | T | R | A |
| 136 UniRef90\_A0A1H3JL41\_4\_373 | S | - | - | - | - | - | - | - | - | - | - | - | - | G | D | V | R | I | S | L | T | S | L | A | F | Q | A | I | L | A | Q | I | C | V | A | I | A | E | G | A | L | A | E | A | A | D | Y | T | R | R |
| 137 UniRef90\_A0A507AW89\_49\_427 | A | K | K | - | - | - | K | P | D | P | S | I | L | G | I | P | F | A | T | L | L | L | P | T | I | Q | L | V | F | S | N | F | Y | I | G | I | A | W | G | A | L | S | E | A | S | A | Y | T | N | K |
| 138 UniRef90\_A0A1X1SEN2\_20\_406 | A | P | A | - | - | - | - | - | - | - | - | - | - | - | E | P | A | D | H | L | D | V | L | Y | M | Y | A | G | F | A | A | I | F | T | G | I | A | R | G | A | F | D | E | A | A | D | Y | T | R | A |
| 139 UniRef90\_A0A2D3UFX4\_26\_403 | T | D | E | - | - | - | D | V | - | - | - | - | - | L | S | P | L | A | A | L | A | S | P | L | G | R | L | L | S | V | Q | L | L | L | G | M | A | E | G | V | L | A | E | A | R | E | Y | S | R | A |
| 140 UniRef90\_A0A089X0S9\_26\_403 | T | E | E | - | - | - | D | A | - | - | - | - | - | L | S | P | R | T | A | L | I | S | P | V | G | R | L | L | S | V | Q | L | R | L | G | M | A | E | G | V | L | A | E | A | R | D | Y | S | R | T |
| 141 UniRef90\_A0A2W5T1D0\_29\_413 | K | V | F | - | - | - | Q | P | - | - | - | - | - | - | L | V | Y | G | T | L | N | V | P | A | I | Q | L | V | F | A | N | F | Y | L | G | I | A | E | G | A | L | E | R | A | A | A | Y | T | R | E |
| 142 UniRef90\_A0A1C4QGR9\_7\_388 | - | - | - | - | - | - | - | - | - | - | - | - | - | T | F | R | G | P | Q | L | H | G | A | T | A | Q | L | L | H | A | A | I | D | V | G | I | A | D | G | A | L | A | E | A | V | S | F | V | R | T |
| 143 UniRef90\_A0A101R4L6\_7\_388 | - | - | - | - | - | - | - | - | - | - | - | - | - | T | F | R | G | P | Q | L | H | G | A | V | A | Q | L | L | H | A | A | I | D | A | G | I | A | S | G | A | L | T | E | A | A | A | F | V | R | T |
| 144 UniRef90\_A0A4D4LHU8\_7\_388 | - | - | - | - | - | - | - | - | - | - | - | - | - | T | F | Q | G | P | Q | L | H | G | A | V | A | Q | L | L | H | A | A | I | D | A | G | I | A | A | G | A | L | A | E | A | A | E | F | V | R | T |
| 145 UniRef90\_L1KK35\_6\_389 | - | - | - | - | - | - | - | - | - | - | - | - | - | T | F | R | G | P | Q | L | H | G | A | V | A | Q | L | L | H | A | A | I | D | A | G | I | A | A | G | A | L | A | D | A | V | E | F | V | R | T |
| 146 UniRef90\_UPI0005601C6B\_13\_390 | - | - | - | - | - | - | - | - | - | - | - | - | - | T | F | R | G | P | Q | L | H | G | A | V | A | Q | L | L | H | A | A | I | D | A | G | I | A | A | A | A | L | G | A | A | V | E | F | V | R | T |
| 147 UniRef90\_A0A1H9UQJ9\_6\_361 | A | G | E | - | - | - | - | - | - | - | - | - | - | - | D | P | R | I | S | L | A | A | I | G | F | Q | L | V | L | A | Q | L | Y | V | A | I | A | E | G | A | L | Q | E | A | A | D | Y | T | R | T |
| 148 UniRef90\_A0A3D9JKY9\_12\_401 | P | D | E | - | - | - | D | V | - | - | - | - | - | L | S | P | F | A | S | L | A | A | P | T | A | R | L | A | S | A | Q | F | C | L | G | I | A | E | G | L | L | A | E | V | H | E | H | G | R | A |
| 149 UniRef90\_D9X7Q2\_26\_403 | A | D | E | - | - | - | D | V | - | - | - | - | - | L | S | P | L | T | S | M | V | S | P | V | G | R | L | L | S | V | Q | L | R | L | G | M | A | E | G | V | L | A | E | A | R | E | Y | S | R | T |
| 150 UniRef90\_A0A1X1DXJ3\_20\_398 | - | - | - | - | - | - | - | - | - | - | - | - | - | S | P | E | K | P | T | L | R | G | A | V | S | Q | L | I | Q | A | A | I | D | A | G | I | A | Q | G | A | L | D | D | A | L | A | F | V | R | D |

  
  

|  |  |  |  |  |  |  |  |  |  |  |  |  |  |  |  |  |  |  |  |  |  |  |  |  |  |  |  |  |  |  |  |  |  |  |  |  |  |  |  |  |  |  |  |  |  |  |  |  |  |  |
| --- | --- | --- | --- | --- | --- | --- | --- | --- | --- | --- | --- | --- | --- | --- | --- | --- | --- | --- | --- | --- | --- | --- | --- | --- | --- | --- | --- | --- | --- | --- | --- | --- | --- | --- | --- | --- | --- | --- | --- | --- | --- | --- | --- | --- | --- | --- | --- | --- | --- | --- |
| **001 Input\_pdb\_SEQRES\_A** | Q | A | R | P | W | T | P | A | - | - | - | G | I | - | - | - | - | Q | Q | A | T | E | D | P | Y | T | I | R | S | Y | G | E | F | T | I | A | L | Q | G | A | D | A | A | A | R | E | A | A | H | L |
| 002 UniRef90\_Q1W1G3\_1\_416 | Q | A | R | P | W | T | P | A | - | - | - | R | V | - | - | - | - | Q | Q | A | T | E | D | P | Y | V | L | R | A | Y | G | E | F | T | I | A | L | Q | G | A | D | A | A | A | R | E | A | A | H | L |
| 003 UniRef90\_T0BM21\_7\_392 | Q | S | R | P | W | Y | T | S | - | - | - | G | V | - | - | - | - | N | A | A | H | R | D | P | Y | T | L | R | K | Y | G | D | M | W | V | E | L | Q | G | A | T | S | L | L | D | A | A | G | H | S |
| 004 UniRef90\_UPI0002AC58FE\_11\_391 | S | T | K | P | W | L | T | S | - | - | - | G | V | - | - | - | - | E | S | A | T | V | D | P | Y | I | L | Q | H | Y | G | N | M | W | V | E | L | Q | G | A | T | C | L | T | D | I | A | G | E | L |
| 005 UniRef90\_A0A3D5CW51\_20\_401 | E | A | R | P | W | K | N | A | - | - | - | N | V | - | - | - | - | D | I | T | S | Q | D | P | Y | I | L | A | H | Y | G | E | F | W | V | G | L | E | A | T | R | A | L | T | N | Q | A | A | E | K |
| 006 UniRef90\_A0A2V4SPP5\_16\_400 | Q | A | R | P | W | F | A | S | - | - | - | G | V | - | - | - | - | D | D | A | I | D | D | P | F | T | Q | H | R | Y | G | Q | L | W | L | T | V | R | P | A | A | L | L | A | D | E | A | A | R | Q |
| 007 UniRef90\_E0UIV5\_8\_393 | T | T | K | P | W | L | T | S | - | - | - | G | V | - | - | - | - | E | S | A | S | E | D | P | Y | I | L | Q | H | Y | G | N | L | W | V | D | L | Q | A | A | A | A | L | V | D | Q | A | G | E | S |
| 008 UniRef90\_A0A1Z4S897\_9\_391 | H | T | R | P | W | I | I | S | - | - | - | P | A | - | - | - | - | E | T | A | A | Q | D | P | Y | I | I | E | Q | Y | G | N | M | W | V | D | L | A | A | T | I | S | H | A | D | H | V | T | L | L |
| 009 UniRef90\_UPI00045E9273\_21\_396 | Q | S | R | A | W | F | L | S | - | - | - | G | V | - | - | - | - | E | S | A | T | R | D | P | Y | V | L | K | K | Y | G | E | F | W | V | D | L | N | A | A | A | L | A | T | D | Y | A | A | S | L |
| 010 UniRef90\_A0A353Y4M9\_17\_384 | W | R | R | P | W | I | S | S | - | - | - | G | V | - | - | - | - | E | Q | A | T | Q | D | P | Y | L | L | Q | H | F | G | E | F | W | V | Q | L | Q | A | A | R | A | L | A | D | E | A | A | V | K |
| 011 UniRef90\_F3KR10\_17\_384 | W | R | R | P | W | V | L | S | - | - | - | N | V | - | - | - | - | E | Q | A | T | Q | D | P | Y | L | L | E | K | F | G | E | F | W | V | Q | L | K | A | A | E | A | L | A | E | Q | A | A | Q | Q |
| 012 UniRef90\_A0A2D8NW56\_20\_404 | Q | A | R | P | W | L | A | A | - | - | - | G | V | - | - | - | - | D | R | A | S | E | D | P | Y | T | Q | R | H | F | G | E | L | W | V | K | L | R | C | A | E | V | L | A | D | L | A | G | S | V |
| 013 UniRef90\_A0A2W7M9P1\_16\_386 | Q | T | R | P | W | V | T | S | - | - | - | N | V | - | - | - | - | D | T | A | A | E | D | P | Y | H | V | Y | N | Y | G | E | L | F | V | K | L | K | A | A | D | A | L | T | K | V | S | N | E | V |
| 014 UniRef90\_A0A398AYR9\_12\_386 | K | T | R | P | W | V | T | S | - | - | - | H | V | - | - | - | - | D | A | A | I | N | D | P | Y | N | I | Y | H | Y | G | D | L | F | V | K | L | K | A | A | D | A | L | V | H | I | S | N | E | L |
| 015 UniRef90\_A0A0T6UXN9\_11\_394 | E | S | R | P | W | F | R | S | - | - | - | S | A | - | - | - | - | R | S | S | G | E | D | P | Y | I | L | R | H | Y | G | D | F | W | V | G | L | E | S | S | R | V | L | I | Q | R | A | V | D | Q |
| 016 UniRef90\_A0A252E884\_11\_390 | Q | T | R | P | W | I | T | S | - | - | - | G | V | - | - | - | - | D | S | A | S | K | D | P | Y | I | L | R | H | Y | G | E | F | W | A | E | L | Q | A | A | I | A | L | S | D | R | T | A | A | Q |
| 017 UniRef90\_A0A1Z4BZ71\_24\_403 | S | S | R | V | W | S | G | S | - | - | - | L | A | - | - | - | - | Q | T | V | Q | E | D | P | Y | T | L | L | H | Y | G | E | F | W | A | S | I | D | G | A | R | L | L | A | D | H | A | A | A | L |
| 018 UniRef90\_A0A1Z4IGC5\_10\_387 | T | T | Q | P | W | G | A | S | - | - | - | G | V | - | - | - | - | H | S | A | T | A | D | P | Y | I | L | Q | T | Y | G | K | L | W | V | D | L | N | A | A | T | V | L | S | D | E | A | A | E | K |
| 019 UniRef90\_A0A1W9JD13\_18\_401 | E | A | K | P | W | H | T | A | - | - | - | N | I | - | - | - | - | A | S | T | S | Q | D | P | Y | I | L | G | H | Y | G | D | F | F | V | G | I | E | A | T | K | A | L | T | D | L | A | A | H | R |
| 020 UniRef90\_A0A0B6S5D2\_8\_393 | R | G | R | P | W | I | H | S | - | - | - | G | V | - | - | - | - | E | R | A | Q | D | D | P | Y | T | L | Q | R | F | G | E | M | R | V | R | A | V | A | A | E | A | L | A | D | R | A | A | R | A |
| 021 UniRef90\_A0A1Y3C786\_14\_396 | E | S | K | A | W | F | L | S | - | - | - | G | V | - | - | - | - | D | Q | A | N | E | D | P | Y | I | L | R | H | Y | G | E | F | W | L | A | L | E | S | L | K | L | L | N | Q | N | A | I | A | K |
| 022 UniRef90\_A0A318KD41\_20\_397 | Q | R | R | L | W | P | A | S | - | - | - | L | A | - | - | - | - | Q | S | A | N | Q | D | P | Y | T | L | L | H | Y | G | E | F | W | V | G | L | E | G | A | R | L | L | V | E | R | A | A | R | Q |
| 023 UniRef90\_A0A352JDP6\_15\_389 | E | T | R | P | F | I | N | T | - | - | - | G | I | - | - | - | - | D | S | A | I | Q | D | P | Y | I | L | E | K | Y | G | D | M | W | I | N | L | Q | A | T | E | A | L | V | D | R | A | G | L | A |
| 024 UniRef90\_A0A329B538\_13\_401 | E | A | R | P | W | H | R | S | - | - | - | A | A | - | - | - | - | A | T | V | G | E | D | P | Y | V | L | N | H | Y | G | E | F | W | L | G | L | E | S | V | R | A | L | V | E | R | A | A | R | M |
| 025 UniRef90\_A0A2N8QAF3\_13\_394 | Y | G | K | P | W | I | S | S | - | - | - | G | V | - | - | - | - | D | Q | A | T | D | D | P | Y | L | I | Q | R | F | G | E | M | R | L | Q | A | V | S | A | E | A | L | A | T | R | A | A | Y | A |
| 026 UniRef90\_A0A2N7XWT8\_11\_394 | E | A | R | P | W | F | R | S | - | - | - | S | A | - | - | - | - | S | H | S | T | Q | D | P | Y | I | L | S | H | Y | G | D | F | W | V | A | L | Q | S | T | R | L | L | V | E | R | A | G | Q | V |
| 027 UniRef90\_A0A2A4HLB2\_33\_412 | Q | G | R | P | W | L | A | S | - | - | - | E | A | - | - | - | - | A | Q | A | Q | D | D | P | Y | L | Q | R | H | F | G | E | L | W | V | K | L | R | S | A | Q | V | L | A | D | I | A | G | D | I |
| 028 UniRef90\_A0A4R3HWL3\_41\_416 | H | A | K | P | W | M | T | S | - | - | - | G | V | - | - | - | - | A | R | A | I | D | D | P | F | V | H | Q | H | Y | G | D | L | W | I | A | L | R | S | A | I | A | L | T | E | A | A | E | G | Q |
| 029 UniRef90\_A0A1M7NXP6\_14\_394 | E | A | R | P | W | F | A | S | - | - | - | G | V | - | - | - | - | A | S | A | G | A | D | P | L | V | Q | H | R | F | G | Q | L | W | L | K | L | R | P | A | S | V | L | A | D | Q | A | A | Q | E |
| 030 UniRef90\_A0A1B4ESK2\_22\_397 | H | G | R | P | W | I | H | S | - | - | - | D | V | - | - | - | - | E | R | A | E | D | D | P | Y | T | L | Q | R | F | G | D | M | R | V | R | G | V | A | A | A | S | L | A | D | R | A | A | I | A |
| 031 UniRef90\_A0A1H6NKV2\_11\_394 | E | T | R | P | W | F | K | S | - | - | - | G | T | - | - | - | - | E | D | I | R | N | D | P | Y | I | L | S | H | Y | G | D | F | W | V | A | L | E | G | V | R | L | L | V | E | R | A | A | E | G |
| 032 UniRef90\_A0A0D0KUF8\_11\_394 | E | G | R | P | W | F | R | S | - | - | - | K | A | - | - | - | - | Q | H | T | S | E | D | P | Y | I | L | R | H | Y | G | E | F | W | V | G | L | E | G | V | R | L | L | V | E | R | A | A | A | Q |
| 033 UniRef90\_A0A0F3K7B4\_20\_401 | E | A | R | P | W | R | A | A | - | - | - | Q | V | - | - | - | - | D | H | T | H | E | D | P | Y | I | L | G | H | Y | G | E | F | W | V | G | L | E | S | V | R | A | L | T | D | R | A | G | N | S |
| 034 UniRef90\_A0A381IMG6\_13\_394 | A | G | R | P | W | V | H | S | - | - | - | G | V | - | - | - | - | D | R | A | A | D | D | P | Y | T | L | Q | R | F | G | D | M | R | V | Q | A | V | S | A | E | A | L | A | D | R | A | A | R | A |
| 035 UniRef90\_A0A2S9K1G1\_26\_400 | A | S | R | P | F | F | A | S | - | - | - | G | V | - | - | - | - | S | R | A | V | D | D | P | Y | V | Q | Q | R | Y | G | Q | L | A | V | L | V | R | P | A | E | L | L | A | D | L | A | A | Q | A |
| 036 UniRef90\_A0A1W6L7W5\_17\_392 | E | S | R | P | W | F | A | S | - | - | - | G | V | - | - | - | - | S | R | A | A | D | D | P | F | V | Q | H | R | Y | G | D | L | W | L | K | V | R | P | A | I | V | L | A | D | D | A | A | Q | R |
| 037 UniRef90\_A0A255HJE3\_14\_398 | Q | A | K | A | W | F | A | S | - | - | - | G | V | - | - | - | - | A | Q | A | A | D | D | P | L | V | Q | H | R | Y | G | Q | L | W | L | L | L | R | P | A | Q | V | L | A | D | L | A | A | L | E |
| 038 UniRef90\_A0A0R3AD45\_11\_394 | E | T | R | A | W | H | K | S | - | - | - | T | A | - | - | - | - | E | D | V | R | Q | D | P | Y | V | L | H | H | Y | G | E | F | W | V | A | L | Q | G | V | R | L | L | V | E | R | A | A | A | L |
| 039 UniRef90\_A0A1W6ZB60\_16\_389 | D | A | R | P | W | F | A | A | - | - | - | D | V | - | - | - | - | A | R | H | A | D | D | P | Y | V | Q | E | R | F | G | E | F | R | L | A | V | R | A | A | E | A | L | A | D | Q | A | G | I | R |
| 040 UniRef90\_A0A2X1DPB7\_14\_397 | H | G | R | P | W | I | H | S | - | - | - | D | V | - | - | - | - | E | R | A | E | D | D | P | Y | T | L | Q | R | F | G | D | M | R | V | R | A | V | A | A | A | A | L | A | D | R | A | A | H | A |
| 041 UniRef90\_A0A0S9M2D1\_4\_380 | E | A | R | P | W | P | A | S | - | - | - | P | A | - | - | - | - | A | Q | A | G | D | D | P | Y | V | L | A | H | Y | G | E | F | W | V | G | L | E | G | V | R | A | L | A | D | R | A | A | G | R |
| 042 UniRef90\_A0A238ZKW1\_13\_401 | E | A | R | A | W | H | L | S | - | - | - | G | V | - | - | - | - | A | L | P | E | D | D | P | Y | T | L | A | H | Y | G | E | F | W | A | S | L | Q | S | A | R | A | L | T | N | L | A | A | A | Q |
| 043 UniRef90\_A0A0D1P8V6\_10\_394 | E | T | R | P | W | F | K | S | - | - | - | T | A | - | - | - | - | L | D | S | R | D | D | P | Y | V | L | G | H | F | G | D | F | W | V | R | L | Q | G | A | R | L | L | L | D | N | A | A | G | E |
| 044 UniRef90\_A0A261SP68\_11\_387 | D | A | R | P | W | F | A | A | - | - | - | D | V | - | - | - | - | Q | R | Q | A | D | D | P | Y | V | Q | A | R | F | G | E | F | R | L | A | T | R | A | A | E | A | L | A | D | A | A | G | R | Q |
| 045 UniRef90\_A0A2N6MRB2\_11\_390 | Q | T | R | P | W | I | T | S | - | - | - | G | V | - | - | - | - | D | S | A | N | Q | D | P | Y | I | L | H | H | Y | G | E | L | W | T | Q | L | Q | A | A | I | A | L | S | D | R | T | L | S | Q |
| 046 UniRef90\_A0A212BVA1\_31\_405 | Q | A | R | P | W | L | G | A | - | - | - | G | V | - | - | - | - | N | E | A | S | E | D | P | F | I | Q | K | R | F | G | E | L | W | L | L | Y | R | G | A | L | L | Q | A | E | L | A | A | E | R |
| 047 UniRef90\_A0A1A9KE82\_11\_394 | E | A | R | P | W | F | R | S | - | - | - | S | A | - | - | - | - | E | S | I | S | E | D | P | Y | V | L | R | H | Y | G | D | F | W | V | G | L | E | G | V | R | L | L | V | E | R | A | A | A | Q |
| 048 UniRef90\_A0A4Q5PVB9\_26\_400 | Q | V | Q | P | W | A | M | A | - | - | - | G | V | - | - | - | - | S | R | A | V | D | D | P | L | L | Q | L | R | A | G | Q | L | W | A | S | L | R | A | A | I | A | L | A | E | E | A | N | A | K |
| 049 UniRef90\_A0A178GPM9\_9\_392 | T | Q | K | A | W | S | K | S | - | - | - | L | V | - | - | - | - | E | D | A | V | N | D | P | F | I | Q | K | H | F | A | E | F | Y | V | Q | L | E | G | V | R | L | L | A | D | K | A | V | Q | A |
| 050 UniRef90\_A0A1P9YC32\_3\_390 | Q | T | R | P | W | I | A | S | - | - | - | Q | A | - | - | - | - | E | R | P | A | D | D | P | Y | V | L | A | N | Y | G | D | F | W | I | A | L | E | G | A | R | L | L | V | E | R | A | M | Q | A |
| 051 UniRef90\_UPI000A1773A9\_40\_416 | E | A | R | P | W | H | V | T | - | - | - | G | L | - | - | - | - | E | R | A | D | D | D | P | Y | V | L | A | H | F | G | E | F | F | V | G | L | E | S | L | R | L | L | A | D | R | A | G | A | Q |
| 052 UniRef90\_A0A158L201\_25\_395 | N | G | R | P | W | I | H | S | - | - | - | N | V | - | - | - | - | T | R | A | A | D | D | P | Y | Q | L | H | R | F | A | E | M | R | L | K | A | V | S | A | E | A | L | A | T | R | A | A | Q | A |
| 053 UniRef90\_A0A4Q4GT15\_13\_391 | S | Q | K | A | W | S | K | S | - | - | - | L | A | - | - | - | - | E | N | A | V | N | D | P | F | T | Q | K | H | F | A | E | F | Y | V | Q | L | E | S | V | R | L | L | A | N | K | A | V | E | T |
| 054 UniRef90\_A0A1C0YC62\_17\_381 | S | A | R | P | W | V | T | A | - | - | - | H | V | - | - | - | - | N | D | A | T | E | D | P | Y | I | L | Q | H | Y | G | T | F | F | A | K | L | Q | A | A | D | A | L | L | E | R | A | V | Q | T |
| 055 UniRef90\_A0A1H7FE04\_32\_405 | Q | T | K | A | W | F | A | S | - | - | - | G | V | - | - | - | - | E | R | A | V | D | D | P | L | V | Q | H | R | Y | G | D | L | W | L | H | V | R | A | A | Q | G | I | A | R | E | A | V | S | A |
| 056 UniRef90\_A0A401MWC4\_5\_385 | T | T | R | P | W | L | L | S | - | - | - | D | V | - | - | - | - | E | S | A | T | Q | D | P | Y | T | L | A | T | Y | G | E | L | A | V | S | A | R | S | V | R | V | L | A | D | H | A | A | E | A |
| 057 UniRef90\_A0A1G6HFW7\_16\_395 | S | S | R | A | W | I | A | S | - | - | - | P | A | - | - | - | - | S | H | P | T | E | D | L | Y | V | Q | G | H | Y | G | R | F | W | L | A | L | E | G | A | R | V | L | V | G | R | A | A | S | A |
| 058 UniRef90\_A0A0P9B999\_30\_403 | H | T | Q | P | W | P | M | A | - | - | - | G | V | - | - | - | - | E | R | V | E | D | D | R | L | L | Q | L | R | A | G | E | M | W | S | A | L | Q | A | A | T | A | L | S | N | Q | A | N | E | R |
| 059 UniRef90\_A0A140K6F0\_14\_392 | S | T | R | P | W | I | S | S | - | - | - | G | V | - | - | - | - | E | R | A | S | L | D | P | F | I | L | H | H | Y | G | E | F | W | T | E | L | Q | A | A | I | A | L | A | E | R | A | A | H | Q |
| 060 UniRef90\_A0A1P8EKI7\_5\_392 | H | Q | K | A | W | A | T | S | - | - | - | S | A | - | - | - | - | T | Q | S | V | D | D | P | Y | I | Q | K | H | F | A | E | F | Y | V | Q | L | E | S | V | R | L | L | T | N | K | A | R | T | L |
| 061 UniRef90\_A0A2U3MYZ3\_7\_392 | Q | Q | K | A | W | S | S | S | - | - | - | L | V | - | - | - | - | D | Q | A | V | N | D | P | Y | T | Q | K | H | F | A | E | F | Y | V | Q | L | E | S | V | R | L | L | T | Q | K | A | I | E | T |
| 062 UniRef90\_A0A1H2EPX5\_7\_397 | A | T | R | P | W | L | A | S | - | - | - | G | V | - | - | - | - | E | R | P | Q | D | D | P | Y | L | L | A | R | H | G | E | I | A | L | A | L | R | S | A | A | L | L | A | E | A | A | A | R | S |
| 063 UniRef90\_UPI00041EF43F\_25\_400 | S | S | R | P | F | F | A | S | - | - | - | G | L | - | - | - | - | A | R | A | V | D | D | P | Y | V | Q | H | R | Y | G | E | L | S | V | L | V | R | P | A | E | V | L | A | D | L | A | A | H | K |
| 064 UniRef90\_D0IW93\_17\_384 | H | G | R | P | W | L | G | S | - | - | - | A | V | - | - | - | - | E | R | A | T | D | D | P | Y | L | L | R | R | M | G | E | M | Q | A | Q | I | S | G | A | A | L | M | A | D | H | G | A | E | L |
| 065 UniRef90\_A0A395D1I6\_11\_388 | A | P | - | - | - | - | A | E | - | - | - | I | R | - | - | - | - | E | R | V | G | Q | D | P | Y | A | L | R | N | F | G | E | L | W | V | E | I | A | A | A | R | A | L | T | D | E | A | Q | R | A |
| 066 UniRef90\_A0A1H0TK45\_27\_400 | Q | G | R | P | W | M | A | S | - | - | - | G | A | - | - | - | - | A | S | A | Q | Q | D | P | Y | I | L | G | H | Y | G | D | F | W | L | A | L | E | G | S | R | L | L | L | Q | N | A | A | A | A |
| 067 UniRef90\_A0A1H8MYU5\_23\_403 | T | S | R | P | W | I | Y | S | - | - | - | G | V | - | - | - | - | E | R | H | S | D | D | P | W | I | K | R | Q | Y | G | E | L | W | T | K | V | Q | A | A | T | A | L | A | D | R | A | A | A | A |
| 068 UniRef90\_A0A1B1M254\_5\_403 | K | S | R | S | W | L | H | G | - | - | - | G | H | - | - | - | - | E | R | A | V | D | E | P | Y | V | I | D | T | Y | G | D | L | T | A | K | L | W | A | V | E | A | L | A | D | A | V | A | A | E |
| 069 UniRef90\_A0A484THM3\_28\_401 | Q | V | Q | P | W | A | A | S | - | - | - | G | V | - | - | - | - | A | H | A | S | D | D | G | L | L | Q | L | R | A | G | D | L | W | L | Q | L | A | G | A | T | A | L | A | D | Q | A | L | D | A |
| 070 UniRef90\_A0A0Q8Q8X6\_8\_377 | Q | G | R | P | W | I | H | A | - | - | - | G | V | - | - | - | - | A | R | A | T | D | D | P | Y | M | I | H | R | F | A | E | M | H | V | Q | I | S | A | A | T | V | L | A | D | R | A | A | A | M |
| 071 UniRef90\_A0A0Q5QDL2\_32\_410 | T | T | R | P | W | L | H | G | - | - | - | G | F | - | - | - | - | D | R | A | V | D | E | P | Y | V | I | D | T | Y | G | D | L | T | T | K | L | W | A | A | E | A | L | A | D | A | V | A | V | E |
| 072 UniRef90\_A0A1A5XM62\_22\_406 | N | G | R | P | W | V | N | S | - | - | - | G | V | - | - | - | - | Q | R | A | A | D | D | P | Y | L | Q | Q | R | F | G | E | M | R | I | Q | A | L | S | A | S | L | L | A | D | R | A | A | S | L |
| 073 UniRef90\_A0A239EI90\_21\_397 | K | T | R | A | W | L | H | S | - | - | - | T | V | - | - | - | - | D | K | A | V | D | E | P | Y | I | I | D | I | Y | G | D | L | T | S | K | L | W | A | V | E | A | L | A | D | A | V | A | L | E |
| 074 UniRef90\_S5SWW2\_23\_403 | R | S | R | P | W | I | Y | S | - | - | - | G | V | - | - | - | - | E | R | H | S | D | D | P | W | I | K | R | Q | Y | G | E | L | W | A | K | V | Q | A | A | T | A | L | A | D | R | A | A | E | A |
| 075 UniRef90\_A0A3A5JJY9\_31\_413 | R | A | R | P | W | A | A | A | - | - | - | D | V | - | - | - | - | E | A | A | I | D | D | P | Y | V | Q | H | R | Y | G | R | L | W | V | R | L | R | A | A | Q | L | L | A | N | H | A | A | Q | A |
| 076 UniRef90\_A0A315ZU85\_7\_384 | T | S | R | A | W | L | L | S | - | - | - | D | S | - | - | - | - | P | S | A | A | Q | D | P | Y | V | L | A | T | Y | G | R | L | V | A | R | L | R | A | A | E | A | L | A | D | S | A | G | E | A |
| 077 UniRef90\_A0A3R9U899\_5\_381 | R | T | R | P | W | L | H | G | - | - | - | G | H | - | - | - | - | E | R | A | V | D | E | P | Y | I | V | D | T | Y | G | D | L | T | A | K | L | W | A | V | E | A | L | A | D | A | V | A | D | E |
| 078 UniRef90\_UPI000DD53D91\_7\_389 | S | S | R | P | W | I | Y | S | - | - | - | G | V | - | - | - | - | E | R | H | I | D | D | P | W | I | K | R | Q | Y | G | D | L | F | V | R | A | L | A | A | S | E | L | A | D | K | A | A | R | S |
| 079 UniRef90\_UPI000DE4BE09\_13\_396 | T | T | R | P | W | V | T | S | - | - | - | G | V | - | - | - | - | Q | R | A | S | D | D | P | Y | I | L | E | R | V | G | E | L | T | A | A | L | K | A | S | A | A | L | A | D | S | A | A | A | A |
| 080 UniRef90\_A0A2T0R7P7\_10\_381 | T | S | R | P | W | L | L | S | - | - | - | D | V | - | - | - | - | E | K | A | V | E | D | P | Y | V | L | A | T | Y | G | R | L | V | A | R | L | R | A | A | E | A | L | G | R | S | V | G | E | S |
| 081 UniRef90\_A0A4R1HYS3\_27\_404 | T | T | R | S | W | L | H | S | - | - | - | D | A | - | - | - | - | E | R | A | V | D | E | P | Y | Q | L | D | L | Y | G | D | Y | Q | S | K | L | W | A | V | E | A | L | A | D | Q | V | A | L | E |
| 082 UniRef90\_UPI000D1537B0\_7\_385 | K | S | R | P | W | L | D | A | - | - | - | G | V | - | - | - | - | A | C | H | H | E | D | P | W | V | R | R | V | F | G | D | L | W | I | R | T | E | A | A | R | L | L | V | E | K | A | D | R | A |
| 083 UniRef90\_A0A1G6ZNM7\_17\_409 | R | T | R | A | W | P | Y | A | - | - | - | A | D | R | - | - | K | A | R | A | V | D | E | F | Y | I | Q | E | T | Y | G | G | L | Q | S | K | L | W | A | A | E | A | L | A | D | R | A | A | G | L |
| 084 UniRef90\_UPI0003765980\_18\_389 | S | S | R | P | W | I | Y | S | - | - | - | G | V | - | - | - | - | E | R | H | I | D | D | P | W | I | K | R | Q | Y | G | E | L | Y | I | R | T | L | A | T | A | E | L | A | D | K | A | A | R | S |
| 085 UniRef90\_A0A071ICK8\_25\_398 | T | T | R | P | W | V | T | S | - | - | - | G | V | - | - | - | - | E | K | A | S | D | D | P | Y | I | L | E | R | V | G | E | F | T | A | A | L | K | A | S | A | A | L | A | D | S | A | A | E | A |
| 086 UniRef90\_A0A2L0WNE5\_33\_396 | T | T | R | P | W | V | T | S | - | - | - | G | V | - | - | - | - | E | R | A | A | D | D | P | Y | I | L | E | R | V | G | E | F | T | A | S | L | K | A | A | T | A | L | A | D | S | A | A | A | A |
| 087 UniRef90\_A0A1Q8CFJ2\_9\_380 | K | T | R | A | W | T | L | S | - | - | - | D | V | - | - | - | - | D | E | A | V | A | D | P | Y | I | L | V | G | Y | G | E | L | V | A | Q | T | R | A | A | G | A | L | T | D | R | A | A | A | A |
| 088 UniRef90\_UPI000835F605\_21\_394 | H | G | R | P | W | P | E | S | - | - | - | G | L | - | - | - | - | E | K | A | T | E | D | P | Y | H | L | Q | L | F | G | R | L | S | A | G | I | A | A | G | V | A | L | A | D | K | A | A | A | L |
| 089 UniRef90\_UPI000374AB86\_31\_398 | T | T | R | P | W | I | T | S | - | - | - | G | V | - | - | - | - | A | R | A | E | E | D | P | Y | I | L | E | R | V | G | E | F | T | A | S | L | R | A | S | A | A | L | A | D | T | A | A | L | T |
| 090 UniRef90\_A0A267RVL6\_22\_399 | T | V | R | P | W | P | E | S | - | - | - | G | V | - | - | - | - | E | S | A | L | E | D | P | Y | H | R | L | K | A | G | E | L | S | S | D | L | A | A | G | I | A | L | A | E | K | T | A | H | A |
| 091 UniRef90\_M3VA40\_23\_395 | N | A | S | A | W | E | T | S | - | - | - | G | L | - | - | - | - | E | A | A | T | D | D | P | Y | I | L | E | L | V | G | E | L | R | A | E | I | T | A | A | V | L | L | A | D | R | A | G | D | A |
| 092 UniRef90\_A0A395GID9\_32\_425 | S | T | R | A | W | P | F | G | - | - | - | G | D | N | - | - | K | D | S | P | T | E | E | F | Y | I | L | E | R | Y | G | N | F | F | A | H | L | R | A | A | E | A | L | A | D | R | A | G | N | E |
| 093 UniRef90\_UPI0008268C2F\_35\_406 | N | A | S | P | W | E | T | S | - | - | - | G | L | - | - | - | - | D | Q | A | T | D | D | P | Y | I | L | E | L | V | G | Q | L | R | S | E | I | S | A | A | A | L | L | A | D | R | A | G | D | A |
| 094 UniRef90\_A0A0S1XV73\_27\_397 | Q | S | R | P | W | I | H | S | - | - | - | G | V | - | - | - | - | E | R | H | V | D | D | P | W | I | Q | R | A | Y | G | D | L | A | I | R | T | L | A | A | I | E | L | A | D | D | A | A | A | H |
| 095 UniRef90\_A0A0F5N116\_19\_398 | Y | A | S | P | W | E | S | S | - | - | - | G | V | - | - | - | - | E | R | A | T | D | D | P | Y | I | L | Q | T | V | G | E | L | V | S | E | V | R | A | A | A | L | L | A | D | R | A | G | D | A |
| 096 UniRef90\_A0A1N6Z7J9\_40\_406 | T | T | R | P | W | V | T | S | - | - | - | G | V | - | - | - | - | A | T | A | S | E | D | P | Y | I | L | E | R | I | G | E | F | R | A | T | L | K | A | S | A | A | L | A | D | A | A | A | H | A |
| 097 UniRef90\_A0A2V4UWQ2\_31\_411 | T | T | R | P | W | I | T | S | - | - | - | G | V | - | - | - | - | E | R | A | A | D | D | P | H | I | L | E | R | V | G | E | F | R | A | A | L | K | A | A | A | A | L | A | D | S | A | G | L | A |
| 098 UniRef90\_A0A4P8KLT1\_5\_401 | R | P | N | S | W | F | L | S | - | - | - | G | V | - | - | - | - | Q | T | Y | S | D | D | P | F | V | R | R | L | Y | G | E | L | V | S | R | T | A | A | V | E | A | L | A | D | R | V | N | S | R |
| 099 UniRef90\_UPI0003068598\_3\_292 | A | G | R | P | W | L | H | S | - | - | - | G | V | - | - | - | - | E | R | A | A | D | D | P | Y | T | L | Q | R | F | G | D | M | R | V | Q | T | V | S | A | E | A | L | A | D | R | A | A | R | A |
| 100 UniRef90\_UPI000DD585C3\_27\_403 | T | T | R | P | W | I | T | S | - | - | - | G | V | - | - | - | - | T | R | A | G | D | D | P | Y | I | L | E | R | I | G | E | L | Q | A | A | L | K | A | S | V | A | L | A | D | V | A | A | G | I |
| 101 UniRef90\_A0A1E3SMN8\_25\_404 | H | A | S | P | W | E | S | S | - | - | - | G | V | - | - | - | - | E | R | A | T | D | D | P | Y | I | L | H | T | V | G | E | L | V | S | E | V | R | A | A | A | L | L | A | D | R | A | G | D | A |
| 102 UniRef90\_UPI00045EBD18\_12\_396 | V | S | R | P | W | Q | A | S | - | - | - | G | V | - | - | - | - | E | K | A | R | L | D | P | Y | I | L | E | H | Y | G | N | L | D | V | E | L | R | A | S | T | A | L | A | D | A | A | G | V | R |
| 103 UniRef90\_A0A1Y1ZNX6\_42\_434 | N | T | R | P | W | P | F | G | - | - | - | G | D | N | - | - | K | E | R | A | T | D | E | F | Y | I | L | S | T | Y | G | N | F | H | A | H | L | R | A | A | V | A | L | A | N | Q | A | G | A | E |
| 104 UniRef90\_A0A0D2FPI6\_31\_423 | K | T | R | A | W | P | Y | G | - | - | - | G | D | N | - | - | K | A | K | A | T | D | E | H | Y | I | L | A | R | Y | G | N | F | H | A | H | L | R | A | A | E | A | L | A | D | L | A | G | E | K |
| 105 UniRef90\_A0A2S0KGS2\_30\_403 | H | A | S | A | W | E | T | S | - | - | - | G | L | - | - | - | - | E | A | A | T | D | D | P | Y | I | L | E | L | V | G | E | L | R | S | Q | I | A | A | A | A | L | L | A | D | R | A | G | D | V |
| 106 UniRef90\_A0A1A7MGQ0\_54\_442 | S | K | R | A | W | P | F | G | - | - | - | G | D | N | - | - | K | D | S | A | T | E | E | F | Y | I | L | E | R | Y | G | N | F | H | A | H | L | L | A | A | E | A | L | T | D | R | A | S | K | E |
| 107 UniRef90\_UPI000413001D\_18\_392 | T | A | R | P | W | Q | A | S | - | - | - | G | V | - | - | - | - | S | E | A | R | Q | D | P | Y | V | L | E | H | Y | G | E | L | D | V | Q | A | R | A | A | A | A | L | A | D | V | A | G | A | A |
| 108 UniRef90\_G7H5P9\_11\_400 | N | A | S | A | W | E | T | S | - | - | - | G | L | - | - | - | - | E | T | A | V | D | D | P | Y | I | L | E | L | V | G | E | L | K | S | Q | V | T | A | A | A | L | L | A | D | R | A | G | D | A |
| 109 UniRef90\_A0A010YHQ5\_11\_404 | K | S | R | A | W | A | T | S | - | - | - | G | V | - | - | - | - | A | E | A | A | D | D | P | L | V | R | V | R | Y | G | E | L | E | S | Q | V | R | A | A | R | A | L | T | D | R | A | G | A | A |
| 110 UniRef90\_B0RBU7\_24\_404 | R | R | G | A | W | F | L | S | - | - | - | G | V | - | - | - | - | D | A | Y | R | D | D | P | F | V | Q | R | V | V | G | E | L | A | S | R | I | A | A | V | E | A | L | A | D | R | V | G | R | A |
| 111 UniRef90\_A0A167RW81\_42\_429 | H | T | R | A | W | P | F | G | - | - | - | G | E | N | - | - | K | E | K | G | K | D | E | F | Y | V | L | S | A | Y | G | N | F | A | A | H | L | R | A | A | E | A | L | T | D | K | A | G | D | E |
| 112 UniRef90\_A0A1X0J8A0\_26\_405 | Q | G | R | P | W | P | E | A | - | - | - | H | S | - | - | - | - | T | Q | V | T | E | D | P | Y | H | Q | Q | L | L | G | R | L | S | A | G | I | A | A | G | I | A | L | A | D | T | V | T | R | E |
| 113 UniRef90\_A0A2J6Q859\_43\_430 | S | T | R | A | W | P | F | G | - | - | - | G | D | N | - | - | K | D | K | A | S | E | E | F | Y | I | L | A | T | Y | G | N | F | Y | A | H | L | R | A | A | R | A | L | A | D | R | A | G | D | R |
| 114 UniRef90\_UPI0003828F02\_7\_389 | T | S | R | A | W | P | A | S | - | - | - | G | V | - | - | - | - | E | H | A | V | D | D | P | H | I | L | A | G | Y | G | S | L | I | S | R | L | D | A | A | D | L | L | V | D | A | A | A | D | S |
| 115 UniRef90\_A0A1B2HDJ6\_6\_361 | K | T | R | P | W | F | V | S | - | - | - | G | V | - | - | - | - | E | H | A | T | E | D | P | Y | I | V | A | A | Y | G | E | M | V | S | Q | T | K | A | A | G | L | L | A | D | E | A | A | K | L |
| 116 UniRef90\_A0A2N3N0U1\_47\_427 | T | T | R | A | W | P | F | G | - | - | - | G | E | N | - | - | K | E | K | G | T | D | E | F | Y | V | L | S | T | Y | G | N | F | H | A | H | L | R | A | A | E | A | L | A | D | Q | A | G | R | E |
| 117 UniRef90\_S5Y4X6\_32\_408 | E | G | R | P | W | P | E | S | - | - | - | G | L | - | - | - | - | S | S | A | V | E | D | P | Y | N | A | V | I | L | G | R | L | S | A | Q | V | E | A | G | I | A | L | A | D | R | A | T | L | A |
| 118 UniRef90\_A0A3R2WQN2\_17\_411 | K | T | R | A | W | P | Y | G | - | - | - | G | D | N | - | - | K | E | S | A | S | D | E | W | Y | I | L | E | A | Y | G | T | L | Q | S | R | L | W | A | A | E | E | L | A | N | S | A | G | A | E |
| 119 UniRef90\_A0A3N1X255\_20\_392 | E | S | R | P | W | I | H | S | - | - | - | G | V | - | - | - | - | E | R | H | T | D | D | P | W | V | Q | R | R | Y | G | G | L | H | I | R | V | Q | A | A | Q | A | L | A | E | Q | A | A | A | Q |
| 120 UniRef90\_A0A1S1LA29\_28\_404 | K | G | R | P | W | P | E | A | - | - | - | Y | S | - | - | - | - | T | E | V | T | E | D | P | Y | H | Q | Q | L | L | G | R | L | S | G | G | I | A | A | G | I | A | L | A | D | S | V | T | R | E |
| 121 UniRef90\_A0A081GNL4\_19\_392 | R | H | G | P | - | - | S | G | - | - | - | G | L | - | - | - | - | G | D | G | T | G | A | V | L | A | R | D | R | F | A | R | L | W | V | P | L | Q | A | A | D | A | H | Y | E | R | S | L | V | R |
| 122 UniRef90\_A0A1I6U758\_27\_419 | H | T | R | A | W | P | Y | G | - | - | - | G | E | G | - | - | K | N | S | G | T | E | E | F | Y | V | L | E | G | Y | G | D | L | Q | S | K | L | W | A | A | E | T | L | T | D | T | A | G | A | S |
| 123 UniRef90\_A0A0F5VZP9\_7\_388 | K | S | R | P | W | F | E | S | - | - | - | G | L | - | - | - | - | D | T | A | A | E | D | P | L | L | I | Q | R | F | G | E | L | A | L | Q | V | R | A | T | E | A | L | L | R | E | A | A | R | A |
| 124 UniRef90\_A0A1H4GIM8\_20\_411 | T | T | R | P | W | P | Y | G | - | - | - | G | D | N | - | - | K | Q | R | A | G | E | E | W | Y | L | L | E | G | Y | G | E | L | Q | S | K | L | W | A | D | E | A | L | L | D | V | A | G | A | E |
| 125 UniRef90\_A0A0M8TTT1\_8\_388 | K | S | R | P | W | F | E | S | - | - | - | G | A | - | - | - | - | E | T | A | A | E | D | P | L | L | I | Q | R | F | G | E | L | A | I | Q | V | R | A | S | E | A | L | L | R | D | A | A | R | A |
| 126 UniRef90\_A0A1Y2MDA3\_48\_437 | G | T | R | A | W | P | F | G | - | - | - | G | D | N | - | - | K | E | R | A | T | D | E | F | Y | I | L | E | R | Y | G | N | F | H | A | H | L | R | A | A | V | A | L | T | D | S | A | G | E | Q |
| 127 UniRef90\_UPI000562F0C5\_9\_388 | K | S | R | P | W | F | E | S | - | - | - | G | V | - | - | - | - | E | T | A | A | E | D | P | L | L | I | Q | R | F | G | E | L | A | L | Q | V | R | A | S | E | A | L | L | R | E | A | A | R | A |
| 128 UniRef90\_A0A506Y8Z1\_43\_442 | N | S | R | A | W | P | Y | G | - | - | - | G | D | D | - | - | K | T | R | A | T | E | E | W | Y | I | L | E | G | Y | G | E | L | Q | S | K | L | W | A | D | E | A | L | L | D | A | V | G | A | E |
| 129 UniRef90\_A0A021VVW0\_28\_409 | R | T | R | P | W | P | Y | G | - | - | - | G | D | D | - | - | R | E | R | G | V | D | E | P | Y | V | L | D | T | Y | G | D | L | Q | S | K | L | W | A | A | E | A | L | A | E | R | A | G | E | A |
| 130 UniRef90\_E3QTS2\_46\_428 | N | T | R | A | W | P | F | G | - | - | - | G | D | N | - | - | K | E | K | A | V | D | E | F | Y | I | L | S | T | Y | G | N | F | F | A | H | L | R | A | A | E | A | L | A | D | K | A | G | L | E |
| 131 UniRef90\_A0A1D8SMV7\_7\_388 | K | S | R | P | W | F | E | S | - | - | - | G | A | - | - | - | - | E | T | A | V | E | D | P | L | L | V | Q | R | F | G | E | L | A | L | A | V | R | A | S | E | A | L | L | S | E | A | A | R | T |
| 132 UniRef90\_A9BUV8\_38\_419 | H | A | R | P | W | V | D | A | - | - | - | G | V | - | - | - | - | A | S | A | T | Q | D | P | L | L | L | Q | Q | L | G | N | V | H | V | R | L | R | A | A | D | A | L | V | A | R | A | A | L | A |
| 133 UniRef90\_A0A1N6TB45\_32\_407 | A | - | - | - | - | - | - | P | - | - | - | A | S | - | - | - | - | R | R | L | Q | H | D | P | G | V | Q | R | L | L | G | E | L | W | S | R | T | R | A | A | T | A | L | A | D | R | A | L | R | A |
| 134 UniRef90\_A0A381IE41\_24\_406 | R | S | R | P | W | I | D | S | - | - | - | G | V | - | - | - | - | E | R | A | A | D | D | P | L | T | I | R | E | T | G | R | L | V | I | H | L | H | A | A | D | A | L | L | E | R | A | A | R | T |
| 135 UniRef90\_A0A3M8TJ33\_18\_397 | A | D | V | P | W | R | L | D | - | - | - | G | P | S | - | - | - | Y | G | S | P | R | N | P | Y | V | L | T | T | Y | G | E | L | T | V | A | T | F | S | A | S | A | L | A | D | Q | A | L | E | A |
| 136 UniRef90\_A0A1H3JL41\_4\_373 | R | A | R | P | W | L | L | S | - | - | - | G | A | - | - | - | - | A | A | A | A | E | D | P | Y | V | L | A | G | Y | G | E | L | V | A | G | V | R | A | A | G | L | L | A | D | H | G | T | A | A |
| 137 UniRef90\_A0A507AW89\_49\_427 | S | T | R | A | W | P | Y | G | - | - | - | G | D | N | - | - | K | E | R | P | Q | D | E | F | Y | I | L | S | T | Y | G | N | F | L | A | H | L | R | A | V | T | A | L | A | D | K | A | G | E | E |
| 138 UniRef90\_A0A1X1SEN2\_20\_406 | R | S | R | P | W | V | E | T | - | - | - | N | Y | - | - | - | - | A | S | A | Q | A | D | P | L | V | L | E | R | F | G | Q | L | W | T | L | V | Q | S | A | E | A | L | T | A | R | A | L | A | A |
| 139 UniRef90\_A0A2D3UFX4\_26\_403 | G | H | S | P | S | L | P | A | - | - | - | W | P | - | - | - | - | A | S | S | P | Q | D | P | Q | V | L | T | A | Y | G | E | L | T | V | L | T | R | S | A | S | A | L | A | D | Q | A | Q | E | A |
| 140 UniRef90\_A0A089X0S9\_26\_403 | G | H | A | P | W | H | P | A | - | - | - | W | P | - | - | - | - | V | G | S | P | H | D | P | Q | V | L | T | T | F | G | E | L | T | V | L | V | R | A | A | S | A | L | A | D | Q | A | L | A | A |
| 141 UniRef90\_A0A2W5T1D0\_29\_413 | T | T | R | A | W | P | Y | G | - | - | - | G | D | E | - | - | K | Q | R | A | S | E | E | W | Y | I | L | E | G | Y | G | G | L | Q | S | K | L | W | A | D | A | A | L | I | D | A | V | G | D | E |
| 142 UniRef90\_A0A1C4QGR9\_7\_388 | R | S | R | P | W | F | E | S | - | - | - | G | A | - | - | - | - | D | T | A | A | E | D | P | L | V | I | Q | R | F | G | E | L | G | V | T | T | R | A | S | R | A | L | L | R | E | A | A | R | A |
| 143 UniRef90\_A0A101R4L6\_7\_388 | R | S | R | P | W | F | E | S | - | - | - | G | A | - | - | - | - | D | T | A | V | E | D | P | L | L | V | Q | R | F | G | E | L | A | L | R | V | R | A | S | E | A | L | L | K | E | A | A | R | A |
| 144 UniRef90\_A0A4D4LHU8\_7\_388 | K | S | R | P | W | F | E | S | - | - | - | G | A | - | - | - | - | E | T | A | A | E | D | P | L | L | I | Q | R | F | G | E | L | A | I | Q | V | R | A | S | D | A | L | L | V | A | A | A | R | A |
| 145 UniRef90\_L1KK35\_6\_389 | K | S | R | P | W | F | E | S | V | D | E | G | H | - | - | - | - | E | T | A | A | E | D | P | L | L | I | Q | R | F | G | E | L | A | I | R | V | R | A | A | E | A | L | L | R | E | A | A | R | A |
| 146 UniRef90\_UPI0005601C6B\_13\_390 | K | S | R | P | W | F | E | S | - | - | - | G | F | - | - | - | - | E | T | A | A | E | D | P | L | L | I | Q | R | F | G | E | L | S | L | A | V | R | S | S | D | A | L | L | R | A | A | A | D | A |
| 147 UniRef90\_A0A1H9UQJ9\_6\_361 | K | T | R | P | W | F | V | S | - | - | - | G | V | - | - | - | - | E | A | A | T | E | D | P | Y | I | V | V | A | Y | G | E | M | M | S | Q | T | K | A | A | G | L | L | V | D | E | A | S | R | Q |
| 148 UniRef90\_A0A3D9JKY9\_12\_401 | V | R | S | P | W | Q | P | F | - | - | - | S | P | E | P | W | P | D | S | P | P | Q | D | P | Y | A | L | T | V | Y | G | E | F | A | V | A | A | R | A | A | S | A | L | A | D | Q | A | V | E | A |
| 149 UniRef90\_D9X7Q2\_26\_403 | G | L | S | H | W | H | P | D | - | - | - | W | P | - | - | - | - | V | G | S | P | Q | D | P | Q | V | L | T | A | Y | G | E | L | T | V | L | T | R | S | A | S | A | L | A | D | Q | A | R | Q | A |
| 150 UniRef90\_A0A1X1DXJ3\_20\_398 | H | S | R | P | W | V | D | A | - | - | - | G | V | - | - | - | - | A | R | N | A | D | D | P | Y | I | L | A | D | I | G | R | I | S | T | E | L | S | A | A | N | A | L | L | R | R | A | A | R | V |

  
  

|  |  |  |  |  |  |  |  |  |  |  |  |  |  |  |  |  |  |  |  |  |  |  |  |  |  |  |  |  |  |  |  |  |  |  |  |  |  |  |  |  |  |  |  |  |  |  |  |  |  |  |
| --- | --- | --- | --- | --- | --- | --- | --- | --- | --- | --- | --- | --- | --- | --- | --- | --- | --- | --- | --- | --- | --- | --- | --- | --- | --- | --- | --- | --- | --- | --- | --- | --- | --- | --- | --- | --- | --- | --- | --- | --- | --- | --- | --- | --- | --- | --- | --- | --- | --- | --- |
| **001 Input\_pdb\_SEQRES\_A** | L | Q | T | - | V | W | D | K | - | - | - | - | - | - | - | G | - | D | A | L | T | P | E | D | R | G | E | L | M | V | K | V | S | G | V | K | A | L | A | T | N | A | A | L | N | I | S | S | G | V |
| 002 UniRef90\_Q1W1G3\_1\_416 | L | Q | T | - | V | W | D | K | - | - | - | - | - | - | - | G | - | D | A | L | A | P | E | D | R | G | E | L | M | V | K | I | P | G | V | K | A | L | A | T | N | A | A | L | D | V | N | S | G | I |
| 003 UniRef90\_T0BM21\_7\_392 | I | D | A | - | A | W | E | K | - | - | - | - | - | - | - | E | - | F | S | L | T | S | D | E | R | G | R | V | A | V | H | T | A | A | A | N | A | L | A | S | K | V | A | L | N | V | T | S | Q | V |
| 004 UniRef90\_UPI0002AC58FE\_11\_391 | L | Q | N | - | A | W | E | Q | - | - | - | - | - | - | - | E | - | W | N | L | T | A | E | Q | R | G | E | C | A | I | A | I | A | T | A | K | V | A | A | T | K | V | G | L | D | I | T | S | Q | I |
| 005 UniRef90\_A0A3D5CW51\_20\_401 | L | D | I | - | A | W | A | K | - | - | - | - | - | - | - | G | - | L | A | L | T | E | E | D | R | G | L | L | A | T | S | I | S | A | A | K | V | L | A | T | R | T | G | L | D | I | T | S | R | M |
| 006 UniRef90\_A0A2V4SPP5\_16\_400 | L | D | A | - | A | I | G | K | - | - | - | - | - | - | - | G | - | D | A | V | T | A | R | E | R | G | V | V | A | I | A | V | A | E | A | K | V | L | A | H | R | A | A | I | E | I | S | N | Q | M |
| 007 UniRef90\_E0UIV5\_8\_393 | L | Q | A | - | T | W | E | Q | - | - | - | - | - | - | - | E | - | W | L | L | T | A | S | Q | R | G | E | C | A | V | A | I | A | T | A | K | V | A | A | T | R | V | G | L | E | V | T | N | R | I |
| 008 UniRef90\_A0A1Z4S897\_9\_391 | A | Q | A | - | A | W | E | K | - | - | - | - | - | - | - | G | - | E | E | L | T | A | A | E | R | G | E | L | A | V | S | V | A | S | A | K | V | L | S | T | R | V | A | L | D | V | T | S | K | I |
| 009 UniRef90\_UPI00045E9273\_21\_396 | L | D | A | - | A | W | R | Q | - | - | - | - | - | - | - | E | - | N | A | L | T | E | A | E | R | G | S | V | A | I | A | S | A | T | A | K | V | L | A | T | R | A | G | L | D | V | T | Q | R | L |
| 010 UniRef90\_A0A353Y4M9\_17\_384 | L | E | A | - | A | W | Q | R | - | - | - | - | - | - | - | G | - | P | Q | L | M | A | E | E | R | G | A | V | S | V | A | V | A | T | A | K | V | L | A | H | R | A | S | L | D | I | S | S | R | L |
| 011 UniRef90\_F3KR10\_17\_384 | I | E | A | - | A | W | Q | R | - | - | - | - | - | - | - | G | - | E | A | L | T | A | E | E | R | G | A | A | S | V | A | V | A | T | A | K | V | V | A | A | R | A | A | L | D | I | G | S | R | L |
| 012 UniRef90\_A0A2D8NW56\_20\_404 | A | Q | R | - | H | F | A | Q | - | - | - | - | - | - | - | G | - | D | A | I | T | A | A | G | R | G | E | T | A | V | A | I | A | E | A | K | V | A | A | H | Q | A | V | L | D | I | T | S | R | L |
| 013 UniRef90\_A0A2W7M9P1\_16\_386 | L | D | K | - | T | W | A | L | - | - | - | - | - | - | - | K | - | H | E | V | T | F | E | Q | R | G | E | C | S | I | A | V | A | T | T | K | V | Q | V | V | Q | T | A | L | D | V | T | S | R | I |
| 014 UniRef90\_A0A398AYR9\_12\_386 | L | D | N | - | T | W | A | L | - | - | - | - | - | - | - | K | - | T | S | I | T | E | E | Q | R | G | E | C | S | I | A | I | A | T | A | K | V | Q | V | V | Q | T | A | L | D | V | T | S | R | V |
| 015 UniRef90\_A0A0T6UXN9\_11\_394 | L | D | E | - | A | W | R | K | - | - | - | - | - | - | - | E | - | H | A | L | T | A | E | E | R | A | Q | L | A | L | T | I | G | T | A | K | V | A | A | S | R | N | A | L | D | I | C | N | R | L |
| 016 UniRef90\_A0A252E884\_11\_390 | V | Q | Q | - | A | W | D | K | - | - | - | - | - | - | - | G | - | S | D | L | T | F | E | E | R | G | E | V | A | I | A | V | F | S | A | K | A | F | V | T | R | V | G | L | D | I | S | N | G | I |
| 017 UniRef90\_A0A1Z4BZ71\_24\_403 | L | D | N | - | A | W | G | K | - | - | - | - | - | - | - | G | - | L | A | L | T | D | A | E | R | G | E | V | A | L | A | V | F | S | A | K | V | N | S | T | K | A | G | L | D | V | T | S | R | I |
| 018 UniRef90\_A0A1Z4IGC5\_10\_387 | F | Q | A | - | A | W | E | R | - | - | - | - | - | - | - | G | - | R | Q | L | T | A | D | E | F | G | K | T | A | V | A | I | S | T | A | A | A | F | T | T | K | V | G | L | E | I | T | N | Q | V |
| 019 UniRef90\_A0A1W9JD13\_18\_401 | L | D | E | - | A | W | Q | K | - | - | - | - | - | - | - | D | - | S | N | L | T | E | N | E | R | G | R | V | A | T | C | I | S | A | A | K | V | M | A | T | R | T | G | L | D | I | T | S | R | M |
| 020 UniRef90\_A0A0B6S5D2\_8\_393 | L | Q | G | - | A | W | D | R | - | - | - | - | - | - | - | G | - | Q | A | L | T | A | E | A | R | A | E | V | S | L | A | V | S | E | A | K | V | V | A | Q | R | A | A | L | E | S | G | E | A | L |
| 021 UniRef90\_A0A1Y3C786\_14\_396 | L | Q | Q | - | A | W | D | I | - | - | - | - | - | - | - | A | - | E | N | L | T | E | A | Q | R | G | E | V | A | L | A | V | A | T | A | K | V | A | A | T | R | T | S | L | D | I | S | N | R | I |
| 022 UniRef90\_A0A318KD41\_20\_397 | L | D | Q | - | A | W | R | Q | - | - | - | - | - | - | - | G | - | E | A | L | T | A | D | E | R | G | E | L | A | I | S | I | A | T | A | K | V | A | T | N | Q | V | G | L | E | L | T | S | R | M |
| 023 UniRef90\_A0A352JDP6\_15\_389 | L | Q | D | - | A | W | S | Q | - | - | - | - | - | - | - | E | - | W | D | L | T | A | Q | Q | R | G | E | T | A | I | A | I | A | T | A | K | V | S | A | H | K | V | G | L | E | V | T | N | R | I |
| 024 UniRef90\_A0A329B538\_13\_401 | L | D | D | - | A | W | A | C | - | - | - | - | - | - | - | G | - | D | A | L | T | A | D | E | R | G | R | L | A | V | S | I | A | S | A | K | V | A | A | T | R | I | G | L | D | V | C | T | R | L |
| 025 UniRef90\_A0A2N8QAF3\_13\_394 | L | E | D | - | A | W | Q | K | - | - | - | - | - | - | - | G | - | P | A | L | D | A | E | T | R | A | R | V | A | L | A | T | S | E | A | K | I | V | A | H | R | A | A | L | D | V | S | E | K | L |
| 026 UniRef90\_A0A2N7XWT8\_11\_394 | L | D | A | - | A | W | A | K | - | - | - | - | - | - | - | G | - | S | A | L | T | S | E | E | R | G | E | T | A | I | A | I | A | T | A | K | V | A | A | S | R | N | G | L | E | L | C | S | K | V |
| 027 UniRef90\_A0A2A4HLB2\_33\_412 | A | Q | R | - | H | F | V | K | - | - | - | - | - | - | - | R | - | T | A | I | S | A | E | N | R | G | E | A | A | I | A | I | A | E | A | K | V | V | A | H | Q | A | A | L | D | A | S | S | R | L |
| 028 UniRef90\_A0A4R3HWL3\_41\_416 | L | Q | Q | - | A | W | E | R | - | - | - | - | - | - | - | G | - | D | A | L | T | A | D | E | R | G | R | L | A | V | T | I | Y | E | A | K | L | V | A | A | R | A | A | L | E | V | T | S | K | I |
| 029 UniRef90\_A0A1M7NXP6\_14\_394 | L | E | R | - | V | F | R | L | - | - | - | - | - | - | - | G | - | S | A | V | T | A | Q | Q | R | G | E | L | A | I | S | V | A | E | A | K | V | L | S | H | R | A | G | V | E | V | S | S | E | I |
| 030 UniRef90\_A0A1B4ESK2\_22\_397 | L | Q | H | - | A | W | A | R | - | - | - | - | - | - | - | R | - | D | A | L | T | A | D | E | R | A | E | V | A | L | A | V | S | E | A | K | I | V | A | Q | R | A | A | L | D | N | G | E | A | L |
| 031 UniRef90\_A0A1H6NKV2\_11\_394 | L | D | Q | - | A | W | A | K | - | - | - | - | - | - | - | G | - | P | E | L | S | A | E | E | R | G | Q | L | A | I | A | I | A | T | A | K | V | A | A | S | R | Q | G | L | E | L | C | S | K | L |
| 032 UniRef90\_A0A0D0KUF8\_11\_394 | L | D | E | - | A | W | S | K | - | - | - | - | - | - | - | E | - | H | A | L | S | A | E | E | R | A | R | L | A | I | S | I | S | T | A | K | V | A | A | S | R | T | G | L | D | I | C | N | R | L |
| 033 UniRef90\_A0A0F3K7B4\_20\_401 | M | V | A | - | A | L | A | R | - | - | - | - | - | - | - | G | - | E | A | L | T | Q | D | E | R | G | E | V | A | M | H | I | A | A | A | K | V | A | A | T | R | M | G | L | N | L | A | C | Q | M |
| 034 UniRef90\_A0A381IMG6\_13\_394 | L | Q | A | - | A | W | A | K | - | - | - | - | - | - | - | K | - | E | A | L | T | A | D | A | R | A | D | V | A | L | A | V | S | E | A | K | I | V | A | Q | R | A | A | L | D | V | S | E | A | L |
| 035 UniRef90\_A0A2S9K1G1\_26\_400 | I | D | Q | - | A | L | A | L | - | - | - | - | - | - | - | G | - | D | A | V | T | A | A | D | R | G | R | V | A | I | A | V | A | E | A | K | A | V | A | H | R | A | A | V | E | V | S | S | Q | I |
| 036 UniRef90\_A0A1W6L7W5\_17\_392 | L | D | Q | - | A | F | R | R | - | - | - | - | - | - | - | G | - | A | A | L | T | A | A | D | R | G | E | V | A | L | A | V | A | E | A | K | V | L | A | H | R | A | A | I | E | V | S | S | Q | L |
| 037 UniRef90\_A0A255HJE3\_14\_398 | L | D | T | - | V | L | R | K | - | - | - | - | - | - | - | G | - | A | L | V | T | A | Q | E | R | G | L | L | A | V | A | V | A | E | A | K | C | L | S | H | K | A | G | L | E | I | S | S | Q | M |
| 038 UniRef90\_A0A0R3AD45\_11\_394 | L | D | Q | - | A | W | A | Q | - | - | - | - | - | - | - | G | - | P | N | L | S | E | N | E | R | G | Q | L | A | I | A | I | A | T | A | K | V | A | A | S | R | Q | G | L | E | L | C | S | R | L |
| 039 UniRef90\_A0A1W6ZB60\_16\_389 | L | D | A | - | A | L | A | R | - | - | - | - | - | - | - | G | - | D | A | V | S | A | A | E | R | G | E | V | A | V | A | G | A | E | A | K | V | W | A | H | R | A | A | L | R | L | G | T | E | L |
| 040 UniRef90\_A0A2X1DPB7\_14\_397 | L | Q | H | - | A | W | A | R | - | - | - | - | - | - | - | Q | - | D | A | L | T | A | D | E | R | A | E | V | A | L | A | V | S | E | A | K | I | V | A | Q | R | A | A | L | D | N | G | E | A | L |
| 041 UniRef90\_A0A0S9M2D1\_4\_380 | L | D | E | - | A | W | R | R | - | - | - | - | - | - | - | G | - | E | Q | L | D | A | A | T | R | G | E | A | A | V | A | I | A | A | A | K | V | A | A | T | R | V | G | L | D | L | C | T | R | M |
| 042 UniRef90\_A0A238ZKW1\_13\_401 | F | D | A | - | A | W | E | Q | - | - | - | - | - | - | - | Q | - | A | S | L | S | E | E | A | R | G | K | V | A | I | E | I | G | A | A | K | V | V | T | T | R | I | G | L | D | I | T | S | R | M |
| 043 UniRef90\_A0A0D1P8V6\_10\_394 | L | D | R | - | A | W | A | K | - | - | - | - | - | - | - | G | - | A | A | L | G | A | D | Q | R | G | R | V | A | V | A | I | A | T | A | K | V | A | A | S | R | D | G | L | E | L | C | S | K | V |
| 044 UniRef90\_A0A261SP68\_11\_387 | L | D | A | - | A | L | G | R | - | - | - | - | - | - | - | G | - | A | A | L | T | A | A | E | R | G | E | L | A | V | A | I | A | E | A | K | I | W | S | H | R | A | A | L | K | V | G | N | E | L |
| 045 UniRef90\_A0A2N6MRB2\_11\_390 | V | Q | Q | - | A | W | E | K | - | - | - | - | - | - | - | E | - | T | S | L | T | F | E | E | R | G | E | V | A | I | A | V | F | T | A | K | T | F | A | T | R | V | G | L | E | I | T | N | R | I |
| 046 UniRef90\_A0A212BVA1\_31\_405 | L | Q | Q | - | A | W | D | K | - | - | - | - | - | - | - | - | - | P | A | L | S | A | A | E | R | G | E | V | A | L | L | I | A | E | A | R | V | S | S | A | R | A | S | L | E | I | T | S | Q | V |
| 047 UniRef90\_A0A1A9KE82\_11\_394 | F | D | A | - | A | W | G | K | - | - | - | - | - | - | - | E | - | Q | R | L | G | A | E | E | R | A | E | L | A | I | A | I | G | T | A | K | V | A | A | T | R | V | G | L | E | L | C | S | R | L |
| 048 UniRef90\_A0A4Q5PVB9\_26\_400 | F | Q | W | - | A | W | D | R | - | - | - | - | - | - | - | G | - | R | D | L | T | E | A | E | R | G | E | A | A | L | L | V | A | A | A | R | T | T | A | A | K | A | A | L | Q | V | T | A | D | I |
| 049 UniRef90\_A0A178GPM9\_9\_392 | L | Q | A | - | A | W | E | L | - | - | - | - | - | - | - | G | - | N | D | L | S | A | E | Q | R | G | E | V | S | V | A | I | A | T | A | K | I | A | A | T | N | T | S | L | Y | I | T | Q | N | I |
| 050 UniRef90\_A0A1P9YC32\_3\_390 | F | D | G | - | A | W | R | R | - | - | - | - | - | - | - | G | - | L | A | L | T | E | G | E | R | G | E | V | A | V | A | V | A | A | S | K | V | A | A | A | R | A | G | L | D | V | A | H | R | M |
| 051 UniRef90\_UPI000A1773A9\_40\_416 | L | D | L | - | A | L | A | R | - | - | - | - | - | - | - | G | - | T | A | L | T | A | D | E | R | G | T | L | A | V | S | I | A | T | A | K | V | A | A | T | R | T | G | L | D | I | C | N | R | L |
| 052 UniRef90\_A0A158L201\_25\_395 | L | D | A | - | A | W | E | R | - | - | - | - | - | - | - | G | - | D | S | L | E | A | G | E | R | A | E | V | A | L | A | V | A | E | A | K | V | L | A | H | R | A | A | L | D | V | S | Q | S | L |
| 053 UniRef90\_A0A4Q4GT15\_13\_391 | F | Q | S | - | A | W | D | I | - | - | - | - | - | - | - | G | - | E | G | L | N | A | E | Q | R | G | E | V | S | I | A | I | A | T | A | K | I | A | A | T | N | T | S | L | F | V | T | Q | N | I |
| 054 UniRef90\_A0A1C0YC62\_17\_381 | L | V | T | - | T | L | H | K | - | - | - | - | - | - | - | K | - | H | H | V | T | E | E | D | R | G | I | C | S | V | A | I | A | T | A | K | V | Q | V | V | E | T | T | L | H | I | T | S | T | M |
| 055 UniRef90\_A0A1H7FE04\_32\_405 | L | H | H | - | A | L | L | R | - | - | - | - | - | - | - | G | - | D | A | L | T | A | Q | E | R | G | E | L | A | V | A | G | A | E | A | K | V | L | A | H | R | A | A | L | Q | V | S | S | Q | L |
| 056 UniRef90\_A0A401MWC4\_5\_385 | V | Q | R | - | G | L | D | R | - | - | - | - | - | - | - | G | - | P | D | L | T | A | D | E | R | G | E | I | A | V | T | V | A | T | A | K | V | A | A | T | K | A | A | L | D | I | T | A | R | I |
| 057 UniRef90\_A0A1G6HFW7\_16\_395 | F | D | S | - | A | W | R | R | - | - | - | - | - | - | - | E | - | T | A | L | T | E | T | E | R | G | K | V | A | I | A | V | A | A | A | K | V | Q | S | A | Q | A | G | L | D | V | T | Q | R | M |
| 058 UniRef90\_A0A0P9B999\_30\_403 | F | Q | Q | - | A | W | E | Q | - | - | - | - | - | - | - | G | - | F | A | L | T | A | D | S | R | G | A | L | A | I | D | V | A | A | A | R | T | Q | A | A | R | T | A | L | H | V | T | S | Q | I |
| 059 UniRef90\_A0A140K6F0\_14\_392 | I | D | E | - | A | W | Q | K | - | - | - | - | - | - | - | G | - | V | S | L | T | F | Q | E | R | G | S | I | A | I | A | V | S | A | A | K | A | F | A | A | K | V | G | L | N | I | T | T | R | M |
| 060 UniRef90\_A0A1P8EKI7\_5\_392 | L | Q | N | - | A | W | N | E | - | - | - | - | - | - | - | G | - | P | D | L | S | A | Q | Q | R | G | E | V | S | V | A | V | A | T | A | K | I | A | A | T | R | T | G | L | M | L | T | Q | D | M |
| 061 UniRef90\_A0A2U3MYZ3\_7\_392 | I | Q | W | - | A | W | N | I | - | - | - | - | - | - | - | G | - | E | N | L | T | A | E | Q | R | G | K | V | S | I | A | I | A | T | A | K | I | A | A | T | N | S | S | L | F | I | T | Q | N | I |
| 062 UniRef90\_A0A1H2EPX5\_7\_397 | L | R | H | - | T | W | E | K | - | - | - | - | - | - | - | G | - | E | N | L | T | E | A | E | R | G | E | A | A | L | D | I | A | A | A | K | I | A | T | T | R | A | A | L | D | I | P | S | K | I |
| 063 UniRef90\_UPI00041EF43F\_25\_400 | L | D | Q | - | A | L | A | L | - | - | - | - | - | - | - | G | - | E | D | V | T | A | S | D | R | G | E | L | A | V | A | V | A | Q | A | K | A | V | A | H | R | A | A | I | D | V | S | T | Q | V |
| 064 UniRef90\_D0IW93\_17\_384 | L | Q | K | - | A | W | Q | R | - | - | - | - | - | - | - | G | - | P | A | L | S | A | A | E | R | A | E | V | A | I | A | V | F | E | A | K | V | M | A | H | R | C | T | L | F | A | T | Q | E | M |
| 065 UniRef90\_A0A395D1I6\_11\_388 | F | Q | Q | - | G | W | E | A | - | - | - | - | - | - | - | G | - | A | D | T | T | P | E | L | R | G | D | I | A | V | T | I | A | A | A | K | V | A | S | S | R | A | S | V | E | V | G | S | R | I |
| 066 UniRef90\_A0A1H0TK45\_27\_400 | F | D | Q | - | A | W | A | S | - | - | - | - | - | - | - | G | - | T | A | L | T | E | V | E | R | G | A | V | A | I | A | V | A | A | A | K | V | A | T | T | R | A | S | L | D | V | V | H | R | M |
| 067 UniRef90\_A0A1H8MYU5\_23\_403 | M | D | S | - | A | Y | A | K | - | - | - | - | - | - | - | G | - | G | A | L | T | A | Q | E | R | G | A | T | A | I | A | V | A | S | A | N | V | L | A | G | N | V | A | L | E | V | T | S | E | I |
| 068 UniRef90\_A0A1B1M254\_5\_403 | G | Q | K | - | L | H | D | D | - | - | - | - | - | - | - | P | - | D | A | V | T | E | Q | A | R | G | D | F | E | V | R | V | A | A | V | K | A | R | A | T | D | V | S | L | E | V | A | G | R | V |
| 069 UniRef90\_A0A484THM3\_28\_401 | F | Q | A | - | A | W | D | S | - | - | - | - | - | - | - | G | - | A | A | F | D | F | D | A | R | G | E | L | G | M | L | V | A | A | A | R | V | Q | A | G | R | T | A | L | R | V | T | E | Q | I |
| 070 UniRef90\_A0A0Q8Q8X6\_8\_377 | I | E | E | - | A | F | L | L | - | - | - | - | - | - | - | G | - | R | D | L | G | G | Q | Q | R | A | A | V | A | I | A | I | A | Q | A | K | V | V | C | H | R | A | G | L | H | A | A | Q | E | L |
| 071 UniRef90\_A0A0Q5QDL2\_32\_410 | Q | Q | K | - | I | H | D | N | - | - | - | - | - | - | - | A | - | W | D | V | T | A | S | E | R | G | A | H | E | V | R | V | A | A | A | K | A | A | A | T | D | V | A | L | E | I | T | T | R | I |
| 072 UniRef90\_A0A1A5XM62\_22\_406 | L | D | A | - | A | W | A | Q | - | - | - | - | - | - | - | G | - | D | A | L | G | A | D | T | R | A | E | V | S | L | A | V | S | E | A | K | I | V | A | H | R | A | A | L | A | A | G | E | Q | L |
| 073 UniRef90\_A0A239EI90\_21\_397 | A | N | E | - | I | H | E | D | - | - | - | - | - | - | - | A | - | W | N | V | T | P | E | Q | R | G | D | H | E | V | R | V | A | A | V | K | A | R | A | S | E | V | A | L | E | I | T | S | T | V |
| 074 UniRef90\_S5SWW2\_23\_403 | M | D | A | - | A | F | A | K | - | - | - | - | - | - | - | G | - | G | A | L | K | A | E | E | R | G | K | T | A | I | A | V | A | A | A | N | V | L | A | G | K | V | A | L | E | V | T | S | E | I |
| 075 UniRef90\_A0A3A5JJY9\_31\_413 | V | D | A | - | L | F | F | R | - | - | - | - | - | - | - | D | - | G | S | I | S | A | A | E | R | G | D | A | A | I | A | V | A | E | A | K | V | A | A | H | D | I | S | L | E | A | G | S | E | L |
| 076 UniRef90\_A0A315ZU85\_7\_384 | L | T | A | - | A | D | A | A | - | - | - | - | - | - | - | G | - | D | A | L | T | W | D | E | R | G | E | L | A | E | L | V | A | A | L | K | V | V | S | S | D | L | A | V | E | A | T | S | A | V |
| 077 UniRef90\_A0A3R9U899\_5\_381 | G | Q | K | - | L | H | D | D | - | - | - | - | - | - | - | P | - | D | A | V | T | E | Q | A | R | G | D | F | E | V | R | V | A | A | V | K | A | R | A | T | D | V | A | L | E | V | T | N | R | I |
| 078 UniRef90\_UPI000DD53D91\_7\_389 | L | D | A | - | A | Y | G | E | - | - | - | - | - | - | - | G | - | P | L | L | T | A | A | G | R | G | A | A | A | I | D | I | A | T | A | N | V | Y | A | G | D | V | G | L | A | V | S | S | E | V |
| 079 UniRef90\_UPI000DE4BE09\_13\_396 | V | Q | A | - | G | L | L | R | - | - | - | - | - | - | - | G | - | E | Q | V | T | A | R | E | R | G | E | A | A | A | E | A | Y | A | A | K | V | H | A | T | H | T | S | L | D | V | T | S | R | V |
| 080 UniRef90\_A0A2T0R7P7\_10\_381 | L | S | Q | - | A | H | A | R | - | - | - | - | - | - | - | G | - | A | D | L | T | W | A | E | R | G | E | V | A | E | E | I | A | A | L | K | V | V | S | S | D | L | A | V | E | A | T | S | A | I |
| 081 UniRef90\_A0A4R1HYS3\_27\_404 | G | L | A | - | I | H | H | D | - | - | - | - | - | - | - | A | - | W | N | V | T | P | E | Q | R | G | A | H | E | V | R | V | A | A | V | K | A | R | A | T | D | V | A | L | E | I | T | S | G | I |
| 082 UniRef90\_UPI000D1537B0\_7\_385 | L | D | A | - | L | W | E | A | - | - | - | - | - | - | - | G | - | Q | D | L | T | E | A | F | R | A | E | Q | A | C | T | I | A | A | A | N | V | L | A | G | E | V | A | L | H | V | T | S | A | V |
| 083 UniRef90\_A0A1G6ZNM7\_17\_409 | I | E | S | - | I | N | A | H | - | - | - | - | - | - | - | A | - | D | A | V | T | P | E | E | R | G | E | A | A | V | V | I | A | S | A | K | Q | T | T | I | D | A | G | L | E | I | G | T | R | I |
| 084 UniRef90\_UPI0003765980\_18\_389 | L | D | A | - | A | Y | I | Q | - | - | - | - | - | - | - | G | - | P | S | L | S | H | A | D | R | G | A | A | A | I | D | I | A | T | A | N | L | Y | A | G | E | V | G | L | A | V | S | S | E | V |
| 085 UniRef90\_A0A071ICK8\_25\_398 | V | Q | A | - | A | L | S | R | - | - | - | - | - | - | - | G | - | K | D | V | T | A | R | E | R | G | E | A | A | A | E | A | Y | A | A | K | V | H | A | T | N | V | S | L | D | I | T | S | R | V |
| 086 UniRef90\_A0A2L0WNE5\_33\_396 | V | Q | T | - | E | L | A | K | - | - | - | - | - | - | - | G | - | H | S | V | T | A | R | E | R | G | A | A | A | A | E | A | Y | A | A | K | V | H | A | T | N | I | S | L | D | I | T | S | R | V |
| 087 UniRef90\_A0A1Q8CFJ2\_9\_380 | L | G | A | - | A | F | D | R | - | - | - | - | - | - | - | G | - | D | A | L | S | E | Q | E | R | G | E | A | A | V | T | I | S | A | A | K | V | V | A | T | R | V | A | N | E | A | G | S | K | I |
| 088 UniRef90\_UPI000835F605\_21\_394 | Y | Q | A | - | A | L | L | A | - | - | - | - | - | - | - | G | - | P | A | L | T | A | Q | A | W | G | E | L | A | L | L | I | D | Q | A | K | V | I | A | T | K | V | S | L | E | V | T | A | D | I |
| 089 UniRef90\_UPI000374AB86\_31\_398 | V | Q | N | - | A | L | A | R | - | - | - | - | - | - | - | G | - | S | S | V | T | A | R | E | R | G | E | A | A | T | E | A | Y | A | A | K | V | H | A | T | N | V | S | L | D | I | T | S | R | I |
| 090 UniRef90\_A0A267RVL6\_22\_399 | F | Q | D | - | A | F | H | A | - | - | - | - | - | - | - | G | - | D | D | L | T | E | T | Q | W | G | E | L | A | L | L | T | D | Q | A | K | V | I | A | N | N | V | A | L | K | I | S | N | E | I |
| 091 UniRef90\_M3VA40\_23\_395 | L | Q | H | - | A | L | D | I | - | - | - | - | - | - | - | G | - | E | A | L | T | D | E | Q | R | A | V | A | A | V | A | I | A | E | A | K | Y | V | T | T | K | V | S | L | D | A | A | S | R | L |
| 092 UniRef90\_A0A395GID9\_32\_425 | L | A | T | - | L | Y | S | Q | H | S | D | N | - | - | - | R | - | P | G | L | T | S | Q | Q | R | G | E | V | A | E | W | I | A | S | V | K | V | V | T | T | D | V | G | L | R | V | T | S | G | V |
| 093 UniRef90\_UPI0008268C2F\_35\_406 | L | Q | H | - | A | L | N | I | - | - | - | - | - | - | - | G | - | P | E | L | T | A | H | Q | R | A | E | A | A | I | A | I | A | E | A | K | Y | V | T | T | K | V | S | L | E | V | T | S | R | L |
| 094 UniRef90\_A0A0S1XV73\_27\_397 | L | D | R | - | A | C | A | L | - | - | - | - | - | - | - | G | - | R | G | L | D | A | E | T | R | G | R | V | S | I | D | L | A | A | A | N | V | Y | A | G | E | N | A | L | D | V | T | E | K | I |
| 095 UniRef90\_A0A0F5N116\_19\_398 | L | Q | A | - | A | I | D | V | - | - | - | - | - | - | - | G | - | P | S | L | T | E | D | Q | R | A | E | A | A | I | A | I | Y | E | A | K | Y | L | S | T | K | V | A | L | D | T | A | S | R | L |
| 096 UniRef90\_A0A1N6Z7J9\_40\_406 | V | Q | A | - | A | L | S | R | - | - | - | - | - | - | - | G | - | R | N | V | T | E | R | E | R | G | E | A | A | I | E | A | Y | T | A | K | V | N | A | T | H | V | S | L | D | V | T | S | R | V |
| 097 UniRef90\_A0A2V4UWQ2\_31\_411 | V | Q | T | - | A | L | A | R | - | - | - | - | - | - | - | G | - | P | G | V | T | E | R | E | R | G | E | A | A | I | E | A | Y | E | S | K | I | N | A | T | H | V | S | L | D | L | T | A | R | V |
| 098 UniRef90\_A0A4P8KLT1\_5\_401 | F | D | E | - | V | I | S | R | - | - | - | - | - | - | - | G | - | A | D | V | T | A | E | D | R | A | E | I | A | I | E | I | A | Q | L | K | V | V | A | S | D | T | A | T | D | V | A | H | R | V |
| 099 UniRef90\_UPI0003068598\_3\_292 | L | Q | G | - | A | W | A | K | - | - | - | - | - | - | - | H | - | E | A | L | T | A | D | A | R | A | E | V | A | L | A | I | S | E | A | K | I | V | A | Q | R | A | A | L | D | V | S | E | A | L |
| 100 UniRef90\_UPI000DD585C3\_27\_403 | V | E | T | - | A | L | A | K | - | - | - | - | - | - | - | G | - | R | S | V | T | E | R | E | R | G | E | A | A | L | E | A | Y | A | A | K | V | S | A | T | H | V | A | L | D | V | T | A | K | V |
| 101 UniRef90\_A0A1E3SMN8\_25\_404 | L | Q | A | - | A | V | D | V | - | - | - | - | - | - | - | G | - | P | S | L | T | E | D | Q | R | A | E | A | A | I | A | V | Y | E | A | K | Y | L | S | T | K | V | S | L | E | T | A | S | R | L |
| 102 UniRef90\_UPI00045EBD18\_12\_396 | F | E | A | - | A | T | A | L | - | - | - | - | - | - | - | G | - | N | A | I | T | P | D | D | R | N | V | A | A | A | A | I | Y | A | A | K | V | H | S | S | R | T | A | L | E | I | T | S | R | I |
| 103 UniRef90\_A0A1Y1ZNX6\_42\_434 | V | D | K | - | I | Y | S | S | Y | A | G | E | A | G | I | R | - | A | K | L | T | A | R | E | R | G | E | L | A | E | W | V | A | S | T | K | V | V | T | T | D | T | G | L | R | V | T | A | G | V |
| 104 UniRef90\_A0A0D2FPI6\_31\_423 | I | R | D | - | V | Y | A | D | H | G | E | K | - | - | - | R | - | - | D | V | S | A | R | R | R | G | E | V | A | E | W | V | A | S | I | K | I | V | A | T | E | T | S | L | R | V | T | S | G | V |
| 105 UniRef90\_A0A2S0KGS2\_30\_403 | L | Q | Q | - | A | L | D | F | - | - | - | - | - | - | - | G | - | P | E | L | S | A | E | Q | R | A | E | A | A | I | A | I | A | E | A | K | Y | L | T | T | K | V | S | L | E | A | A | S | R | L |
| 106 UniRef90\_A0A1A7MGQ0\_54\_442 | I | S | D | - | I | F | L | T | H | G | G | K | - | - | - | R | - | - | D | V | S | V | R | Q | R | G | E | V | A | E | W | V | A | S | A | K | V | V | T | T | D | T | S | L | R | V | T | V | G | V |
| 107 UniRef90\_UPI000413001D\_18\_392 | L | E | G | - | A | I | A | R | - | - | - | - | - | - | - | G | - | H | D | L | T | V | P | E | R | H | A | A | A | A | A | I | Y | A | A | K | V | Q | S | S | R | A | A | L | D | I | T | S | R | I |
| 108 UniRef90\_G7H5P9\_11\_400 | L | Q | E | - | S | L | D | F | - | - | - | - | - | - | - | G | - | P | E | L | T | A | E | Q | R | A | A | T | A | V | A | I | A | E | A | K | Y | L | T | T | K | V | S | L | E | V | A | S | R | L |
| 109 UniRef90\_A0A010YHQ5\_11\_404 | W | M | A | - | A | A | S | R | - | - | - | - | - | - | - | G | - | W | E | L | T | H | A | E | R | G | E | V | S | V | E | L | S | A | V | K | V | V | T | T | R | A | A | L | D | A | T | Q | G | V |
| 110 UniRef90\_B0RBU7\_24\_404 | F | D | G | - | V | V | D | L | - | - | - | - | - | - | - | G | - | D | G | V | T | A | E | I | R | G | R | I | A | I | D | V | A | K | L | K | V | V | A | T | E | V | G | V | E | V | A | N | R | V |
| 111 UniRef90\_A0A167RW81\_42\_429 | L | S | A | - | L | Y | A | K | H | A | A | D | - | - | - | R | - | A | G | L | T | T | A | A | R | G | D | V | A | E | W | I | A | S | A | K | V | V | T | T | D | T | G | L | R | V | T | A | G | V |
| 112 UniRef90\_A0A1X0J8A0\_26\_405 | F | E | Q | - | A | L | G | A | - | - | - | - | - | - | - | G | - | E | T | P | N | A | A | Q | W | G | A | L | A | I | R | V | D | Q | A | K | S | I | A | T | E | V | S | L | D | V | T | H | N | I |
| 113 UniRef90\_A0A2J6Q859\_43\_430 | I | S | A | - | V | Y | A | A | H | S | T | D | - | - | - | R | - | S | S | L | T | A | R | A | R | G | E | L | A | E | E | V | A | S | V | K | V | V | T | T | D | T | G | L | R | V | T | S | G | I |
| 114 UniRef90\_UPI0003828F02\_7\_389 | F | T | A | - | A | A | A | R | - | - | - | - | - | - | - | G | - | D | G | L | T | A | T | E | R | G | E | T | S | L | R | I | S | A | A | K | T | V | T | S | E | L | A | T | E | I | A | S | R | V |
| 115 UniRef90\_A0A1B2HDJ6\_6\_361 | L | W | E | - | A | S | E | L | - | - | - | - | - | - | - | G | - | K | N | L | T | P | Q | K | R | A | Q | V | A | V | E | I | S | A | A | K | V | V | S | T | K | L | V | N | E | V | S | S | R | I |
| 116 UniRef90\_A0A2N3N0U1\_47\_427 | I | D | A | - | V | Y | A | K | G | T | P | D | - | - | - | R | - | S | K | V | E | A | R | D | R | G | E | L | A | E | T | V | A | S | L | K | V | V | T | T | D | T | G | L | R | V | T | S | G | V |
| 117 UniRef90\_S5Y4X6\_32\_408 | F | Q | E | - | A | L | F | A | - | - | - | - | - | - | - | G | - | P | D | L | A | R | D | D | W | G | R | L | A | V | L | V | D | Q | A | K | V | V | A | N | D | V | S | L | E | V | T | A | R | I |
| 118 UniRef90\_A0A3R2WQN2\_17\_411 | I | S | A | L | L | H | A | E | - | - | - | - | - | - | - | R | - | D | A | L | T | P | G | A | R | G | R | V | A | V | R | I | A | A | A | K | Q | A | A | V | D | I | G | L | E | I | G | T | K | V |
| 119 UniRef90\_A0A3N1X255\_20\_392 | L | D | E | - | L | Y | P | Q | - | - | - | - | - | - | - | G | - | H | G | L | T | A | H | A | R | G | S | L | A | I | S | A | A | A | A | N | V | V | A | G | E | V | G | L | A | V | A | E | E | V |
| 120 UniRef90\_A0A1S1LA29\_28\_404 | F | E | Q | - | T | L | A | T | - | - | - | - | - | - | - | G | - | E | A | P | T | E | A | G | W | G | A | L | A | I | R | V | D | Q | A | K | S | V | A | T | E | I | S | L | D | V | T | H | N | I |
| 121 UniRef90\_A0A081GNL4\_19\_392 | L | E | H | - | C | W | N | L | - | - | - | - | - | - | - | G | - | R | G | L | T | P | V | E | R | G | D | C | A | I | A | I | A | V | T | K | V | L | A | S | E | V | A | L | A | V | G | S | A | L |
| 122 UniRef90\_A0A1I6U758\_27\_419 | L | A | Q | L | L | H | S | D | - | - | - | - | - | - | - | R | - | D | G | V | T | R | E | M | R | G | R | A | A | V | L | V | S | A | A | K | Q | R | T | I | D | V | G | L | E | I | A | N | R | I |
| 123 UniRef90\_A0A0F5VZP9\_7\_388 | V | E | A | - | A | - | - | - | - | - | - | - | - | - | - | Q | - | A | D | L | S | D | D | S | A | A | E | A | S | I | A | V | A | A | A | K | V | R | A | A | E | A | A | V | E | V | A | S | A | L |
| 124 UniRef90\_A0A1H4GIM8\_20\_411 | I | S | A | L | L | H | A | P | - | - | - | - | - | - | - | R | - | E | G | L | S | E | R | R | R | G | E | V | A | V | R | I | A | A | G | K | L | R | I | V | D | D | G | L | E | V | A | T | K | I |
| 125 UniRef90\_A0A0M8TTT1\_8\_388 | V | D | G | - | A | - | - | - | - | - | - | - | - | - | - | R | - | A | D | L | T | D | D | S | A | A | E | A | S | I | A | V | A | A | A | K | A | H | A | A | Q | T | A | V | E | V | G | S | A | L |
| 126 UniRef90\_A0A1Y2MDA3\_48\_437 | V | S | N | - | I | Y | A | A | Y | S | G | S | L | E | T | R | - | A | K | L | T | A | R | E | R | G | E | L | A | E | W | V | A | S | A | K | V | V | T | T | D | T | G | L | R | V | T | A | G | V |
| 127 UniRef90\_UPI000562F0C5\_9\_388 | V | D | A | - | A | - | - | - | - | - | - | - | - | - | - | R | - | A | D | L | T | D | D | S | A | A | E | A | S | I | A | V | A | A | A | K | V | R | A | A | R | T | A | V | E | V | A | S | A | L |
| 128 UniRef90\_A0A506Y8Z1\_43\_442 | I | S | A | V | L | H | A | P | - | - | - | - | - | - | - | R | - | G | E | L | T | E | R | R | R | G | E | I | A | V | R | I | A | A | A | K | A | R | I | V | D | D | G | L | E | A | A | T | K | V |
| 129 UniRef90\_A0A021VVW0\_28\_409 | I | T | A | - | L | H | L | D | - | - | - | - | - | - | - | P | - | A | A | L | T | P | R | Q | R | G | E | V | A | V | L | V | A | A | A | K | Q | R | A | I | D | V | G | L | E | I | G | T | R | V |
| 130 UniRef90\_E3QTS2\_46\_428 | I | D | K | - | L | Y | R | S | - | S | G | D | P | S | E | R | - | A | N | V | T | A | E | A | R | G | E | A | A | E | W | V | A | S | V | K | V | T | A | T | D | T | G | L | R | V | T | A | G | V |
| 131 UniRef90\_A0A1D8SMV7\_7\_388 | V | D | A | - | A | - | - | - | - | - | - | - | - | - | - | S | - | A | G | L | T | D | D | S | A | A | E | A | S | I | A | V | A | A | A | K | V | Q | A | A | R | T | A | V | E | V | G | S | A | L |
| 132 UniRef90\_A9BUV8\_38\_419 | V | D | A | - | A | - | - | - | - | - | - | - | - | - | - | Q | - | R | T | P | T | D | D | S | V | A | A | A | S | V | A | V | A | Q | A | K | A | L | S | T | S | A | S | L | L | A | G | S | K | L |
| 133 UniRef90\_A0A1N6TB45\_32\_407 | L | D | A | - | A | T | A | R | - | - | - | - | - | - | - | G | - | V | H | L | T | A | E | E | R | G | E | A | A | V | A | I | A | A | A | N | V | L | A | G | E | V | A | L | Q | A | S | T | G | L |
| 134 UniRef90\_A0A381IE41\_24\_406 | L | D | E | - | I | A | A | Q | - | - | - | - | - | - | - | - | - | S | E | I | T | E | D | D | V | A | R | A | S | V | A | V | G | E | A | K | V | L | T | T | E | I | A | L | L | A | S | E | K | L |
| 135 UniRef90\_A0A3M8TJ33\_18\_397 | L | D | C | - | G | L | A | R | - | - | - | - | - | - | - | G | - | E | D | L | T | D | D | E | C | A | E | I | T | M | L | A | A | A | A | E | A | A | A | A | G | A | A | Q | D | V | T | A | R | A |
| 136 UniRef90\_A0A1H3JL41\_4\_373 | L | Q | D | - | A | A | D | R | - | - | - | - | - | - | - | G | - | A | E | L | T | A | E | Q | R | G | A | A | A | A | T | I | S | A | A | K | I | V | A | T | R | T | A | N | E | T | T | A | G | I |
| 137 UniRef90\_A0A507AW89\_49\_427 | V | T | S | - | L | Y | N | N | - | S | G | S | A | E | D | R | - | S | K | V | T | A | E | A | R | G | E | A | A | V | W | V | A | S | V | K | V | V | A | T | D | T | G | L | R | V | T | S | G | L |
| 138 UniRef90\_A0A1X1SEN2\_20\_406 | V | D | E | - | V | R | D | R | - | - | - | - | - | - | - | G | - | A | G | L | T | W | E | Q | R | A | E | A | V | T | L | I | N | A | A | R | T | H | A | G | E | I | A | I | T | I | A | S | R | L |
| 139 UniRef90\_A0A2D3UFX4\_26\_403 | V | R | G | - | G | L | A | R | - | - | - | - | - | - | - | G | - | E | D | L | T | Y | D | E | Y | A | E | I | S | V | L | V | A | M | A | E | A | A | A | S | R | A | A | Q | E | S | T | A | R | A |
| 140 UniRef90\_A0A089X0S9\_26\_403 | V | C | A | - | G | L | S | L | - | - | - | - | - | - | - | G | - | D | D | L | T | F | E | E | H | A | D | I | A | V | L | V | A | M | A | E | A | A | A | A | R | A | A | Q | E | S | T | T | R | A |
| 141 UniRef90\_A0A2W5T1D0\_29\_413 | I | S | V | L | L | H | A | P | - | - | - | - | - | - | - | R | - | E | Q | L | T | E | R | S | R | G | E | V | A | V | R | I | A | A | A | K | L | R | I | S | D | D | G | L | E | T | A | T | K | V |
| 142 UniRef90\_A0A1C4QGR9\_7\_388 | V | E | E | - | A | - | - | - | - | - | - | - | - | - | - | R | - | A | D | L | T | D | D | T | A | A | E | A | S | V | A | V | A | A | A | K | A | H | A | A | R | T | A | V | E | V | A | D | A | L |
| 143 UniRef90\_A0A101R4L6\_7\_388 | V | D | A | - | A | - | - | - | - | - | - | - | - | - | - | R | - | A | G | L | T | D | D | S | A | A | E | A | S | L | A | V | A | A | A | K | V | Q | A | A | E | T | A | V | E | V | A | S | A | L |
| 144 UniRef90\_A0A4D4LHU8\_7\_388 | V | D | A | - | A | - | - | - | - | - | - | - | - | - | - | R | - | A | D | L | T | D | D | S | A | A | E | A | S | L | A | V | A | A | A | K | V | H | T | A | S | T | A | V | E | V | A | G | A | L |
| 145 UniRef90\_L1KK35\_6\_389 | V | D | D | - | A | - | - | - | - | - | - | - | - | - | - | R | - | A | D | L | T | D | D | S | A | A | E | A | S | I | A | V | A | A | A | K | V | T | A | A | E | A | A | V | E | V | G | S | A | L |
| 146 UniRef90\_UPI0005601C6B\_13\_390 | V | D | A | - | A | - | - | - | - | - | - | - | - | - | - | E | - | A | D | L | T | D | D | S | A | A | E | A | S | I | A | V | A | A | A | K | V | Q | A | A | A | A | A | V | D | T | G | S | A | L |
| 147 UniRef90\_A0A1H9UQJ9\_6\_361 | L | R | D | - | A | S | E | L | - | - | - | - | - | - | - | G | - | R | K | L | T | A | A | K | R | A | E | V | A | V | T | I | S | A | A | K | V | V | S | T | K | L | V | N | E | I | T | S | R | I |
| 148 UniRef90\_A0A3D9JKY9\_12\_401 | L | L | R | - | G | L | A | Q | - | - | - | - | - | - | - | R | - | E | D | L | D | D | E | E | C | A | E | I | T | V | L | A | S | A | A | E | A | A | A | S | R | A | A | Q | E | I | T | T | R | A |
| 149 UniRef90\_D9X7Q2\_26\_403 | V | L | D | - | G | L | A | R | - | - | - | - | - | - | - | G | - | E | D | L | G | Y | E | E | Y | A | E | I | S | V | L | V | A | M | A | E | A | A | A | S | K | A | A | Q | E | S | T | A | R | A |
| 150 UniRef90\_A0A1X1DXJ3\_20\_398 | L | D | A | - | I | - | - | - | - | - | - | - | - | - | - | D | P | A | A | L | T | A | E | N | S | A | A | A | S | I | A | V | A | E | A | K | V | L | T | T | E | I | A | L | R | A | S | E | K | L |

  
  

|  |  |  |  |  |  |  |  |  |  |  |  |  |  |  |  |  |  |  |  |  |  |  |  |  |  |  |  |  |  |  |  |  |  |  |  |  |  |  |  |  |  |  |  |  |  |  |  |  |  |  |
| --- | --- | --- | --- | --- | --- | --- | --- | --- | --- | --- | --- | --- | --- | --- | --- | --- | --- | --- | --- | --- | --- | --- | --- | --- | --- | --- | --- | --- | --- | --- | --- | --- | --- | --- | --- | --- | --- | --- | --- | --- | --- | --- | --- | --- | --- | --- | --- | --- | --- | --- |
| **001 Input\_pdb\_SEQRES\_A** | F | E | V | I | G | A | R | G | T | H | P | R | Y | G | F | D | R | F | W | R | N | V | R | T | H | S | L | H | D | P | V | S | Y | K | I | A | D | V | G | K | H | T | L | N | G | Q | Y | P | I | P |
| 002 UniRef90\_Q1W1G3\_1\_416 | F | E | V | I | G | A | R | G | T | H | P | K | Y | G | F | D | R | F | W | R | N | V | R | T | H | T | L | H | D | P | V | S | Y | K | I | A | D | V | G | K | H | T | L | N | G | Q | Y | P | I | P |
| 003 UniRef90\_T0BM21\_7\_392 | F | E | V | M | G | A | H | S | A | T | K | N | H | G | F | D | R | F | W | R | N | V | R | T | H | T | L | H | N | P | I | D | Y | K | F | K | N | I | G | N | W | Y | V | N | D | E | P | P | K | P |
| 004 UniRef90\_UPI0002AC58FE\_11\_391 | F | E | V | M | G | A | R | A | T | T | G | K | Y | G | F | D | R | Y | W | R | N | L | R | T | F | T | L | H | D | P | I | D | Y | K | V | L | D | I | G | N | W | L | L | N | E | Q | L | P | K | P |
| 005 UniRef90\_A0A3D5CW51\_20\_401 | F | E | V | A | G | S | R | A | T | H | A | G | L | R | L | D | R | H | W | R | N | L | R | T | H | T | L | H | D | P | L | D | Y | K | I | K | E | L | G | E | W | A | L | T | G | S | F | P | N | P |
| 006 UniRef90\_A0A2V4SPP5\_16\_400 | F | E | L | L | G | A | R | A | T | S | S | R | F | G | F | D | R | F | W | R | N | A | R | V | H | T | L | H | D | P | V | D | Y | K | L | R | D | L | G | R | Y | A | L | S | G | R | V | P | D | P |
| 007 UniRef90\_E0UIV5\_8\_393 | F | E | V | M | G | A | R | A | T | A | K | K | Y | G | F | D | R | Y | W | R | N | L | R | T | F | T | L | H | D | P | L | D | Y | K | L | R | D | L | G | N | W | A | L | N | D | Q | L | P | K | P |
| 008 UniRef90\_A0A1Z4S897\_9\_391 | F | E | V | T | G | A | R | S | A | A | N | K | Y | R | F | D | R | Y | W | R | N | V | R | T | H | T | L | H | D | P | V | A | Y | K | V | Y | E | V | G | N | W | V | L | N | G | E | I | P | D | F |
| 009 UniRef90\_UPI00045E9273\_21\_396 | F | E | V | T | G | A | R | A | T | S | A | K | A | G | F | D | R | Y | W | R | N | L | R | T | H | S | L | H | D | P | V | D | Y | K | L | R | D | L | G | E | W | V | L | N | A | K | P | P | T | P |
| 010 UniRef90\_A0A353Y4M9\_17\_384 | F | D | V | A | G | T | S | A | L | H | A | P | L | G | L | D | R | Y | W | R | N | A | R | T | H | T | L | H | D | P | L | D | Y | K | L | R | D | L | G | Q | W | A | L | N | G | V | V | P | A | S |
| 011 UniRef90\_F3KR10\_17\_384 | F | E | V | A | G | T | S | S | L | H | A | P | L | G | H | D | R | F | W | R | N | A | R | T | H | T | L | H | D | P | I | E | Y | K | L | R | D | L | G | N | W | A | L | N | D | V | L | P | P | P |
| 012 UniRef90\_A0A2D8NW56\_20\_404 | F | D | I | A | G | A | R | A | T | Q | R | R | F | G | F | D | R | F | W | R | N | A | R | T | H | T | L | H | D | P | V | D | Y | K | I | R | D | L | G | R | W | A | L | N | R | H | F | P | E | P |
| 013 UniRef90\_A0A2W7M9P1\_16\_386 | F | Q | V | M | G | A | R | S | T | S | A | Q | Y | N | F | D | R | Y | W | R | N | V | R | T | H | T | L | H | D | P | I | D | Y | K | I | R | D | I | G | Q | Y | T | L | N | N | I | Y | P | - | - |
| 014 UniRef90\_A0A398AYR9\_12\_386 | F | Q | M | M | G | A | R | A | T | S | A | Q | Y | N | F | D | R | Y | W | R | N | V | R | T | H | T | L | H | D | P | I | D | Y | K | I | R | D | L | G | Q | Y | T | L | N | N | Q | Y | P | - | - |
| 015 UniRef90\_A0A0T6UXN9\_11\_394 | F | E | V | T | G | A | R | A | T | H | A | S | L | R | F | D | R | F | W | R | N | L | R | T | Q | S | L | H | D | P | V | D | Y | R | I | H | E | L | G | D | W | A | L | N | G | T | R | P | T | P |
| 016 UniRef90\_A0A252E884\_11\_390 | F | E | V | T | G | T | R | S | T | A | S | K | Y | G | F | D | R | Y | W | R | D | L | R | T | F | T | L | H | D | P | V | D | Y | K | L | N | H | I | G | N | W | L | L | N | Q | E | L | P | I | - |
| 017 UniRef90\_A0A1Z4BZ71\_24\_403 | F | E | V | A | G | A | R | A | T | T | A | K | L | G | M | D | R | F | W | R | N | L | R | V | Y | T | L | H | D | P | I | D | Y | K | L | R | D | L | G | D | W | A | L | N | G | N | Y | P | A | H |
| 018 UniRef90\_A0A1Z4IGC5\_10\_387 | F | E | L | M | G | A | R | A | T | A | T | D | Y | G | F | D | R | Y | W | R | N | L | R | T | L | T | L | H | D | P | L | A | Y | K | I | Q | E | V | G | K | W | A | L | N | R | E | F | P | - | - |
| 019 UniRef90\_A0A1W9JD13\_18\_401 | F | E | V | T | G | S | R | A | T | H | A | G | L | R | L | D | R | H | W | R | N | L | R | T | H | T | L | H | D | P | L | D | Y | K | I | K | E | L | G | E | W | A | L | N | N | R | F | P | E | P |
| 020 UniRef90\_A0A0B6S5D2\_8\_393 | F | D | A | C | G | A | R | A | T | A | A | P | L | A | L | D | R | F | W | R | N | A | R | T | H | T | L | H | D | P | L | D | Y | R | L | R | D | L | G | R | H | A | L | T | G | E | L | P | - | - |
| 021 UniRef90\_A0A1Y3C786\_14\_396 | F | E | V | T | G | A | R | A | T | H | A | A | L | C | L | D | R | F | W | R | N | V | R | T | Q | S | L | H | D | P | V | E | Y | K | I | K | D | L | G | Q | W | A | L | N | D | I | Y | P | K | A |
| 022 UniRef90\_A0A318KD41\_20\_397 | F | D | V | T | G | A | R | A | T | S | A | A | L | R | L | D | R | F | W | R | N | L | R | T | H | S | L | H | D | P | V | D | Y | K | L | Q | E | L | G | D | W | A | L | N | D | A | Y | P | K | P |
| 023 UniRef90\_A0A352JDP6\_15\_389 | F | E | V | M | G | A | R | S | T | S | A | K | Y | G | F | D | R | Y | W | R | N | L | R | T | F | T | L | H | D | P | V | E | Y | K | I | K | A | I | G | D | W | A | L | N | H | Q | I | P | - | - |
| 024 UniRef90\_A0A329B538\_13\_401 | F | E | V | T | G | A | R | A | T | H | A | A | L | R | L | D | R | H | W | R | N | L | R | T | Q | T | L | H | D | P | V | D | Y | K | L | R | E | L | G | D | W | A | L | N | Q | Q | P | P | A | P |
| 025 UniRef90\_A0A2N8QAF3\_13\_394 | F | D | A | C | G | A | R | A | T | H | A | P | L | A | L | D | R | F | W | R | N | A | R | V | H | T | L | H | D | P | L | D | Y | R | V | R | D | V | G | R | Y | A | L | S | G | T | L | P | - | - |
| 026 UniRef90\_A0A2N7XWT8\_11\_394 | F | E | V | T | G | A | R | A | T | N | A | S | V | A | L | D | R | H | W | R | N | L | R | T | Q | S | L | H | D | P | L | D | Y | K | L | H | E | L | G | E | W | A | L | N | G | T | A | P | I | P |
| 027 UniRef90\_A0A2A4HLB2\_33\_412 | F | D | I | A | G | A | R | A | T | A | R | P | L | G | L | D | R | F | W | R | N | A | R | T | H | T | L | H | D | P | V | D | Y | K | I | R | D | L | G | R | W | A | L | L | D | Q | Y | P | V | P |
| 028 UniRef90\_A0A4R3HWL3\_41\_416 | F | E | V | M | G | A | R | A | T | A | A | K | Y | G | F | D | R | F | W | R | N | V | R | V | H | T | L | H | D | S | L | D | Y | K | L | K | D | V | G | S | W | V | L | T | G | T | V | P | A | P |
| 029 UniRef90\_A0A1M7NXP6\_14\_394 | F | E | L | T | G | A | R | S | T | S | I | K | F | G | F | D | R | F | W | R | N | A | R | V | H | T | L | H | D | P | V | D | Y | K | I | R | D | L | G | R | Y | A | L | D | G | T | L | P | E | P |
| 030 UniRef90\_A0A1B4ESK2\_22\_397 | F | D | A | C | G | A | R | A | T | A | A | S | L | G | L | D | R | F | W | R | N | A | R | T | H | T | L | H | D | P | L | D | Y | R | L | R | D | V | G | R | F | A | L | T | G | E | L | P | - | - |
| 031 UniRef90\_A0A1H6NKV2\_11\_394 | F | E | V | T | G | A | R | A | T | H | A | S | L | R | L | D | R | H | W | R | N | L | R | T | Q | T | L | H | D | P | L | D | Y | K | L | Q | E | L | G | D | W | A | L | N | Q | T | L | P | I | P |
| 032 UniRef90\_A0A0D0KUF8\_11\_394 | F | E | V | T | G | A | R | A | T | H | A | S | L | R | L | D | R | H | W | R | N | L | R | T | Q | T | L | H | D | P | V | D | Y | K | I | Q | E | L | G | D | W | A | L | N | Q | T | R | P | T | P |
| 033 UniRef90\_A0A0F3K7B4\_20\_401 | F | E | V | T | G | A | R | A | T | H | A | G | L | R | L | D | R | H | W | R | N | L | R | T | H | T | L | H | D | P | V | D | Y | K | L | K | E | L | G | E | W | A | L | I | R | N | Y | P | T | P |
| 034 UniRef90\_A0A381IMG6\_13\_394 | F | D | A | C | G | A | R | A | T | A | A | P | L | A | L | D | R | F | W | R | N | A | R | T | H | T | L | H | D | P | L | D | Y | R | L | R | D | V | G | R | Y | A | L | T | G | V | L | P | - | - |
| 035 UniRef90\_A0A2S9K1G1\_26\_400 | F | E | L | T | G | A | S | A | T | S | A | R | F | G | L | D | R | F | W | R | N | A | R | V | H | T | L | H | D | P | I | D | Y | K | L | R | D | L | G | R | H | A | L | T | G | S | F | P | E | P |
| 036 UniRef90\_A0A1W6L7W5\_17\_392 | F | E | L | T | G | A | R | S | T | S | T | R | L | G | L | D | R | F | W | R | N | A | R | V | H | T | L | H | D | P | V | D | Y | K | L | R | T | L | G | R | H | A | L | L | G | E | L | P | E | P |
| 037 UniRef90\_A0A255HJE3\_14\_398 | F | E | L | T | G | A | R | S | T | S | A | Q | F | G | Y | D | R | Y | W | R | N | V | R | V | H | T | L | H | D | P | V | D | Y | K | L | R | D | L | G | R | Y | A | L | S | G | T | V | P | E | P |
| 038 UniRef90\_A0A0R3AD45\_11\_394 | F | E | V | T | G | A | R | S | T | H | A | S | L | R | L | D | R | H | W | R | N | L | R | T | Q | S | L | H | D | P | V | D | Y | K | L | H | E | L | G | D | W | A | L | N | Q | S | L | P | T | P |
| 039 UniRef90\_A0A1W6ZB60\_16\_389 | F | E | V | T | G | A | R | S | T | S | A | R | Y | G | Y | D | R | Y | W | R | N | A | R | V | H | T | L | H | D | P | V | A | Y | K | V | R | D | L | G | R | Y | A | L | D | G | R | A | P | E | P |
| 040 UniRef90\_A0A2X1DPB7\_14\_397 | F | D | A | C | G | A | R | A | T | A | A | S | L | G | L | D | R | F | W | R | N | A | R | T | H | T | L | H | D | P | L | D | Y | R | L | R | D | V | G | R | F | A | L | T | D | A | L | P | - | - |
| 041 UniRef90\_A0A0S9M2D1\_4\_380 | F | D | V | A | G | A | R | A | T | H | G | G | L | R | L | D | R | H | W | R | N | L | R | T | Q | S | L | H | D | P | A | D | Y | K | L | R | E | L | G | E | W | A | L | K | R | Q | H | P | H | A |
| 042 UniRef90\_A0A238ZKW1\_13\_401 | F | E | V | A | G | S | R | A | T | H | A | G | L | R | L | D | R | H | W | R | N | L | R | T | H | T | L | H | D | P | V | D | Y | K | I | K | E | L | G | E | W | A | L | K | Y | Q | Y | P | R | P |
| 043 UniRef90\_A0A0D1P8V6\_10\_394 | F | E | V | T | G | A | R | A | T | Q | A | S | V | G | L | D | R | H | W | R | N | L | R | T | Q | S | L | H | D | P | L | D | Y | K | L | H | E | L | G | D | W | A | L | N | Q | R | L | P | I | P |
| 044 UniRef90\_A0A261SP68\_11\_387 | F | E | A | T | G | A | R | S | T | S | A | R | Y | G | Y | D | R | F | W | R | N | A | R | V | H | T | L | H | D | P | V | A | Y | K | I | R | D | L | G | R | Y | E | L | D | G | R | A | P | E | P |
| 045 UniRef90\_A0A2N6MRB2\_11\_390 | F | E | V | M | G | T | R | S | T | A | S | K | Y | G | F | D | R | Y | W | R | D | L | R | T | F | T | L | H | D | P | V | D | Y | K | L | R | D | I | G | N | W | V | L | N | Q | E | L | P | L | - |
| 046 UniRef90\_A0A212BVA1\_31\_405 | F | E | T | M | G | A | R | A | T | A | S | S | Y | G | F | D | R | F | W | R | N | V | R | V | H | S | L | H | D | P | L | D | Y | K | V | R | D | I | G | Q | W | L | T | R | S | V | P | P | T | P |
| 047 UniRef90\_A0A1A9KE82\_11\_394 | F | E | V | T | G | A | R | A | T | H | A | A | L | R | L | D | R | H | W | R | N | L | R | T | Q | S | L | H | D | P | L | D | Y | R | V | Q | E | L | G | A | W | A | L | N | R | Q | R | P | S | P |
| 048 UniRef90\_A0A4Q5PVB9\_26\_400 | F | E | L | M | G | A | R | A | T | T | S | A | N | G | F | D | R | Y | W | R | N | V | R | V | H | T | L | H | D | P | V | D | Y | R | S | K | A | L | G | Q | W | M | L | T | G | E | L | P | D | P |
| 049 UniRef90\_A0A178GPM9\_9\_392 | F | Q | V | M | G | A | R | A | T | T | A | K | L | N | L | D | R | F | W | R | N | V | R | T | Q | T | L | H | D | P | I | D | Y | K | Y | Q | E | V | G | E | W | V | L | T | G | K | V | P | D | P |
| 050 UniRef90\_A0A1P9YC32\_3\_390 | F | E | V | T | G | A | R | S | T | T | A | A | L | R | L | D | R | F | W | R | N | V | R | V | H | T | L | H | D | P | L | D | Y | K | V | R | E | L | G | D | W | A | L | N | E | R | I | P | K | P |
| 051 UniRef90\_UPI000A1773A9\_40\_416 | F | E | V | T | G | A | R | S | T | H | A | G | L | G | L | D | R | H | W | R | N | L | R | T | H | T | L | H | D | P | V | Q | Y | K | I | R | E | L | G | E | W | A | L | K | S | H | L | P | A | P |
| 052 UniRef90\_A0A158L201\_25\_395 | F | D | A | C | G | A | R | A | T | H | G | P | L | A | L | D | R | F | W | R | N | A | R | V | H | T | L | H | D | P | L | D | Y | K | L | R | D | I | G | R | F | A | L | S | G | V | L | P | E | - |
| 053 UniRef90\_A0A4Q4GT15\_13\_391 | F | Q | V | M | G | A | R | A | T | T | A | K | L | N | L | D | R | F | W | R | N | V | R | T | Q | T | L | H | D | P | I | D | Y | K | Y | Q | E | V | G | E | W | V | L | T | G | K | V | P | E | P |
| 054 UniRef90\_A0A1C0YC62\_17\_381 | F | Q | V | M | G | A | R | A | T | N | A | Q | Y | N | F | D | R | F | W | R | N | V | R | T | H | T | L | H | D | P | I | D | Y | K | L | R | D | L | G | Q | Y | A | L | N | D | R | Y | P | T | - |
| 055 UniRef90\_A0A1H7FE04\_32\_405 | F | E | L | T | G | A | R | S | T | S | A | R | F | G | F | D | R | F | W | R | N | A | R | V | H | T | L | H | D | P | V | D | Y | K | V | R | D | I | G | R | Y | R | V | D | G | R | I | P | D | P |
| 056 UniRef90\_A0A401MWC4\_5\_385 | L | E | V | T | G | A | R | S | T | A | S | A | H | G | F | D | R | F | W | R | N | A | R | T | H | T | L | H | D | P | V | A | Y | K | L | R | E | V | G | D | H | F | L | N | G | T | H | P | - | - |
| 057 UniRef90\_A0A1G6HFW7\_16\_395 | F | E | V | T | G | A | R | A | T | T | A | A | L | R | F | D | R | F | W | R | N | L | R | V | H | T | L | H | D | P | I | D | Y | K | I | R | E | L | G | D | W | A | L | N | E | R | F | P | D | P |
| 058 UniRef90\_A0A0P9B999\_30\_403 | F | E | L | V | G | A | R | G | T | A | S | K | H | N | L | D | R | Y | W | R | N | V | R | V | H | T | L | H | D | P | L | D | Y | R | H | Q | G | I | G | A | W | L | L | A | G | D | V | P | N | P |
| 059 UniRef90\_A0A140K6F0\_14\_392 | F | E | V | M | G | S | R | A | T | S | N | R | Y | G | F | D | R | Y | W | R | D | Q | R | T | F | T | L | H | D | P | I | D | Y | K | L | L | D | V | G | N | W | L | L | N | D | I | F | P | T | P |
| 060 UniRef90\_A0A1P8EKI7\_5\_392 | F | Q | V | M | G | A | R | A | T | T | A | A | L | N | L | D | R | F | W | R | N | V | R | T | Q | T | L | H | D | P | I | D | Y | K | Y | Q | E | V | G | E | W | V | L | T | G | K | V | P | H | P |
| 061 UniRef90\_A0A2U3MYZ3\_7\_392 | F | Q | V | M | G | A | R | A | T | T | A | K | L | N | L | D | R | F | W | R | N | V | R | T | Q | T | L | H | D | P | I | D | Y | K | Y | Q | E | V | G | E | W | I | L | T | E | Q | V | P | K | P |
| 062 UniRef90\_A0A1H2EPX5\_7\_397 | F | D | L | V | G | A | K | A | A | V | H | A | A | R | L | D | R | F | W | R | N | A | R | L | H | T | L | H | D | P | I | D | Y | K | V | R | E | L | G | V | H | A | L | L | G | T | H | P | K | P |
| 063 UniRef90\_UPI00041EF43F\_25\_400 | F | E | L | T | G | A | G | A | T | A | S | R | L | G | L | D | R | F | W | R | N | A | R | V | H | T | L | H | D | P | L | D | Y | K | L | R | D | L | G | R | H | A | L | L | G | R | Y | P | E | P |
| 064 UniRef90\_D0IW93\_17\_384 | F | D | V | V | G | S | R | G | T | H | A | D | L | G | F | D | R | F | W | R | N | V | R | T | H | T | L | H | D | P | L | D | Y | K | L | Q | A | L | G | R | W | A | V | H | G | E | E | P | - | - |
| 065 UniRef90\_A0A395D1I6\_11\_388 | F | D | L | M | G | A | R | S | T | A | A | A | H | R | F | D | R | F | W | R | N | A | R | T | H | T | L | H | D | P | L | D | H | K | L | R | E | L | G | S | F | A | F | E | G | Q | F | P | T | P |
| 066 UniRef90\_A0A1H0TK45\_27\_400 | F | E | V | T | G | A | R | A | T | T | A | A | L | R | L | D | R | Y | W | R | N | L | R | V | H | T | L | H | D | P | V | D | Y | K | L | R | E | L | G | D | W | A | L | N | E | T | L | P | H | P |
| 067 UniRef90\_A0A1H8MYU5\_23\_403 | F | E | V | M | G | A | R | S | A | V | R | P | L | G | F | D | R | F | W | R | N | V | R | I | H | T | L | H | N | P | A | E | Y | K | T | R | N | V | G | A | W | F | L | T | G | D | Y | P | E | P |
| 068 UniRef90\_A0A1B1M254\_5\_403 | F | E | V | T | G | A | R | S | T | A | T | A | E | G | L | D | R | F | W | R | N | V | R | T | H | T | L | H | D | P | V | A | Y | K | R | R | E | V | G | R | W | V | L | E | G | E | L | P | E | P |
| 069 UniRef90\_A0A484THM3\_28\_401 | F | D | L | V | G | A | R | A | T | T | R | Q | L | G | L | D | R | Y | W | R | N | V | R | V | H | T | L | H | D | P | L | D | H | R | Q | Q | A | I | G | R | W | L | L | T | G | L | - | - | - | - |
| 070 UniRef90\_A0A0Q8Q8X6\_8\_377 | L | E | V | A | G | A | R | G | A | K | A | E | L | G | F | D | R | F | W | R | N | V | R | T | H | T | L | H | D | P | L | D | Y | K | L | S | V | L | G | K | W | A | L | T | G | E | S | P | - | - |
| 071 UniRef90\_A0A0Q5QDL2\_32\_410 | F | E | V | T | G | A | R | S | T | S | S | K | V | G | L | D | L | F | F | R | N | V | R | T | H | T | L | H | D | P | V | A | Y | K | R | R | E | V | G | R | H | Q | L | I | G | E | L | P | E | P |
| 072 UniRef90\_A0A1A5XM62\_22\_406 | F | D | A | C | G | A | R | A | T | A | A | A | L | A | F | D | R | F | W | R | N | A | R | V | H | T | L | H | D | P | L | D | Y | R | V | R | D | V | G | R | H | A | L | T | R | E | L | P | E | P |
| 073 UniRef90\_A0A239EI90\_21\_397 | F | E | A | L | G | A | R | A | T | A | S | K | F | G | F | D | R | F | W | R | N | V | R | T | H | T | L | H | D | P | V | A | Y | K | R | R | E | V | G | R | W | V | L | T | G | E | L | P | E | P |
| 074 UniRef90\_S5SWW2\_23\_403 | F | E | V | M | G | A | R | S | A | V | R | P | H | G | F | D | R | F | W | R | N | V | R | I | H | T | L | H | N | P | A | E | Y | K | A | R | N | V | G | T | W | F | L | T | G | D | F | P | E | P |
| 075 UniRef90\_A0A3A5JJY9\_31\_413 | F | E | L | G | G | T | A | T | V | A | R | P | R | H | L | D | R | F | W | R | N | A | R | T | H | T | L | H | D | P | V | D | Y | K | L | R | D | L | G | R | W | A | I | S | G | T | Y | P | E | P |
| 076 UniRef90\_A0A315ZU85\_7\_384 | F | E | V | T | G | A | R | A | T | S | N | R | V | G | L | D | R | F | W | R | N | V | R | T | H | T | L | H | D | P | V | Q | Y | K | A | R | E | V | G | D | H | F | L | N | G | T | H | P | - | - |
| 077 UniRef90\_A0A3R9U899\_5\_381 | F | E | V | T | G | A | R | A | T | A | S | A | L | G | L | D | R | F | W | R | N | V | R | T | H | T | L | H | D | Q | - | - | - | - | - | - | - | - | - | - | - | - | - | - | - | - | - | - | - | - |
| 078 UniRef90\_UPI000DD53D91\_7\_389 | F | E | V | M | G | A | R | S | A | T | T | E | N | G | F | N | R | F | W | R | N | V | R | T | H | T | L | H | N | P | A | E | Y | K | K | R | T | V | G | T | W | L | L | T | G | E | F | P | V | P |
| 079 UniRef90\_UPI000DE4BE09\_13\_396 | F | E | L | T | G | A | R | S | T | A | E | H | Y | R | F | D | R | F | W | R | N | V | R | T | H | T | L | H | D | P | V | F | Y | K | A | R | E | V | G | E | F | V | L | N | D | T | I | P | - | - |
| 080 UniRef90\_A0A2T0R7P7\_10\_381 | Y | E | V | T | G | A | R | A | T | A | N | K | H | G | F | D | R | F | W | R | N | V | R | T | H | T | L | H | D | P | V | Q | Y | K | A | R | E | V | G | N | H | F | L | T | G | A | H | P | - | - |
| 081 UniRef90\_A0A4R1HYS3\_27\_404 | F | E | G | L | G | A | R | A | T | T | S | Q | L | G | F | D | R | F | W | R | N | V | R | T | H | T | L | H | D | P | V | A | Y | K | R | R | E | V | G | A | F | L | L | R | D | E | L | P | E | P |
| 082 UniRef90\_UPI000D1537B0\_7\_385 | F | E | V | M | G | A | R | S | A | T | R | A | N | G | F | D | R | F | W | R | N | V | R | V | H | T | L | H | N | P | A | E | Y | K | T | R | N | V | G | T | W | V | L | T | G | Q | Y | P | P | P |
| 083 UniRef90\_A0A1G6ZNM7\_17\_409 | F | E | V | T | G | A | R | A | S | A | N | A | V | G | L | D | L | F | W | R | N | I | R | T | H | S | L | H | D | P | V | A | H | K | R | A | E | V | G | R | Y | A | L | T | G | E | L | P | E | P |
| 084 UniRef90\_UPI0003765980\_18\_389 | F | E | V | M | G | A | R | S | A | T | Q | A | N | G | F | D | R | F | W | R | N | V | R | T | H | T | L | H | N | P | A | E | Y | K | K | R | T | V | G | A | F | V | L | T | G | E | F | P | V | P |
| 085 UniRef90\_A0A071ICK8\_25\_398 | F | E | L | T | G | A | R | S | T | A | S | S | Y | R | F | D | R | F | W | R | N | V | R | T | H | T | L | H | D | P | V | F | Y | K | A | K | E | V | G | E | F | V | L | N | G | K | I | P | T | - |
| 086 UniRef90\_A0A2L0WNE5\_33\_396 | F | E | L | T | G | A | R | S | T | A | D | K | Y | R | F | D | R | F | W | R | N | V | R | T | H | T | L | H | D | P | V | F | Y | K | A | K | E | V | G | E | F | V | L | N | D | K | I | P | - | - |
| 087 UniRef90\_A0A1Q8CFJ2\_9\_380 | Y | E | F | T | G | A | R | A | S | A | N | K | Y | G | L | D | R | F | W | R | N | A | R | T | L | T | L | H | D | P | V | A | Y | K | A | R | E | V | G | A | H | F | L | T | G | E | P | P | - | - |
| 088 UniRef90\_UPI000835F605\_21\_394 | Y | E | A | T | G | A | R | S | T | A | N | K | Y | G | L | D | I | Y | W | R | N | V | R | T | H | T | V | H | D | P | V | S | Y | R | L | R | E | I | G | E | F | A | L | N | Q | T | L | P | K | P |
| 089 UniRef90\_UPI000374AB86\_31\_398 | F | E | L | T | G | A | R | S | T | A | E | S | Y | R | F | D | R | F | W | R | N | V | R | T | H | T | L | H | D | P | V | F | Y | K | A | R | E | V | G | E | F | V | L | S | D | K | I | P | A | - |
| 090 UniRef90\_A0A267RVL6\_22\_399 | F | E | L | T | G | G | R | S | T | S | N | Q | Y | G | L | D | V | F | W | R | N | V | R | T | H | T | L | H | D | P | V | T | Y | R | T | R | E | V | G | D | Y | V | L | S | G | K | L | P | T | P |
| 091 UniRef90\_M3VA40\_23\_395 | F | E | I | Q | G | A | R | A | T | T | T | D | Y | G | F | D | R | H | W | R | N | L | R | T | H | T | V | H | D | P | V | A | Y | K | A | R | E | V | G | D | W | T | L | N | H | R | A | P | - | - |
| 092 UniRef90\_A0A395GID9\_32\_425 | F | E | V | T | G | A | R | A | T | S | L | K | V | G | L | D | R | F | W | R | D | I | R | T | H | T | L | H | D | P | V | A | Y | K | N | R | E | L | G | R | Y | A | L | L | G | E | V | P | E | P |
| 093 UniRef90\_UPI0008268C2F\_35\_406 | F | E | I | Q | G | A | R | A | T | T | S | A | Y | G | F | D | R | H | W | R | N | L | R | T | H | T | V | H | D | P | V | A | Y | K | A | R | E | V | G | D | W | T | L | N | R | R | A | P | - | - |
| 094 UniRef90\_A0A0S1XV73\_27\_397 | F | E | L | M | G | A | R | S | A | T | R | A | R | G | Y | D | R | F | W | R | N | V | R | T | H | T | L | H | N | P | A | E | Y | K | K | R | T | I | G | A | W | L | L | R | H | P | Y | - | - | - |
| 095 UniRef90\_A0A0F5N116\_19\_398 | F | E | I | Q | G | A | R | A | T | T | S | K | Y | G | F | D | R | H | W | R | N | L | R | T | H | T | V | H | D | P | V | A | Y | K | A | K | E | V | G | D | W | V | L | N | G | Q | R | P | - | - |
| 096 UniRef90\_A0A1N6Z7J9\_40\_406 | F | E | L | M | G | A | R | A | T | A | S | H | Y | R | F | D | R | Y | W | R | N | V | R | T | H | T | L | H | D | P | V | F | Y | K | A | R | E | V | G | D | F | A | L | N | G | R | V | P | E | P |
| 097 UniRef90\_A0A2V4UWQ2\_31\_411 | F | E | L | M | G | A | R | A | T | A | G | H | Y | R | F | D | R | Y | W | R | N | V | R | T | H | T | L | H | D | P | V | F | Y | K | A | R | E | V | G | D | F | A | L | N | D | R | V | P | T | P |
| 098 UniRef90\_A0A4P8KLT1\_5\_401 | F | E | A | T | G | T | S | S | T | A | N | R | V | G | L | D | L | H | W | R | N | I | R | T | H | S | L | H | D | P | V | D | Y | K | K | L | E | V | G | A | H | F | L | T | G | A | V | - | - | - |
| 099 UniRef90\_UPI0003068598\_3\_292 | F | D | A | C | G | A | R | A | T | A | A | P | L | A | L | D | R | F | W | R | N | A | R | T | H | T | L | H | D | P | L | D | Y | R | L | R | D | V | G | R | Y | A | L | T | G | A | L | P | - | - |
| 100 UniRef90\_UPI000DD585C3\_27\_403 | F | E | L | M | G | A | R | A | T | A | S | H | Y | G | F | D | R | Y | W | R | N | V | R | T | H | T | L | H | D | P | V | S | Y | K | A | R | E | V | G | D | F | A | L | N | G | R | I | P | S | - |
| 101 UniRef90\_A0A1E3SMN8\_25\_404 | F | E | I | Q | G | A | R | A | T | T | S | A | Y | G | F | D | R | H | W | R | N | L | R | T | H | T | L | H | D | P | V | A | Y | K | A | R | E | V | G | D | W | V | L | N | H | R | H | P | - | - |
| 102 UniRef90\_UPI00045EBD18\_12\_396 | F | E | L | M | G | A | R | A | T | A | G | H | Y | G | Y | D | R | F | W | R | N | I | R | T | H | T | L | H | D | P | V | V | Y | K | S | R | E | V | G | N | Y | A | L | N | G | V | I | - | - | - |
| 103 UniRef90\_A0A1Y1ZNX6\_42\_434 | F | E | V | T | G | A | K | A | T | S | A | K | A | G | L | D | R | F | W | R | D | I | R | T | H | S | L | H | D | P | V | A | Y | K | N | R | E | L | G | R | F | Q | L | L | D | E | V | P | E | P |
| 104 UniRef90\_A0A0D2FPI6\_31\_423 | F | E | V | T | G | A | S | S | T | A | R | K | V | G | L | D | R | F | W | R | D | V | R | T | H | T | L | H | D | P | V | A | Y | K | E | R | E | L | G | T | F | Y | L | L | D | E | V | P | E | P |
| 105 UniRef90\_A0A2S0KGS2\_30\_403 | F | E | I | Q | G | A | R | A | T | T | S | A | Y | G | F | D | R | H | W | R | N | L | R | T | H | T | V | H | D | P | V | A | Y | K | A | R | E | I | G | D | W | T | L | N | H | R | A | P | - | - |
| 106 UniRef90\_A0A1A7MGQ0\_54\_442 | F | E | V | T | G | S | R | A | T | G | R | K | V | G | L | D | R | F | W | R | D | V | R | T | H | T | L | H | D | P | V | A | Y | K | N | R | E | L | G | R | Y | F | L | L | D | E | V | P | E | P |
| 107 UniRef90\_UPI000413001D\_18\_392 | F | E | L | M | G | A | R | A | T | A | T | S | Y | G | F | D | R | F | W | R | N | I | R | T | H | T | L | H | D | P | V | F | Y | K | A | R | E | V | G | N | Y | A | L | N | G | T | - | - | - | - |
| 108 UniRef90\_G7H5P9\_11\_400 | F | E | I | Q | G | A | R | A | T | T | S | A | Y | G | F | D | R | H | W | R | N | L | R | T | H | T | V | H | D | P | V | A | Y | K | A | R | E | V | G | D | W | T | L | N | R | R | A | P | - | - |
| 109 UniRef90\_A0A010YHQ5\_11\_404 | F | E | L | T | G | A | R | A | T | K | T | G | T | G | L | D | R | Y | W | R | D | V | R | T | L | T | L | H | D | P | V | S | H | K | A | I | E | V | G | D | H | L | L | R | G | A | Y | P | E | P |
| 110 UniRef90\_B0RBU7\_24\_404 | F | E | V | T | G | S | S | S | A | R | S | S | T | G | L | D | L | F | W | R | N | V | R | T | H | S | L | H | D | P | V | D | Y | K | K | L | E | V | G | A | H | A | L | T | G | E | L | - | - | - |
| 111 UniRef90\_A0A167RW81\_42\_429 | F | E | V | T | G | S | R | A | T | A | T | A | V | G | L | D | R | F | W | R | D | I | R | T | H | T | L | H | D | P | V | A | Y | K | N | H | E | L | G | R | F | Q | L | L | G | A | Y | P | E | P |
| 112 UniRef90\_A0A1X0J8A0\_26\_405 | Y | Q | A | T | G | A | R | S | T | A | N | S | V | G | L | D | I | Y | W | R | N | A | R | T | H | T | T | H | D | P | L | P | Y | R | Q | R | E | I | G | R | Y | L | L | T | D | E | W | P | L | P |
| 113 UniRef90\_A0A2J6Q859\_43\_430 | F | E | A | T | G | A | T | S | T | K | S | K | F | G | L | D | R | F | W | R | D | I | R | T | H | T | L | H | D | P | V | A | Y | K | N | R | E | L | G | R | F | Q | L | L | N | E | V | P | E | P |
| 114 UniRef90\_UPI0003828F02\_7\_389 | F | E | F | T | G | A | R | A | T | A | A | K | H | G | M | D | R | F | W | R | N | A | R | T | L | T | L | H | D | P | A | V | Y | K | A | A | E | V | G | R | H | L | L | T | G | E | Y | P | T | P |
| 115 UniRef90\_A0A1B2HDJ6\_6\_361 | F | E | Q | V | G | A | R | G | T | A | Q | K | Y | G | V | D | R | F | W | R | N | A | R | T | L | T | L | H | D | P | V | V | Y | K | A | R | E | V | G | E | H | F | L | T | G | A | R | P | A | - |
| 116 UniRef90\_A0A2N3N0U1\_47\_427 | F | E | V | T | G | A | R | T | T | S | A | K | Y | G | L | D | R | F | W | R | D | L | R | T | H | T | L | H | D | P | V | A | Y | K | N | K | E | L | G | R | F | Q | L | L | N | E | V | P | E | P |
| 117 UniRef90\_S5Y4X6\_32\_408 | Y | E | L | T | G | G | R | S | T | A | N | R | F | G | L | D | H | F | W | R | N | I | R | T | H | T | T | H | D | P | V | S | Y | R | A | R | E | I | G | L | A | R | I | S | G | T | L | P | V | P |
| 118 UniRef90\_A0A3R2WQN2\_17\_411 | F | E | V | T | G | A | R | A | T | A | N | A | V | G | L | D | I | F | W | R | N | I | R | T | H | S | L | H | D | P | I | A | Y | K | R | R | E | V | G | A | Y | A | L | R | G | Q | I | P | E | P |
| 119 UniRef90\_A0A3N1X255\_20\_392 | F | E | V | M | G | A | R | S | A | T | R | A | R | G | Y | D | R | F | W | R | N | V | R | T | H | T | L | H | N | P | A | E | Y | K | K | R | T | L | G | Q | W | L | L | D | G | S | H | P | Q | P |
| 120 UniRef90\_A0A1S1LA29\_28\_404 | Y | Q | A | T | G | A | R | S | T | A | N | S | V | G | L | D | I | Y | W | R | N | A | R | T | H | T | T | H | D | P | L | P | Y | R | Q | R | E | I | G | R | H | L | L | T | D | E | W | P | S | P |
| 121 UniRef90\_A0A081GNL4\_19\_392 | F | D | R | V | G | A | R | Y | T | H | A | A | L | G | L | D | R | Y | W | R | N | A | R | T | L | S | L | H | D | P | L | D | V | K | L | Q | E | I | G | D | H | V | L | N | G | Q | H | P | R | P |
| 122 UniRef90\_A0A1I6U758\_27\_419 | Y | E | L | T | G | A | R | A | T | S | N | A | V | G | L | D | L | Y | W | R | N | L | R | T | H | S | L | H | D | P | V | A | Y | K | R | V | E | V | G | R | Y | A | L | L | G | D | L | P | T | P |
| 123 UniRef90\_A0A0F5VZP9\_7\_388 | F | E | V | S | G | T | R | S | A | L | N | S | L | N | L | H | R | H | W | R | D | A | R | T | H | T | L | H | D | P | T | R | W | K | I | Q | H | I | G | R | Y | V | L | N | G | T | R | P | - | - |
| 124 UniRef90\_A0A1H4GIM8\_20\_411 | Y | E | L | A | G | A | R | A | S | A | S | S | V | G | L | D | I | F | W | R | N | L | R | T | H | S | L | H | D | P | I | A | Y | K | K | R | E | V | G | E | Y | V | L | L | N | Q | I | P | E | P |
| 125 UniRef90\_A0A0M8TTT1\_8\_388 | F | E | V | S | G | T | R | S | A | L | D | S | L | N | L | H | R | H | W | R | D | A | R | T | H | T | L | H | D | P | A | R | W | K | V | Q | H | I | G | R | Y | V | L | N | G | T | R | P | - | - |
| 126 UniRef90\_A0A1Y2MDA3\_48\_437 | F | E | V | T | G | S | K | A | T | S | L | R | V | G | L | D | R | F | W | R | D | I | R | T | H | T | L | H | D | P | V | A | Y | K | N | R | E | L | G | R | F | E | L | L | D | E | I | P | - | - |
| 127 UniRef90\_UPI000562F0C5\_9\_388 | F | E | V | S | G | T | R | S | A | L | N | S | L | N | L | H | R | H | W | R | D | A | R | T | H | T | L | H | D | P | V | R | W | K | I | Q | H | I | G | R | Y | V | L | S | G | T | R | P | - | - |
| 128 UniRef90\_A0A506Y8Z1\_43\_442 | F | E | L | T | G | A | R | A | S | A | N | T | V | G | L | D | I | H | W | R | N | L | R | T | H | S | L | H | D | P | V | P | Y | K | R | R | E | V | G | R | F | A | L | L | G | E | I | P | E | P |
| 129 UniRef90\_A0A021VVW0\_28\_409 | F | E | V | T | G | A | R | A | T | A | S | D | V | G | L | D | R | F | W | R | N | V | R | T | H | S | L | H | D | P | V | A | Y | K | R | R | E | V | G | R | F | A | L | L | D | E | V | P | E | P |
| 130 UniRef90\_E3QTS2\_46\_428 | F | E | V | T | G | A | R | A | T | A | T | K | V | G | L | D | R | F | W | R | D | I | R | T | H | S | L | H | D | P | V | A | Y | K | N | R | E | L | G | R | Y | Q | L | L | G | E | I | P | E | P |
| 131 UniRef90\_A0A1D8SMV7\_7\_388 | F | E | V | S | G | T | R | A | A | L | D | S | L | N | L | H | R | H | W | R | D | A | R | T | H | T | L | H | D | P | T | R | W | K | V | Q | H | I | G | R | H | V | L | T | G | T | R | P | - | - |
| 132 UniRef90\_A9BUV8\_38\_419 | F | E | L | A | G | T | A | S | T | L | A | G | Q | G | L | D | R | F | W | R | N | A | R | T | H | T | L | H | D | P | V | R | W | K | Y | H | A | V | G | N | Y | V | L | N | G | I | R | P | - | - |
| 133 UniRef90\_A0A1N6TB45\_32\_407 | L | E | A | L | G | P | L | A | A | D | H | G | A | G | L | D | R | F | W | R | N | V | R | I | H | T | L | H | N | P | A | E | Y | K | V | R | N | V | G | R | H | F | L | S | G | Q | P | P | A | P |
| 134 UniRef90\_A0A381IE41\_24\_406 | F | E | L | A | G | T | Q | A | T | L | A | E | H | G | L | D | R | H | W | R | N | A | R | T | H | T | L | H | D | P | V | R | W | K | Y | H | L | V | G | N | Y | Y | L | N | G | V | A | P | - | - |
| 135 UniRef90\_A0A3M8TJ33\_18\_397 | L | D | V | L | G | A | R | S | A | A | S | A | Y | G | F | D | R | F | W | R | N | V | R | T | H | T | L | C | V | P | V | A | P | R | L | Q | E | V | G | D | Y | F | L | H | G | E | H | P | - | - |
| 136 UniRef90\_A0A1H3JL41\_4\_373 | F | E | F | L | G | A | R | A | T | A | G | S | F | G | F | D | R | F | W | R | N | A | R | T | L | T | L | H | D | P | V | V | Y | K | A | R | E | L | G | A | H | Y | L | T | G | E | P | P | E | P |
| 137 UniRef90\_A0A507AW89\_49\_427 | F | E | V | T | G | A | K | A | T | A | T | K | V | G | L | D | R | F | W | R | D | V | R | T | H | T | L | H | D | P | V | S | Y | K | N | R | E | L | G | R | Y | Q | L | L | R | E | L | P | E | P |
| 138 UniRef90\_A0A1X1SEN2\_20\_406 | F | E | V | T | G | A | R | S | T | A | A | P | E | G | L | D | R | F | W | R | N | A | R | T | L | T | L | H | D | P | L | H | H | K | Q | T | Q | I | G | D | Y | V | L | N | G | V | A | P | A | P |
| 139 UniRef90\_A0A2D3UFX4\_26\_403 | L | D | I | L | G | A | R | S | T | S | S | R | L | G | F | D | R | F | W | R | N | A | R | T | H | T | L | Y | E | P | V | A | H | R | L | R | D | V | G | D | Y | F | L | N | G | A | H | P | - | - |
| 140 UniRef90\_A0A089X0S9\_26\_403 | L | D | V | V | G | A | R | S | T | P | A | R | L | G | L | D | R | F | W | R | D | A | R | T | H | T | L | Y | E | P | V | A | P | R | L | R | D | V | G | D | Y | F | L | N | G | A | H | P | - | - |
| 141 UniRef90\_A0A2W5T1D0\_29\_413 | F | E | L | T | G | A | R | A | S | A | N | S | V | G | L | D | I | F | W | R | N | L | R | T | H | S | L | H | D | P | I | P | Y | K | R | R | E | V | G | V | F | A | L | L | G | E | V | P | E | P |
| 142 UniRef90\_A0A1C4QGR9\_7\_388 | F | D | L | A | G | T | R | S | A | L | D | S | L | N | L | H | R | H | W | R | D | A | R | T | H | T | L | H | D | P | A | R | W | K | I | Q | H | I | G | R | H | L | L | N | G | T | R | P | - | - |
| 143 UniRef90\_A0A101R4L6\_7\_388 | F | E | V | S | G | T | R | S | A | L | D | S | L | N | L | H | R | Y | W | R | D | A | R | T | H | T | L | H | D | P | P | R | W | K | L | Q | H | L | G | R | H | V | L | T | G | A | R | P | - | - |
| 144 UniRef90\_A0A4D4LHU8\_7\_388 | F | E | V | S | G | T | R | S | A | L | N | S | L | N | L | H | R | H | W | R | D | A | R | T | H | T | L | H | D | P | A | R | W | K | I | Q | H | I | G | R | H | V | L | N | G | T | K | P | - | - |
| 145 UniRef90\_L1KK35\_6\_389 | F | E | V | A | G | T | R | S | A | L | D | S | L | G | L | H | R | H | W | R | D | A | R | T | H | T | L | H | D | P | A | R | W | K | V | Q | H | I | G | R | Y | V | L | S | G | I | K | P | - | - |
| 146 UniRef90\_UPI0005601C6B\_13\_390 | F | E | V | A | G | T | R | S | A | L | D | S | L | N | L | H | R | Y | W | R | D | A | R | T | H | T | L | H | D | P | A | R | W | K | V | Q | H | I | G | R | Y | V | L | N | G | T | R | P | - | - |
| 147 UniRef90\_A0A1H9UQJ9\_6\_361 | F | E | Q | V | G | A | R | G | T | A | A | K | H | G | V | D | R | F | W | R | N | A | R | T | L | T | L | H | D | P | V | V | Y | K | A | R | E | V | G | E | H | F | L | T | G | A | R | P | A | - |
| 148 UniRef90\_A0A3D9JKY9\_12\_401 | L | D | V | I | G | A | D | A | A | S | V | R | H | G | F | D | R | F | W | R | N | A | R | T | H | T | L | R | E | P | V | A | H | R | L | R | E | I | G | D | Y | F | L | N | G | A | H | P | - | - |
| 149 UniRef90\_D9X7Q2\_26\_403 | L | D | I | I | G | A | R | S | A | S | A | R | L | G | F | D | R | F | W | R | N | A | R | T | H | T | L | Y | E | P | V | A | H | R | L | R | D | V | G | D | Y | F | L | N | G | A | H | P | - | - |
| 150 UniRef90\_A0A1X1DXJ3\_20\_398 | L | E | W | G | G | S | R | A | T | L | L | Q | H | G | L | D | R | H | W | R | N | A | R | T | H | T | L | H | D | P | V | R | W | K | T | H | A | I | G | N | Y | Y | L | N | A | V | Y | P | A | - |

  
  

|  |  |  |  |  |
| --- | --- | --- | --- | --- |
| **001 Input\_pdb\_SEQRES\_A** | G | F | T | S |
| 002 UniRef90\_Q1W1G3\_1\_416 | G | F | T | S |
| 003 UniRef90\_T0BM21\_7\_392 | G | W | - | - |
| 004 UniRef90\_UPI0002AC58FE\_11\_391 | N | F | Y | - |
| 005 UniRef90\_A0A3D5CW51\_20\_401 | T | F | Y | - |
| 006 UniRef90\_A0A2V4SPP5\_16\_400 | - | - | - | - |
| 007 UniRef90\_E0UIV5\_8\_393 | N | F | Y | - |
| 008 UniRef90\_A0A1Z4S897\_9\_391 | - | - | - | - |
| 009 UniRef90\_UPI00045E9273\_21\_396 | S | F | Y | - |
| 010 UniRef90\_A0A353Y4M9\_17\_384 | G | - | - | - |
| 011 UniRef90\_F3KR10\_17\_384 | S | - | - | - |
| 012 UniRef90\_A0A2D8NW56\_20\_404 | - | - | - | - |
| 013 UniRef90\_A0A2W7M9P1\_16\_386 | - | - | - | - |
| 014 UniRef90\_A0A398AYR9\_12\_386 | - | - | - | - |
| 015 UniRef90\_A0A0T6UXN9\_11\_394 | S | F | Y | - |
| 016 UniRef90\_A0A252E884\_11\_390 | - | - | - | - |
| 017 UniRef90\_A0A1Z4BZ71\_24\_403 | S | F | Y | - |
| 018 UniRef90\_A0A1Z4IGC5\_10\_387 | - | - | - | - |
| 019 UniRef90\_A0A1W9JD13\_18\_401 | T | F | Y | - |
| 020 UniRef90\_A0A0B6S5D2\_8\_393 | - | - | - | - |
| 021 UniRef90\_A0A1Y3C786\_14\_396 | S | F | - | - |
| 022 UniRef90\_A0A318KD41\_20\_397 | S | F | Y | - |
| 023 UniRef90\_A0A352JDP6\_15\_389 | - | - | - | - |
| 024 UniRef90\_A0A329B538\_13\_401 | S | F | Y | - |
| 025 UniRef90\_A0A2N8QAF3\_13\_394 | - | - | - | - |
| 026 UniRef90\_A0A2N7XWT8\_11\_394 | T | F | Y | - |
| 027 UniRef90\_A0A2A4HLB2\_33\_412 | T | - | - | - |
| 028 UniRef90\_A0A4R3HWL3\_41\_416 | S | I | - | - |
| 029 UniRef90\_A0A1M7NXP6\_14\_394 | - | - | - | - |
| 030 UniRef90\_A0A1B4ESK2\_22\_397 | - | - | - | - |
| 031 UniRef90\_A0A1H6NKV2\_11\_394 | T | F | Y | - |
| 032 UniRef90\_A0A0D0KUF8\_11\_394 | T | F | Y | - |
| 033 UniRef90\_A0A0F3K7B4\_20\_401 | T | F | Y | - |
| 034 UniRef90\_A0A381IMG6\_13\_394 | - | - | - | - |
| 035 UniRef90\_A0A2S9K1G1\_26\_400 | S | - | - | - |
| 036 UniRef90\_A0A1W6L7W5\_17\_392 | - | - | - | - |
| 037 UniRef90\_A0A255HJE3\_14\_398 | - | - | - | - |
| 038 UniRef90\_A0A0R3AD45\_11\_394 | T | F | Y | - |
| 039 UniRef90\_A0A1W6ZB60\_16\_389 | - | - | - | - |
| 040 UniRef90\_A0A2X1DPB7\_14\_397 | - | - | - | - |
| 041 UniRef90\_A0A0S9M2D1\_4\_380 | S | F | - | - |
| 042 UniRef90\_A0A238ZKW1\_13\_401 | S | F | Y | - |
| 043 UniRef90\_A0A0D1P8V6\_10\_394 | T | F | Y | - |
| 044 UniRef90\_A0A261SP68\_11\_387 | - | - | - | - |
| 045 UniRef90\_A0A2N6MRB2\_11\_390 | - | - | - | - |
| 046 UniRef90\_A0A212BVA1\_31\_405 | S | - | - | - |
| 047 UniRef90\_A0A1A9KE82\_11\_394 | T | F | Y | - |
| 048 UniRef90\_A0A4Q5PVB9\_26\_400 | - | - | - | - |
| 049 UniRef90\_A0A178GPM9\_9\_392 | S | F | Y | - |
| 050 UniRef90\_A0A1P9YC32\_3\_390 | S | F | Y | - |
| 051 UniRef90\_UPI000A1773A9\_40\_416 | S | F | Y | - |
| 052 UniRef90\_A0A158L201\_25\_395 | - | - | - | - |
| 053 UniRef90\_A0A4Q4GT15\_13\_391 | S | F | Y | - |
| 054 UniRef90\_A0A1C0YC62\_17\_381 | - | - | - | - |
| 055 UniRef90\_A0A1H7FE04\_32\_405 | - | - | - | - |
| 056 UniRef90\_A0A401MWC4\_5\_385 | - | - | - | - |
| 057 UniRef90\_A0A1G6HFW7\_16\_395 | S | F | Y | - |
| 058 UniRef90\_A0A0P9B999\_30\_403 | - | - | - | - |
| 059 UniRef90\_A0A140K6F0\_14\_392 | S | - | - | - |
| 060 UniRef90\_A0A1P8EKI7\_5\_392 | S | F | Y | - |
| 061 UniRef90\_A0A2U3MYZ3\_7\_392 | S | F | Y | - |
| 062 UniRef90\_A0A1H2EPX5\_7\_397 | T | F | Y | - |
| 063 UniRef90\_UPI00041EF43F\_25\_400 | T | - | - | - |
| 064 UniRef90\_D0IW93\_17\_384 | - | - | - | - |
| 065 UniRef90\_A0A395D1I6\_11\_388 | S | F | Y | - |
| 066 UniRef90\_A0A1H0TK45\_27\_400 | S | F | Y | - |
| 067 UniRef90\_A0A1H8MYU5\_23\_403 | G | I | - | - |
| 068 UniRef90\_A0A1B1M254\_5\_403 | T | W | - | - |
| 069 UniRef90\_A0A484THM3\_28\_401 | - | - | - | - |
| 070 UniRef90\_A0A0Q8Q8X6\_8\_377 | - | - | - | - |
| 071 UniRef90\_A0A0Q5QDL2\_32\_410 | T | W | - | - |
| 072 UniRef90\_A0A1A5XM62\_22\_406 | T | - | - | - |
| 073 UniRef90\_A0A239EI90\_21\_397 | T | W | - | - |
| 074 UniRef90\_S5SWW2\_23\_403 | G | V | - | - |
| 075 UniRef90\_A0A3A5JJY9\_31\_413 | T | - | - | - |
| 076 UniRef90\_A0A315ZU85\_7\_384 | - | - | - | - |
| 077 UniRef90\_A0A3R9U899\_5\_381 | - | - | - | - |
| 078 UniRef90\_UPI000DD53D91\_7\_389 | A | - | - | - |
| 079 UniRef90\_UPI000DE4BE09\_13\_396 | - | - | - | - |
| 080 UniRef90\_A0A2T0R7P7\_10\_381 | - | - | - | - |
| 081 UniRef90\_A0A4R1HYS3\_27\_404 | T | W | - | - |
| 082 UniRef90\_UPI000D1537B0\_7\_385 | S | - | - | - |
| 083 UniRef90\_A0A1G6ZNM7\_17\_409 | T | - | - | - |
| 084 UniRef90\_UPI0003765980\_18\_389 | A | - | - | - |
| 085 UniRef90\_A0A071ICK8\_25\_398 | - | - | - | - |
| 086 UniRef90\_A0A2L0WNE5\_33\_396 | - | - | - | - |
| 087 UniRef90\_A0A1Q8CFJ2\_9\_380 | - | - | - | - |
| 088 UniRef90\_UPI000835F605\_21\_394 | - | - | - | - |
| 089 UniRef90\_UPI000374AB86\_31\_398 | - | - | - | - |
| 090 UniRef90\_A0A267RVL6\_22\_399 | R | - | - | - |
| 091 UniRef90\_M3VA40\_23\_395 | - | - | - | - |
| 092 UniRef90\_A0A395GID9\_32\_425 | S | W | - | - |
| 093 UniRef90\_UPI0008268C2F\_35\_406 | - | - | - | - |
| 094 UniRef90\_A0A0S1XV73\_27\_397 | - | - | - | - |
| 095 UniRef90\_A0A0F5N116\_19\_398 | - | - | - | - |
| 096 UniRef90\_A0A1N6Z7J9\_40\_406 | S | L | - | - |
| 097 UniRef90\_A0A2V4UWQ2\_31\_411 | S | L | - | - |
| 098 UniRef90\_A0A4P8KLT1\_5\_401 | - | - | - | - |
| 099 UniRef90\_UPI0003068598\_3\_292 | - | - | - | - |
| 100 UniRef90\_UPI000DD585C3\_27\_403 | - | - | - | - |
| 101 UniRef90\_A0A1E3SMN8\_25\_404 | - | - | - | - |
| 102 UniRef90\_UPI00045EBD18\_12\_396 | - | - | - | - |
| 103 UniRef90\_A0A1Y1ZNX6\_42\_434 | T | - | - | - |
| 104 UniRef90\_A0A0D2FPI6\_31\_423 | T | - | - | - |
| 105 UniRef90\_A0A2S0KGS2\_30\_403 | - | - | - | - |
| 106 UniRef90\_A0A1A7MGQ0\_54\_442 | T | - | - | - |
| 107 UniRef90\_UPI000413001D\_18\_392 | - | - | - | - |
| 108 UniRef90\_G7H5P9\_11\_400 | - | - | - | - |
| 109 UniRef90\_A0A010YHQ5\_11\_404 | S | - | - | - |
| 110 UniRef90\_B0RBU7\_24\_404 | - | - | - | - |
| 111 UniRef90\_A0A167RW81\_42\_429 | T | - | - | - |
| 112 UniRef90\_A0A1X0J8A0\_26\_405 | R | - | - | - |
| 113 UniRef90\_A0A2J6Q859\_43\_430 | T | - | - | - |
| 114 UniRef90\_UPI0003828F02\_7\_389 | G | G | - | - |
| 115 UniRef90\_A0A1B2HDJ6\_6\_361 | - | - | - | - |
| 116 UniRef90\_A0A2N3N0U1\_47\_427 | T | - | - | - |
| 117 UniRef90\_S5Y4X6\_32\_408 | R | - | - | - |
| 118 UniRef90\_A0A3R2WQN2\_17\_411 | S | W | - | - |
| 119 UniRef90\_A0A3N1X255\_20\_392 | - | - | - | - |
| 120 UniRef90\_A0A1S1LA29\_28\_404 | - | - | - | - |
| 121 UniRef90\_A0A081GNL4\_19\_392 | D | F | Y | - |
| 122 UniRef90\_A0A1I6U758\_27\_419 | S | - | - | - |
| 123 UniRef90\_A0A0F5VZP9\_7\_388 | - | - | - | - |
| 124 UniRef90\_A0A1H4GIM8\_20\_411 | T | - | - | - |
| 125 UniRef90\_A0A0M8TTT1\_8\_388 | - | - | - | - |
| 126 UniRef90\_A0A1Y2MDA3\_48\_437 | - | - | - | - |
| 127 UniRef90\_UPI000562F0C5\_9\_388 | - | - | - | - |
| 128 UniRef90\_A0A506Y8Z1\_43\_442 | T | - | - | - |
| 129 UniRef90\_A0A021VVW0\_28\_409 | T | - | - | - |
| 130 UniRef90\_E3QTS2\_46\_428 | T | - | - | - |
| 131 UniRef90\_A0A1D8SMV7\_7\_388 | - | - | - | - |
| 132 UniRef90\_A9BUV8\_38\_419 | - | - | - | - |
| 133 UniRef90\_A0A1N6TB45\_32\_407 | G | - | - | - |
| 134 UniRef90\_A0A381IE41\_24\_406 | - | - | - | - |
| 135 UniRef90\_A0A3M8TJ33\_18\_397 | - | - | - | - |
| 136 UniRef90\_A0A1H3JL41\_4\_373 | - | - | - | - |
| 137 UniRef90\_A0A507AW89\_49\_427 | - | - | - | - |
| 138 UniRef90\_A0A1X1SEN2\_20\_406 | G | F | Y | - |
| 139 UniRef90\_A0A2D3UFX4\_26\_403 | - | - | - | - |
| 140 UniRef90\_A0A089X0S9\_26\_403 | - | - | - | - |
| 141 UniRef90\_A0A2W5T1D0\_29\_413 | S | W | - | - |
| 142 UniRef90\_A0A1C4QGR9\_7\_388 | - | - | - | - |
| 143 UniRef90\_A0A101R4L6\_7\_388 | - | - | - | - |
| 144 UniRef90\_A0A4D4LHU8\_7\_388 | - | - | - | - |
| 145 UniRef90\_L1KK35\_6\_389 | - | - | - | - |
| 146 UniRef90\_UPI0005601C6B\_13\_390 | - | - | - | - |
| 147 UniRef90\_A0A1H9UQJ9\_6\_361 | - | - | - | - |
| 148 UniRef90\_A0A3D9JKY9\_12\_401 | - | - | - | - |
| 149 UniRef90\_D9X7Q2\_26\_403 | - | - | - | - |
| 150 UniRef90\_A0A1X1DXJ3\_20\_398 | - | - | - | - |

  
  

|  |  |  |  |  |  |  |  |  |  |  |  |  |  |  |  |  |  |
| --- | --- | --- | --- | --- | --- | --- | --- | --- | --- | --- | --- | --- | --- | --- | --- | --- | --- |
| |  |  |  |  |  |  |  |  |  | | --- | --- | --- | --- | --- | --- | --- | --- | --- | | 1 | 2 | 3 | 4 | 5 | 6 | 7 | 8 | 9 |   |  |  |  |  |  |  | | --- | --- | --- | --- | --- | --- | | **Variable** |  | **Average** |  | **Conserved** | | | |  |  |  | | --- | --- | | **X | - Insufficient data - the calculation for this site was performed on less than 10% of the sequences. |** |
