# Supplementary material for: Engineering DszC Mutants from Transition State Macrodipole Considerations and Evolutionary Sequence Analysis
Source: J Chem Inf Model. 2022 Dec 19;63(1):20–6. doi: 10.1021/acs.jcim.2c01337 (PMC9832474; doi:10.1021/acs.jcim.2c01337)
Supplement: Supplementary file 3 — ci2c01337_si_003.zip [file ci2c01337_si_003.zip › query_final_homolougs.html]

> 3x0y | Input\_pdb\_SEQRES\_A   
MTLSPEKQHVRPRDAADNDPVAVARGLAEKWRATAVERDRAGGSATAEREDLRASGLLSLLVPREYGGWGADWPTAIEVVREIAAADGSLGHLFGYHLTN  
APMIELIGSQEQEEHLYTQIAQNNWWTGNASSENNSHVLDWKVRATPTEDGGYVLNGTKHFCSGAKGSDLLFVFGVVQDDSPQQGAIIAAAIPTSRAGVT  
PNDDWAAIGMRQTDSGSTDFHNVKVEPDEVLGAPNAFVLAFIQSERGSLFAPIAQLIFANVYLGIAHGALDAAREYTRTQARPWTPAGIQQATEDPYTIR  
SYGEFTIALQGADAAAREAAHLLQTVWDKGDALTPEDRGELMVKVSGVKALATNAALNISSGVFEVIGARGTHPRYGFDRFWRNVRTHSLHDPVSYKIAD  
VGKHTLNGQYPIPGFTS
  
> UniRef90\_Q1W1G3\_1\_416 | Mutant DBT monooxygenase n=1 Tax=Gordonia sp. WQ-01A TaxID=378807 RepID=Q1W1G3\_9ACTN | E\_val=1.8e-256   
MTLSVEKQHVRPGDADNDPVAVARGLAEKWRATAVERDRAGGSATVEREDLRASGLLSLLIPRQYGGWGADWPTAIEVVREIAAADGSLGHLLGYHLSSA  
PMIELFGSQEQEQRLYRQIAQNDWWTGNASSENNSHVLDWKVSASPTEDGGYLLNGTKHFCSGAKGSDLLLVFGVIQDDSPQQGAIIAAVIPTSRHGVQV  
NDDWAAIGMRQTDSGSTDFHSVKVEPDEVLGEPNAFIVAFIQSERGSLFAPIVQLILANLYLGIAHGALDAAREYTRTQARPWTPARVQQATEDPYVLRA  
YGEFTIALQGADAAAREAAHLLQTVWDKGDALAPEDRGELMVKIPGVKALATNAALDVNSGIFEVIGARGTHPKYGFDRFWRNVRTHTLHDPVSYKIADV  
GKHTLNGQYPIPGFTS
  
> UniRef90\_T0BM21\_7\_392 | Uncharacterized protein n=1 Tax=Alicyclobacillus acidoterrestris (strain ATCC 49025 / DSM 3922 / CIP | E\_val=2.9e-109   
VQDDIWASVQRLAEAFAVDAVEREQAGGNAKRQRDMLRESGLLTLLIPERYGGGGETWSTVLRIVRELAKVDGSLAHLYGYHFLQLVAPHLVGTPEQKRY  
FYTESAKHNWFWGNAFNPLDQRLRGNREDGRVVLNGRKSFCTGAQDSDRLLVSWVEGDDPAALYTAVIPTRREGVLVHDDWDGLGQRQTDSGTVEFQDVV  
VEPHEIIAVPLANKSPFSTIDAPLSQMILANIFAGSATGALEAAKEYTRTQSRPWYTSGVNAAHRDPYTLRKYGDMWVELQGATSLLDAAGHSIDAAWEK  
EFSLTSDERGRVAVHTAAANALASKVALNVTSQVFEVMGAHSATKNHGFDRFWRNVRTHTLHNPIDYKFKNIGNWYVNDEPPKPGW
  
> UniRef90\_UPI0002AC58FE\_11\_391 | acyl-CoA dehydrogenase n=1 Tax=Synechocystis sp. PCC 7509 TaxID=927677 RepID=UPI0002AC58FE | E\_val=4.8e-100   
DYLTLATALAEEFAITAVERDAKGGTPKQERDRLRQSGLLKLIIPKEYGGLGETWITTLKISRELAKADSSIAHVFSYHHLGVIIPHIFGSEAQKEQYYS  
KTIENNWFWCNALNPLDRRTTLTPDGNNFRLNGVKSFCSGSKDSDILPITATNNDEITILAIPTQRQGINIQDDWDNMGQRQTDSGSIVFDNVVVYEDEI  
LALRDRTPTPFNTIRACLTQLNLANIYLGIAQGALAAAKQYTRTSTKPWLTSGVESATVDPYILQHYGNMWVELQGATCLTDIAGELLQNAWEQEWNLTA  
EQRGECAIAIATAKVAATKVGLDITSQIFEVMGARATTGKYGFDRYWRNLRTFTLHDPIDYKVLDIGNWLLNEQLPKPNFY
  
> UniRef90\_A0A3D5CW51\_20\_401 | Monooxygenase n=2 Tax=Methylophilus TaxID=16 RepID=A0A3D5CW51\_9PROT | E\_val=3e-99   
ALAVAKQLAESFSLTAVERDHQGGTPKLERDAIRNSGLLSLIIPRQYGGYGANWQETLQVVREFAKVDSSIAHVYGFQHLMLATVRLFSRPDQWERWYEQ  
TAQSNWFWGNALNPLDERTLCKSYDHWHEFSGKKSFCSGAMDSEMLIASAIKKTDGQLVIAAIPTLRTGVTILDDWDNIGQRQTDSGSVNFEKVRVEENE  
LLTEPGPLSNPFACLRPLIAQLILTNIYLGIAEGAFNDAQHYTLREARPWKNANVDITSQDPYILAHYGEFWVGLEATRALTNQAAEKLDIAWAKGLALT  
EEDRGLLATSISAAKVLATRTGLDITSRMFEVAGSRATHAGLRLDRHWRNLRTHTLHDPLDYKIKELGEWALTGSFPNPTFY
  
> UniRef90\_A0A2V4SPP5\_16\_400 | Monooxygenase n=6 Tax=Proteobacteria TaxID=1224 RepID=A0A2V4SPP5\_PSESJ | E\_val=2.6e-98   
TAARSGPLAIAADLAERLAATAVERDRAGGHPKHERELIRDSGLLTLSVPAEFGGQGADWATVMAAVRILAKADSALAHLFGFHHLQLAGVQLYGTELQQ  
RSLLTATVRQKLFWGNALNPLDKRTRAVASPAGYILDGVKSFASGSVGSDWLTVSAWDSEADAALIAVVPTEQAGVHVEADWDAFGQRQTDSGNVHFEKV  
YLPTALVLQAPGQAPTARSTLRSQVAQLVMANLYLGIAQGAFDAARDYTLEQARPWFASGVDDAIDDPFTQHRYGQLWLTVRPAALLADEAARQLDAAIG  
KGDAVTARERGVVAIAVAEAKVLAHRAAIEISNQMFELLGARATSSRFGFDRFWRNARVHTLHDPVDYKLRDLGRYALSGRVPDP
  
> UniRef90\_E0UIV5\_8\_393 | Acyl-CoA dehydrogenase type 2 domain protein n=1 Tax=Cyanothece sp. (strain PCC 7822) TaxID=497965 R | E\_val=1.4e-97   
TTNDHLNLAASLSQEFAATAIERDERGGTPKPERDRLRESGLLKLIIPKEYGGLAETWLTLFKITREFAKVDSSIAHIFSYHHLGVIIPHIFGTEHQKKQ  
SYLNTIKNNWFWCNALNPLDRRTTLTPEKNAFRLKGNKSFCSGAKDSDILPLTAIHQETGELTVLAIPTSREGIQINDDWDNIGQRQTDSGSISFNNVLV  
YPDEIFKNKEHSGKPFNTIRSCLTQLNLANIYLGIALGAFEAAKQYTKTTTKPWLTSGVESASEDPYILQHYGNLWVDLQAAAALVDQAGESLQATWEQE  
WLLTASQRGECAVAIATAKVAATRVGLEVTNRIFEVMGARATAKKYGFDRYWRNLRTFTLHDPLDYKLRDLGNWALNDQLPKPNFY
  
> UniRef90\_A0A1Z4S897\_9\_391 | Acyl-CoA dehydrogenase type 2 n=1 Tax=Nostoc sp. NIES-4103 TaxID=2005458 RepID=A0A1Z4S897\_9NOSO | E\_val=3.3e-97   
TDFLAIATTLAEEFAKTAVLRDAKGGTPTEELKKLRESGLLNLVIPKEYGGIGETWPNVLKVVREIAKTDGSVGQLLGYHYFNSAIPRFFGTPEQYAEFS  
KASARHNWFWSDAANPRDPDSILTPDGENFRLNGLKNFATGTKCSDVVLVGGRREDLGNVVYAVIPSDREGLQINDDWNYIGQRQTESGSVVFHNVLIRR  
DEILGNPDSNDDPRPFATLFTPLGQLVFVHLYLGIALGAFAEAKKYTLAHTRPWIISPAETAAQDPYIIEQYGNMWVDLAATISHADHVTLLAQAAWEKG  
EELTAAERGELAVSVASAKVLSTRVALDVTSKIFEVTGARSAANKYRFDRYWRNVRTHTLHDPVAYKVYEVGNWVLNGEIPDF
  
> UniRef90\_UPI00045E9273\_21\_396 | monooxygenase n=1 Tax=Methylosarcina lacus TaxID=136992 RepID=UPI00045E9273 | E\_val=1.8e-96   
AEQLAAEFAATAVERDRRGGTAKNERDSLRQSGLLNLIIPGEYGGHGLDWHDTLQIVRIISRADSSLGHLFGFQHLLLATLRLFGDQWPHYYRETVKNSW  
FWGNTLNPLDTRAKISADGqDWLVHGTKSFCSGASDSDHLIVSALTEAGGKLVIAAIPSDRPGIRIHSDWDNMGQRQTDSGTVDFDRVRIYDQEILSQPG  
PLGSVFATLRPLIAQLVLINIYLGIGEGALAEAIGYTRSQSRAWFLSGVESATRDPYVLKKYGEFWVDLNAAALATDYAASLLDAAWRQENALTEAERGS  
VAIASATAKVLATRAGLDVTQRLFEVTGARATSAKAGFDRYWRNLRTHSLHDPVDYKLRDLGEWVLNAKPPTPSFY
  
> UniRef90\_A0A353Y4M9\_17\_384 | Acyl-CoA dehydrogenase n=1 Tax=Curvibacter sp. TaxID=1888168 RepID=A0A353Y4M9\_9BURK | E\_val=1.3e-95   
SLAATAVERDRHGGHAAAERALLRDSGLLALAVPACFGGQGADWPTVLRAVRRIAAVDSALAHLFAFQHLQVASITFFGSEEQQADLLTRTVQERWFWGN  
ALNPADRRTTAAEFDEGLVLNGVKSFCSGAVGADVLLVSAYLECGRFIVAAIPAHREGVQIEGDWNAIGQRQTDSGNVIFTDVLVEPHELLQpGPGSSPW  
ATLRSCLAQSILVNIYLGIAEGADQSAREHTTRWRRPWISSGVEQATQDPYLLQHFGEFWVQLQAARALADEAAVKLEAAWQRGPQLMAEERGAVSVAVA  
TAKVLAHRASLDISSRLFDVAGTSALHAPLGLDRYWRNARTHTLHDPLDYKLRDLGQWALNGVVPASG
  
> UniRef90\_F3KR10\_17\_384 | Acyl-CoA dehydrogenase type 2 n=3 Tax=Hylemonella gracilis TaxID=80880 RepID=F3KR10\_9BURK | E\_val=2.6e-95   
SLAATAVERDRRGGHAAAERALLRDSGLLTLAVPAKFGGQGERWPVILRAIRRIAAVDSSMAHLFAFQHLQVASLSFFGSEAQQQEWLTRTVRERWFWGN  
ANNPADKRVTAVEFDEGLVLNGVKSFCSGAVGADVLLVSAHLECGRLIAATVPTDREGLQIQGDWNAIGQRQTDSGNVVFEDVLVKPEEVLNPgPGSSAW  
ASLRSTLSQSMLVNIYLGIAEGAHQAARDHTRQWRRPWVLSNVEQATQDPYLLEKFGEFWVQLKAAEALAEQAAQQIEAAWQRGEALTAEERGAASVAVA  
TAKVVAARAALDIGSRLFEVAGTSSLHAPLGHDRFWRNARTHTLHDPIEYKLRDLGNWALNDVLPPPS
  
> UniRef90\_A0A2D8NW56\_20\_404 | Monooxygenase n=1 Tax=Salinicola sp. TaxID=1978524 RepID=A0A2D8NW56\_9GAMM | E\_val=5.4e-95   
DGADHWLTVADSVAERLAETAVERDRRGGHAAEARELLRQQGLLRLSVPAWAGGVGASWSLIYHVVRRIARTDSALAHLLAFHHLQVGSVLLYGSRAQQS  
RLLGGTARENWFWGNTLNPLDRRTLASDRAAGGFLFHGDKGFCSGALGSDYLTASAWYEASQSLVVAAIPTGRRGITVHDDWDAMGQRQTDSGTVTFDSV  
VVEPEEVLIAPGAPWTPATQFRSCLAQLVLVNLYVGIAEGALEEARRYTLEQARPWLAAGVDRASEDPYTQRHFGELWVKLRCAEVLADLAGSVAQRHFA  
QGDAITAAGRGETAVAIAEAKVAAHQAVLDITSRLFDIAGARATQRRFGFDRFWRNARTHTLHDPVDYKIRDLGRWALNRHFPEP
  
> UniRef90\_A0A2W7M9P1\_16\_386 | Dibenzothiophene monooxygenase n=1 Tax=Psychrobacillus insolitus TaxID=1461 RepID=A0A2W7M9P1\_9BA | E\_val=1.6e-94   
LIAEISKTASERDKLGGTAKKERDLIRKSGLLRLASPKAYGGEKADWSTILHITREIAKVDSSVAHLFGYHFLCLASVELYGTAEQVSHFTKETAENDNF  
WGNAFNPLDIHVKAKKTANGWLVQGKKSFCSGATDSDRLLISAQKEDGSGVVIAVIPSIREGVIIGNDWDSFGQRQTDSGSVEFHNVEVKHSEVLEAFQI  
NDDNVFATVRTHIAQSILIHVLLGTAEGAFDVAKEYTKTQTRPWVTSNVDTAAEDPYHVYNYGELFVKLKAADALTKVSNEVLDKTWALKHEVTFEQRGE  
CSIAVATTKVQVVQTALDVTSRIFQVMGARSTSAQYNFDRYWRNVRTHTLHDPIDYKIRDIGQYTLNNIYP
  
> UniRef90\_A0A398AYR9\_12\_386 | Monooxygenase n=2 Tax=Bacillus asahii TaxID=228899 RepID=A0A398AYR9\_9BACI | E\_val=2.8e-94   
IVKSLTEEFAKTASERDKRGGTAKEERDLIRHSGLLRLTAPEQYGGYGENWKRVLHITREMAKVDSSVAHLFGYHFLCLASVELYGTPEQVDYFTKETVE  
NNYFWGNAFNPLDTHVTATKGDNGWVINGKKSFCSGAVDSDRLLISAQKDDGSGVLVAVIPTNRGGVLLGHDWDSFGQRQTDSGSVTFEQVIVHDAEVLD  
AYEATDSNLFATVRTHIAQSILIHVLLGTAEGAFEVAKDYTKTKTRPWVTSHVDAAINDPYNIYHYGDLFVKLKAADALVHISNELLDNTWALKTSITEE  
QRGECSIAIATAKVQVVQTALDVTSRVFQMMGARATSAQYNFDRYWRNVRTHTLHDPIDYKIRDLGQYTLNNQYP
  
> UniRef90\_A0A0T6UXN9\_11\_394 | Monooxygenase n=1 Tax=Pseudomonas sp. TTU2014-080ASC TaxID=1729724 RepID=A0A0T6UXN9\_9PSED | E\_val=5e-94   
APLQIARELAAQFAETAVERDARGGTPKAERDAIRASGLLSLIIPTQYGGLGATWSETFEVVREFARVDSSVAHVFGFQHLMLATVRLFASPAQWEPWLE  
LTARKNWFWGNALNPLDTRTVMKKFDGYYEFSGKKSFCSGATDSEMLIASAVDENASGKLVIAAIPTGRTGITLHGDWDNIGQRQTDSGSATFERVRVEH  
AELLLDPGPLSTPFACLRPLIAQLLFANMFLGIAEGAFAEARQYTLKESRPWFRSSARSSGEDPYILRHYGDFWVGLESSRVLIQRAVDQLDEAWRKEHA  
LTAEERAQLALTIGTAKVAASRNALDICNRLFEVTGARATHASLRFDRFWRNLRTQSLHDPVDYRIHELGDWALNGTRPTPSFY
  
> UniRef90\_A0A252E884\_11\_390 | Monooxygenase n=8 Tax=Nostocales TaxID=1161 RepID=A0A252E884\_9NOSO | E\_val=7.5e-94   
QDWIAIASSLAAELTATAVERDQKAGLPDIEIQRLRESGLLPLVVPKEYGGTGATWAEAFKVIQQLSTADGSIGQLYGNHLNLTTLAHVSGTPEQKERYY  
RQTAEKNLFWANAINTRDTRLKITPDGENFRVDGAKSFGTGVAIADLRVFSALQDGVEAPWLFVIPKDRPGVVSNQDWDNIGQRRTDSDTFTFHDVLVKK  
DEILGYPHPSDSGFATFLGIIAQLTKTYIYLGIAEGALAAAKEYTKTQTRPWITSGVDSASKDPYILRHYGEFWAELQAAIALSDRTAAQVQQAWDKGSD  
LTFEERGEVAIAVFSAKAFVTRVGLDISNGIFEVTGTRSTASKYGFDRYWRDLRTFTLHDPVDYKLNHIGNWLLNQELPI
  
> UniRef90\_A0A1Z4BZ71\_24\_403 | Monooxygenase n=2 Tax=Methylovulum psychrotolerans TaxID=1704499 RepID=A0A1Z4BZ71\_9GAMM | E\_val=1.6e-93   
AQAEQLSAYFYETAAERDKQGGTPLAQRQAIRDSGLLKLSIAKEFGGYGLAWPDIYKIIRIIARADSSLAHVFAFQFLMLASIRLYGSGEQWQSLFKETA  
AQNLWWGNALNPLDTRTIASEAAEHLLFNGFKSFCSGATDSDRLIVSAITADTKKFIVAAVPTQRAGIYVHDDWDNMGQRQTDSGSVEFKGLRVEKQELL  
INPGPLSSPFSSLRSMVAQLIFTNIYIGLAEGALQEGKKYTQTSSRVWSGSLAQTVQEDPYTLLHYGEFWASIDGARLLADHAAALLDNAWGKGLALTDA  
ERGEVALAVFSAKVNSTKAGLDVTSRIFEVAGARATTAKLGMDRFWRNLRVYTLHDPIDYKLRDLGDWALNGNYPAHSFY
  
> UniRef90\_A0A1Z4IGC5\_10\_387 | Acyl-CoA dehydrogenase type 2 n=2 Tax=Nostoc TaxID=1177 RepID=A0A1Z4IGC5\_9NOSO | E\_val=2.8e-93   
DYIKLAASLVPVFAQTAVERDKQGGTAKHERSHLRQSGLLKLIIPKEYGGLGETWITTLQIVRQFAQVDSSIAHLFGYHHLQVITPYLYGTPEQAQNYYT  
WTARHNWFWGNALNPLDKRLTLSSDGkNYRLNGLKSFCSGAQDSDMLTVSALPIGESQPVVVAIPTFSHGITVHDDWDNIGQRQTDSGSVSFANVLVKEF  
EILANPESIGTPFAALRIYISHLVRVNILLGIALGAFAQAKEYTTTTTQPWGASGVHSATADPYILQTYGKLWVDLNAATVLSDEAAEKFQAAWERGRQL  
TADEFGKTAVAISTAAAFTTKVGLEITNQVFELMGARATATDYGFDRYWRNLRTLTLHDPLAYKIQEVGKWALNREFP
  
> UniRef90\_A0A1W9JD13\_18\_401 | Monooxygenase n=2 Tax=Proteobacteria TaxID=1224 RepID=A0A1W9JD13\_9PROT | E\_val=4.3e-93   
KTPLEIAHDLSAEFAKTVAERDYLGGTPKAERDMLRESGLLSLTIPKEYGGQGASWHETLLVIREIAKTDSSLAHLFGFQHLMLATVRLFSKPEQWQRWY  
EQTAELSWFWGNALNPLDDRTISKSFNGWHEFSGKKSFCSGAIDSEMLIASAIKQKNGKLVIAAIPTSRTGVCVLGDWANIGQRQTDSGSVNFEKVRVEE  
NELLNDPGPLSTPFSCLRPLVAQLILTNIYLGIAEGAFADAKQYTLKEAKPWHTANIASTSQDPYILGHYGDFFVGIEATKALTDLAAHRLDEAWQKDSN  
LTENERGRVATCISAAKVMATRTGLDITSRMFEVTGSRATHAGLRLDRHWRNLRTHTLHDPLDYKIKELGEWALNNRFPEPTFY
  
> UniRef90\_A0A0B6S5D2\_8\_393 | Acyl-CoA dehydrogenase n=5 Tax=Burkholderia TaxID=32008 RepID=A0A0B6S5D2\_9BURK | E\_val=6e-93   
TAAAADPDAPRTFGELIVALRASAAERDRRGGHAAREKQWMAAVGLQTLAVPRAFGGQEAEWPIVYQTIRALARVDSALAHLVGFQVLQIVSVEVWGSDA  
QRERYLRGTVEQGWWWGNAVNSLDTRLVARQEADGGYRLDGIKSFCSGTRGSSRMTISAHDPATGKPVFAVVPTARDGITVHDDWDPIGQRQTDSGSVSF  
NGVRVEPDEVLHRSEVPPTPRATLRTLVSQLVLTNLFVGLAEGALAEAREYVGTRGRPWIHSGVERAQDDPYTLQRFGEMRVRAVAAEALADRAARALQG  
AWDRGQALTAEARAEVSLAVSEAKVVAQRAALESGEALFDACGARATAAPLALDRFWRNARTHTLHDPLDYRLRDLGRHALTGELP
  
> UniRef90\_A0A1Y3C786\_14\_396 | Monooxygenase n=1 Tax=Acinetobacter sp. ANC 3903 TaxID=1977883 RepID=A0A1Y3C786\_9GAMM | E\_val=1e-92   
SDVIPSAQFLAQQFAQTAIHRDRTGGTPKYERDLIRQSGLLALSIPQEFGGFSGTWKDTFDVVRIFAQTDSSIAHVFGFHHLLLATIRLFGNAQQWQRWY  
KVTAEKSWFWGNALNPLDKRTIVKHQNGWYEFSGKKSFCSGALDSEMLIASGIDENNGKLLIAAVPTSRSGITLYHDWDNIGQRQTDSGSSIFERVRVEA  
KDMLLDPGPLSTPFSSLRPLIAQLVFVSMFLGVAEGAFTEAKHYTRNESKAWFLSGVDQANEDPYILRHYGEFWLALESLKLLNQNAIAKLQQAWDIAEN  
LTEAQRGEVALAVATAKVAATRTSLDISNRIFEVTGARATHAALCLDRFWRNVRTQSLHDPVEYKIKDLGQWALNDIYPKASF
  
> UniRef90\_A0A318KD41\_20\_397 | Alkylation response protein AidB-like acyl-CoA dehydrogenase n=1 Tax=Rivicola pingtungensis TaxI | E\_val=1.2e-92   
AARLAARFAETAVERDQRGGTPKAERDALRDSGLLTLIIPREYGGLGAGWQDTLNVVRQFARVDSSVAHVFAFQHLLLATVRLFGRPAQWQPWFEATVRE  
RWFWGNALNPLDGRTEAQPDGDGYVFHGSKSFCSGALDSDMLVVSARQPGADKLLIAAVPSRREGITLFHDWDNMGQRQTDSGSADFKQVKVAHNELLLD  
PGPLSTPFSALRPLIAQLILSTIYLGIAEGALSEARRYTLSQRRLWPASLAQSANQDPYTLLHYGEFWVGLEGARLLVERAARQLDQAWRQGEALTADER  
GELAISIATAKVATNQVGLELTSRMFDVTGARATSAALRLDRFWRNLRTHSLHDPVDYKLQELGDWALNDAYPKPSFY
  
> UniRef90\_A0A352JDP6\_15\_389 | Monooxygenase (Fragment) n=1 Tax=Pseudanabaena sp. TaxID=1153 RepID=A0A352JDP6\_9CYAN | E\_val=2.2e-92   
ASVKKLADEFTTTAIERDRVGGTPKYERDRLRDLGLLKIAIPKAYGGWELPWHEVLRISREFAKVDSAIAHVYSYHHLGVTIPHIFGSKEQKEKFYTETV  
NNNWFWCNALNPLDRRSTLTRDGDiWILNGIKSFCSGSKDSDILPITAVNQETSELLALAIPTNREGVTIHDDWDNIGQRQTDSGSISFNQVEVFPHEIF  
GNRSQSDRPFKTIRACLTQLNLANIYLGIAIGAFETARNYTQTETRPFINTGIDSAIQDPYILEKYGDMWINLQATEALVDRAGLALQDAWSQEWDLTAQ  
QRGETAIAIATAKVSAHKVGLEVTNRIFEVMGARSTSAKYGFDRYWRNLRTFTLHDPVEYKIKAIGDWALNHQIP
  
> UniRef90\_A0A329B538\_13\_401 | Alkylation response protein AidB-like acyl-CoA dehydrogenase n=2 Tax=Microvirgula TaxID=57479 Re | E\_val=3.7e-92   
ARPRHDDIFAIADELAQRFAATAVARDQAGGTPKAERDALRRSGLLTLAIPREFGGLGASWSEMLTIVRRFARVDASIAHVFGFQHLMLATVRLFAGPAQ  
WQPWFEHTARRQWFWGNALNPLDPRTQARPRPGHYLFAGQKRFCSGALDSEMLIVSARHADDQRLLIAAVPTARSGITLLDDWDSIGQRQTDSGTVQFEQ  
VRVENSEILADPGPLTTPFSCLRPLIAQLTLTCVYLGIAEGAFDEARHFSLHEARPWHRSAAATVGEDPYVLNHYGEFWLGLESVRALVERAARMLDDAW  
ACGDALTADERGRLAVSIASAKVAATRIGLDVCTRLFEVTGARATHAALRLDRHWRNLRTQTLHDPVDYKLRELGDWALNQQPPAPSFY
  
> UniRef90\_A0A2N8QAF3\_13\_394 | Acyl-CoA dehydrogenase n=14 Tax=Burkholderiaceae TaxID=119060 RepID=A0A2N8QAF3\_9BURK | E\_val=5.2e-92   
LPTDAAPVVRDLAGLLKALRASAAQRDRDGGHAAQEKQWIADAGLLTLAVPRELGGLGARWPEIYETIRQIARVDSALAHLLGFTCLQVVSVNVWGNAEQ  
RARYLSGTVEGRWWWGNAVNPLDTRLVAGATRDGGYRLDGQKGFCSGTRGSHMMTVSALDPTTGKAVFAVVPTTREGITVHEDWNPIGQRQTDSGSVSFA  
SVRVEPHEVLARADTPYASLRTLISQQVLTNLFVGIAQGALEEAREYVTQYGKPWISSGVDQATDDPYLIQRFGEMRLQAVSAEALATRAAYALEDAWQK  
GPALDAETRARVALATSEAKIVAHRAALDVSEKLFDACGARATHAPLALDRFWRNARVHTLHDPLDYRVRDVGRYALSGTLP
  
> UniRef90\_A0A2N7XWT8\_11\_394 | Monooxygenase n=7 Tax=Pseudomonas TaxID=286 RepID=A0A2N7XWT8\_9PSED | E\_val=9.1e-92   
TPLEIARDLATEFARTAVDRDASGGTPKAERDKLRSSGLLSLSIPGQFGGLDANWSETFEVVREFARVDSSIAHVFGFHHLMLATVRLFATPAQWQTWFS  
LTASNNWFWGNTLNPLDTRTVVKRHTGWREFSGQKNFCSGANDSEMLIASAIDESAGGKLLIAAIPSARSGIILHNDWNNMGQRQTDSGSVTFKRVRVEE  
SDLLLDPGPLSTPFACLRPLIAQLHFANIFLGIAEGAFEEARQYTLEEARPWFRSSASHSTQDPYILSHYGDFWVALQSTRLLVERAGQVLDAAWAKGSA  
LTSEERGETAIAIATAKVAASRNGLELCSKVFEVTGARATNASVALDRHWRNLRTQSLHDPLDYKLHELGEWALNGTAPIPTFY
  
> UniRef90\_A0A2A4HLB2\_33\_412 | Monooxygenase n=2 Tax=Halomonas TaxID=2745 RepID=A0A2A4HLB2\_9GAMM | E\_val=1.2e-91   
LAITDQLAARLAESAVERDRLGGHASFERELIRDSGLLTLAVPTEYGGQGQPWSLVYHVVRRLARVDSALAHVFAFHHLQVGSVLLYGNQHQQQRLLTAT  
AQEHLFWGNTLNPLDKRTVATERGNGGFILQGKKGFCSGARGSDYLTVSAWHEPTQSLVIAAVPTHRPGITVHDDWDAIGQRQTDSGTVSFEQVEVLESE  
VLLPPGFTWSPSAQFRACLAQLVLVNLYVGIAEGAFEEAKRFTLEQGRPWLASEAAQAQDDPYLQRHFGELWVKLRSAQVLADIAGDIAQRHFVKRTAIS  
AENRGEAAIAIAEAKVVAHQAALDASSRLFDIAGARATARPLGLDRFWRNARTHTLHDPVDYKIRDLGRWALLDQYPVPT
  
> UniRef90\_A0A4R3HWL3\_41\_416 | Alkylation response protein AidB-like acyl-CoA dehydrogenase n=1 Tax=Paucimonas lemoignei TaxID= | E\_val=1.9e-91   
ASVLALTAPERDRQGGTAWAERQALRDSGLLTLAVPAAFGGPEASWPFIYRVIRRFAEVDSSLAHLFAFqHLQVASIILFANPVQKARYLGGTVSEGWFW  
GNGTNSLGNQVRLVWREEGGYyELSGTKTFCSGATGSDMLNVTAPHPERPDERVFFAIPTKRAGIHVLDDWDNLGQRQTDSGSVIFDNVRVERDEVLGPP  
GTAGTPRATLRTLVSQVILTEIYIGNAQGALQNAWKYTHEHAKPWMTSGVARAIDDPFVHQHYGDLWIALRSAIALTEAAEGQLQQAWERGDALTADERG  
RLAVTIYEAKLVAARAALEVTSKIFEVMGARATAAKYGFDRFWRNVRVHTLHDSLDYKLKDVGSWVLTGTVPAPSI
  
> UniRef90\_A0A1M7NXP6\_14\_394 | Acyl-CoA dehydrogenase n=1 Tax=Duganella sacchari TaxID=551987 RepID=A0A1M7NXP6\_9BURK | E\_val=4.2e-91   
YNPLSVAAELAQRLAATANTRDQAGGHAAQEREWIRESGLLTLSIPTAFGGQGADWPTVYQVIRILARADSALAHVFGFHHLQLAGIQLYGSAQQQRRLL  
TQTVEQNLFWGNALNPLDKRTTATDADYGFQLDGIKSFSSGSVGSDWLTISAWHAETQSALIGVVPARQHGVSVQADWDAFGQKQTDSGNVHFNQVSLPS  
TQVLQAPGVQATPQATLRSQVAQLIMANLYLGIGEGAFEAAREYLAKEARPWFASGVASAGADPLVQHRFGQLWLKLRPASVLADQAAQELERVFRLGSA  
VTAQQRGELAISVAEAKVLSHRAGVEVSSEIFELTGARSTSIKFGFDRFWRNARVHTLHDPVDYKIRDLGRYALDGTLPEP
  
> UniRef90\_A0A1B4ESK2\_22\_397 | Acyl-CoA dehydrogenase n=2 Tax=Burkholderia TaxID=32008 RepID=A0A1B4ESK2\_9BURK | E\_val=5e-91   
DALARAIDALRATAVARDRAGGHAADEKQRLADAGLLTLAVPREFGGQEAEWPAIYDTIRRIARVDSALAHLVGFQILQVVSVDVWGSAAQRERFLRGTV  
EHRWWWGNAVNPLDTRLVATATPDGGYRLDGVKGFCSGTRGSQRMTVSAHDPETGRAVFGVVPTDRDGITVNDDWDPVGQRQTDSGSVRFDGVTLAPDDV  
LHRSETPPTPRATLRTLVSQLVLTNLFVGLAEGALAEAREYVRQHGRPWIHSDVERAEDDPYTLQRFGDMRVRGVAAASLADRAAIALQHAWARRDALTA  
DERAEVALAVSEAKIVAQRAALDNGEALFDACGARATAASLGLDRFWRNARTHTLHDPLDYRLRDVGRFALTGELP
  
> UniRef90\_A0A1H6NKV2\_11\_394 | Acyl-CoA dehydrogenase n=4 Tax=Pseudomonas TaxID=286 RepID=A0A1H6NKV2\_9PSED | E\_val=6.7e-91   
DPLQTARLLAAEFAETAVERDERGGTPKAQRDALRHSGLLALSIPTQYGGLGARWSETLSVVREFAKVDSSIAHVFGFHHLMLATVRLFSRPEQWQPWFE  
QTARKNWFWGNALNPLDTRTVVRKLDGWREFSGKKSFCSGASDSQMLIASAVDESAGGKLLIAAIPSGRSGITLHNDWHNMGQRQTDSGSASFERVRVEE  
SELLLEPGPLSTPFACLRPLIAQLTFSHMFLGIAEGAFEEARRYTLTETRPWFKSGTEDIRNDPYILSHYGDFWVALEGVRLLVERAAEGLDQAWAKGPE  
LSAEERGQLAIAIATAKVAASRQGLELCSKLFEVTGARATHASLRLDRHWRNLRTQTLHDPLDYKLQELGDWALNQTLPIPTFY
  
> UniRef90\_A0A0D0KUF8\_11\_394 | Monooxygenase n=1 Tax=Pseudomonas fulva TaxID=47880 RepID=A0A0D0KUF8\_9PSED | E\_val=1e-90   
SPLQTARKLAADFAETAVERDEAGGTPKAERDAIRQSGLLALSIPTQFGGLGASWTETLGVVREFAKVDSSIAHVFGFQHLMLATVRLFSRPEQWQPWYE  
QTARKNWFWGNALNPLDTRTIVKKFDGWREFSGKKSFCSGATDSEMLIASAVDESAGGKLLIAAIPSGRTGITLHDDWNNMGQRQTDSGSATFERVRVEE  
EELLLDPGPLSTPFACLRPLIAQLHFSHIFLGIAEGALEDARHYTLKEGRPWFRSKAQHTSEDPYILRHYGEFWVGLEGVRLLVERAAAQLDEAWSKEHA  
LSAEERARLAISISTAKVAASRTGLDICNRLFEVTGARATHASLRLDRHWRNLRTQTLHDPVDYKIQELGDWALNQTRPTPTFY
  
> UniRef90\_A0A0F3K7B4\_20\_401 | Monooxygenase n=7 Tax=Aquitalea TaxID=407217 RepID=A0A0F3K7B4\_9NEIS | E\_val=1.8e-90   
ALLLAQQLAAQFAATAAERDHAGGTPKAERDAIRASGLLGLSIASELGGLGANWQQTLLVVREFAKADSSVAHVFAFHHLMLATVELFGQPEQWQPWHEI  
TTRQNWFWGNALNPLDERTVSRRFDGWHEFSGQKSFCSGALDSQMLVVSARQQHSGQLVVAAIPTGRSGISVQQDWNNIGQRQTDSGSVNFEKVRIENNE  
VLDNPGPLTTPRSCLRPLLAQLILTNIYLGIAEGAFEEARHYTLHEARPWRAAQVDHTHEDPYILGHYGEFWVGLESVRALTDRAGNSMVAALARGEALT  
QDERGEVAMHIAAAKVAATRMGLNLACQMFEVTGARATHAGLRLDRHWRNLRTHTLHDPVDYKLKELGEWALIRNYPTPTFY
  
> UniRef90\_A0A381IMG6\_13\_394 | Flavin-dependent monooxygenase, oxygenase subunit HsaA n=99 Tax=Burkholderiaceae TaxID=119060 Re | E\_val=2.9e-90   
EADREPRTLAELIAALRASAPERDRAGGHAAREKRWIADAGLLTLAVPREFGGQEAGWPVIYHTIRALARVDSALAHLLGFQCLQIVSVDVWGSAAQRER  
YLRGTVEHDWWWGNAVNPLDARLVAHATGDGGYRLDGVKGFCSGTRGSQRMTISAHDPDTGKPVFAVVPTEREGIVVRDDWDPIGQRQTDSGSVSFDGVR  
VEPDEVLHCSETPPTPRETLRTLVSQLVLTNLFVGIAEGALAEARDYVQRAGRPWVHSGVDRAADDPYTLQRFGDMRVQAVSAEALADRAARALQAAWAK  
KEALTADARADVALAVSEAKIVAQRAALDVSEALFDACGARATAAPLALDRFWRNARTHTLHDPLDYRLRDVGRYALTGVLP
  
> UniRef90\_A0A2S9K1G1\_26\_400 | Monooxygenase n=1 Tax=Malikia granosa TaxID=263067 RepID=A0A2S9K1G1\_9BURK | E\_val=5.2e-90   
EALAQALAANAVERDRAGGHAAAERELIRASGLLDLTTPCAYGGWGQSWQTFYAGLRRIAQADSSLAHLYAFHHLQVATILLYGKPAQHEALLRSTVEQR  
LFWGNALNPNDKRALATEDGtGWRISGPKSYCSGSVGSDRLTLSAWHEPSQSLLIGALPSDRAGVTIRADWDAFGQKQTDSGTVTFDQVRLEPHEVLVQP  
GQVATPRATLRPMLAQLILTNLYAGIARGALQEGLRYTRDASRPFFASGVSRAVDDPYVQQRYGQLAVLVRPAELLADLAAQAIDQALALGDAVTAADRG  
RVAIAVAEAKAVAHRAAVEVSSQIFELTGASATSARFGLDRFWRNARVHTLHDPIDYKLRDLGRHALTGSFPEPS
  
> UniRef90\_A0A1W6L7W5\_17\_392 | Monooxygenase n=2 Tax=Rhizobacter gummiphilus TaxID=946333 RepID=A0A1W6L7W5\_9BURK | E\_val=7.5e-90   
MADRLALRLATTAVARDQAGGHAAEERQWIRDSGLLSLTIPAAHGGQGADWPTFYRTLRRLAEADSALAHVYGFHHLQLASVQLYGSPEQHARYLRGTIE  
HGWFWGNALNPLDKRLVATEVDGGYRLDGLKGFASGALGSDQLVVSAWLPDREALLVGVVPTGRDGVAVQGDWDAFGQRQTDSGTVTFDQVALAHAEVLQ  
APGTVPTPQATLRSQLAQLILVNLYTGIARGAFDAARRYTVEESRPWFASGVSRAADDPFVQHRYGDLWLKVRPAIVLADDAAQRLDQAFRRGAALTAAD  
RGEVALAVAEAKVLAHRAAIEVSSQLFELTGARSTSTRLGLDRFWRNARVHTLHDPVDYKLRTLGRHALLGELPEP
  
> UniRef90\_A0A255HJE3\_14\_398 | Monooxygenase n=2 Tax=Janthinobacterium TaxID=29580 RepID=A0A255HJE3\_9BURK | E\_val=1.5e-89   
PAAPPDAIAVATFLAARLAATANARDQAGGHAAEEREWIRDSGLLTLSIPAQFGGQGAPWPLVYQVIRILARADSALAHVFGFHHLQLAGLQLYGSAQQQ  
RRLLTLTVDERLFWGNALNPLDKRVTATDSGDGFVLDGIKSFSSGSVGSDWLTVSAWHAPTQTALIAALPTRQPGITVQPDWDAFGQRQTDSGNVHFEQV  
ALPAELVLQSPAQAATPQTTVRSQIAQLIMTNLYLGIAEGAFEAARGYTSEQAKAWFASGVAQAADDPLVQHRYGQLWLLLRPAQVLADLAALELDTVLR  
KGALVTAQERGLLAVAVAEAKCLSHKAGLEISSQMFELTGARSTSAQFGYDRYWRNVRVHTLHDPVDYKLRDLGRYALSGTVPEP
  
> UniRef90\_A0A0R3AD45\_11\_394 | Monooxygenase n=26 Tax=Pseudomonas TaxID=286 RepID=A0A0R3AD45\_9PSED | E\_val=2.8e-89   
SPLQTARQLAAEFALTAVERDERGGTPKTERDALRQSGLLALSIPTQYGGLGARWSDTLAIVREFAKVDSSIAHVFGFHHLMLATVRLFSRPDQWQPWFE  
QTARKNWFWGNALNPLDTRTQVKDFGGWrEFSGKKSFCSGASDSEMLIASAVDESAGGKLLIAAIPSGRSGITLHNDWNNIGQRQTDSGSASFERVRVEE  
SELLLDPGPLSTPFACLRPLIAQLTFTHMFLGIAEGAFDEARNYTLTETRAWHKSTAEDVRQDPYVLHHYGEFWVALQGVRLLVERAAALLDQAWAQGPN  
LSENERGQLAIAIATAKVAASRQGLELCSRLFEVTGARSTHASLRLDRHWRNLRTQSLHDPVDYKLHELGDWALNQSLPTPTFY
  
> UniRef90\_A0A1W6ZB60\_16\_389 | Monooxygenase n=1 Tax=Bordetella genomosp. 13 TaxID=463040 RepID=A0A1W6ZB60\_9BORD | E\_val=6.1e-89   
EALAAQLAATAVERDRAGGHAAHERELIRASGLLALSVPTEFGGAQAPWSVTLAAVRRLAQADSALAHLFGFHHLQVAGVLLFGRPEQHEYFLAPTVRRK  
LFWGNALNPLDRRVLAANEGEGFRLDGVKSFSSGSVGSDILTLSAWHKPTETALIAALPTDADGITVNPDWDAFGQRQTDSGTVRFDHVRLDPVQILQAP  
GVTPTPRATLRSQVAQLIITNLYLGIAQGALAEARRFVHDDARPWFAADVARHADDPYVQERFGEFRLAVRAAEALADQAGIRLDAALARGDAVSAAERG  
EVAVAGAEAKVWAHRAALRLGTELFEVTGARSTSARYGYDRYWRNARVHTLHDPVAYKVRDLGRYALDGRAPEP
  
> UniRef90\_A0A2X1DPB7\_14\_397 | Acyl-CoA dehydrogenase n=49 Tax=Burkholderiaceae TaxID=119060 RepID=A0A2X1DPB7\_9BURK | E\_val=9.1e-89   
TDAPAVDRAALARAIDALRVSAAERDRAGGHAAQEKQWLADAGLLTLAVPRAFGGQEAAWPAIYDVIRQVARVDSALAHLVGFQCLQVVSVDVWGNAAQR  
ERYLRGTVDGRWWWGNAVNPLDTRLVATATPDGGYRLDGVKGFCSGTRGSQRMTVSAHDPAIGRTVFGVVPTDRAGITVNDDWDPVGQRQTDSGSVRFDG  
VTLAADEVLHRSEAPPTPRATLRTLVSQLVLTNLFVGLAEGALAEARDYVRQHGRPWIHSDVERAEDDPYTLQRFGDMRVRAVAAAALADRAAHALQHAW  
ARQDALTADERAEVALAVSEAKIVAQRAALDNGEALFDACGARATAASLGLDRFWRNARTHTLHDPLDYRLRDVGRFALTDALP
  
> UniRef90\_A0A0S9M2D1\_4\_380 | Monooxygenase n=4 Tax=Comamonadaceae TaxID=80864 RepID=A0A0S9M2D1\_9BURK | E\_val=1.4e-88   
AQALATRFAATAAERDRRGGTPKAERDALRDSGLLALSIPRAYGGHGGDWQLTLRVVRILAAADSSLAHVFGFHHLMLATVRLFGQRAQWEPWFAQTARH  
AWFWGNALNPLDARTVATPQDGWHAFTGQKSFCSGALDSQMLVASALHARTRQLLVAAVPTARSGIQAAPDWDNMGQRQTDSGSVTFEKVRVEDAELLTD  
PGPLSTPFACLRPLVAQLVLANVYLGIAENAFEDARRYTLHEARPWPASPAAQAGDDPYVLAHYGEFWVGLEGVRALADRAAGRLDEAWRRGEQLDAATR  
GEAAVAIAAAKVAATRVGLDLCTRMFDVAGARATHGGLRLDRHWRNLRTQSLHDPADYKLRELGEWALKRQHPHASF
  
> UniRef90\_A0A238ZKW1\_13\_401 | Acyl-CoA dehydrogenase n=1 Tax=Methylobacillus rhizosphaerae TaxID=551994 RepID=A0A238ZKW1\_9PROT | E\_val=2e-88   
PADTLKSSLEIARELAAEFRLTAVERDQAGGTPKIERDLIRNSGLLALSIPGTEGGLGANWQETMQIVREFARVDSSIAHVFAFHHLMLATLRMFGRPDQ  
WQPWFRHTARLNWFWGNALNPLDARTTSRQVKHWHEFSGKKSFCSGAMDSEMLIVSAYAQDTKELLIAALPTARTGINVLQDWHNIGQRQTDSGSVNFER  
VRVEKDDLLQEPGPLSTPFSCLRPLLAQLILTNIYLGIAEGAFEDARHYTLNEARAWHLSGVALPEDDPYTLAHYGEFWASLQSARALTNLAAAQFDAAW  
EQQASLSEEARGKVAIEIGAAKVVTTRIGLDITSRMFEVAGSRATHAGLRLDRHWRNLRTHTLHDPVDYKIKELGEWALKYQYPRPSFY
  
> UniRef90\_A0A0D1P8V6\_10\_394 | Monooxygenase n=7 Tax=Pseudomonas TaxID=286 RepID=A0A0D1P8V6\_PSEPU | E\_val=4.6e-88   
STPLQIARDLAQTFSRSVVERDRKGGTPKAERDALRASGLLALSIPREFGGLNATWSQTFDVVREFARVDSSIAHVFGFHHLMLATVRLFGAPSQWQPWY  
ELTARKNWFWGNALNPLDTRTIVKHHGSWrEFSGKKSFCSGASDSQMLIASAVDEAAGGKLLIAAIPSGRTGITLHDDWHNMGQRQTDSGSATFERVRVE  
EGDLLLDPGPLSTPFACLRPLIAQLHFANICLGIAEGAFEEARQYTLNETRPWFKSTALDSRDDPYVLGHFGDFWVRLQGARLLLDNAAGELDRAWAKGA  
ALGADQRGRVAVAIATAKVAASRDGLELCSKVFEVTGARATQASVGLDRHWRNLRTQSLHDPLDYKLHELGDWALNQRLPIPTFY
  
> UniRef90\_A0A261SP68\_11\_387 | Monooxygenase n=1 Tax=Bordetella genomosp. 10 TaxID=1416804 RepID=A0A261SP68\_9BORD | E\_val=7.5e-88   
AVVDDLVKKLAASAVERDRQGGHAAAERELIRASGLLLLSIPREHGGLETDWSTILGIVRRLARVDSALAHVFGFHHLQIAGVLLSGGPAQHAHFLPPTV  
EGNLFWGNALNPLDRRAIAAEEPGGFRLDGVKSFSSGSIGSDVLTISAWHRASGTALVAALPTDTQGVTVNPDWDAFGQRQTDSGTVRFDHVRLEFLQVL  
QAPGFSPTPRSTLRTLFSQAIMANLYLGIAQGALAEACRFVLEDARPWFAADVQRQADDPYVQARFGEFRLATRAAEALADAAGRQLDAALGRGAALTAA  
ERGELAVAIAEAKIWSHRAALKVGNELFEATGARSTSARYGYDRFWRNARVHTLHDPVAYKIRDLGRYELDGRAPEP
  
> UniRef90\_A0A2N6MRB2\_11\_390 | Monooxygenase n=2 Tax=Fischerella TaxID=1190 RepID=A0A2N6MRB2\_9CYAN | E\_val=1.6e-87   
KDWIAIASSLSAELTTTAVERDTKAGLPDVEIQLLRESGLLPLVVPKEYGGTGATWIEALRIVQELSKADGSIGQLYGNHLNLTALGHVSGTSEQKERYY  
RETATHNLFWANAINTRDSRLKITPDGEHFRVNGIKSFGTGVAIADLRVFSAVQDGIEVPWLFIIPKDRTGVVSEQDWDNIGQRRTDSDTFTFHNVLVKK  
DEILGYPHPPNHPFSTFLGIIAQLTKTYVYLGIAEGAFTAAKEYTKTQTRPWITSGVDSANQDPYILHHYGELWTQLQAAIALSDRTLSQVQQAWEKETS  
LTFEERGEVAIAVFTAKTFATRVGLEITNRIFEVMGTRSTASKYGFDRYWRDLRTFTLHDPVDYKLRDIGNWVLNQELPL
  
> UniRef90\_A0A212BVA1\_31\_405 | Monooxygenase n=2 Tax=Pseudomonas TaxID=286 RepID=A0A212BVA1\_9PSED | E\_val=3.2e-87   
KVERLARKLAENAVARDRQGGHARAERELIRESGLLALAIPQRFDGLEKPWPQIYRIVRHLAAADSSLAHLFAFNHLQVATILLHGSTEQQRHWLSRAAH  
ERWFWGNATNGRDLGLQLQKREEHFELNGRKSFCSGALGADALVVSAPRGGSTSERAFLVLPTQREGLAINDDWDAFGQRQTDSGTVQFENVFVDRGELL  
VSGGQSPRSSLRVCVSQLILTQLYLGNSQAALDGALRYVREQARPWLGAGVNEASEDPFIQKRFGELWLLYRGALLQAELAAERLQQAWDKPALSAAERG  
EVALLIAEARVSSARASLEITSQVFETMGARATASSYGFDRFWRNVRVHSLHDPLDYKVRDIGQWLTRSVPPTPS
  
> UniRef90\_A0A1A9KE82\_11\_394 | Monooxygenase n=14 Tax=Proteobacteria TaxID=1224 RepID=A0A1A9KE82\_9PSED | E\_val=4.6e-87   
NPLQIARQLAAGFAENAAERDERGGTPKAERDAIRHSGLLALSIPGAFGGHGADWSLTLEVVREFARADSSVAHVFGFHHLMLATVRLFSRPEQWQPWFE  
LTARKNWFWGNALNPLDTRTQVKWFDGWCEFSGKKSFCSGANDSEMLIASAVDESAGGKLLIAALPSGRSGIGVHDDWDNMGQRQTDSGSVTFERVRVER  
SELLLDPGPLSTPFACLRPLLAQLIFAHMFLGIAEGAFEEFRGYTLKEARPWFRSSAESISEDPYVLRHYGDFWVGLEGVRLLVERAAAQFDAAWGKEQR  
LGAEERAELAIAIGTAKVAATRVGLELCSRLFEVTGARATHAALRLDRHWRNLRTQSLHDPLDYRVQELGAWALNRQRPSPTFY
  
> UniRef90\_A0A4Q5PVB9\_26\_400 | Monooxygenase n=1 Tax=Comamonadaceae bacterium TaxID=1871071 RepID=A0A4Q5PVB9\_9BURK | E\_val=9.9e-87   
EKVAEELARTAATRDKTGGTALAERRLLRESGLLTLAVPGEHGGHGAGWPLIFRIVRRFAQADSSLAHLFAFQQHQVAAVILFGSPAQQENYLGKTVSER  
WFWGNAVNARDTRLAATRTDGGWRLDGVKAFCSGAKDSDVMNVSIATGPVPADRLYAVVPTGRDGITVNDDWDNMGQRQTDSGSVTFSNVFVGEAEILGP  
PGNATSPRASLRNMLGQAVLTEIYLGNAQGALQQAVDYTRTQVQPWAMAGVSRAVDDPLLQLRAGQLWASLRAAIALAEEANAKFQWAWDRGRDLTEAER  
GEAALLVAAARTTAAKAALQVTADIFELMGARATTSANGFDRYWRNVRVHTLHDPVDYRSKALGQWMLTGELPDP
  
> UniRef90\_A0A178GPM9\_9\_392 | Monooxygenase n=7 Tax=Acinetobacter TaxID=469 RepID=A0A178GPM9\_9GAMM | E\_val=1.6e-86   
NQSPVDIAQKLAENFALTAAERDKQGGNPKAERDLIRQSGLLGLSIPKQYGGQEADWQTIFKTIQIMAQVDSSLAHVYGFHHLLIATVQLFSQAEQYGPW  
FEQTAQNNLFWGNTLNPLDRRTTATKVSENEYIFHGDKSFCSGSIDSDMLLCSGYNDAGKLLIGVIPTQRKGVSFLGDWNNMGQRQTDSGTSHFEQVKIH  
QNELLLNPGPLSTPYSSLRPLIAQLIFVHLFLGVAEGAFDVAKQTVQTQKAWSKSLVEDAVNDPFIQKHFAEFYVQLEGVRLLADKAVQALQAAWELGND  
LSAEQRGEVSVAIATAKIAATNTSLYITQNIFQVMGARATTAKLNLDRFWRNVRTQTLHDPIDYKYQEVGEWVLTGKVPDPSFY
  
> UniRef90\_A0A1P9YC32\_3\_390 | Monooxygenase n=7 Tax=Burkholderiaceae TaxID=119060 RepID=A0A1P9YC32\_9BURK | E\_val=4.2e-86   
RTQDAPWNEPLEAVLREWARTAAARDKTGGTAFHERRLLRESGLLRLSVPVEHGGDGAPWSVTLDVVRRMARVDSSLAHLFAFHHLQLATIRLFGSDAQI  
RAWLGETVTQGWFWGNALNPLDKSTRALPDGAGGYVFDGRKSFCSGARDSDRLLASAFDGDGRLLIGVTPTNRAGIAVLDDWDNMGQRQTDSGTVVFERV  
PIAKDELLLQPGPLSSPFATLRPLLAQAILSHIYLGIGEGALQAAREATLAQTRPWIASQAERPADDPYVLANYGDFWIALEGARLLVERAMQAFDGAWR  
RGLALTEGERGEVAVAVAASKVAAARAGLDVAHRMFEVTGARSTTAALRLDRFWRNVRVHTLHDPLDYKVRELGDWALNERIPKPSFY
  
> UniRef90\_UPI000A1773A9\_40\_416 | monooxygenase n=1 Tax=Derxia lacustris TaxID=764842 RepID=UPI000A1773A9 | E\_val=6.2e-86   
DELVARFAETAPSRDLLGGTPKLQRDQLRRSGLLALSIPTAFGGLGGDWKLVLDVVRRFARVDSSVAHVFAFHHLMLATTQLFARPEQWEQWFRHTARHD  
WFWGNALNPLDRRTVCRRLDGWSEFSGRKSFCSGALDSEMLIVSGHRETDGSLVVAAVPTGRSGIAVIQDWDNIGQRQTDSGSVDFERVRVDDAELLLDP  
GPLTTPRSCLRPLIAQLILTNIYVGIAEGAFNEAREYTLREARPWHVTGLERADDDPYVLAHFGEFFVGLESLRLLADRAGAQLDLALARGTALTADERG  
TLAVSIATAKVAATRTGLDICNRLFEVTGARSTHAGLGLDRHWRNLRTHTLHDPVQYKIRELGEWALKSHLPAPSFY
  
> UniRef90\_A0A158L201\_25\_395 | Monooxygenase n=1 Tax=Caballeronia choica TaxID=326476 RepID=A0A158L201\_9BURK | E\_val=1e-85   
VAALRETAEARDREGGHAAREKQLIADAGLLTIAVPREFGGEGARWVDVYETIRALARVDSALAHLLGFTCLQVVSVTVWGNEAQRERYLRGTVAHRWWW  
GNAVNPLDTRLIAHATGDGGYRLSGQKGFCSGTRGSQMMTLSAHDPATGKPIFAVVPTTRAGITVHDDWDPVGQRQTDSGSVSFDDVELRDDEVLHRSEV  
PPTPRATLRTLVSQLVLTNLFVGIAEGALQEARDYVAKNGRPWIHSNVTRAADDPYQLHRFAEMRLKAVSAEALATRAAQALDAAWERGDSLEAGERAEV  
ALAVAEAKVLAHRAALDVSQSLFDACGARATHGPLALDRFWRNARVHTLHDPLDYKLRDIGRFALSGVLPE
  
> UniRef90\_A0A4Q4GT15\_13\_391 | Monooxygenase n=1 Tax=Acinetobacter piscicola TaxID=2006115 RepID=A0A4Q4GT15\_9GAMM | E\_val=2.2e-85   
EIAQQLAHTFAKTAAERDKAGGNAKVERDLIRQSGLLSLSIPKQYGGQGADWATIFQTVRTIAQVDSSVAHIYAFHHLLIATVQLFAQPTQYAKWFEDTA  
KNNLFWGNTLNPLDRRTKATQVSEQEFIFHGDKSFCSGSIDSDILLCSGFNEAGKLLIGVIPTTREGVSFLGDWNNMGQRQTDSGTSHFEQVKISKDELL  
LNPGPLSTPYSSLRPLIAQLIFVHLFLGVAEGAFDIAKEMVQSQKAWSKSLAENAVNDPFTQKHFAEFYVQLESVRLLANKAVETFQSAWDIGEGLNAEQ  
RGEVSIAIATAKIAATNTSLFVTQNIFQVMGARATTAKLNLDRFWRNVRTQTLHDPIDYKYQEVGEWVLTGKVPEPSFY
  
> UniRef90\_A0A1C0YC62\_17\_381 | Monooxygenase n=1 Tax=Caryophanon tenue TaxID=33978 RepID=A0A1C0YC62\_9BACL | E\_val=6.6e-85   
TTFAKTAAKRDKQGGTAYEERQLLRQSGLLYLFQDGDIPWQTVLRITRQLATVDSSLAHLFGYHYLCIATIELYGTQAHYERAITQGTQHQHFYGNAFNP  
LDTHVTATKQADGQWLLQGTKYFCSGASDADCLLVSAQKKDGSGLLMAVIPANRAGVHIHNDWDSFGQRQTDSGAVTFNDVSILPHECLALHTEEQHYFS  
TCRTHIAQTILLHVLLGTAEGAFQEAKSYTKTSARPWVTAHVNDATEDPYILQHYGTFFAKLQAADALLERAVQTLVTTLHKKHHVTEEDRGICSVAIAT  
AKVQVVETTLHITSTMFQVMGARATNAQYNFDRFWRNVRTHTLHDPIDYKLRDLGQYALNDRYPT
  
> UniRef90\_A0A1H7FE04\_32\_405 | Acyl-CoA dehydrogenase n=1 Tax=Roseateles sp. YR242 TaxID=1855305 RepID=A0A1H7FE04\_9BURK | E\_val=1.8e-84   
LAQRLDETAVARDQAGGHAAAERELIRASGLLALTVPQALGGLGGGVTELFHAVRVVARVDSALAHVLGFHHLQLYGVSLYGGPAALEHGArhlRETAEQ  
RLFWGNALNPADTRLRATLAPGGWRLDGAKSFCSGALGSDRLTVSATTPDGGFLTGVVPTRRQGVAVEQDWDAFGQRQTDSGTVRFDNVSLADEELLQVP  
GQAPTPRATLRSQIAQLVMTHLYLGIAEGAFEQARRYTATQTKAWFASGVERAVDDPLVQHRYGDLWLHVRAAQGIAREAVSALHHALLRGDALTAQERG  
ELAVAGAEAKVLAHRAALQVSSQLFELTGARSTSARFGFDRFWRNARVHTLHDPVDYKVRDIGRYRVDGRIPDP
  
> UniRef90\_A0A401MWC4\_5\_385 | Acyl-CoA dehydrogenase n=2 Tax=Streptomyces TaxID=1883 RepID=A0A401MWC4\_9ACTN | E\_val=2.5e-84   
DWIATARAVAADLAQDAAARDKANKPPFEEAERLRAAGLLTLLVPAEYGGGGADWRTAYAIIREIAAGDGSIGQLLGYHYLLSWNPRFFGEPATVERLER  
AAAGGQWLWGGAFNPRDPDVTLTPDGDGFRLDGRKSFATGARVADRLVVGATRTHTGEPVVVFLDPAHPGVVRNDDWDNFGQRLSASGSVEFDAVPVAAD  
DVFGSLAQDEGTLSPFATLVTPAIQLVFVHFYLGIAEGALAAAADYTRTTTRPWLLSDVESATQDPYTLATYGELAVSARSVRVLADHAAEAVQRGLDRG  
PDLTADERGEIAVTVATAKVAATKAALDITARILEVTGARSTASAHGFDRFWRNARTHTLHDPVAYKLREVGDHFLNGTHP
  
> UniRef90\_A0A1G6HFW7\_16\_395 | Acyl-CoA dehydrogenase n=1 Tax=Burkholderia sp. TNe-862 TaxID=416944 RepID=A0A1G6HFW7\_9BURK | E\_val=4.8e-84   
PVVDSVAEELERTAVARDRAGGTALHERRVLRDSGLLGLSVPRAYGGHGASWSDTLAVVRRFAQVDSSLAHLFAFQHLHLATVRLFGRPRQWEPWFEQTI  
AQRWFWGNALNPLDQGTRAVPDRAGGWYFVGRKSFCSGAADSDRLLASAFDEQGRLLIGVVPTSRKGIGVLDDWDNMGQRQTDSGTVVFDQVAVAGNEVL  
ADPGPLSSPFACLRPLLAQLILAHIYLGLGEGALRTARDFTRASSRAWIASPASHPTEDLYVQGHYGRFWLALEGARVLVGRAASAFDSAWRRETALTET  
ERGKVAIAVAAAKVQSAQAGLDVTQRMFEVTGARATTAALRFDRFWRNLRVHTLHDPIDYKIRELGDWALNERFPDPSFY
  
> UniRef90\_A0A0P9B999\_30\_403 | Monooxygenase n=4 Tax=Variovorax TaxID=34072 RepID=A0A0P9B999\_VARPD | E\_val=8.7e-84   
ALANTLAASAAERDRAGGTALAERALLRRSGLLTLAIPAAHGGQQAAWPLIFRILRRLAQADSSLAHLFGFQHLQVASVLLFGSEAQQARFLGEAVEKRW  
FWGNAVNARDTRLQAARTDDGIEIDGVKGFCSGAPDSDVLNVSVVLGPEPTDRLFAVVPTSRAGITVLGDWDNMGQRQTDSGNVEFRRVRIAHDEILGPP  
GVASSPRATLRNLIGQIVLTEIYLGNAFGALRAAIEHLRAHTQPWPMAGVERVEDDRLLQLRAGEMWSALQAATALSNQANERFQQAWEQGFALTADSRG  
ALAIDVAAARTQAARTALHVTSQIFELVGARGTASKHNLDRYWRNVRVHTLHDPLDYRHQGIGAWLLAGDVPNP
  
> UniRef90\_A0A140K6F0\_14\_392 | Acyl-CoA dehydrogenase type 2 domain protein n=1 Tax=Stanieria sp. NIES-3757 TaxID=1807358 RepID | E\_val=2.5e-83   
QVVAEELSEAIAASALERDQKAAIPEAEINLFKKSGLLLLSIPKKYGGMEASWLEVYRVIQTISKADGSSGQLYANHITLVTLGEVMGRLGQAEHFYRRT  
CENNLFWANAVNARDARLKITPDGNNFLVNGIKSFGTGVAVGDLNVIGASMEGVETPIVFILPKNREGISYNYDWNNMGQRCTASGSYTFNNVQVFPKEI  
VGPPPIPESAFPSLIFLVTQLSKTFVYLGIAEGALKAAREYTLTSTRPWISSGVERASLDPFILHHYGEFWTELQAAIALAERAAHQIDEAWQKGVSLTF  
QERGSIAIAVSAAKAFAAKVGLNITTRMFEVMGSRATSNRYGFDRYWRDQRTFTLHDPIDYKLLDVGNWLLNDIFPTPS
  
> UniRef90\_A0A1P8EKI7\_5\_392 | Monooxygenase n=9 Tax=Acinetobacter TaxID=469 RepID=A0A1P8EKI7\_9GAMM | E\_val=9e-83   
YFAQTFDPLVQAQSLATAFEKTAAVRDKQGGTAKIERDLIRASGLLSLSIPKAYGGQGADWETIFKTIQLIAQVDSSLAHVYGFHHLLIATVQLFARPEQ  
YENWFRQTAQQQLFWGNALNPLDRRTQVQPLNDDTFIFQGEKSFCSGSIDSDMLLCSGYTQEDKLLIAVVPTQRDGIRFLGDWNNMGQRQTDSGTTHFEQ  
VKILKHELLLNPGPLSTPYSSLRPLIAQLIFVHLFLGVAEGAFKVAHQAVQHQKAWATSSATQSVDDPYIQKHFAEFYVQLESVRLLTNKARTLLQNAWN  
EGPDLSAQQRGEVSVAVATAKIAATRTGLMLTQDMFQVMGARATTAALNLDRFWRNVRTQTLHDPIDYKYQEVGEWVLTGKVPHPSFY
  
> UniRef90\_A0A2U3MYZ3\_7\_392 | Dibenzothiophene desulfurization enzyme C n=1 Tax=Acinetobacter sp. KPC-SM-21 TaxID=2126983 RepI | E\_val=1.5e-82   
SSEFNAIQTAEQLAEQFAQTAAERDKKGGNPKFERDLIRQSGLLALSIPEQYGGQGANWDTIFQTIRAIARVDSSVAHVYGFHHLLIATVQLFAQPEQYK  
KWFEDTAKNNLFWGNTLNPLDRRTTAQQISENEFIFHGDKSFCSGSIDSDILLCSGYDSASKLLIGVIPTQRDGVSFLGDWNNMGQRQTDSGTSHFEQVK  
IYKDELLLNPGPLSTPYSGLRPLIAQLIFVNLFLGVAEGAFTVAKETVRQQKAWSSSLVDQAVNDPYTQKHFAEFYVQLESVRLLTQKAIETIQWAWNIG  
ENLTAEQRGKVSIAIATAKIAATNSSLFITQNIFQVMGARATTAKLNLDRFWRNVRTQTLHDPIDYKYQEVGEWILTEQVPKPSFY
  
> UniRef90\_A0A1H2EPX5\_7\_397 | Acyl-CoA dehydrogenase n=1 Tax=Verrucomicrobium sp. GAS474 TaxID=1882831 RepID=A0A1H2EPX5\_9BACT | E\_val=3.4e-82   
LSTAETRSAAVGALAETFARTAAERDRLGGTAQAERDLLRASGLLTLAIPEALGGQGAPWPEILGHVRRFAAVDGSLAHLFGFQHLLLATVALFGSEAQQ  
ESFFRRTVEGRWFWGNALNPLDPGTLASATEGGRRINGRKSFSSGSVDADALVVSASDEAHGGKLLVGVLPLAerRDGVRVLADWDAFGQRQTDSGAVLF  
ENVLLRDDEILRTPGPLGDIRATLRPLLAQSILVHVYLGLAEGAFAEALPLASAATRPWLASGVERPQDDPYLLARHGEIALALRSAALLAEAAARSLRH  
TWEKGENLTEAERGEAALDIAAAKIATTRAALDIPSKIFDLVGAKAAVHAARLDRFWRNARLHTLHDPIDYKVRELGVHALLGTHPKPTFY
  
> UniRef90\_UPI00041EF43F\_25\_400 | monooxygenase n=1 Tax=Azohydromonas australica TaxID=364039 RepID=UPI00041EF43F | E\_val=1.5e-81   
TEALARALEESAVARDQRGGHAHEERELIRGSGLLDMTTPHAFGGWGHSWQLFFQGLRRLAQVDSALAHVYAFHHLQVATVLLYGTPEQHEFFLRPtVGE  
RLFWGNALNPNDKRTLAHDDGDGFIVHGPKSYCSGSVGSDQLTFSAWHEASQSLLIAALPSRHPGISIQGDWDAFGQKQTDSGTVTFNQVRVQAHQVLVR  
PGTVPRPRATLRSQIAQLVLVNLYAGIAQGALSQGLRYTRDSSRPFFASGLARAVDDPYVQHRYGELSVLVRPAEVLADLAAHKLDQALALGEDVTASDR  
GELAVAVAQAKAVAHRAAIDVSTQVFELTGAGATASRLGLDRFWRNARVHTLHDPLDYKLRDLGRHALLGRYPEPT
  
> UniRef90\_D0IW93\_17\_384 | Acyl-CoA dehydrogenase, type 2-like protein n=20 Tax=Comamonas TaxID=283 RepID=D0IW93\_COMT2 | E\_val=2.7e-81   
KTAVARDRQGGHAAAEKALLRDAGLLRLAIPREHGGDALSWPDIYRHVRALAAVDSALAHVLAFHQLQVATVLIYGSRQQQRLwLRRTIDENGWWGNALN  
PRDTRLQALPRSSELAGGYVLDGLKGFCSGTRGSHYLTVSASVAGHAQPVLGLLETASPGIAVKDDWNPMGQRQTDSGSVQFNRVQLPASAVMRDESAEV  
TAFHTLRNCLAQLVLVNLFVGVAQGARRQARDYAREHGRPWLGSAVERATDDPYLLRRMGEMQAQISGAALMADHGAELLQKAWQRGPALSAAERAEVAI  
AVFEAKVMAHRCTLFATQEMFDVVGSRGTHADLGFDRFWRNVRTHTLHDPLDYKLQALGRWAVHGEEP
  
> UniRef90\_A0A395D1I6\_11\_388 | Monooxygenase n=1 Tax=Methylocystaceae bacterium TaxID=2052174 RepID=A0A395D1I6\_9RHIZ | E\_val=9.9e-81   
PNAAVEALVADFSATAAVRDAAGGTAKRERDLIRESGLLRLSIPVELGGAGADWVETLGVVRRLASVDSSLAHVFAFHHLMLATFQFFGSPAQAQEFLRR  
TAQDGWFWGNALNPRDPRTQLQAVEGGYRLDGHKSFCSGASDSDMIIVSALDRETKALRVAAIPTRRDGVLVHGDWDNMGQRQTDSGTVSFDNVFVAESE  
LLLSPGPLGSPFASLRPCLAQLVLTNIYLGLAQGALEQAKTYLHGAPAEIRErVGQDPYALRNFGELWVEIAAARALTDEAQRAFQQGWEAGADTTPELR  
GDIAVTIAAAKVASSRASVEVGSRIFDLMGARSTAAAHRFDRFWRNARTHTLHDPLDHKLRELGSFAFEGQFPTPSFY
  
> UniRef90\_A0A1H0TK45\_27\_400 | Acyl-CoA dehydrogenase n=2 Tax=Rhodoferax TaxID=28065 RepID=A0A1H0TK45\_9BURK | E\_val=1.5e-80   
TQALAATAVARDQRGGTAKAERDAIRASGLLGLSVPTALGGLGASWQQTLEVVRGFARVDSALAHLFAFHHLLLATTQLFGRPAQWQPWIEQTLAQRWFW  
GNALNPLDKSTHALQQADGSYTFTGRKSFCSGASDSDMLLASALDVHGRLLISVVPTARAGIRVLNDWDNMGQRQTDSGSVIFDQVAVAADELLTDPGPL  
STPYACLRPLLAQLILAHIYQGLGEGALAEARQFTLQQGRPWMASGAASAQQDPYILGHYGDFWLALEGSRLLLQNAAAAFDQAWASGTALTEVERGAVA  
IAVAAAKVATTRASLDVVHRMFEVTGARATTAALRLDRYWRNLRVHTLHDPVDYKLRELGDWALNETLPHPSFY
  
> UniRef90\_A0A1H8MYU5\_23\_403 | Putative acyl-CoA dehydrogenase YdbM n=4 Tax=Rhizobium TaxID=379 RepID=A0A1H8MYU5\_9RHIZ | E\_val=3.4e-80   
NPYLAKAIELREIFAVDAVERDQQGGRPSDQIRRLKESGLVNLLIPREFGGEGQPYSTALRIVREFAKVDGSLAHLFGYHFSPIQNAVTSGRDERSAAIL  
RRSAEGRWFWGNTTNSFSKSLFGRKVEGGVLLNGDRPFASGSHVADYLMVAWEDEKTNARRFAYIPANRPGITIADDWDGIGQRQTGSGRVFYKDVRVRD  
DEILSERPKDPSELLIPqQQQSVLLNVFVGSAQGALNAARDYTVTTSRPWIYSGVERHSDDPWIKRQYGELWTKVQAATALADRAAAAMDSAYAKGGALT  
AQERGATAIAVASANVLAGNVALEVTSEIFEVMGARSAVRPLGFDRFWRNVRIHTLHNPAEYKTRNVGAWFLTGDYPEPGI
  
> UniRef90\_A0A1B1M254\_5\_403 | FMNH2-dependent monooxygenase n=26 Tax=Streptomyces TaxID=1883 RepID=A0A1B1M254\_STRLN | E\_val=5.9e-80   
TPTHWQTGPAPRTAQDWIARAAEVAAVLATDAAERDHAGATPYAEVQLLKDSGLVTLLGPTEHGGAGQDWPTAYRVVREVAKADGSIGQLLGYHYLWFWA  
ARLVGTREQWEHVEAEASRNRWFFGGAVNPRDKDVIVTEDGDdLVFTGRKSFSTGSKVSDVTVLEGVLDGTDQHVFAIVPSDSEGLTFLDDWDNIGQRLT  
ESGGVTLDGVRTPWSSAAGYVDKQFKPRVYNTLNVPTIQLVFVNFYLGIAAGALETAATYTREKSRSWLHGGHERAVDEPYVIDTYGDLTAKLWAVEALA  
DAVAAEGQKLHDDPDAVTEQARGDFEVRVAAVKARATDVSLEVAGRVFEVTGARSTATAEGLDRFWRNVRTHTLHDPVAYKRREVGRWVLEGELPEPTW
  
> UniRef90\_A0A484THM3\_28\_401 | Acyl-CoA dehydrogenase probable dibenzothiophene desulfurization enzyme n=8 Tax=plant metagenome | E\_val=1.9e-79   
AKVEALAVALASTAAQRDREGGSPQAERQRLRDSGLLTLAVPREHGGAGERWPVLLRIVRRLAQADSSLAHLLAFQHLQVASVILFGSAAQQDtYLRATV  
SQGWFWGNAVNARDTRLEVTRTADGYRLDGIKGFCSGALGSDVLNVSVALGPAPTDRLFLVVPTSRAGIVVNDDWDNMGQRQTDSGTVVFNGVQVGLDEA  
LGPPGAASSARATLRNVIGQLVLTELYLGNALGALDEAVGRVRTQVQPWAASGVAHASDDGLLQLRAGDLWLQLAGATALADQALDAFQAAWDSGAAFDF  
DARGELGMLVAAARVQAGRTALRVTEQIFDLVGARATTRQLGLDRYWRNVRVHTLHDPLDHRQQAIGRWLLTGL
  
> UniRef90\_A0A0Q8Q8X6\_8\_377 | Acyl-CoA dehydrogenase n=1 Tax=Noviherbaspirillum sp. Root189 TaxID=1736487 RepID=A0A0Q8Q8X6\_9BU | E\_val=6.9e-79   
LLATLRATAVERDRTGGHAAAEKELIRAHGLLGLSIPRHYGGDGLPWPVIYNCVREIATVDSALAHVLAFHHLQIVTVLIYGSDAQKERwLVDTMARRCW  
WGNAMNPLDLRLTAEDRGDGLVLNGQKGFCSGTRGSSYMTLSAVHVSSRRTVLGVVPTGHHGISILDDWDPIGQRQTDSNSVSFANVPLSGGNLLRRHDE  
EQTFYQSLRTCFAQLVLVNLYLGIAMGAFEEARRYLLEQGRPWIHAGVARATDDPYMIHRFAEMHVQISAATVLADRAAAMIEEAFLLGRDLGGQQRAAV  
AIAIAQAKVVCHRAGLHAAQELLEVAGARGAKAELGFDRFWRNVRTHTLHDPLDYKLSVLGKWALTGESP
  
> UniRef90\_A0A0Q5QDL2\_32\_410 | Monooxygenase n=2 Tax=Williamsia TaxID=85043 RepID=A0A0Q5QDL2\_9ACTN | E\_val=2.6e-78   
AHEVASILAADAVERDRAGATPHAEVALLKESGLVTLLGPVEHGGGGQNWVTAYEVIRAVAAGDGSIGQLLGYHLLWFWAARLVGTREQIEAVETAATQN  
KWFFGGAVNPRDSDVTITDNGDTIsYTGRKSFSTGSHVSDVTVLEGVLETTGAHIFAIVPSNSEGLTFHHDWDNLGQRLTESGSVTIDNVTTEWASAAGF  
VDKEFQPRVYNTLNVPIIQLVFINFYLGIARGALQTAADYTRDTTRPWLHGGFDRAVDEPYVIDTYGDLTTKLWAAEALADAVAVEQQKIHDNAWDVTAS  
ERGAHEVRVAAAKAAATDVALEITTRIFEVTGARSTSSKVGLDLFFRNVRTHTLHDPVAYKRREVGRHQLIGELPEPTW
  
> UniRef90\_A0A1A5XM62\_22\_406 | Acyl-CoA dehydrogenase n=4 Tax=Paraburkholderia TaxID=1822464 RepID=A0A1A5XM62\_9BURK | E\_val=5.1e-78   
TLAATLRESAARRDLAGGHAAAEKQLIADAGLLALAVPREFGGAfddafddatgaqnGARWPEIYRTIRTLARADSALAHLLGFQFLQIVSVDLWGSAAQ  
RADWLRGTVEHRWWWGNAVNPLDTRLVAQADGEGGWRLHGKKGFCSGTYGSQMMTVSAHDPATGQPVFAVVPTTRAGITVHDDWDPIGQRQTDSGTVSFD  
AVEVRAHEVLKRPDTPRASLRTLVSQLVLTNLFVGIAEGALEEAREYVLANGRPWVNSGVQRAADDPYLQQRFGEMRIQALSASLLADRAASLLDAAWAQ  
GDALGADTRAEVSLAVSEAKIVAHRAALAAGEQLFDACGARATAAALAFDRFWRNARVHTLHDPLDYRVRDVGRHALTRELPEPT
  
> UniRef90\_A0A239EI90\_21\_397 | Acyl-CoA dehydrogenase n=1 Tax=Rhodococcus kyotonensis TaxID=398843 RepID=A0A239EI90\_9NOCA | E\_val=3e-77   
EVAQLLAVDAVARDKGDAPPVDEVQLLKDSGLVTLLGPVEHGGGGQDWPTAYQVIRRVAAADGSIGQLLGYHLLWFWAARLVGTPEQIEAVEADATSNKW  
FFGGAVNPRDSDVVVTDEGDTIVFEGAKTFSTGSRVSDVTVLEGALSGSDAHVFAIVPSNIEGLTFHDDWDNIGQRRTESGSVTIDKVRVDWSSAAGFVD  
KQFQPRVYNTLNVPTIQLVFVNFYLGIARGALEEAAAYTKDKTRAWLHSTVDKAVDEPYIIDIYGDLTSKLWAVEALADAVALEANEIHEDAWNVTPEQR  
GDHEVRVAAVKARASEVALEITSTVFEALGARATASKFGFDRFWRNVRTHTLHDPVAYKRREVGRWVLTGELPEPTW
  
> UniRef90\_S5SWW2\_23\_403 | Acyl-CoA dehydrogenase protein n=1 Tax=Rhizobium etli bv. mimosae str. Mim1 TaxID=1328306 RepID=S5SW | E\_val=9.1e-77   
NPYLAKAVELREIFARDAAERDRLGGRPREQVRHLKESGLVNLLIPKEFGGEGQPYSTALRIVREFAKVDGSLGHLYGYHFGPLQNAATAEADPRSRDIL  
RRFAAGRWFWGNTTNSFSNSLFGKRDAKGVVLNGVRPFASGSHVADYLLVAWENEETNERSFAHIPANREGIVIADDWDGIGQRQTGSGQVFYHNVRIEA  
GEILPERPKRPAALLGPQqQQSVLLNVFVGSAQGALIAARDYTVTRSRPWIYSGVERHSDDPWIKRQYGELWAKVQAATALADRAAEAMDAAFAKGGALK  
AEERGKTAIAVAAANVLAGKVALEVTSEIFEVMGARSAVRPHGFDRFWRNVRIHTLHNPAEYKARNVGTWFLTGDFPEPGV
  
> UniRef90\_A0A3A5JJY9\_31\_413 | Monooxygenase n=1 Tax=Salinisphaera sp. Q1T1-3 TaxID=2321229 RepID=A0A3A5JJY9\_9GAMM | E\_val=2.5e-76   
ADWLAIADDVAARLDITAAERDAVGGHAAAERALIRGSGLLDLSIPAAIGGAGQSWATIHGVVRRLARADSAVAHVLAFHHLQIATVMLYATPDQQRRLL  
QRTVRENLFWGNALNPRDRRCRAYRHPSGGYVFNGDKSFCSGSVDADYLTVSAYDEASDALVIAAIPGDRAGLDIRGDWDAIGQRQTDSGSIGFNRVRIT  
AEDVLLAADAPTTTRHQLRSCLAQLVLVNLYVGLAEGALATAREMTTTRARPWAAADVEAAIDDPYVQHRYGRLWVRLRAAQLLANHAAQAVDALFFRDG  
SISAAERGDAAIAVAEAKVAAHDISLEAGSELFELGGTATvARPRHLDRFWRNARTHTLHDPVDYKLRDLGRWAISGTYPEPT
  
> UniRef90\_A0A315ZU85\_7\_384 | Alkylation response protein AidB-like acyl-CoA dehydrogenase n=2 Tax=Actinobacteria TaxID=201174 | E\_val=6.3e-76   
TLAARDAAAKLAVDALERDRANAEPLAEAQLLREHRLPGLLLPVEVGGGGGTWADALRAVREVAVADGSIAQLLGYHYVNQANLDWVADAATRERWQRRS  
GAAQWLWGDAVNPVDPDLTLTPAGQGGYRLNGRKSFATGVSSGDVSVVSGQDSATGRHVLVVVERGAAGVQPGGDWDNLGQRLSASGSVTFTDVEVPAEN  
VLGFLDGDAATPRASLVTPAIQAVFGNFYVGVVRGALQTAAEYTRTTSRAWLLSDSPSAAQDPYVLATYGRLVARLRAAEALADSAGEALTAADAAGDAL  
TWDERGELAELVAALKVVSSDLAVEATSAVFEVTGARATSNRVGLDRFWRNVRTHTLHDPVQYKAREVGDHFLNGTHP
  
> UniRef90\_A0A3R9U899\_5\_381 | Monooxygenase (Fragment) n=1 Tax=Streptomyces sp. WAC00469 TaxID=2487415 RepID=A0A3R9U899\_9ACTN | E\_val=1.1e-75   
TPPDFTTRPTPRTAADWIARAGEVAAVLATDAVERDRAGATPYAEVQLLKDSGLVTLLGPVAHGGGGQDWRTAYRVVREISKADGSIGQLLGYHYLWNWA  
ARLVGTREQWEHIEAEAARNRWFFGGAVNPRDKDVVVTEDGDdLVFTGHKTFSTGSKVSDVTVLEGVFEGTDRHVFAVVPSDSEGLTFHDDWDNIGQRLT  
ESGSVTLDGVRTPWSSAAGYVNKQFQPRVYNTLNVPTIQLVFVNLYLGIAGGALETAAAYTRTRTRPWLHGGHERAVDEPYIVDTYGDLTAKLWAVEALA  
DAVADEGQKLHDDPDAVTEQARGDFEVRVAAVKARATDVALEVTNRIFEVTGARATASALGLDRFWRNVRTHTLHDQ
  
> UniRef90\_UPI000DD53D91\_7\_389 | monooxygenase n=6 Tax=Rhizobiales bacterium TaxID=1909294 RepID=UPI000DD53D91 | E\_val=2.5e-75   
ADDTLLAKAIELRQAFHRDAVERDKAGGRPVDQIRLLKESGLASAQIPRDYGGRGASWLTILRIVREFARTDGSLAHLFGYHHLPLNHILFRGSTAQRQR  
WLTGSVAGNWFWSNSGNALSKTSSGERSATGWIINGSRPFSSGSHVADYIQISWENADGARLTAAVPADRDGIVIENDWDGIGQRQTGSGTVRFVNLEID  
DDELIGSPDLPLTPFSSLTSLLQQSVLLNVFVGSAQGILDEGRNYTTTSSRPWIYSGVERHIDDPWIKRQYGDLFVRALAASELADKAARSLDAAYGEGP  
LLTAAGRGAAAIDIATANVYAGDVGLAVSSEVFEVMGARSATTENGFNRFWRNVRTHTLHNPAEYKKRTVGTWLLTGEFPVPA
  
> UniRef90\_UPI000DE4BE09\_13\_396 | acyl-CoA dehydrogenase n=1 Tax=Rhizobiales bacterium TaxID=1909294 RepID=UPI000DE4BE09 | E\_val=8.8e-75   
NSSREDLFSRAEAISADLATRAAELDRQGRPPLAEIVKLKNSGLLNALHAPEIGGGGLDWVDGLKLVRILARGESSIGQLLGYHFVNSQYVDWAFDEARA  
RALGSEtVAKSLYWGAAVNPRDPGLVLSRRGNGYVLNGRKSFSTAAHVSDYINANASIGDRIVGFAVPTSRAGYTANDDWDNFGQRLSDSGSVEFHDFPV  
YEEDFVTSPTAPDTAPSVLSTFNTPLIQLVFVNFYLGTAEGALQAAIDYVRTTTRPWVTSGVQRASDDPYILERVGELTAALKASAALADSAAAAVQAGL  
LRGEQVTARERGEAAAEAYAAKVHATHTSLDVTSRVFELTGARSTAEHYRFDRFWRNVRTHTLHDPVFYKAREVGEFVLNDTIP
  
> UniRef90\_A0A2T0R7P7\_10\_381 | Alkylation response protein AidB-like acyl-CoA dehydrogenase n=1 Tax=Kineococcus rhizosphaerae T | E\_val=2.8e-74   
AARDVANHLALDALERDRANAEPFTEAELLRKAGLPSVLLPASIGGAGLPWSVALEVVREIARTDGSIAQLIAYHYVNAHNLVWVADDAGRARWGVPSVA  
NQWLWGDSVNPVDPDLRLARDGEDWVLSGTKNFSTGASVGDVTLVGGLTDDGRDLLVLVPRETPGFVKGGDWDNLGQRLSASGSVRFDDVRITPDAVLGS  
ASSSGAFGSLVTPSIQAAFGHFYLGVTRGALEAASEYTRTTSRPWLLSDVEKAVEDPYVLATYGRLVARLRAAEALGRSVGESLSQAHARGADLTWAERG  
EVAEEIAALKVVSSDLAVEATSAIYEVTGARATANKHGFDRFWRNVRTHTLHDPVQYKAREVGNHFLTGAHP
  
> UniRef90\_A0A4R1HYS3\_27\_404 | Alkylation response protein AidB-like acyl-CoA dehydrogenase n=1 Tax=Pseudonocardia endophytica | E\_val=7.1e-74   
REVRRALETDAAARDRAGETPYDEIALLKRSGLVTLLGPVEHGGAGLEWPAAYRVVREVAAGDGSIGQLLGYHYLWFWAARLVGTPEQIEAVEADATRNQ  
WFFGGAVNPRDDDVTIRDTGDEIVYNGRKSFSTGSRVSDVTVLEGVLEGTDTHVFAIVPSAQDGIVFGDDWDNIGQRLTESGSVTISDVTVPWASAAGFV  
DKAFQPRVYNTLNVPVIQLVFVSFYLGIARGALETALAYTRTTTRSWLHSDAERAVDEPYQLDLYGDYQSKLWAVEALADQVALEGLAIHHDAWNVTPEQ  
RGAHEVRVAAVKARATDVALEITSGIFEGLGARATTSQLGFDRFWRNVRTHTLHDPVAYKRREVGAFLLRDELPEPTW
  
> UniRef90\_UPI000D1537B0\_7\_385 | monooxygenase n=2 Tax=Acidovorax avenae TaxID=80867 RepID=UPI000D1537B0 | E\_val=1.8e-73   
LARARDLADTLAPSAAARDRQGGRPCAEIALLKRSGLLEAMVPQAWGGGGHPWSAVLAVVREFARVDGSIAHLYGYHFLLMNMPVIAGQLAQAEALQRAS  
VRHRWLWGNAVSSRDPTLKGRESGTGHVLDGFRPFATGSHVADMLYIGWEDEAGAGKRFAAIPASREGVQIMDDWDGFGQTQTGSGSVRFHGVRVLPEEV  
VDmAPSRGKPVSTLLPQMSQSVLSSVFIGSALGALEEARRYAMTKSRPWLDAGVACHHEDPWVRRVFGDLWIRTEAARLLVEKADRALDALWEAGQDLTE  
AFRAEQACTIAAANVLAGEVALHVTSAVFEVMGARSATRANGFDRFWRNVRVHTLHNPAEYKTRNVGTWVLTGQYPPPS
  
> UniRef90\_A0A1G6ZNM7\_17\_409 | Acyl-CoA dehydrogenase n=1 Tax=Glycomyces harbinensis TaxID=58114 RepID=A0A1G6ZNM7\_9ACTN | E\_val=3.1e-73   
ATSAAWVARAREVAARLAVDAVERDREGATPYAEVQLLKDAGLVPLLAPAEHGGGGQDWTTAYRVIRAVAAGDGSIGQLLGYHYLWAWAARLVATPGQIE  
AVEKQAAEQEWFFGGAVNPRDDDLVIREEDGELVFNGRKTFSTGGKVSDVTVLEGILEGTDRHVFAIVPTDQDGIVFAGDWDNLGQRLTESGSALIEGVR  
VPWEAAAGYVaTEQGHEFAPRTYNTLNVPLIQLVFTNFYLGIAQGALDTAAAYTRERTRAWPYAADRKarAVDEFYIQETYGGLQSKLWAAEALADRAAG  
LIESINAHADAVTPEERGEAAVVIASAKQTTIDAGLEIGTRIFEVTGARASANAVGLDLFWRNIRTHSLHDPVAHKRAEVGRYALTGELPEPT
  
> UniRef90\_UPI0003765980\_18\_389 | monooxygenase n=6 Tax=Rhizobiales TaxID=356 RepID=UPI0003765980 | E\_val=1.4e-72   
LRAVFHLDAVDRDKQGGRPLEQIRILKESGLPSAQIAKAYGGQGASWLTILRIVREFAKTDGSLAHLFGYHHLPLNLILFRGSNAQKEKWLSGSAAGNWI  
WSNSGNAMSKTSTGERIKGGWTINGSRPFSSGSHIADYIQISFENSAGERLTAAVPADRPGIVIVDDWDGIGQRQTGSGTVHFHDLRIDDDEMISAPSVP  
LTPYTSLTSLLQQSVLLNVFVGSAQGILEEGREYTVGSSRPWIYSGVERHIDDPWIKRQYGELYIRTLATAELADKAARSLDAAYIQGPSLSHADRGAAA  
IDIATANLYAGEVGLAVSSEVFEVMGARSATQANGFDRFWRNVRTHTLHNPAEYKKRTVGAFVLTGEFPVPA
  
> UniRef90\_A0A071ICK8\_25\_398 | Acyl-CoA dehydrogenase n=10 Tax=Rhizobium/Agrobacterium group TaxID=227290 RepID=A0A071ICK8\_AGRR | E\_val=2e-72   
EISADFAKRAAELDREGLPPLKEIIRLKNSGLLNALHAPQIGGGGLNWVDALKLVRIISRGESSLGQLLGYHFVNSQYIYWALDDAARAHaLGAEtVAKN  
YYWGAAVNPRDPGLELTRRGSAYVLNGRKSFSTAAHVSDYINANAALDGKVASFAVPTNRPGYVANDDWDNIGQRLSDSGTVEFRDFPVYEEDFIAPPSE  
PDAPPPVLSTFNTPLIQLVFVNFYLGTAEGALEAALDYVRTTTRPWVTSGVEKASDDPYILERVGEFTAALKASAALADSAAEAVQAALSRGKDVTARER  
GEAAAEAYAAKVHATNVSLDITSRVFELTGARSTASSYRFDRFWRNVRTHTLHDPVFYKAKEVGEFVLNGKIPT
  
> UniRef90\_A0A2L0WNE5\_33\_396 | Sulfur acquisition oxidoreductase SfnB family protein n=2 Tax=Rhizobium TaxID=379 RepID=A0A2L0WN | E\_val=3.7e-72   
RAAALDREGRPPLAEIIKLKKAGLLNALHSREIGGGGLDWVDGLRLVRILARGESSIGQLLGYHFVNSQYIYWALDPVRANALGEEtVAENLYWGAAVNP  
RDPGLVLTRRGNGYVLNGRKTFSTAAHVSDYINANAALDDKIVGFTIRTNRPGYIANDDWDNFGQRLSDSGSVDFRDFPVYTEDFVGQPVAPDAAPPVLS  
TFNTPLIQLVFVNFYLGTAEGALQAALDYVRATTRPWVTSGVERAADDPYILERVGEFTASLKAATALADSAAAAVQTELAKGHSVTARERGAAAAEAYA  
AKVHATNISLDITSRVFELTGARSTADKYRFDRFWRNVRTHTLHDPVFYKAKEVGEFVLNDKIP
  
> UniRef90\_A0A1Q8CFJ2\_9\_380 | Acyl-CoA dehydrogenase n=1 Tax=Actinophytocola xinjiangensis TaxID=485602 RepID=A0A1Q8CFJ2\_9PSEU | E\_val=1.1e-71   
ADQLARTLRADAPHREQAAAEPEAEVALLRASGLLTLLLPASAGGRGAGWLAANETARTVAAADASVGHLLGYHYLQLWRTELFGRPDLVERLHRDtVAG  
NLFWAGVSNPLDAALELTPVEGGFRVEGRKSFATGASVADRLVVSATRVDSGQKLTFVVDAHAPGLSYLHDWDNLGQRLTASGGVVFRDVHVPDEDVLGV  
HDETSVRASLAALGFQLMLAQIYVGLAEGALTEAAEYTRTKTRAWTLSDVDEAVADPYILVGYGELVAQTRAAGALTDRAAAALGAAFDRGDALSEQERG  
EAAVTISAAKVVATRVANEAGSKIYEFTGARASANKYGLDRFWRNARTLTLHDPVAYKAREVGAHFLTGEPP
  
> UniRef90\_UPI000835F605\_21\_394 | monooxygenase n=1 Tax=Labrys sp. WJW TaxID=1737983 RepID=UPI000835F605 | E\_val=2.3e-71   
SAAAELAATALERDRANQDPFEEIELLRKAGLLGLAVPRALGGAGANLAQALEISRIISAADGSIGQLIAYHYSNGVWSYILGTPEQWQATARGVGEEGW  
FQGGVSNPRDPKAELEKTEKGYLISGRRTFATGASIAQILTVTVWDGEKRVHFQVPPSRKGISFEGDWDNLGQRLTASGSVIFDRVEATDADLLSGLDHY  
AGDVEQRDGLRVLFSQLIFVNFYLGIAEGALKAASAYVRSHGRPWPESGLEKATEDPYHLQLFGRLSAGIAAGVALADKAAALYQAALLAGPALTAQAWG  
ELALLIDQAKVIATKVSLEVTADIYEATGARSTANKYGLDIYWRNVRTHTVHDPVSYRLREIGEFALNQTLPKP
  
> UniRef90\_UPI000374AB86\_31\_398 | acyl-CoA dehydrogenase n=1 Tax=Rhizobium sp. 2MFCol3.1 TaxID=1246459 RepID=UPI000374AB86 | E\_val=6.1e-71   
ALRAATLDREGRPPLNEIVKLKNAGLLNALHAPALGGGGLDWVDGLKLVRILARGESSIGQLLGYHFVNSQYIDWALDDRARAHaLSAETVANNLYWGAA  
VNPRDPGLMLTRRGNGYVLNGKKSFSTAARISDYINANATLAGRIASFAVPTNRPGYVANDDWDNIGQRLSDSGSVEFHDFPVYNEDFIGAPVDEGAEPS  
VLASFNTPLIQLVFVNFYIGTSEGALHQALDYVRKTTRPWITSGVARAEEDPYILERVGEFTASLRASAALADTAALTVQNALARGSSVTARERGEAATE  
AYAAKVHATNVSLDITSRIFELTGARSTAESYRFDRFWRNVRTHTLHDPVFYKAREVGEFVLSDKIPA
  
> UniRef90\_A0A267RVL6\_22\_399 | Monooxygenase n=5 Tax=Enterobacteriaceae TaxID=543 RepID=A0A267RVL6\_9ENTR | E\_val=1e-70   
VAQEVADRLYATLLERDRANKHPFEEIDWLRQSGLLKLAVPKSLGGGGANLVQALEIGRIISAADGSIGQLITYHYSNGVWSYILGNREQWEFIARGVAN  
EGWFQSSISNPRDPRLkLEWDGNDLYVTGRRTFATGAAVSQVMTIAVWIEDRLVQYQVTADRPGIVFNDDWDNLGQRLTASGTLEFNRVRLqEKDRLTGL  
EEKPESALLRNALRNQFSQLIFVHFYLGIAEGALKQAAAYFRDTVRPWPESGVESALEDPYHRLKAGELSSDLAAGIALAEKTAHAFQDAFHAGDDLTET  
QWGELALLTDQAKVIANNVALKISNEIFELTGGRSTSNQYGLDVFWRNVRTHTLHDPVTYRTREVGDYVLSGKLPTPR
  
> UniRef90\_M3VA40\_23\_395 | Putative acyl-CoA dehydrogenase n=1 Tax=Gordonia malaquae NBRC 108250 TaxID=1223542 RepID=M3VA40\_9AC | E\_val=1.4e-70   
DLVAAELRATAAERDRANAAPTAEIELLRSHDLLQVGEPVELGGSGLNYAQTQQITRRIARGDTSIAHLLGYHYAQQRIAHLFGTPEQAEALSRRNAQEK  
LFWGGVQnPRGGSGLVLTRDEDGFRLNGRRTFASGAGVGDQLSVTATFGDALVFLSLPAGKEGFTPLGDWDNIGQRLTDSGGVEFVNTPISRDEILGDPD  
SPPTFTAFQQLVTPHWQLAFVNFYIGTAEGALDEALDWTRLNASAWETSGLEAATDDPYILELVGELRAEITAAVLLADRAGDALQHALDIGEALTDEQR  
AVAAVAIAEAKYVTTKVSLDAASRLFEIQGARATTTDYGFDRHWRNLRTHTVHDPVAYKAREVGDWTLNHRAP
  
> UniRef90\_A0A395GID9\_32\_425 | Thermophilic desulfurizing enzyme family protein n=3 Tax=Aspergillus TaxID=5052 RepID=A0A395GID9 | E\_val=2.9e-70   
LARAREVAQVLGQDAAQRDQENKSPRAEVVLLKHSGLLKLLGPKKYGGGEQPWDIGYKAIREVAKGDGSIGMLLGYHLLWSTTANIVGTPEQAERIQEWI  
ISNNYFVGGAVNPRDSDLKITSDGeDIVFNGAKFFNTGGVVSDLTVLEGVLDNTGEHIFALVETRQPGIQFAHNWHNIGLRLTESGGVKIENVRVPWADA  
LGWDTASKTPrddVLKVPFASLLLPTIQLVFSNFYLGIAQGALDFASKYTVTSTRAWPFGGDNkdSPTEEFYILERYGNFFAHLRAAEALADRAGNELAT  
LYsqhsDNRPGLTSQQRGEVAEWIASVKVVTTDVGLRVTSGVFEVTGARATSLKVGLDRFWRDIRTHTLHDPVAYKNRELGRYALLGEVPEPSW
  
> UniRef90\_UPI0008268C2F\_35\_406 | acyl-CoA dehydrogenase n=1 Tax=Gordonia hydrophobica TaxID=40516 RepID=UPI0008268C2F | E\_val=5e-70   
VAAQLRATAAERDRANKAPLAEVALLREADLLQVGEPVAYGGSGLDYAQSQQITRRIARGDTSIAHLLGYHYAQQRIPHLFGTPEQADQLSRRNAEFRPF  
WGGVQNPRGAAGLTLTRDGaGFRLNGARTFASGASISDQLSVTAALDGDLVFLSIPTRRDGFTPQGDWDNIGQRLTDSGGVVFDNTPVERSEVLGDDPLT  
GRIPSAYQTLVTPHWQLAFVNFYIGTAEGALEEALDWTRLNASPWETSGLDQATDDPYILELVGQLRSEISAAALLADRAGDALQHALNIGPELTAHQRA  
EAAIAIAEAKYVTTKVSLEVTSRLFEIQGARATTSAYGFDRHWRNLRTHTVHDPVAYKAREVGDWTLNRRAP
  
> UniRef90\_A0A0S1XV73\_27\_397 | Monooxygenase n=2 Tax=Alcaligenaceae TaxID=506 RepID=A0A0S1XV73\_9BORD | E\_val=1e-69   
AVALRPLLQADAVQRDKAGGRPVAQIALLKQQKLNAAAIAPQYGGEGASWVSILRVVRELARSDGSLAHLYGYQHLPLHTVLARGTDEQQQRWFSQAATE  
QWLWSNSGNAMSKTSAAERVPGGWIVDGFRPFSSGSHVADVIHIAWQDSTGARWSALIPADRAGVVIEDDWDGIGQTQTGSGTVSFHGVRVRDDEVLGDA  
GSPLSPFESVVSLLQQSVLANVFIGSAVGALDEAREYTVTQSRPWIHSGVERHVDDPWIQRAYGDLAIRTLAAIELADDAAAHLDRACALGRGLDAETRG  
RVSIDLAAANVYAGENALDVTEKIFELMGARSATRARGYDRFWRNVRTHTLHNPAEYKKRTIGAWLLRHPY
  
> UniRef90\_A0A0F5N116\_19\_398 | Acyl-CoA dehydrogenase n=3 Tax=Mycobacteriaceae TaxID=1762 RepID=A0A0F5N116\_9MYCO | E\_val=2.3e-69   
LATADRVADELRQTAAARDKANAAPRAEIELLRRNDLLHVQEPVEYGGSGLSYPQASQVTRRIARGDTSIAHLVGYHYAQTRIAGLFGTPEQADALSRRN  
ASEKlFWGGIQNPRGGSDLVLTRDGdGFRLNGSRTFASGASTGDQLSVTASLDGSLVFLSLDVRGGRQGFTFLDDWDNIGQRLTDSGGVRIVDARIEHRE  
VLGEEPFGSADLTPYQTLVTPHWQLAFVNFYVGTAEGALAEAFDWTRAYASPWESSGVERATDDPYILQTVGELVSEVRAAALLADRAGDALQAAIDVGP  
SLTEDQRAEAAIAIYEAKYLSTKVALDTASRLFEIQGARATTSKYGFDRHWRNLRTHTVHDPVAYKAKEVGDWVLNGQRP
  
> UniRef90\_A0A1N6Z7J9\_40\_406 | Acyl-CoA dehydrogenase n=1 Tax=Rhizobium sp. RU20A TaxID=1907412 RepID=A0A1N6Z7J9\_9RHIZ | E\_val=4.3e-69   
AKLDAEGRPPLEDITTLKAAGLLTALHPTEIGGGGLDWIDGLRLVRILARGESSIGQLLGYHYVNSQYPYWAVSESQKAHdLGVETVQKSLYWGAAVNPR  
DPGLTLTKRGDQYLLNGRKTFSTGAHVSDRINANAAYRNQVANLVLPTDRAGYIAYDDWDNIGQRLSDSGSVEFKDYVVYESDFLFPLVEKGAAPTVLAT  
FNTPLIQLVFVNFYIGTAEGALSAATDYVRTTTRPWVTSGVATASEDPYILERIGEFRATLKASAALADAAAHAVQAALSRGRNVTERERGEAAIEAYTA  
KVNATHVSLDVTSRVFELMGARATASHYRFDRYWRNVRTHTLHDPVFYKAREVGDFALNGRVPEPSL
  
> UniRef90\_A0A2V4UWQ2\_31\_411 | Alkylation response protein AidB-like acyl-CoA dehydrogenase n=8 Tax=Rhizobiales TaxID=356 RepID | E\_val=8.7e-69   
ARATAIAADLRARGAALDRDGRPPFSEITELKAAGLLNALHPPEIGGGGLDWVDGLRLVRILARGESSIGQLLGYHFVNSQYISWAADDsDLAWRLSSEt  
VAKSLYWGAAVNPRDPGLVLTRQDDHYVLNGRKTFSTGARISDRINASAAFGDQIASLVLPTDRPGYIAHDDWDNIGQRLSDSGSVEFKDFPVHDSDFLL  
PLAAPDAAPKVQATFNTPLIQLVFANFYIGTAEGALAEALDYVRDTTRPWITSGVERAADDPHILERVGEFRAALKAAAALADSAGLAVQTALARGPGVT  
ERERGEAAIEAYESKINATHVSLDLTARVFELMGARATAGHYRFDRYWRNVRTHTLHDPVFYKAREVGDFALNDRVPTPSL
  
> UniRef90\_A0A4P8KLT1\_5\_401 | Acyl-CoA dehydrogenase n=1 Tax=Microbacterium sp. RG1 TaxID=2489212 RepID=A0A4P8KLT1\_9MICO | E\_val=1.4e-68   
TLTPARTHFQgTADAAELAhWDAVAASVAERLALDALARDRANAQPFAEARLLKDAGLTTLLDPAEYGGGGAHWESAFRAVRILARADASIAQVLGYHYV  
NEANIALVAPAAERERWFRATIAGRWVWGDSVNPVDPNLTLTVDGdGYRLNGFKRYSTGSGVGEALLINAVVEDGPDAGAFAFLVLPYGHPGVELVDDWD  
NLGQRLSASNTVTYRDVRVEPEHVLGFGTDEPIQSFVTPAIQLVFGNLYLGIAQGALAQARELTNARPNSWFLSGVQTYSDDPFVRRLYGELVSRTAAVE  
ALADRVNSRFDEVISRGADVTAEDRAEIAIEIAQLKVVASDTATDVAHRVFEATGTSSTANRVGLDLHWRNIRTHSLHDPVDYKKLEVGAHFLTGAV
  
> UniRef90\_UPI0003068598\_3\_292 | acyl-CoA dehydrogenase n=1 Tax=Burkholderia pseudomallei TaxID=28450 RepID=UPI0003068598 | E\_val=2.1e-68   
GSAAQRERYLRGTVEHDWWWGNAVNPLDTRLVARATGDGGYRLDGVKGFCSGTRGSQRMTVSAHDSVTGKPVFAVVPTQREGIAVRDDWDPIGQRQTDSG  
SVAFDGVRVAPDEVLHRSEAPPTPRATLRALVSQLVLTNLFVGIAEGALAEARDYVQRAGRPWLHSGVERAADDPYTLQRFGDMRVQTVSAEALADRAAR  
ALQGAWAKHEALTADARAEVALAISEAKIVAQRAALDVSEALFDACGARATAAPLALDRFWRNARTHTLHDPLDYRLRDVGRYALTGALP
  
> UniRef90\_UPI000DD585C3\_27\_403 | acyl-CoA dehydrogenase n=2 Tax=Rhizobiales bacterium TaxID=1909294 RepID=UPI000DD585C3 | E\_val=2.5e-68   
QASEVSAKFARRGPELDRLGLPPRDEIEALRQAGLLSALHAPEVGGAGLSWVDGLRLVRIVARGESSIGQLLGYHYVNSQYVYWAAADPRQAWVFgAETV  
ERQLYWGAAVNPRDpGLVLTRRGGHYVLNGRKTFSTGAHVSDRINVNATLDDKVANFVVPTDRPGYVAHDDWDNIGQRLSDSGSVEFQDFPVyERDFILP  
LAAPSDPPPVQATFNTPLIQLVFVNFYLGTAEGALAAALNYVSTTTRPWITSGVTRAGDDPYILERIGELQAALKASVALADVAAGIVETALAKGRSVTE  
RERGEAALEAYAAKVSATHVALDVTAKVFELMGARATASHYGFDRYWRNVRTHTLHDPVSYKAREVGDFALNGRIPS
  
> UniRef90\_A0A1E3SMN8\_25\_404 | Acyl-CoA dehydrogenase n=1 Tax=Mycobacterium intermedium TaxID=28445 RepID=A0A1E3SMN8\_9MYCO | E\_val=4.3e-68   
LATADRVADELRRTAAERDKANATPRAEIELLRHNDLLQVQEPAEYGGSGLNFAQAVQIVRRIARGDTSIAHLIGYHYAQTRVAGLFGTAAQADAQSRRN  
AsEKLFWGGIQNPRGGSALVLTRDGdGFRLNGYRSFASGASTGDQLSVTAALDGELVFLSLDVRGGRQGFTFLDDWDNIGQRLTDSGGVRVVDARIEHHE  
VLGEEPFIGNTLTPHQTLITPHWQLAFVNFYVGTAEGALAEALEWTRAHASPWESSGVERATDDPYILHTVGELVSEVRAAALLADRAGDALQAAVDVGP  
SLTEDQRAEAAIAVYEAKYLSTKVSLETASRLFEIQGARATTSAYGFDRHWRNLRTHTLHDPVAYKAREVGDWVLNHRHP
  
> UniRef90\_UPI00045EBD18\_12\_396 | acyl-CoA dehydrogenase n=2 Tax=Xanthobacter TaxID=279 RepID=UPI00045EBD18 | E\_val=8.8e-68   
SLAPAAPARPDWIARAQSVADALTPDAAERDRIGASPRRELALLRDAGLLELLFSPEHGGGGGSFTDALLAVRVIGRADASIAQLLAYHYLHLTNALWRA  
RPEQAAALARASVAGRWLWGGASNPRDPESRLAPVEDFYRLTGRKTFASNAALADRITLRAQLGDGFAVLVVPGDREGVTHGNDWDAFGQRLSESGTITF  
ADVKVEADEILGDPAAPPPPRTSLVVPFHQLLIVNFYIGIAKGALAEANRYIREVSRPWQASGVEKARLDPYILEHYGNLDVELRASTALADAAGVRFEA  
ATALGNAITPDDRNVAAAAIYAAKVHSSRTALEITSRIFELMGARATAGHYGYDRFWRNIRTHTLHDPVVYKSREVGNYALNGVI
  
> UniRef90\_A0A1Y1ZNX6\_42\_434 | Acyl-CoA dehydrogenase/oxidase n=1 Tax=Clohesyomyces aquaticus TaxID=1231657 RepID=A0A1Y1ZNX6\_9P | E\_val=1.2e-67   
AQEVADVLAVDAVLRDKENKSPRAEVELLKHSGLLKLLGPKKYGGGEQPWSVGYKAIRKVAEADGSIGMLLGYHLLWSITANVVGSPEQADRYQKLIIEN  
NYFIGGVVNPRDNDLKITSEGDNIVFNGFKHFNTGGVISDLTVLEGVLDGTTDHIFAFAPTNQDGIKFSHDWNNVGLRLTESGSVKLENIVVPWSDALGW  
DTTQrkpDPAILGIPFGSLLLPTIQLVFSNFYLGIALGAQDYASKYTTKNTRPWPFGGDnkERATDEFYILSTYGNFHAHLRAAVALANQAGAEVDKIYS  
SyaGEAgirakLTARERGELAEWVASTKVVTTDTGLRVTAGVFEVTGAKATSAKAGLDRFWRDIRTHSLHDPVAYKNRELGRFQLLDEVPEPT
  
> UniRef90\_A0A0D2FPI6\_31\_423 | Unplaced genomic scaffold supercont1.20, whole genome shotgun sequence n=2 Tax=Cladophialophora | E\_val=1.7e-67   
WIARAQDVGQILAADAPKRDIENKSPFSEIQLLKASGLTKLLGPKNYGGAGQDWAIAYKAIREVAKGDGSIGMLLGYHLLWSTTANVVGTDEQKHRIQKL  
ILDNDWFVGGAVNPRNADLKITSDGeEIVFNGFKSFNTGGVVSDATILEGVLDGTEDHIFTIVPTKQPGIQFGHDWDNIGMRLTESGSVKIENVRVPWTD  
AFGWDSKTkrpIAEVLKVPFASLLLPTIQLVFSNFYVGIGLGALEEAKKWTTSKTRAWPYGGDNkaKATDEHYILARYGNFHAHLRAAEALADLAGEKIR  
DVYaDHGEKrdVSARRRGEVAEWVASIKIVATETSLRVTSGVFEVTGASSTARKVGLDRFWRDVRTHTLHDPVAYKERELGTFYLLDEVPEPT
  
> UniRef90\_A0A2S0KGS2\_30\_403 | Acyl-CoA dehydrogenase n=1 Tax=Gordonia iterans TaxID=1004901 RepID=A0A2S0KGS2\_9ACTN | E\_val=2.9e-67   
DLVAAELRASAAERDRANAAPVAEVELLRTHDLLQVGEPVELGGDGLNYAQSQQLTRRIARGDTSVAHLLGYHYAQQRIPHLFGTREQAEALSRRNASEK  
LFWGGVQNPRGGSALELTRDGDGFRLNGRRTFASGASIADQLSVTAVFEGDLVFLSLPAGVEGFTPLGDWDNIGQRLTDSGGVEFDDTPVARDQILGSDP  
LTGKELSAYQQLVTPHWQLAFANFYIGTAEGALDEALDWTRLHASAWETSGLEAATDDPYILELVGELRSQIAAAALLADRAGDVLQQALDFGPELSAEQ  
RAEAAIAIAEAKYLTTKVSLEAASRLFEIQGARATTSAYGFDRHWRNLRTHTVHDPVAYKAREIGDWTLNHRAP
  
> UniRef90\_A0A1A7MGQ0\_54\_442 | GABA permease n=42 Tax=Aureobasidium pullulans TaxID=5580 RepID=A0A1A7MGQ0\_AURPU | E\_val=3.7e-67   
ARDVASILATNAAARDIDNKSPFAEISLLKSSGLLKVLGPTEYGGGGQEWEIGYKVIREVAKGDGSIGMLLGYHLLWSKTADIVGTDEQKERFQKLIIEN  
NYFVGGAVNPRDNDLAISDHGDHLVFSGSKHFNTGGVISDLTVLEGVLSGTSNHIFAIAPTSQPGFEFAHNWNNIGLRLTESGSVKINDIKVPWSDALGw  
dATSKKPLDSvLSIPFATLLLPTIQLVFANFYLGIAQGSLEFARGYTTTSKRAWPFGGDnkDSATEEFYILERYGNFHAHLLAAEAltdrASKEISDIFL  
THGGKRDVSVRQRGEVAEWVASAKVVTTDTSLRVTVGVFEVTGSRATGRKVGLDRFWRDVRTHTLHDPVAYKNRELGRYFLLDEVPEPT
  
> UniRef90\_UPI000413001D\_18\_392 | acyl-CoA dehydrogenase n=1 Tax=Azorhizobium doebereinerae TaxID=281091 RepID=UPI000413001D | E\_val=7.4e-67   
AAAGQVAEALRADAAARDHAGLAPRRELELLRASGLLALLNPPAHGGGGGSFGDAFRAVRRIARVDTSVAQLLSYHYLHLINALWRAGAGQGEQLSRASV  
SGRWFWGGASNPRDPESQLTADGpDFRLNGRKTFASNASLADRITLRAQIGGGFAVLTVPGDRAGVTHGNDWDAFGQRLTESGTIAFDNVRIGRGEILGE  
AEPGSAAPLSPRVSLVVPLHQLLIVNFYVGTAEGALAEANAYLRSTARPWQASGVSEARQDPYVLEHYGELDVQARAAAALADVAGAALEGAIARGHDLT  
VPERHAAAAAIYAAKVQSSRAALDITSRIFELMGARATATSYGFDRFWRNIRTHTLHDPVFYKAREVGNYALNGT
  
> UniRef90\_G7H5P9\_11\_400 | Putative FMNH2-dependent monooxygenase n=3 Tax=Terrabacteria group TaxID=1783272 RepID=G7H5P9\_9ACTN | E\_val=1.3e-66   
NVADQQGRFDDALRRADLVAAELRSTAAERDRANADPVAEVDLLRQADLLQVGEPVEFGGSGLNYAQSQQLTRRIARGDTSIAHLLGYHYAQQRIPHLFG  
TPEQAQEISRRNAEEKTFWGGVQNPRGGSALELTRDGDGFRLNGRRTFASGASVADWLSVTAVFEGDLVFLALPRDVEGFRPLGDWDNIGQRLTDSGGVE  
FVNTPVSRDQILGADPITGKEFSAYQTLVTPHWQLAFVNFYIGTAEGALEEALDWTKLNASAWETSGLETAVDDPYILELVGELKSQVTAAALLADRAGD  
ALQESLDFGPELTAEQRAATAVAIAEAKYLTTKVSLEVASRLFEIQGARATTSAYGFDRHWRNLRTHTVHDPVAYKAREVGDWTLNRRAP
  
> UniRef90\_A0A010YHQ5\_11\_404 | Acyl-CoA dehydrogenase n=1 Tax=Cryptosporangium arvum DSM 44712 TaxID=927661 RepID=A0A010YHQ5\_9A | E\_val=1.8e-66   
TDRPDPAEHARWVAVADRVAAQLSSDVVARDRSGAAPHKEVELLRDAGLLPLLIPARNGGHGGSWLTAFEVVTRVGRADTSIGHVLGYHYLHNWRTRLAR  
RLDVVERLATETAANNWLWGGAGNPRDAGLELEPTSGGYLVRGKKFFATGAEVSDRVIASGTDTVTGQKYGFALPTRTAGVVHGEDWDSLGQRASASGSI  
AFDGAFLAEEDILGPGEQTDPDAPAYPSLSALGFQILLGLLAVATAEGALRFAAEYTRTKSRAWATSGVAEAADDPLVRVRYGELESQVRAARALTDRAG  
AAWMAAASRGWELTHAERGEVSVELSAVKVVTTRAALDATQGVFELTGARATKTGTGLDRYWRDVRTLTLHDPVSHKAIEVGDHLLRGAYPEPS
  
> UniRef90\_B0RBU7\_24\_404 | Putative dehydrogenase n=1 Tax=Clavibacter michiganensis subsp. sepedonicus (strain ATCC 33113 / DSM | E\_val=2.9e-66   
ELAHWRGIAEHVAATlaedALARDRAGLDPTAELDLLRDSGLVNLLDPAEHGGGGGHWESAVLAIRVLARADASIAQVLAYHYINSGNLGFTATGDVRAD  
GYRRTIAGRWVWGDSVNPTDPDLRLTPDGDGYQLDGLKRFSTGASAGDVILVNAVVAGGELDGRIVVFALDHDRPGIAYLGDWDALGQRLSASGSVRFTD  
VRVEPDDVLGVGSDEPFSTLVTPAIQLAFGNLYLGIAEGALAQALDLVRARRGAWFLSGVDAYRDDPFVQRVVGELASRIAAVEALADRVGRAFDGVVDL  
GDGVTAEIRGRIAIDVAKLKVVATEVGVEVANRVFEVTGSSSARSSTGLDLFWRNVRTHSLHDPVDYKKLEVGAHALTGEL
  
> UniRef90\_A0A167RW81\_42\_429 | Acyl-CoA dehydrogenase domain-containing protein n=1 Tax=Sporothrix insectorum RCEF 264 TaxID=10 | E\_val=5.7e-66   
VLARDAGVRERENKSPRAEVALLKHAGLLTVLGPTRYGGGGQPWRVGYKVVREVAKADGSLGMLLGYHLTWSTTANIVGSPEQADRFQKLIVTNNYFVGG  
AVNPRDSDLRITLDDAAGGDHlVYNGAKHFNTGGVVSDLTVLEGVLAGTNDHIFAIVPTQQPGIQFAHNWDNIGLRLTESGSVRIENVHVPWADALGWnP  
QTKqpDPAILSIPFASLLLPTIQLVFGNLYLGIAQGSLDFASAYTTKHTRAWPFGGEnkEKGKDEFYVLSAYGNFAAHLRAAEALTDKAGDELSALYAKH  
AAdragLTTAARGDVAEWIASAKVVTTDTGLRVTAGVFEVTGSRATATAVGLDRFWRDIRTHTLHDPVAYKNHELGRFQLLGAYPEPT
  
> UniRef90\_A0A1X0J8A0\_26\_405 | Monooxygenase n=3 Tax=Mycobacteroides saopaulense TaxID=1578165 RepID=A0A1X0J8A0\_9MYCO | E\_val=1.5e-65   
TAQRVADQLAATALDRDRANQNPSGEIGLLREYGLLSFATAREFGGAGGSLTQALQLSRIIAAADGSIGQVLLYHYSNGVWTHILGSPSQREHIARGVGE  
LGWFQGSVSNPRDPGIRVTRTDEGYRVDGKRTFATGVALADLITVLLYEDEPINAVIPRDREGLRFNDDWDNLGQRLTASGSVEFDSVLLRHDEVLtGLA  
ELSASDGSRERRDGLRALFSQLIFVHLYLGIAEGALAAGVTYIREQGRPWPEAHSTQVTEDPYHQQLLGRLSAGIAAGIALADTVTREFEQALGAGETPN  
AAQWGALAIRVDQAKSIATEVSLDVTHNIYQATGARSTANSVGLDIYWRNARTHTTHDPLPYRQREIGRYLLTDEWPLPR
  
> UniRef90\_A0A2J6Q859\_43\_430 | Thermophilic desulfurizing enzyme family protein n=1 Tax=Pezoloma ericae TaxID=1745343 RepID=A0A | E\_val=2.2e-65   
EVAEILAVDVVQRDRENKSPRAEIVLLKHAGLTKVLGPKKYGGGGQPWALAYKLIREVAKVDGSIGMLLGYHLLWTTTANVVGTPEQADRWQEKILSNNY  
FVGGAVNPRNSDLKITSDGeDIVFNGTKFFNTGGVVSDVTVLEGVLDGTQDHIFTIVKTQQPGIQFAHDWDNVGLRLTESGGVKLENVRASWKDAFGWdP  
SSKkpIPEILTIPFATLLLPTIQLVFSNFYLGIALGGLDFASKYTAKSTRAWPFGGDnkDKASEEFYILATYGNFYAHLRAARALADRAGDRISAVYaah  
sTDRSSLTARARGELAEEVASVKVVTTDTGLRVTSGIFEATGATSTKSKFGLDRFWRDIRTHTLHDPVAYKNRELGRFQLLNEVPEPT
  
> UniRef90\_UPI0003828F02\_7\_389 | hypothetical protein n=1 Tax=Actinokineospora enzanensis TaxID=155975 RepID=UPI0003828F02 | E\_val=5.4e-65   
VAVAREVAARLVADAVARERETAEPVAEVALVRDSGLLPLMIPADHGGHGEDWATAHAVLAVVAAADASVGHLLGYHYLHVWRTSLFDVPATAAALDRAT  
VEHGWfWAGVANPRDDALTVSDVDGGFAVAGRKFFATGASVADRLVVSGQHATTGRKLTFTLDARAPGIRYLGDWDNLGQRRSASGGVEFANVALAPDAV  
LGWTPEPDDPRAQRDSLAALGFQLVLSRVLAAIGRGAITEAARYTRETSRAWPASGVEHAVDDPHILAGYGSLISRLDAADLLVDAAADSFTAAAARGDG  
LTATERGETSLRISAAKTVTSELATEIASRVFEFTGARATAAKHGMDRFWRNARTLTLHDPAVYKAAEVGRHLLTGEYPTPGG
  
> UniRef90\_A0A1B2HDJ6\_6\_361 | Acyl-CoA dehydrogenase n=2 Tax=Actinobacteria TaxID=201174 RepID=A0A1B2HDJ6\_9PSEU | E\_val=8.6e-65   
DVARDVAATLRSDAADRDRANQPPTKEVELLRQSGLLNVSDWATQQRINRIIGAADANAGHLLGYHYLQIWRSGLFDTAFSRHDDQFWAGVSNPLDAALE  
LTPTGTGFLLNGRKTFATGASVADRLVVSATRTDNGEKLTFLLDGKATGITCLDDWDNIGQRLTASGGVVFENVEVTEVLGVQPANEDPRISLAAIGFQL  
VLAQLYVAIAEGALDEAADYTRTKTRPWFVSGVEHATEDPYIVAAYGEMVSQTKAAGLLADEAAKLLWEASELGKNLTPQKRAQVAVEISAAKVVSTKLV  
NEVSSRIFEQVGARGTAQKYGVDRFWRNARTLTLHDPVVYKAREVGEHFLTGARPA
  
> UniRef90\_A0A2N3N0U1\_47\_427 | Uncharacterized protein n=1 Tax=Lomentospora prolificans TaxID=41688 RepID=A0A2N3N0U1\_9PEZI | E\_val=1.5e-64   
VDAAQREKENKTPRAEVALLKYAGLLKVLGPKKYGGGEQPLSVGYKVIREVAKADGSLGMLLGYHLLWSLTASIVGTTEQAERWQEIIITNNYFVGGAVN  
PRDGDLKITEDGdNLVFNGFKNFSTGGVISDLTVLEGVLEGTENHIFAFVKSDQPGVQFQRNWNNIGLRLTESGSVKIENVTAPWSDALGWDvqkKEPDL  
AYLGIPFATLLLPTIQLVFSNFYLGIAQGALATASKYTVATTRAWPFGGenKEKGTDEFYVLSTYGNFHAHLRAAEALADQAGREIDAVYAKGtpDrsKV  
EARDRGELAETVASLKVVTTDTGLRVTSGVFEVTGARTTSAKYGLDRFWRDLRTHTLHDPVAYKNKELGRFQLLNEVPEPT
  
> UniRef90\_S5Y4X6\_32\_408 | Acyl-CoA dehydrogenase n=1 Tax=Paracoccus aminophilus JCM 7686 TaxID=1367847 RepID=S5Y4X6\_PARAH | E\_val=3.4e-64   
ARAIAAELAATISARDRANLDPHAEIELLRQAGLLGLAAPRDFGGGGASLLQAMEIVRLISAGDGSIGQLIAYHYSNGVWTYILGTPAQWAETTRHVAER  
GWFQGGVSNPRDQWSEIETEGTRRFISGKRSFATGTAISQIITVSLWDKGRRVHYQIPTDRAGISFGNDWDNLGQRLTASGSVTFDRVELFEHERLSALD  
HWPGESAERDGLRGLFSQIIFAQFYLGIAEGALEAAETYIREEGRPWPESGLSSAVEDPYNAVILGRLSAQVEAGIALADRATLAFQEALFAGPDLARDD  
WGRLAVLVDQAKVVANDVSLEVTARIYELTGGRSTANRFGLDHFWRNIRTHTTHDPVSYRAREIGLARISGTLPVPR
  
> UniRef90\_A0A3R2WQN2\_17\_411 | Dibenzothiophene desulfurization enzyme C n=3 Tax=compost metagenome TaxID=702656 RepID=A0A3R2WQ | E\_val=4.5e-64   
KPRPADAPQWLARAAEVAAILAVDQVARDRQQAVPTAEVQLLKDAGLTTLLGPVQHGGAGQPWDTAYKVIRIVASGDGSIGQLLGYHYLWAWAVHLVGTS  
EQIDAVEALYTANDLFFGGAVNPRDGDLVITEVDGQLAYNGRKSFSTGSKVSDLTVLEGVLQGTDKHIFAIVPSKQDGIRYLDDWDHLGQRLSESGGVII  
EDVRIDWASAAGYVDKVYQSRtYETLNLPAIQLVFANFYLGIAEGALKTAAGYTREKTRAWPYGGDnkESASDEWYILEAYGTLQSRLWAAEELANSAgA  
EISALLHAERDALTPGARGRVAVRIAAAKQAAVDIGLEIGTKVFEVTGARATANAVGLDIFWRNIRTHSLHDPIAYKRREVGAYALRGQIPEPSW
  
> UniRef90\_A0A3N1X255\_20\_392 | Alkylation response protein AidB-like acyl-CoA dehydrogenase n=1 Tax=Comamonas sp. BIGb0124 TaxI | E\_val=1.1e-63   
AAALREVLHADAVARDEAGGQPLDAVRALKASGLNAAWIGRAYGGEGASWTSILRVVREFARTDGSIAHLYGYQHLPLHITAARATPAQLANWFPASVRE  
NWLWSNSGNVMSRTSTARRVGTHWVLDGHRPFSSGTHVADVIQVSWDGDGGRYTAVIPADRDGVGIAHDWDGIGQRQTGSGTVLFQGVRIADEELLGRPG  
DEVTPFVSLNSLLQQLVLAQVFIGSAQGALAEGRDYTVRESRPWIHSGVERHTDDPWVQRRYGGLHIRVQAAQALAEQAAAQLDELYPQGHGLTAHARGS  
LAISAAAANVVAGEVGLAVAEEVFEVMGARSATRARGYDRFWRNVRTHTLHNPAEYKKRTLGQWLLDGSHPQP
  
> UniRef90\_A0A1S1LA29\_28\_404 | Monooxygenase n=3 Tax=Mycobacteroides franklinii TaxID=948102 RepID=A0A1S1LA29\_9MYCO | E\_val=1.6e-63   
ERVAGQLAATALTRDRANQNPVAEIGLLRHHGLLSFATAREFGGAGGSLAQALQLSRIIAAADGSIGQLLVYHYSNGVWTYILGSPAQREYIARGVGEHG  
WFQGSVSNPRDPGITVTRTEEGYRVNGKRTFATGVALADLITVLLYEAEPINAIIPGDRAGLRFNDDWDNLGQRLTASGSVEFDDVLLRHDEVLTGIAEY  
SdRDGSRERRDGLRALFSQLIFVHLYLGIAEGALAAGVAYVRDKGRPWPEAYSTEVTEDPYHQQLLGRLSGGIAAGIALADSVTREFEQTLATGEAPTEA  
GWGALAIRVDQAKSVATEISLDVTHNIYQATGARSTANSVGLDIYWRNARTHTTHDPLPYRQREIGRHLLTDEWPSP
  
> UniRef90\_A0A081GNL4\_19\_392 | Uncharacterized protein n=1 Tax=Cyanobium sp. CACIAM 14 TaxID=1496688 RepID=A0A081GNL4\_9CYAN | E\_val=4.2e-63   
DLCDHLTDDAAARDRLGGVPLGARQRIRQGGLLSLSIPVPWGGGGWSWSRLSSLVRRIARLDSSVAHLLSYHYLGLTIPVIFGEGDlAETHLRATAAGHL  
FWCNALNPLDRRSRLRRDGDGWRLEGSKSFCSGSVDSDVIPTTAILEDTGEVVIVILPTASAGVKVIDDWDNIGQRQTSSGTVQFEQVAVRSDQILFPVR  
QGATPFSTIRTLLAQLNLANLYLGLAEGALKEACERFHERHGPSGGLGDGTGAVLARDRFARLWVPLQAADAHYERSLVRLEHCWNLGRGLTPVERGDCA  
IAIAVTKVLASEVALAVGSALFDRVGARYTHAALGLDRYWRNARTLSLHDPLDVKLQEIGDHVLNGQHPRPDFY
  
> UniRef90\_A0A1I6U758\_27\_419 | Acyl-CoA dehydrogenase n=2 Tax=Saccharopolyspora flava TaxID=95161 RepID=A0A1I6U758\_9PSEU | E\_val=5.7e-63   
PTPSTPEEWIARARAVARVLAADAVQRDRAGTPPTAEVRLLKESGLVTLLGPAEHGGAEQHWTTACRVVREISAGDGSIGQLLGHHYVWAWAMRLVGTPE  
QiaeTDELYTTRAHFFGGVVNPRDRDLIVTDEGEtLLYRGKKSFSTGAKIADLLVVEGTLEPGDQSVFAVVPARQDAITFGDDWDNLGQRLTVSGSVEIT  
DLRVPWSAAAGYVDKRFRASAFSTLHVPALHLVFANLYLGIAQAALRAGVAYTRGHTRAWPYggEGKNSGTEEFYVLEGYGDLQSKLWAAETLTDTAgAS  
LAQLLHSDRDGVTREMRGRAAVLVSAAKQRTIDVGLEIANRIYELTGARATSNAVGLDLYWRNLRTHSLHDPVAYKRVEVGRYALLGDLPTPS
  
> UniRef90\_A0A0F5VZP9\_7\_388 | Monooxygenase n=21 Tax=Streptomyces TaxID=1883 RepID=A0A0F5VZP9\_9ACTN | E\_val=1.6e-62   
ADDTEALAVAAALAEEFRAGATARDAERRLPRAELDRLSASGLLAVTVPAEHGGADVGPQTLAETFRLLASADASLAQIPQSHFAYVNVIRRQGTEEQRK  
FFFAELLSGRRLGNAQSEAgTRHVQDIRTRLAPRPDGSYVLDGVKHYSTGALFADWIPVLARAEEDKLHVAYVPRDAPGLTVIDDWDGLGQRTTASGTVR  
LEGVEVPADRVLPHHLTFDGPQLHGTLAQLLHAAIDAGIAGGALAEAAEFVRTKSRPWFESGLDTAAEDPLLIQRFGELALQVRATEALLREAARAVEAA  
QADLSDDSAAEASIAVAAAKVRAAEAAVEVASALFEVSGTRSALNSLNLHRHWRDARTHTLHDPTRWKIQHIGRYVLNGTRP
  
> UniRef90\_A0A1H4GIM8\_20\_411 | Acyl-CoA dehydrogenase n=2 Tax=Leifsonia TaxID=110932 RepID=A0A1H4GIM8\_9MICO | E\_val=2.5e-62   
RPHDSAGWIRRAQEVADILSVDALERDRANATPYTEVQLLKESGLVTLLGPREHGGAGESWDTAYKVIRAVARGDGSIGQVLGYHYLWAWAARLVATEEQ  
IAAVEELYTTNNFVFGGAVNPRDSDLTIREDGDeLVYSGRKSFSTGGQISDLTVLEGVLEGAETHIFAIVPTAQEGIVFAGDWDSLGQRLTESGSVEIRD  
VRVPWTAAAGFVDREFQPLVYNTLNVPTIQLVFANFYLGIAQGALEAGSSYTRSTTRPWPYGGDnkQRAGEEWYLLEGYGELQSKLWADEALLDVAgAEI  
SALLHAPREGLSERRRGEVAVRIAAGKLRIVDDGLEVATKIYELAGARASASSVGLDIFWRNLRTHSLHDPIAYKKREVGEYVLLNQIPEPT
  
> UniRef90\_A0A0M8TTT1\_8\_388 | Monooxygenase n=1 Tax=Streptomyces sp. MMG1533 TaxID=1415546 RepID=A0A0M8TTT1\_9ACTN | E\_val=3.4e-62   
DDVEALTVARSLADEFRAGASQRDAERRLPRQELDRITASGLLAVTVPAEHGGADVRQETLAEIFRLLASADASLAQIPQSHFVYVNVIHRQGTPEQQEF  
FFGEVLAGRRFGNAQSEaGTKHVQDIRTRLAPRPDGSYTLTGVKHYSTGALFADWIPVLARAEDDNLHVAYVPRGAPGLTVIDDWDGMGQRTTASGTVRL  
EEVPVPADRVLPHHLTFRGPQLHGAVAQLLHAAIDAGIAGGALAAAAEFVRTKSRPWFESGAETAAEDPLLIQRFGELAIQVRASEALLRDAARAVDGAR  
ADLTDDSAAEASIAVAAAKAHAAQTAVEVGSALFEVSGTRSALDSLNLHRHWRDARTHTLHDPARWKVQHIGRYVLNGTRP
  
> UniRef90\_A0A1Y2MDA3\_48\_437 | Uncharacterized protein n=1 Tax=Epicoccum nigrum TaxID=105696 RepID=A0A1Y2MDA3\_EPING | E\_val=5.3e-62   
AQDVADVLAVDAAVRDQENKSPRAEIALLKHAGLLKILGPKKYGGGEQPWGVGYKAIRKVAEADGSIGMLLGYHLLWSTTANVVGNEEQAERYQKLIVEN  
NYFIGGAVNPRDNDLKITPEGDKLVFNGFKHFNTGGVISDLTVLEGDFNGTGDHIFAFVPTDQPGIQFGHDWKNIGLRLTESGSVKINDVTVPWTDALGW  
DAEQkkpDPKVLQNSFAALLLPTIQLVFSNFYLGIALGAQQFASKYTVKGTRAWPFGGdnKERATDEFYILERYGNFHAHLRAAVALTDSAGEQVSNIYa  
aysgslETRAKLTARERGELAEWVASAKVVTTDTGLRVTAGVFEVTGSKATSLRVGLDRFWRDIRTHTLHDPVAYKNRELGRFELLDEIP
  
> UniRef90\_UPI000562F0C5\_9\_388 | SfnB family sulfur acquisition oxidoreductase n=1 Tax=Streptomyces griseus TaxID=1911 RepID=U | E\_val=9.2e-62   
EAEALAVAAALADEFRPGACERDARRRLPRPELDRLSASGLLAVSVPAEHGGADVGAGTLAEIFRLLASADGSLAQIPQSHFVYVNVIRRQGAPEQQKFF  
FAELLAGRRLGNAQSEAgTRHVQDIRTRLIPRPDGSYVLSGVKHYATGALFADWIPVLARAEDDTLHVACVPRDAPGLTVIDDWDGLGQRTTASGTVRLD  
DVPVPADRVLPHHLTFHGPQLHGAVAQLLHAAIDAGIAGDALVQAVEFVRTKSRPWFESGVETAAEDPLLIQRFGELALQVRASEALLREAARAVDAARA  
DLTDDSAAEASIAVAAAKVRAARTAVEVASALFEVSGTRSALNSLNLHRHWRDARTHTLHDPVRWKIQHIGRYVLSGTRP
  
> UniRef90\_A0A506Y8Z1\_43\_442 | Monooxygenase n=1 Tax=Schumannella sp. 10F1B-5-1 TaxID=2590780 RepID=A0A506Y8Z1\_9MICO | E\_val=1.4e-61   
SARPSDAAGWIARAREVADILAADAVDRDRAGAPPRSEVALLKASGLVTLLGPTAHGGAGQSWETAYRVIREVARGDGSIGQLLGYHYLWAWAARLVATE  
EQIAAVEELATSSTAFYGGAVNPRDADLTVRDEGDTLVFSGRKSFSTGGVISDLTVLEGVIEGSDADggPGIHVFAIVPTAQEGIRFAHDWDNLGQRLTE  
SGSVEIRDVRVPWAAAAGFVDKTFQPLIYNTLNVPTIQLVFANFYLGIAQGTLERATAYTRENSRAWPYGGDDktRATEEWYILEGYGELQSKLWADEAL  
LDAvgAEISAVLHAPRGELTERRRGEIAVRIAAAKARIVDDGLEAATKVFELTGARASANTVGLDIHWRNLRTHSLHDPVPYKRREVGRFALLGEIPEPT  
  
> UniRef90\_A0A021VVW0\_28\_409 | Monooxygenase n=1 Tax=Actinotalea ferrariae CF5-4 TaxID=948458 RepID=A0A021VVW0\_9CELL | E\_val=2.5e-61   
ARAEEVAAILAVDAVERDRALRTPHDEVRLLKQAGLVTLLGPRAAGGAGQSWATAYQVIRRVAEGDGSIGQLLGYHYLWSQLPAFFGTPDQARDIIGEAT  
REAWFFGGAVNPRDADLVAVDQGDHLVVSGHKTFSTGSKVSDVTWLEAAVDGHEDHVFALVASDDPAITFHDGWDALGQRLTESGSVTVQDAVVPWTAAL  
GWVDKAFRPHVYNTLCLVAIQLVFTSFYLGIASGGLQTALEYTRARTRPWPYGGDdrERGVDEPYVLDTYGDLQSKLWAAEALAERAGEAITALHLDPAA  
LTPRQRGEVAVLVAAAKQRAIDVGLEIGTRVFEVTGARATASDVGLDRFWRNVRTHSLHDPVAYKRREVGRFALLDEVPEPT
  
> UniRef90\_E3QTS2\_46\_428 | Acyl-CoA dehydrogenase domain-containing protein n=2 Tax=Colletotrichum TaxID=5455 RepID=E3QTS2\_COLG | E\_val=3.5e-61   
RDAALRERENKSPRAEIALLKHSGLLKVLGWKKYGGGEQPWSVGYKVIRTVAKGDGSIGMLLGYHLLWSTTANVLGNPEQADRFQDLIISNNYFVGGAVN  
PRDSDHKITSDGDkIVFNGLKHFNTGGVISDLTVLEGVLEGTEDHIYAIVKTDQAGVRFLHNWDNVGLRLSESGSVIIENVTAPWADALGWDASKkkpDP  
SILSIPFATLLLPTIQLVFSNFYIGIAWGALNFASAYTKRNTRAWPFGGDnkEKAVDEFYILSTYGNFFAHLRAAEALADKAGLEIDKLYrSSGDPsera  
nVTAEARGEAAEWVASVKVTATDTGLRVTAGVFEVTGARATATKVGLDRFWRDIRTHSLHDPVAYKNRELGRYQLLGEIPEPT
  
> UniRef90\_A0A1D8SMV7\_7\_388 | SfnB family sulfur acquisition oxidoreductase n=2 Tax=Streptomyces olivaceus TaxID=47716 RepID=A | E\_val=5.3e-61   
ADDAEALAVATALADEFRPGASARDSERRLPRAELDRLSASGLLAVTVPADHGGADVRATTLAEVFRLLASADGSLAQIPQSHFAYVNVIRRQGTEEQRT  
FFFAELLAGRRFGNAQSEaGTKHVQDIRTRLTRRPDGSYALDGVKHYSTGALFADWIPVLARTEDDNLHVAYVPRDAPGVTVTDDWDGLGQRTTASGTVR  
LADVAVPADRVLPHHLTFQGPQLHGAVAQLLHAAIDAGIAAGALAEAAEFVRTKSRPWFESGAETAVEDPLLVQRFGELALAVRASEALLSEAARTVDAA  
SAGLTDDSAAEASIAVAAAKVQAARTAVEVGSALFEVSGTRAALDSLNLHRHWRDARTHTLHDPTRWKVQHIGRHVLTGTRP
  
> UniRef90\_A9BUV8\_38\_419 | Acyl-CoA dehydrogenase type 2 domain n=22 Tax=Burkholderiales TaxID=80840 RepID=A9BUV8\_DELAS | E\_val=1e-60   
EALEAARSFAALIAPGALARDRDRLLPWQEVEQFSQSGLWGITIPREYGGAGVSTATLTRAIALIAAADGNFGHIPQNHYYSLEVLRVGGSPAQKAFFYD  
RVLRGERLGNALAEIGHRDFQRRTRLLRDSGGWFVQGRKFYCTGALFAHWIPTLVSAQEEAPGQGErMYLVFIPRDAAGVTVTDDWDGFGQRVTGSGSVQ  
FDRVRVEPEWVVPFTSSFERPTTIGPFAQIIHAALDAGIGHGALQAALPFIREHARPWVDAGVASATQDPLLLQQLGNVHVRLRAADALVARAALAVDAA  
QRTPTDDSVAAASVAVAQAKALSTSASLLAGSKLFELAGTASTLAGQGLDRFWRNARTHTLHDPVRWKYHAVGNYVLNGIRP
  
> UniRef90\_A0A1N6TB45\_32\_407 | Acyl-CoA dehydrogenase n=3 Tax=Pseudacidovorax TaxID=433923 RepID=A0A1N6TB45\_9BURK | E\_val=1.7e-60   
AAALRPLFARDAAERDRAGGRPSAQIALLRQEGLLALLLPTAVGGEAQPWSTALRITRLLSQVDSSVGHLYGYHYVSLLGLGLRQVPAQVEPLWRRSAQA  
QWFWGNTANSFSRSLFGRREGDHFVLDGFRPFTSGSHVADWLSIAWEDAATGERRTAAIEAGREGVVIEDDWDGIGQRQTGSGRVSFHGVRVHADEVLGP  
PAPLDPTRAPAEPWRTVVPMlQQSVLLNVFVGSAQGAVRTARDTLLAAPASRRLQHDPGVQRLLGELWSRTRAATALADRALRALDAATARGVHLTAEER  
GEAAVAIAAANVLAGEVALQASTGLLEALGPLAADHGAGLDRFWRNVRIHTLHNPAEYKVRNVGRHFLSGQPPAPG
  
> UniRef90\_A0A381IE41\_24\_406 | Flavin-dependent monooxygenase, oxygenase subunit HsaA n=57 Tax=Burkholderiaceae TaxID=119060 Re | E\_val=2.6e-60   
ASDAEALDVARALAARLAQGAAERDRERRLPYDEVDWFSQSGLWAITVPNAYGGAGVSHVTLTEVAKIVAAADPSLGQLPQNHFGLVDVIALTGTDEQKR  
FFFGEILKGKRFGNGFSEKGTKNVLDLKTKVIRDGDGYRVDGTKFYSTGALFAHYVPVLGIDDDRKGWLAYIPKGTPGLSVIDDWSGFGQRTTASGTVRL  
DNVRVPASHVFPAHRVSDQPTLNGPLSQIIQAAIDAGIAHAALDDTLRFVRERSRPWIDSGVERAADDPLTIRETGRLVIHLHAADALLERAARTLDEIA  
AQSEITEDDVARASVAVGEAKVLTTEIALLASEKLFELAGTQATLAEHGLDRHWRNARTHTLHDPVRWKYHLVGNYYLNGVAP
  
> UniRef90\_A0A3M8TJ33\_18\_397 | Acyl-CoA dehydrogenase n=4 Tax=Streptomyces TaxID=1883 RepID=A0A3M8TJ33\_9ACTN | E\_val=3.4e-60   
QVAHEAADDLATDAVVRDQAGKTPFDEVSRLREAGLLSLLIPVESGGGGADWPTAHAVIRTIAAADGAIGQVLGCHYLLSSSARFFGGPGLAARVERESA  
AGQWcWGGGLASVEPRLTLTPGPGGYVLNGRQGYATGVLVADRLAVRADHSETGEPLAVLVDARRPGVVAGGDgGDTFGQRLAAGGSVEFDAVAVESDAV  
LGPLSSDDGTMSPYAGLSAPTGRLVSAQICLGIAQGVLAEAREYTRAADVPWRLDGPSYgSPRNPYVLTTYGELTVATFSASALADQALEALDCGLARGE  
DLTDDECAEITMLAAAAEAAAAGAAQDVTARALDVLGARSAASAYGFDRFWRNVRTHTLCVPVAPRLQEVGDYFLHGEHP
  
> UniRef90\_A0A1H3JL41\_4\_373 | Acyl-CoA dehydrogenase n=2 Tax=Saccharopolyspora TaxID=1835 RepID=A0A1H3JL41\_9PSEU | E\_val=5e-60   
ASAKAVADRLRADAADRDRANRRPVEEVELLRGAGLLVIPPDDHVTTHAVTRIVAAADPSIGHLLGYHYLHLWRAGLFGNPEAAARMRRRTAEQGlFWAG  
VSSPPNPADAGLSMTTVDGGFLVNGRRTFVTGAAVADRLVASAPDGSGEIRTFLVDARAPGIGHPDDWDNIGQRLSASGSIVFDDVLIDAADILGSPPSG  
DVRISLTSLAFQAILAQICVAIAEGALAEAADYTRRRARPWLLSGAAAAAEDPYVLAGYGELVAGVRAAGLLADHGTAALQDAADRGAELTAEQRGAAAA  
TISAAKIVATRTANETTAGIFEFLGARATAGSFGFDRFWRNARTLTLHDPVVYKARELGAHYLTGEPPEP
  
> UniRef90\_A0A507AW89\_49\_427 | Uncharacterized protein n=1 Tax=Phialemoniopsis curvata TaxID=1093900 RepID=A0A507AW89\_9PEZI | E\_val=7.9e-60   
GLRERENKSPRAEVALLKHSGLLKVLGLKKYGGGEQPWSVGYKVIRKVAEGDGSLGMLLGYHLLWSTTANVVGTAEQADRIQKLIISNNYFVGGAVNPRD  
QDLRITSDGDDIVFNGFKFFNTGGVVSDLTVLEGSYEDTNDHIFAFVKTEQPGIQFSHDWNNVGLRLTESGSVKIDKVKAPWADALGWDAAKKkpdPSIL  
GIPFATLLLPTIQLVFSNFYIGIAWGALSEASAYTNKSTRAWPYGGDNKerPQDEFYILSTYGNFLAHLRAVTALADKAGEEVTSLYNNsGSAedrskVT  
AEARGEAAVWVASVKVVATDTGLRVTSGLFEVTGAKATATKVGLDRFWRDVRTHTLHDPVSYKNRELGRYQLLRELPEP
  
> UniRef90\_A0A1X1SEN2\_20\_406 | Uncharacterized protein n=1 Tax=Mycolicibacterium confluentis TaxID=28047 RepID=A0A1X1SEN2\_9MYCO | E\_val=1.2e-59   
VRQELARTAVARDRAAGTPTFEIGLLKEAGLLGIFIPEEHGGGGADFSQAAAVVGEIARADSSVAHILLYHYFGSIAGTRGENGFLgprrAQRIARENLF  
HGTVAQAAYPPLISADPTPGGFVLNGSKPFTSGAAVSDVLLAWvqfgtGSVLNGADVSGQLATVHIEGGAAGVSFGDDWDNVGQRLTVSGTTTLTGVEIP  
VDAVIGYGYGVAPAEPADHLDVLYMYAGFAAIFTGIARGAFDEAADYTRARSRPWVETNYASAQADPLVLERFGQLWTLVQSAEALTARALAAVDEVRDR  
GAGLTWEQRAEAVTLINAARTHAGEIAITIASRLFEVTGARSTAAPEGLDRFWRNARTLTLHDPLHHKQTQIGDYVLNGVAPAPGFY
  
> UniRef90\_A0A2D3UFX4\_26\_403 | Acyl-CoA dehydrogenase n=2 Tax=Streptomyces TaxID=1883 RepID=A0A2D3UFX4\_STRC0 | E\_val=1.6e-59   
RVARETADDLATDAVAREQAGKAPFDEVSRLREAGLLTLLIPAELGGGGEDWPTAYAVVREIATADGAIGQLLGCHYFLSWSARFFIEPALAAQVERKST  
REQWCWGGGFARQELPLTLARTADGYVLDGRQSYATGVLVADRLAVRAVRADTGEPLAVVVDPTRHGVGIDDDADTFGQRLAAGGSVEFDAVPVAADDVL  
GSLSTDEDVLSPLAALASPLGRLLSVQLLLGMAEGVLAEAREYSRAGHSPSLPAWPASSPQDPQVLTAYGELTVLTRSASALADQAQEAVRGGLARGEDL  
TYDEYAEISVLVAMAEAAASRAAQESTARALDILGARSTSSRLGFDRFWRNARTHTLYEPVAHRLRDVGDYFLNGAHP
  
> UniRef90\_A0A089X0S9\_26\_403 | Type 2 domain-containing Acyl-CoA dehydrogenase n=3 Tax=Streptomyces TaxID=1883 RepID=A0A089X0S9 | E\_val=2e-59   
RVIRETADDLATDAVEREQAGKAPFDEVSRLREAGLLTLLIPAELGGGGADWPTAYAAVREIAAADGAVAQLLGSHTFLSWSARFLGEPALAARIGRRSA  
AEQWCWGGGLARQEPALALTPSAGGQVLDGRQSYVTGVLVADRVAVRAVRADSAEPVAVLVDPAAPGVRVDNDAEAFGQRLAAGGSVEFDAVPVAADDIL  
GSLSTEEDALSPRTALISPVGRLLSVQLRLGMAEGVLAEARDYSRTGHAPWHPAWPVGSPHDPQVLTTFGELTVLVRAASALADQALAAVCAGLSLGDDL  
TFEEHADIAVLVAMAEAAAARAAQESTTRALDVVGARSTPARLGLDRFWRDARTHTLYEPVAPRLRDVGDYFLNGAHP
  
> UniRef90\_A0A2W5T1D0\_29\_413 | Monooxygenase n=1 Tax=Leifsonia xyli TaxID=1575 RepID=A0A2W5T1D0\_9MICO | E\_val=2.4e-59   
LARAEEVSQILAADAVERDRAGASPVDEVALLKNSGLVTLLGPAEHGGGGQQWATAYRVIRIIARGDGSIGQLLGYHYLWAWAARLVATDAQIAAVEKLY  
TENRYLFGGAVNPRDADLTVRDEGDELVFTGRKSFSTGGVVSDLTVLEGVLEGTDTHVFAIVPTDQPGIVFGRDWDSLGQRLTESGSVRIEGVRVPWADA  
AGFVDKVFQPLVYGTLNVPAIQLVFANFYLGIAEGALERAAAYTRETTRAWPYGGDekQRASEEWYILEGYGGLQSKLWADAALIDAVgdEISVLLHAPR  
EQLTERSRGEVAVRIAAAKLRISDDGLETATKVFELTGARASANSVGLDIFWRNLRTHSLHDPIPYKRREVGVFALLGEVPEPSW
  
> UniRef90\_A0A1C4QGR9\_7\_388 | Sulfur acquisition oxidoreductase, SfnB family n=1 Tax=Streptomyces sp. SolWspMP-5a-2 TaxID=1838 | E\_val=3.9e-59   
ADDQEALAVARALAEEFRAGASERDRERRLPRAELERLSASGLLAVTVPAAHGGADVGAGTLAEIFRLLASADPSLAQIPQSHFVYVNVIRRQGTPEQRR  
FFFGELLAGRRLGNAQSEAgTRHVQDIGTRLTPRPDGSLLLTGEKHYSTGALFADWIPVLARAADDALHVAYVPRDAPGLTVVDDWDGMGQRTTASGTVR  
LTGVVVPADRVLPHHLTFRGPQLHGATAQLLHAAIDVGIADGALAEAVSFVRTRSRPWFESGADTAAEDPLVIQRFGELGVTTRASRALLREAARAVEEA  
RADLTDDTAAEASVAVAAAKAHAARTAVEVADALFDLAGTRSALDSLNLHRHWRDARTHTLHDPARWKIQHIGRHLLNGTRP
  
> UniRef90\_A0A101R4L6\_7\_388 | SfnB family sulfur acquisition oxidoreductase n=3 Tax=Streptomyces TaxID=1883 RepID=A0A101R4L6\_9 | E\_val=9.3e-59   
ADDAEALAVAAELADAFRAGAARRDAERVLPRAELDRLSASGLLAVTVPAEHGGADVGAATLAEVFRLLGAADPSLAQIPQSHFVYVNVIRRQGTTEQRT  
FFFAEVLAGRRFGNAQSEaGTKHVQDIRTRLTPRPDGSYRLDGVKHYSTGALFADWIPVLARADDDDLHVAYVPRDAPGLTVVDDWDGMGQRTTASGTVH  
LEGVEVPADRVLPHHLTFRGPQLHGAVAQLLHAAIDAGIASGALTEAAAFVRTRSRPWFESGADTAVEDPLLVQRFGELALRVRASEALLKEAARAVDAA  
RAGLTDDSAAEASLAVAAAKVQAAETAVEVASALFEVSGTRSALDSLNLHRYWRDARTHTLHDPPRWKLQHLGRHVLTGARP
  
> UniRef90\_A0A4D4LHU8\_7\_388 | SfnB family sulfur acquisition oxidoreductase n=2 Tax=Streptomyces avermitilis TaxID=33903 RepID | E\_val=1.8e-58   
ADDAEALSVAAALADEFRAGASGRDAERRLPRAELDRLSASGLLAVTVPAEHGGADVRQETLAEIFRLLASADASLAQIPQNHFVYVNVIRRQGIEEQRK  
FFFAEVLAGRRFGNAQSEaGTRHVQDIRTRLAPRPDGSYLLTGVKHYSTGALFADWIPVLARAENDDLHVAYVPRDADGVTVVDDWDGMGQRTTASGTVR  
LEAVSVPADRVLPHHLTFQGPQLHGAVAQLLHAAIDAGIAAGALAEAAEFVRTKSRPWFESGAETAAEDPLLIQRFGELAIQVRASDALLVAAARAVDAA  
RADLTDDSAAEASLAVAAAKVHTASTAVEVAGALFEVSGTRSALNSLNLHRHWRDARTHTLHDPARWKIQHIGRHVLNGTKP
  
> UniRef90\_L1KK35\_6\_389 | Acyl-CoA dehydrogenase, C-terminal domain protein n=12 Tax=Streptomyces TaxID=1883 RepID=L1KK35\_9ACT | E\_val=2.7e-58   
DDAEALAVAAELAADFRKGAAERDARRRLPHAELERLSASGLLAVTVPAEFGGADVRAETLAEIFRLLAAVDASLAQIPQSHFVYVNVLRRQGTHEQQEF  
FLGEVLRGKRFGNAQSEaGTQHVQDIRTRLTRLPDGSYALDGVKHYSTGALFAHWIPVLARTEDDTLHVAYVPRDAPGLTVVDDWDGMGQRTTASGTVRL  
ESVPVPADRVVPHHLTFRGPQLHGAVAQLLHAAIDAGIAAGALADAVEFVRTKSRPWFESvdeGHETAAEDPLLIQRFGELAIRVRAAEALLREAARAVD  
DARADLTDDSAAEASIAVAAAKVTAAEAAVEVGSALFEVAGTRSALDSLGLHRHWRDARTHTLHDPARWKVQHIGRYVLSGIKP
  
> UniRef90\_UPI0005601C6B\_13\_390 | SfnB family sulfur acquisition oxidoreductase n=1 Tax=Streptomyces yeochonensis TaxID=89050 R | E\_val=6.6e-58   
EALDVAARLAAGFRVDADTRDAERRLPRAELEKLSASGLLGITVPAEYGGAGVSARTLAEVLRLLATADGSLAQIPQNHFVYVNVLRRQGTAAQRRFFFA  
EVLAGRRFGNAQSEaGTKHVQDIRTRLESRPDGSYLLDGEKHYSTGALFAHWIPVLARAADDALHVAYVAADTPGVTVVDDWDGMGQRTTASGTVRLAGV  
AVPADRVVPHHLTFRGPQLHGAVAQLLHAAIDAGIAAAALGAAVEFVRTKSRPWFESGFETAAEDPLLIQRFGELSLAVRSSDALLRAAADAVDAAEADL  
TDDSAAEASIAVAAAKVQAAAAAVDTGSALFEVAGTRSALDSLNLHRYWRDARTHTLHDPARWKVQHIGRYVLNGTRP
  
> UniRef90\_A0A1H9UQJ9\_6\_361 | Acyl-CoA dehydrogenase n=1 Tax=Lechevalieria xinjiangensis TaxID=402600 RepID=A0A1H9UQJ9\_9PSEU | E\_val=1.7e-57   
DVARDVAATLRSDAADRDRANQPPAKEVELLRHSGLLDVADWDLQQRIGRIVGAADANVGHLLGYHYLQIWRSGLFDAPFSARPGQFWAGVSNPLDAALE  
LTPAGDGYVLNGRKTFATGAAVADRLVVSATRTDNGEKLTFLLDGKADGITYLDDWDNIGQRLTASGGVSFEDVHVTEVLGVQPAGEDPRISLAAIGFQL  
VLAQLYVAIAEGALQEAADYTRTKTRPWFVSGVEAATEDPYIVVAYGEMMSQTKAAGLLVDEASRQLRDASELGRKLTAAKRAEVAVTISAAKVVSTKLV  
NEITSRIFEQVGARGTAAKHGVDRFWRNARTLTLHDPVVYKAREVGEHFLTGARPA
  
> UniRef90\_A0A3D9JKY9\_12\_401 | Alkylation response protein AidB-like acyl-CoA dehydrogenase n=3 Tax=Streptomyces TaxID=1883 Rep | E\_val=2.9e-57   
GAGQADWLHIAREMADDLATDAVEREQAGKAPLDEVARLRESGLLTLLAPAEHGGGGADWRTAYAVVRTVAAADGAIGHLLGNHYFLSFSARFFADPTRT  
ARIERESTAALWcWGGGIASHEPPLVLTPTGDGYLLDGYQRYAAGAGIADRLVVRAVGYGTGEPLAVLVDPTRPGVVRGSGGDTFGQRLAAEGGVEFDSV  
LVGADAVLGflSPDEDVLSPFASLAAPTARLASAQFCLGIAEGLLAEVHEHGRAVRSPWQPFSPEpwpdSPPQDPYALTVYGEFAVAARAASALADQAVE  
ALLRGLAQREDLDDEECAEITVLASAAEAAASRAAQEITTRALDVIGADAASVRHGFDRFWRNARTHTLREPVAHRLREIGDYFLNGAHP
  
> UniRef90\_D9X7Q2\_26\_403 | FMNH2-dependent monooxygenase n=1 Tax=Streptomyces viridochromogenes (strain DSM 40736 / JCM 4977 / | E\_val=4.2e-57   
RVARETADDLATDAVTREQAGKPPFDEVSRLRESGLLTLLVPAGLGGGGGDWSTAYAVVREIAAADGAIGQLLGCHYVMSWSARFLTEPDLAVRLEQRST  
AEQWcWGGGLARQEPALTIARNATGYVLNGRQSYAAGALVADRLTVRAVRADTGEPLALVVDPALPGVLTDGDADPFGQRLAAGGSVEFDAVPVSAHDVL  
GSLAADEDVLSPLTSMVSPVGRLLSVQLRLGMAEGVLAEAREYSRTGLSHWHPDWPVGSPQDPQVLTAYGELTVLTRSASALADQARQAVLDGLARGEDL  
GYEEYAEISVLVAMAEAAASKAAQESTARALDIIGARSASARLGFDRFWRNARTHTLYEPVAHRLRDVGDYFLNGAHP
  
> UniRef90\_A0A1X1DXJ3\_20\_398 | SfnB family sulfur acquisition oxidoreductase n=3 Tax=Pantoea TaxID=53335 RepID=A0A1X1DXJ3\_9GAMM | E\_val=1.4e-56   
DQAIQAAHSLAEQAKPGAVERDQQRIYPVDLLNEFTRLGLGSISVPRRFGGGGLDYQTLAEVFRIISASDPSLGQIPQNHFGLIQFILGEGEPEQQETLL  
QAVVNGHRLGNGGPEKNtRHTRDVQARLSGKGDSRFLTGEKFYSTGALFADILVTTALQDDDRPVMAFIPLPAAGIEIVDDWSGMGQRTTASGTVRLNQV  
AVDPAWIIPLPSPEKPTLRGAVSQLIQAAIDAGIAQGALDDALAFVRDHSRPWVDAGVARNADDPYILADIGRISTELSAANALLRRAARVLDAIDPAAL  
TAENSAAASIAVAEAKVLTTEIALRASEKLLEWGGSRATLLQHGLDRHWRNARTHTLHDPVRWKTHAIGNYYLNAVYPA
  
